# Thermodynamics of firms' growth

**DOI:** 10.1098/rsif.2015.0789

**Published:** 2015-11-06

**Authors:** Eduardo Zambrano, Alberto Hernando, Aurelio Fernández Bariviera, Ricardo Hernando, Angelo Plastino

**Affiliations:** 1 Social Thermodynamics Applied Research (SThAR), EPFL Innovation Park, Bâtiment C, 1015 Lausanne, Switzerland; 2 Department of Business, Universitat Rovira i Virgili, Av. Universitat 1, 43204 Reus, Spain; 3 National University of La Plata, Physics Institute (IFLP-CCT-CONICET) C.C.737, 1900 La Plata, Argentina

**Keywords:** firms' growth, Zipf's law, thermodynamics

## Abstract

The distribution of firms' growth and firms' sizes is a topic under intense scrutiny. In this paper, we show that a thermodynamic model based on the maximum entropy principle, with dynamical prior information, can be constructed that adequately describes the dynamics and distribution of firms' growth. Our theoretical framework is tested against a comprehensive database of Spanish firms, which covers, to a very large extent, Spain's economic activity, with a total of 1 155 142 firms evolving along a full decade. We show that the empirical exponent of Pareto's law, a rule often observed in the rank distribution of large-size firms, is explained by the capacity of economic system for creating/destroying firms, and that can be used to measure the health of a capitalist-based economy. Indeed, our model predicts that when the exponent is larger than 1, creation of firms is favoured; when it is smaller than 1, destruction of firms is favoured instead; and when it equals 1 (matching Zipf's law), the system is in a full macroeconomic equilibrium, entailing ‘free’ creation and/or destruction of firms. For medium and smaller firm sizes, the dynamical regime changes, the whole distribution can no longer be fitted to a single simple analytical form and numerical prediction is required. Our model constitutes the basis for a full predictive framework regarding the economic evolution of an ensemble of firms. Such a structure can be potentially used to develop simulations and test hypothetical scenarios, such as economic crisis or the response to specific policy measures.

## 1. Introduction

Many natural, social and economic phenomena follow power laws. Their ubiquity has been previously ascertained in the distribution of financial or econometric values such as wealth and income, [1–8], or the size of cities [9–13], and even in human language and frequency of words [14–16], Internet networks [17] or scientific publications and citations [18–21], among many other human-related measurable observables. Finding a complete theory for describing this kind of systems seems an impractical task, given the huge amount of degrees of freedom involved in discussing these social systems. This notwithstanding, remarkable regularities were reported and studied, such as Zipf's law [22–25], or the celebrated Gibrat's law of proportional growth [26], which constitute important milestones on the quest for a unified framework that could mathematically describe predictable tendencies [7,10,27–29].

Firm size distributions (FSDs) are the outcome of the complex interaction among several economic forces. Entry of new firms, growth rates, business environment, government regulations, etc., may shape different FSDs. The underlying dynamics that drives the distribution of firms' sizes is still an issue under intense scrutiny. According to Gaffeo *et al*. [30], there is an active debate going on among industrial organization scholars, in which lognormal, Pareto, Weibull or a mixture of them compete for the best-fitting distributions of FSDs. One of the controversial issues is the very definition of ‘size’, which can be measured by different proxies, such as annual sales, number of employees, total assets, etc.

The seminal contribution by Gibrat [26] initiated a research line concerning the formal model that governs firms' sizes and industry structure. The introduction of a theoretical model that would underlie the industrial demography could be of great help for authorities interested in maintaining fair competence and antitrust policies or tracking wealth inequality [3,7].

Hart & Prais [31] find, using a database of large firms, that average growth rates and sizes are independent variables. Quandt [32] states that Pareto's distribution is often rejected when analysing industry subsectors. Other independent empirical studies, carried out by Simon & Bonnini [33], Mansfield [34] and Bottazzi & Secchi [35], among others, confirm that firms' growth rates are not related to firm size and that FSDs follow a lognormal distribution. Jacquemin & Cardon de Lichtbuer [36] study the degree of firms and industry concentration in the UK using Fortune's 200 largest industrial companies outside the USA, ranked according to sales. This study detects an increasing degree of concentration.

Kwasnicki [37] asserts that skewed size distributions could be found even in the absence of economies of scale, and that the shape of the distribution is the outcome of innovation in firms. In particular, according to his simulations, cost-improving innovations generate Pareto-like skewed distributions. This work also reconciles the finding by Ijiri & Simon [38] about the concavity towards the origin of log–log rank size plots. Such concavity could be produced by evolutionary forces and by innovation. Jovanovic [39] finds that rates of growth for smaller firms are larger and more variable than those for bigger firms. Similar results are found empirically for Dutch companies by Marsili [40]. On the contrary, Vining [41] had argued that the origin of the concavity is the existence of decreasing returns to scale.

Segal & Spivak [42] develop a theoretical model in which, under the presence of bankruptcy costs, the rate of growth of small firms is prone to be higher and more variable than that of larger firms. The same model also predicts that, for the largest firms, the sequence of growth rates is convergent, satisfying Gibrat's law, namely1.1
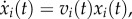
where *x_i_*(*t*) is the size of the *i*th firm at time *t*, 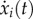 its change in time, and *v_i_*(*t*) a size-independent growth rate. This model is consistent with some previous empirical evidence, as that of Mansfield [34]. Sutton [43] has published a review of the literature on markets' structure, highlighting the current challenges concerning FSD modelling. During the 1990s, the interest in FSD increased with the availability of new databases. A drawback of early studies was a biased selection of firms. Typically, data comprised only publicly traded firms, i.e. the largest ones. In recent years, new, more comprehensive data sources became available.

Stanley *et al*. [44], used the Zipf-plot technique in order to verify fittings of selected data for US manufacturing firms and find a non-lognormal right tail. Shortly afterwards, Stanley *et al*. [45] encountered that the distribution of growth rates has an exponential form. Kattuman [46] studies intra-enterprise business size distributions, finding also a skewed distribution. Power-law decays were reported by Plerou *et al*. [47], looking for similarities between university research growth and business firms. Axtell [48], using census data for all US firms, encounters that the FSD is right-skewed, giving support for the workings of Pareto's law. A similar finding is due to Cabral & Mata [49] for Portuguese manufacturing firms, although a lognormal distribution underestimates the skewness of the distribution and is not suitable for its lower tail. In this line, Stanley and co-workers [50,51] find that, for pharmaceutical firms in 21 countries, and for US publicly traded firms, growth rates exhibit a central portion distributed according to a Laplace distribution, with power-law tails. Palestrini [52] agrees with a power-law distribution for firm sizes, although he models firm growth as a Laplace distribution, which could change over business cycles. Zhang *et al*. [53] find Zipf's distributions for the biggest Chinese companies, and propose an explanation based on an AK model of economic growth [54].

According to Riccaboni *et al*. [55], the simultaneous study of firm sizes and growth presents an intrinsic difficulty, arising from two facts: (i) the size distribution follows a Pareto law and (ii) firms' growth rate is independent of firm size. This latter property is known as the ‘law of proportionate effect’. Growiec *et al*. [56] study firms' growth and size distributions using firms' business units as units of measurements. This study reveals that the size of products follows a lognormal distribution, whereas firm sizes decay as a power law.

Gaffeo *et al*. [30], using data from 38 European countries, find that log mean and log variance size are linearly related at sectoral levels, and that the strength of this relationship varies among countries. Di Giovanni *et al*. [57] find that the exponent of the power law for French exporting firms is lower than that for non-exporting firms, raising an argument on the influence of firms' heterogeneity in the industrial demography. Additionally, Gallegati & Palestrini [58] and Segarra & Teruel [59] show that sampling sizes influence the power-law distribution.

One can fairly assert that the concomitant literature has not yet reached a consensus regarding what model could best fit empirical data. An overview of several alternative models is detailed in [60], and references therein. As shown in the above literature review, previous attempts to model growth and sizes of firms have not been entirely successful. In particular, there is a dispute concerning the underlying stochastic process that steers FSD.

A possible solution in terms of agent-based models was proposed [61]. These models are remarkable as descriptive tools, but they do not furnish an overall panorama because they are single-purpose models. Besides, they are sensitive to the initial conditions, and, in some cases, their outcome depends on the length of the simulation time. Recently, success and failure of firms were studied by Daepp *et al*. [62], showing that mortality rates are independent of firms' age.

The aim of this paper is twofold. First, to develop a thermodynamic-like theoretical model, able to capture typical features of firms' distributions. We try to uncover the putative universal nature of FSD, which could be characterized by general laws, independent of ‘microscopic’ details. Second, to validate our theoretical model using an extensive database of Spanish manufacturing firms during a long time period.

This paper contributes to the literature in several aspects. (i) It shows how first dynamical and thermodynamic principles, extensively used in physics, can be applied to economic systems. (ii) It presents a general mathematical framework that provides explanations for the stochastic distribution of firms' sizes. The understanding of FSD is relevant for economic policy because it deals with market concentration, and thus with competition and antitrust policy measures—for example, Naldi [63] exhibits a relationship between Zipf's law and some concentration indices, and deviations from Zipf's law in the FSD can be used for policy diagnostics [64]. (iii) Additionally, our model is tested with an extensive sample of Spanish firms during 10 years, displaying interesting empirical properties of the Spanish economy.

The paper is organized as follows. First, we present the theoretical framework and perform numerical experiments to validate our analytical approach. Afterwards, we undertake empirical applications to Spanish firms. Finally, we draw some conclusions from our work.

## 2. Theoretical framework

Our theoretical framework is based on two fundamental hypotheses:

(1) a microeconomic dynamical hypothesis for individual firm growth and
(2) the use of the maximum entropy principle, with dynamical prior information, for describing macroeconomic equilibrium.

### 2.1. Microdynamics

As our microeconomical hypothesis, we use the *earnings before interest, taxes, depreciation and amortization* (EBITDA) indicator as proxy for the size of a firm, and Gibrat's law of proportional growth (equation (1.1)) as the main mechanism underlying firms' size evolution. A finite-size term (FST) owing to the central limit theorem [65] becomes dominant for medium and small sizes, being proportional to the square root of the size. In addition to these two terms, we also assume that non-proportional forces become eventually effective for the smallest values. Thus, our full dynamical equation is written as2.1
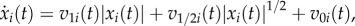
where *v_qi_*(*t*) (*q* = 1, 1/2 and 0) are independent growth rates. It is expected that the growth rates will be of a stochastic nature. Thus, a *temperature* can be defined from their variance *T_q_* = var[*v_q_*]. Accordingly, assuming that the variation in the growth rates is much larger than the variation in the observable *x*—as done in [12,28]—the variance of the growth for several realizations for positive (+) and negative (−) values of *x*, respectively, becomes2.2
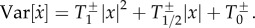


**Figure 1.**
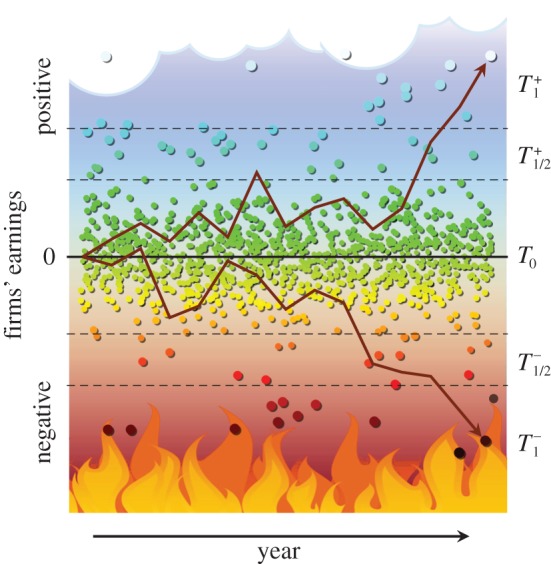
Conceptual sketch of firms' dynamics: heaven (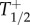 and 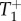 regimens), hell (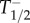 and 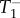 regimens) and purgatory (*T*_0_ regimen, both positive and negative), according to equations (2.1) and (2.2). Firms (represented here by dots) evolve as random walkers in time—similar to particles in a gas—according to the regime defined by their earnings (delimited by dashed horizontal lines) and the empirical temperature at that regime: linear growth at purgatory, and proportional growth at heaven and hell with a variance defined by the temperature in each case. Brown solid arrows show the path for two of the firms who eventually evolve from purgatory at their birth to heaven and hell respectively at the present time.

This equation defines six regimes (three for negative and three for positive EBITDA) according to the size: small sizes 
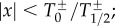
 medium sizes 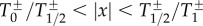; and large sizes 
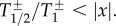
 Because of the existence of the non-proportional term, *x* is allowed to move across negative and positive values. In principle, we will assume that the set of temperatures at the negative domain is independent of that at the positive one. Because all these temperatures can be measured from the raw data, their properties can be empirically determined. In figure 1, we display a conceptual sketch of the ensemble of firms evolving in time as random walkers along the different regimes, with a corresponding temperature and dynamics for each of them.

### 2.2. MaxEnt principle

For an ensemble of firms following equation (1.1), we assume that dynamical equilibrium is asymptotically reached when some macroscopic constraints are obeyed. As such constraints, we cite here the average total number of firms *N* and the typical wealthiness of a given particular region, or any other objective observable. In view of the success of an entropic procedure for describing equilibrium distributions in other social systems (e.g. city population distributions), we take as our macroeconomic hypothesis the principle of maximum entropy (MaxEnt) with dynamical prior information [12,65–67] to predict the equilibrium density of the system. We focus our analytical derivation on that particular regime that has received greatest attention in the literature: the proportional growth one: 
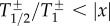
 for the largest sizes. According to [12], the entropy of a system following Gibrat's law is measured in terms of the new dynamical variable 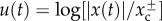 (independently for positive and negative domains, and where 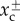 is some reference value, in our case, the transition size 
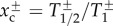
) which linearizes the dynamical equation as 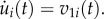 Thus, we write the macroscopic entropy for the system's density distribution *ρ*(*u*) for *N* firms as2.3
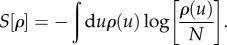


The equilibrium density is obtained by extremizing *S* under the empirical constraints [66,67], such as the total number of firms and the minimum size of a firms, among others. Lacking them, as sometimes happens in physics, we will use a symmetry criterion [68]: employ constraints that preserve a symmetry of scale of *x*(*t*), i.e. translation symmetry in *u*(*t*). For this purpose, we define an *energy function*, 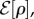 that depends on powers of the dynamical variable *u*, namely2.4
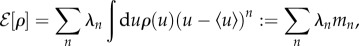
where *m_n_* are the central moments of *ρ* and *λ_n_* the coupling constants. The maximization problem is written as 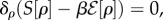 where *β* is a Lagrange multiplier (*β*, *λ_n_* become then the multipliers for each term), and the general solution is of the form2.5
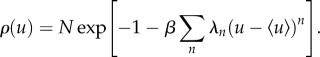


The values of the multipliers are obtained by solving the system of Lagrange equations, 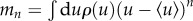 for the distribution of equation (2.5).

### 2.3. Connection with thermodynamics

We consider, for simplicity and separately for negative and positive domains (no super-indices are used for the temperature), only the first two moments *n* = 0 (a constraint on the average total number of firms *m*_0_ = *N*) and *n* = 1 (a constraint on the mean value 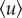 written as *m*_1_ = 0). Because the equations are formally equivalent to those found in thermodynamics, and traditionally the multipliers associated with these constraints are [69,70] 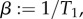 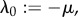 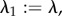 we have a thermodynamic potential2.6
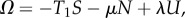
where 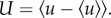 The variational problem becomes 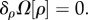 We obtain the distribution2.7
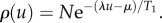


The distribution is cast in terms of the observable *x* as2.8
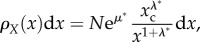
where 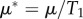 and 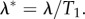 Accordingly, we obtain a power-law density. Useful for analysing the empirical data is the complementary of the cumulative distribution 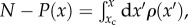 that reads2.9
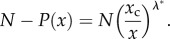


The solutions of the Lagrange equations lead to2.10
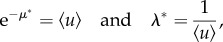
and to the equation of state2.11
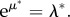


This is the relevant equation for interpreting the empirical data, because λ* can be measured from the data and μ* can be interpreted, thanks to the thermodynamic analogy.

Indeed, comparing our results with those of a physical system, one can identify *μ* with the chemical potential. We interpret *μ* as the ‘cost’ for including/creating or excluding/extinguishing firms in the proportional large-size regime. Following MaxEnt [12,65,66], the system is in contact with a reservoir of firms, and tends to minimize *Ω*. Because 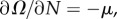 *Ω* decreases for *μ* > 0 when a new firm enters in the proportional regime, and thus making more likely the emergence of a flow of firms entering the system. However, for *μ* < 0, any new firm will increase the value of *Ω*, allowing for a flow of firms exiting the system. In the particular case *μ* = 0, there is no cost for the flow of firms, in what we expect to be an equilibrium, stable and healthy situation for a capitalist economy.

The thermodynamic variable *λ* defines the exponent of the distribution, and can be interpreted as a measure of the typical wealth of a region. Specifically, it determines the scale of the size of firms, because it constrains the geometrical mean of *x* at the proportional regime. Indeed, the use of the geometrical mean instead of the mean is common for systems with scale invariance, where long-tailed distributions have undefined moments but well-defined log moments [71,72]. This value will change from one economy to another.

Thanks to the equation of state, equation (2.11), we can provide an intuitive, physically based interpretation of that exponent:

— for λ* < 1 (μ* < 0), the system favours the extinction of firms;
— for λ* > 1 (μ* > 0), the system favours the creation of firms; and
— for λ* = 1 (μ* = 0), the system freely creates and extinguishes firms.

This last particular case corresponds to the Zipf law distribution, namely2.12
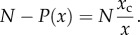


## 3. Numerical experiments

With the aim of testing our theoretical procedure, we have performed numerical experiments in terms of random walkers via a Monte Carlo (MC) simulation. At the initial time, the *N* random walkers are randomly located using a uniform distribution. We assume independent stochastic Wiener coefficients for different firms, within each of the regimes, or3.1
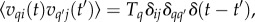
where *q* defines the specific dynamical regime as in equation (2.1). In order to make explicit the mechanisms that govern the dynamics, we will use a reduced approach where the walkers evolve following only the dominant term according to the size *x*. Therefore, instead of simulating the whole dynamics, i.e. equation (2.1), we aim to understand the particular contribution of each term in that equation. Henceforth, we will focus on the interplay between the linear and proportional growth regimes, disregarding the intermediary regime. To achieve this goal, we use the following equation for the microscopic dynamics:3.2
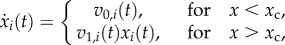
where *x*_c_ defines the border between the linear and the proportional regimes. Because the number of walkers in the proportional regime is not constrained, we follow here the well-known recipe for a grand canonical ensemble [69,70], where *μ* is fixed and the fluctuation in the number of walkers is determined by the probabilities of including (*P*_+_) or extracting (*P*_−_) a walker as3.3
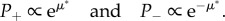


**Figure 2.**
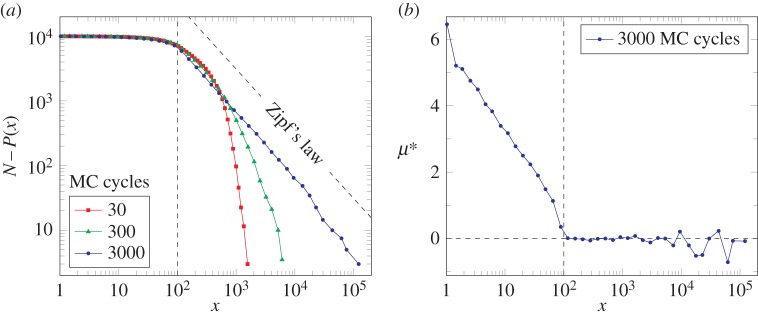
Cumulative distribution (*a*) and chemical potential μ* as measured from the distribution (*b*) for the simplified dynamics equation (3.2). We use *N* = 10^4^ random walkers with transition to proportional growth at *x*_c_ = 100 (dashed vertical line). The equilibrium distribution is reached in general after approximately 3000 MC cycles independently of the initial distribution, with an upper tail starting at *x*_c_ following Zipf's law—as expected for the given thermodynamic conditions (see text). At the proportional-growth regime (*x* > *x*_c_), the measure of the chemical potential shows numerical fluctuations around the global thermodynamic value μ* = 0, and diverges outside the regime (*x* < *x*_c_).

According to these probabilities, in an ensemble with μ* = 0 any walker may leave or enter the system without any restriction. Additionally, following equation (2.10), the constraint 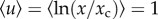 should be obeyed. We have performed several realizations with different initial conditions, and let the system evolve until reaching equilibrium. In figure 2, we show the size distribution equation (2.9) for different simulation times measured in MC steps. Here, we choose *x*_c_ = 100. We see that the equilibrium distribution (for approx. 3000 MC cycles) follows Zipf's law: for large values, the complementary of the cumulative distribution follows equation (3.3), as predicted by our thermodynamic framework. The distribution deviates from the analytical result as the size of *x* reaches the transition critical valued *x*_c_. Remarkably, we find that the equilibrium does not depend on the initial conditions.

For an independent measurement of the chemical potential directly from the distribution—for both validating the theoretical approach and testing the measure procedure for empirical data—we proceed as follows: (i) we compute the derivative of 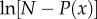 in equation (2.9) to obtain a measure of λ* and (ii) we use the equation of state (2.11) to obtain μ*. This measurement only has sense in the proportional growth regime *x*_c_ < |*x*|. One expects μ* to be constant in this domain (up to numerical fluctuations) and diverge outside it. We show in figure 2 the chemical potential for the walkers' equilibrium distribution. We see that, up to some fluctuations, the constraint μ* = 0 is respected for the proportional growth regime and blows up after the transition |*x*| < *x*_c_. In view of these results, we succeeded in numerically validating our analytical procedure.

## 4. Empirical application

In order to empirically verify our theoretical model, we consider the Spanish SABI database [73,74], which is a comprehensive one for all firms that have the obligation to disclose balance sheets in the Spanish Mercantile Register. Our sample consists of 1 155 142 firms along a decade, with more than 500 000 firms per year. We select those firms which have been active at any time during the past 10 years and use, as our observable *x_i_*(*t*) for the *i*th firm at year *t*, the reported value for EBITDA. Indeed, this quantity is widely employed for assessing companies' performances. It is homogeneous across companies and is not affected by different forms of financing. We believe that our proxy for size is a clear indicator of both corporate performance and size.

### 4.1. Microdynamics

**Figure 3.**
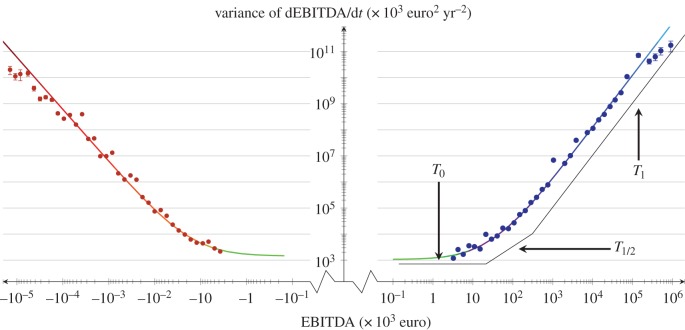
Variance of firms' growth dEBITDA/d*t* for the set of all Spanish firms in 2009, as a function of their EBITDA. Solid red and blue lines correspond to the fit following equation (2.1) for firms with negative and positive EBITDA, respectively. The black solid line sketches the trend regime: linear for small-firm sizes, FST for medium and proportional growth for the largest sizes—a pattern that specularly appears as well for negative EBITDA. The measured temperatures for positive EBITDA are 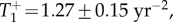 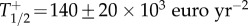 and 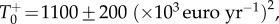 and for negative EBITDA 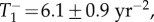 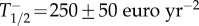 and 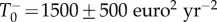.

**Figure 4.**
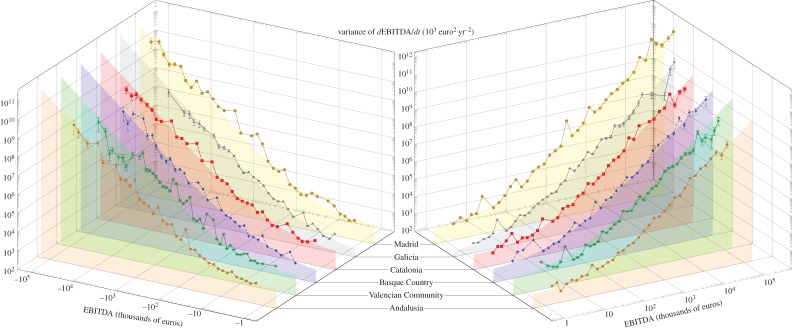
Variance of firms' growth dEBITDA/d*t* versus firms' size for some Spanish autonomous communities in 2009: Madrid, Galicia, Catalonia, Basque Country, Valencia and Andalusia. The bottom-filled curves correspond to the fit according to equation (2.1), in similar fashion as figure 3.

**Figure 5.**
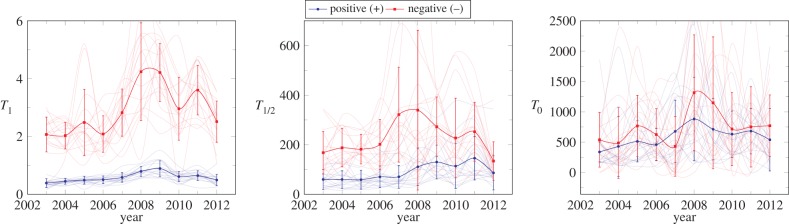
Temperatures *T*_1_, *T*_1/2_ and *T*_0_ for positive (blue) and negative (red) EBITDA—as a function of the year. Faded lines represent the temperature evolution for every autonomous community, whereas bold blue and red lines represent the Spanish mean temperature and error bars its standard variation. Hell is systematically hotter than heaven for proportional regime (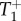 and 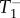) and FST (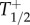 and 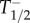), whereas the temperature at purgatory can be considered the same under the standard variation (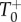 and 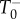).

We first test our microscopic dynamical hypothesis by measuring the variance of the EBITDA growth for each year and separately for positive and negative domains. We first analyse all the Spanish firms in the same set, displaying in figure 3 the dependence of the growth variance on the EBITDA for the year 2009. We find a remarkable match to equation (2.1) for both positive and negative domains. The transition to proportional growth takes place, in this case, at 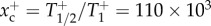 euros and 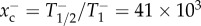 euros. Additionally, in both domains, the linear regime temperature 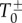 has approximately the same value within the error bars (1190 ± 200 versus 1500 ± 500 (× 10^3^ euro per year)^2^). As shown in the electronic supplementary material, all the available data for 10 years match equation (2.1), with slightly changing temperatures. A similar analysis, made per each Spanish autonomous community, shows that the dynamics is also obeyed individually by regions, as shown in figure 4. We do not find any exception in all the 15 Spanish autonomous communities during these 10 years. In view of these results, we empirically confirm the validity of the dynamical equation (2.1). Additionally, we find that the temperatures 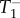 and 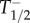 are significantly higher than those at the positive regime. Remarkably, as shown in figure 5, 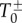 can be, in general, considered the same for positive and negative EBITDA, indicating that the same non-proportional regime is connecting both domains.

### 4.2. Macroequilibrium

**Figure 6.**
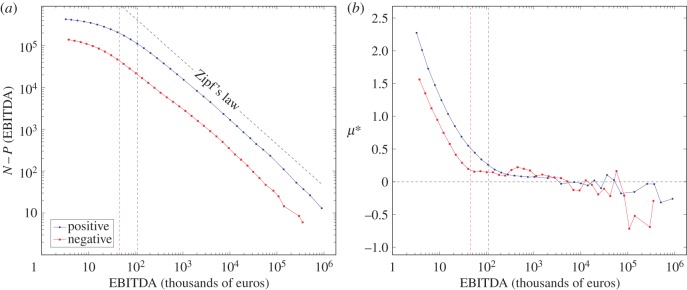
Cumulative distribution (*a*) and chemical potential μ* as measured from the distribution (*b*) for the empirical data of Spain as a whole in 2009. Vertical dashed lines display the transition to proportional growth regimes as estimated from the temperatures (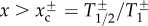, see figure 3 for the measured values), where the equation of state (2.11) holds. Remarkably, the measured μ* converges to a value around 0 just after the transition, shown as Zipf's law for the cumulative distribution. Some numerical fluctuations also appear, as in the case of the simulation with walkers (figure 2).

Once the dynamical equation has been validated, we pass to EBITDA distributions. We plot in figure 6 the complementary cumulative function 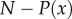 for all Spanish firms in 2009, including those with positive and negative EBITDA. We observe that for large values the power-law equation (2.12) is followed, as predicted by our thermodynamic equilibrium hypothesis, with an exponent very close to that of Zipf's law *λ* = 1. For smaller values, the distribution deviates from the power law. We have checked that this deviation systematically takes place at about the same transition value 
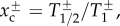
 as predicted by our numerical experiments. This is compelling evidence for the relation between the dynamics and the distribution given by our theoretical framework. We also measure the chemical potential μ* as we did in the numerical experiment with walkers. This chemical potential is, in general, with some deviations, close to *μ* = 0, a value for which the creation/extinction of firms has no cost for the energy potential function 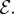 Remarkably, transition threshold 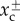 is again the point where the chemical potential blows up, exhibiting agreement between the macroequilibrium distribution and the microdynamical variances.

We find the same picture when studying the firms' distribution per each community. In general, all distributions are very close to the Zipf regime where creation and destruction have no cost to the system. In view of these results, we consider that our theoretical framework properly describes the dynamics and equilibrium of the ensemble of Spanish firms.

## 5. Discussion

Some interesting assertions can be made with regards to our theoretical framework. The most relevant is the thermodynamic interpretation of the exponent λ* of the long tail of size distributions. Thanks to the equation of state equation (2.11), we provide for the first time, to the best of our knowledge, a clear explanation for this exponent, linking this dimensionless number with a dynamical, intuitive mechanism as the cost to the system of creating or extinguishing a firm, measured by the chemical potential μ*. This interpretation can be used to measure the macroscopic effect of particular economical policies, and to measure how healthy is a capitalist-based economy. We find that, in general, the value of μ* in Spanish regions can be considered as zero within the given confidence level—as shown in figure 6—indicating the freedom of creating or extinguishing a firm. We expect that other datasets from other regions around the world, where the economy exhibits large deviations from the exponent value λ* = 1, can be used to quantitatively display this correlation with the creation/destruction of firms. Indeed, using (i) some of the tools used in [62] for analysing the success and failure rates of given firms and (ii) an analysis of the dynamical time correlations as done in [28,75] for population dynamics, the cost to the system for creating/destroying a firm can be estimated and correlated with the Zipf exponent. Remarkably, the derivation of equation (2.11) has been made just under the assumptions of proportional growth and two simplest forms of constraints, implying that any stochastic system under the same dynamical conditions—and not only an ensemble of firms—can potentially be described at the macroscopic level by this thermodynamic approach.

In addition to the macroscopic description, we establish here the form of the microscopic dynamics and its dependence on size by equation (2.1). Contrary to other social systems following proportional growth [12,28,76], there exists, here, the possibility of *negative* values. This requires an additional dynamical mechanism for the evolution of firms, which is successfully included in our current approach as a linear term dominant for small sizes. Firms can be classified according to the dynamical regime, no matter whether they are in the negative (losses) or positive (gain) domain. In a pictorial fashion, we can talk about *heaven* (positive proportional regime, where rich get richer), *hell* (negative proportional regime, where poor get poorer) or *purgatory* (linear regime). The fact that the temperatures in the positive domain are systematically smaller than in the negative one, as illustrated in figure 5, can be described by stating that *hell is warmer than heaven*. Thus, a firm in *hell* loses money in a faster fashion than it would equivalently earn it in *heaven*.

**Figure 7.**
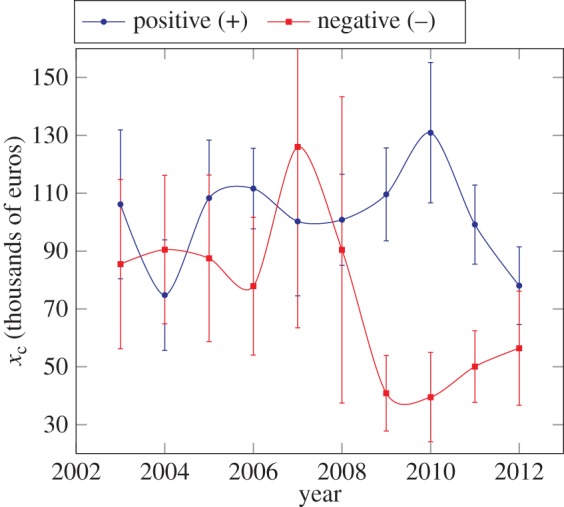
Evolution of the position of the transition to proportional growth 
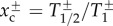
 for positive and negative EBITDA for Spain as a whole. Remarkably, the value is similar in hell and heaven before the crisis, diverges during the burst of the crisis, and slowly converges again to a lower value after the crisis.

We also find useful as a macroeconomic indicator the position of the transition zone between medium and proportional growth regime in the negative domain that gives an estimate of the minimum losses a firm can afford before going bankrupt—or metaphorically, *hell's gate*. Similarly, the same transition but in the positive domain provides an estimation of the success region for firms—that we might wish to call *heaven's door*. Figure 7 shows both transition values from 2003 to 2012 as measured by the respective temperature ratios 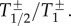 We observe that, before the 2008 global financial crisis, both transitions were approximately equivalent in size, exhibiting a symmetry between positive and negative regimes. Right before this crisis, the negative value reached its maximum, indicating some abnormal economic growth, potentially related with the speculative bubble. The confidence interval for this specific year is higher than the absolute value, indicating that this phenomenon did not happen with the same intensity among all the autonomous communities. Finally, in the succeeding years, the negative value was reduced to a half, augmenting the probability of firms to go bankrupt, whereas the positive transition also decreased, although not as rapidly as in the negative domain. After the burst of the crisis, both transitions tend to converge again to a similar value, but lower than before the crisis. Because the equation of state equation (2.11) and the constrained value of 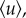 this lower value reflects a general reduction of the wealth in the whole system, because it diminishes, on average, the scale of the successful firms at the proportional regime.

As a final remark, the numerical simulation provided here, based on walkers and the grand canonical ensemble, opens the possibility of developing simulation tools where economical forces can be introduced in the same fashion as one does for physical forces in gases and liquids. Indeed, we open a bridge between the mathematical tools used in statistical mechanics and firms' dynamics. Our analytical and numerical procedures can be used to analyse the empirical data measuring and parametrizing the economic forces in play, and develop a full quantitative theory concerning the dynamics. Alternatively to walkers, the system can also be described in terms of the Fokker–Planck equation, as done by Yakovenko and co-workers [3,4,7] for household income and wealth. Indeed, if all the terms of the dynamical equation (2.1) are correctly introduced into a Fokker–Planck form, we should obtain equivalent solutions for the shape of the density distribution. Work in this direction is currently in progress.

## 6. Conclusion

We advanced in this paper a complete thermodynamic structure that accommodates the FSD of a given region. We attempted an empirical proof of a microscopic dynamical hypothesis, and showed how firms obey the maximum entropy principle at the macroscopic level. We analytically proved the connection between microscopic dynamics and equilibrium FSDs via MaxEnt, and formulated the equation of state that relates the exponent of size distributions with a well-known thermodynamic observable, namely the chemical potential. This leads to a clear and intuitive interpretation of the exponents, showing that they can be used as indicators of the health of an economy. Indeed, the emergence of Zipf's law is associated with the free cost (to the system) of creating and extinguishing firms, as expected in a capitalist-based economy. All these theoretical considerations have been validated by comparison with empirical data concerning Spanish firms, in a window of a decade.

Summarizing, this work contributes to the modelling of economies and to quantitative economics in three ways.

(i) The systematic use and application of a thermodynamic principle, the maximum entropy one, to ensembles of firms, taking into account dynamical symmetries like proportional growth.
(ii) The development of a formal mathematical structure, that of the thermodynamics of firms' growth, that exhibits predictive power. More than a mere model, we are erecting into which other mechanisms and forces can be easily accommodated by following recipes borrowed from physics. Indeed, other forces—those representing special policies or special economic situations—can be included as additional constraints and one can predict their effect in the EBITDA distributions.
(iii) Additionally, we also present in the electronic supplementary material an exhaustive analysis of Spanish firms' data that can be used for empirical testing.

We expect this work to be just a first step towards the formalization of a theory of the evolution of firms that will yield a better understanding of underlying forces and laws of evolution.

## Supplementary Material

Suporting Information: Thermodynamics of firms' growth
