# Supplementary material for: Thermodynamics of firms' growth
Source: J R Soc Interface. 2015 Nov 6;12(112):20150789. doi: 10.1098/rsif.2015.0789 (PMC4685849; doi:10.1098/rsif.2015.0789)
Supplement: Suporting Information: Thermodynamics of firms' growth [file rsif20150789supp1.pdf]

Supporting Information
"Thermodynamics of firms' growth"

Eduardo Zambrano*

*Max-Planck-Institut für Physik komplexer Systeme,
Nönthnizer Str. 38, D-01187, Dresden, Germany

Alberto Hernando†

†SThAR, Social Thermodynamics Applied Research,
CH-1025, Lausanne, Switzerland

Aurelio Fernández-Bariviera‡

†Department of Business, Universitat Rovira i Virgili,
Av. Universitat 1, 43204, Reus, Spain

Ricardo Hernando†

†SThAR, Social Thermodynamics Applied Research,
CH-1025, Lausanne, Switzerland

Angelo Plastino§

§National University of La Plata, Physics Institute (IFLP-CCT-CONICET)
C.C. 737, 1900, La Plata, Argentina

This Supporting Information contains the figures and tables for all the autonomous Spanish communities[1] in the period 2003-2012. The figures are: the rank-plot, the chemical potential, and the variance of EBITDA as function of the EBITDA. The tables show the respective temperatures by year.

1. Spain
2. Andalusia
3. Aragon
4. Asturias
5. Balearic islands
6. Basque country
7. Canary islands
8. Cantabria
9. Castile and Leon
10. Castile-La Mancha
11. Catalonia
12. Extremadura
13. Galicia
14. La Rioja
15. Madrid
16. Murcia
17. Navarre
18. Valencian community

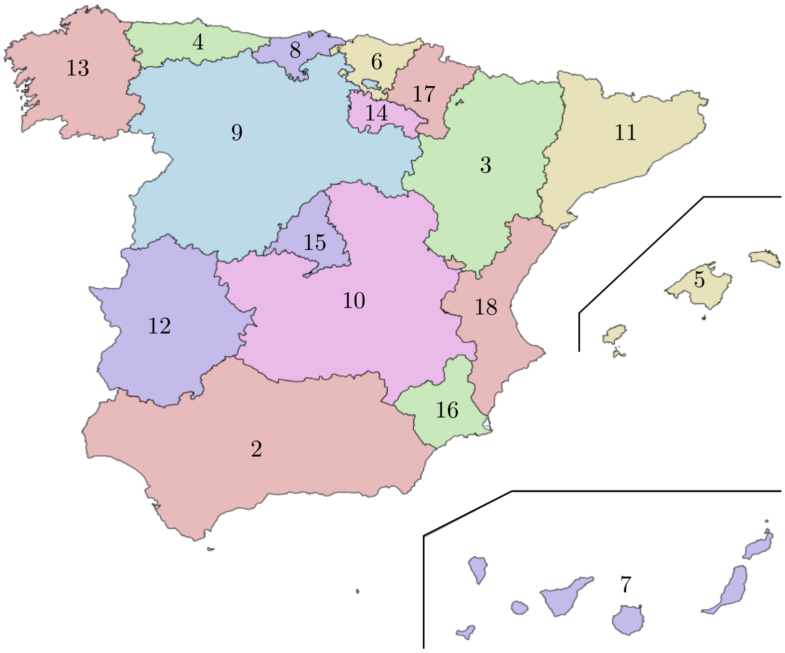

[1]The autonomous cities of Ceuta and Melilla are not included in this work.

∗ Figure based on Creative Commons CC BY-SA 3.0-“*Autonomous communities of Spain*” by Habbit.

Spain

Tables of Temperatures

Positive EBITDA

Year	T_1	$T_{1/2}$	T_0	$T_{1/2}/T_1$	Num. Firms
2003	0.57 ± 0.11	60.52 ± 14.56	528.40 ± 193.19	105.3	415865
2004	0.64 ± 0.09	47.87 ± 12.14	664.95 ± 184.10	74.5	428486
2005	0.62 ± 0.10	67.16 ± 12.35	837.13 ± 159.74	109.1	449524
2006	0.67 ± 0.06	74.79 ± 9.29	965.96 ± 148.94	112.0	470854
2007	0.78 ± 0.15	78.21 ± 19.91	981.09 ± 221.10	100.7	485222
2008	1.14 ± 0.14	114.96 ± 17.80	1298.55 ± 213.37	100.4	432543
2009	1.27 ± 0.19	139.21 ± 20.18	1066.93 ± 189.10	109.7	461783
2010	0.90 ± 0.21	117.83 ± 21.63	902.29 ± 252.06	131.0	464758
2011	1.10 ± 0.14	109.10 ± 14.90	1088.83 ± 176.94	99.5	422307
2012	0.92 ± 0.13	71.81 ± 12.24	604.28 ± 136.09	78.5	230209

Negative EBITDA

Year	T_1	$T_{1/2}$	T_0	$T_{1/2}/T_1$	Num. Firms
2003	2.86 ± 0.59	244.52 ± 141.86	418.37 ± 162.48	85.4	103152
2004	2.78 ± 0.47	251.65 ± 115.62	493.50 ± 152.97	90.7	104869
2005	2.35 ± 0.46	205.63 ± 107.68	1018.63 ± 165.08	87.4	111046
2006	2.93 ± 0.49	228.14 ± 132.45	814.21 ± 198.22	77.8	117356
2007	3.48 ± 1.17	438.48 ± 407.53	359.78 ± 243.03	125.9	112675
2008	5.96 ± 2.08	538.87 ± 948.45	895.34 ± 424.41	90.4	109301
2009	6.06 ± 0.91	245.91 ± 288.57	1470.71 ± 461.58	40.6	171427
2010	5.19 ± 0.90	205.09 ± 216.31	917.49 ± 269.41	39.5	196737
2011	5.14 ± 0.63	257.43 ± 134.08	877.67 ± 169.99	50.1	187046
2012	2.73 ± 0.48	154.07 ± 104.56	1024.32 ± 181.77	56.3	108898

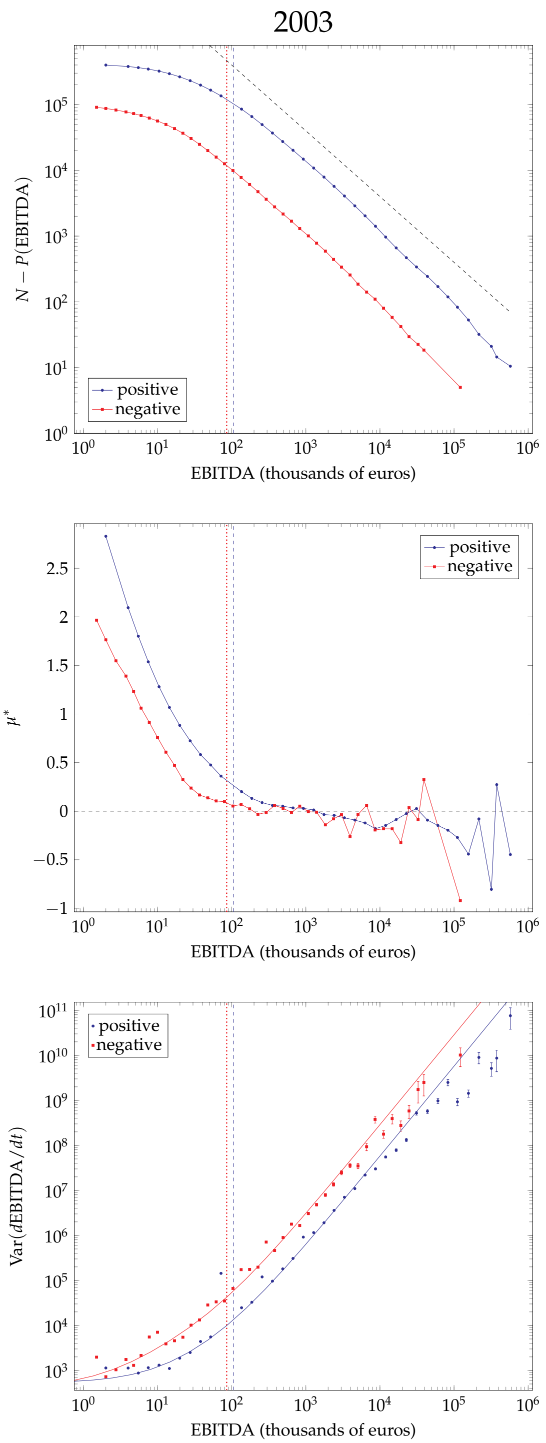

Figure 1: **Spain 2003: Rank plot, chemical potential and variance.**
Positive EBITDA: 415865 firms.
$T_1 = 0.57 \pm 0.11$, $T_{1/2} = 60.52 \pm 14.56$, and $T_0 = 528.40 \pm 193.19$
Negative EBITDA: 103152 firms.
$T_1 = 2.86 \pm 0.59$, $T_{1/2} = 244.52 \pm 33.23$, and $T_0 = 418.37 \pm 162.48$.
Total active firms 519017, total created firms 99120, and total destroyed firms 46258

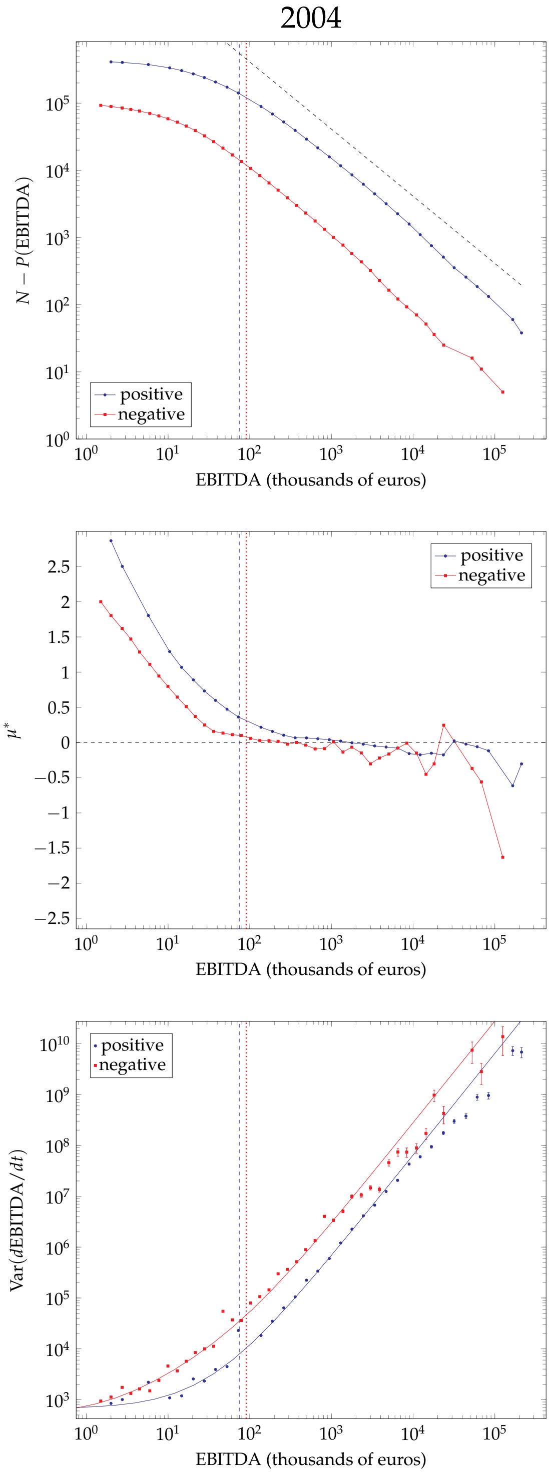

Figure 2: **Spain 2004: Rank plot, chemical potential and variance**.
Positive EBITDA: 428486 firms.
$T_1 = 0.64 \pm 0.09$, $T_{1/2}$= 47.87 ± 12.14, and T_0 =664.95 ± 184.10
Negative EBITDA: 104869 firms.
$T_1 = 2.78 \pm 0.47$, $T_{1/2}$= 251.65 ± 28.76, and T_0 =493.50 ± 152.97.
Total active firms 533355, total created firms 95864, and total destroyed firms 84521

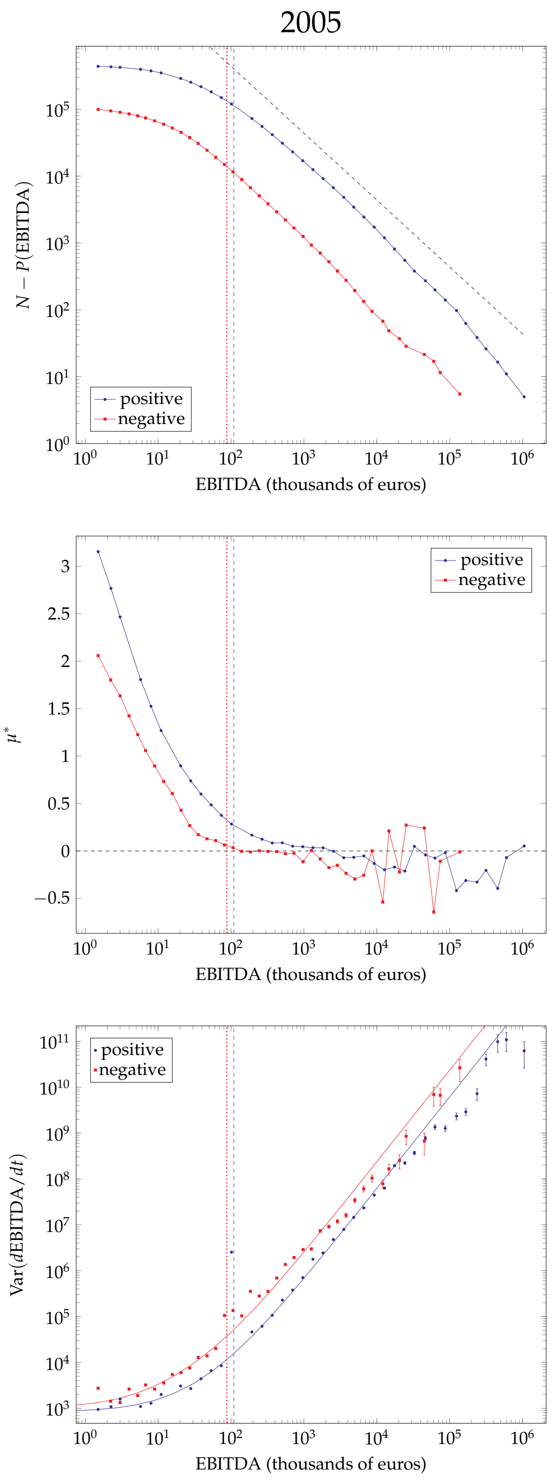

Figure 3: **Spain 2005: Rank plot, chemical potential and variance**.
Positive EBITDA: 449524 firms.
$T_1 = 0.62 \pm 0.10$, $T_{1/2} = 67.16 \pm 12.35$, and $T_0 = 837.13 \pm 159.74$
Negative EBITDA: 111046 firms.
$T_1 = 2.35 \pm 0.46$, $T_{1/2} = 205.63 \pm 27.35$, and $T_0 = 1018.63 \pm 165.08$.
Total active firms 560570, total created firms 98002, and total destroyed firms 69531

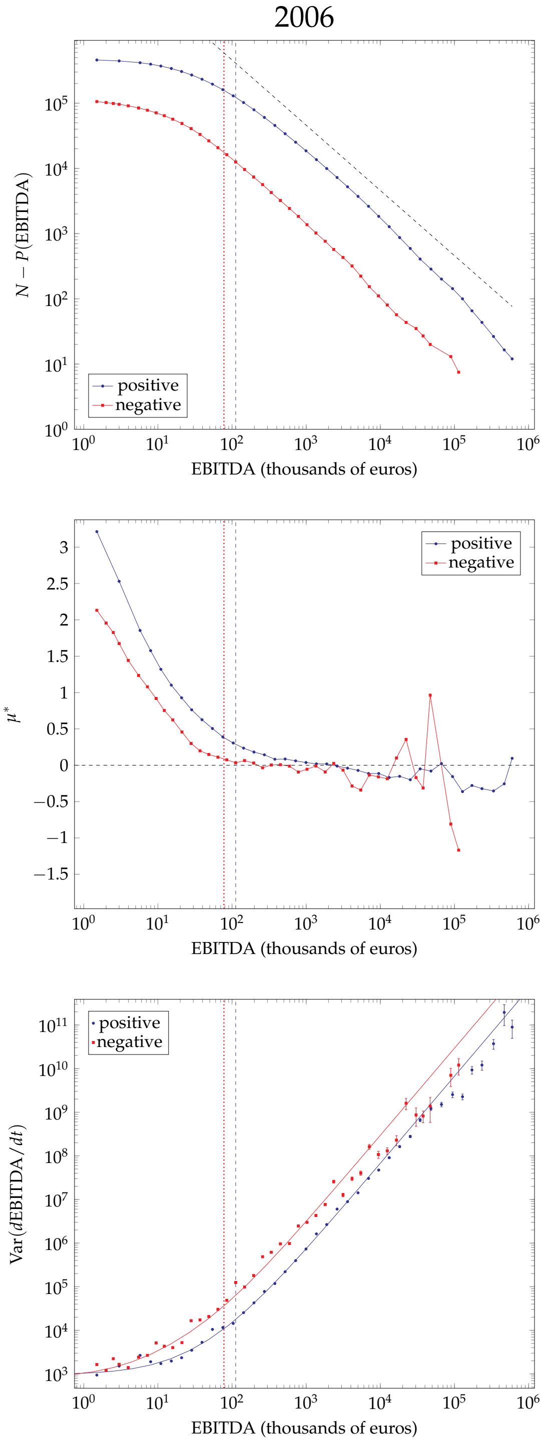

Figure 4: **Spain 2006: Rank plot, chemical potential and variance.**
Positive EBITDA: 470854 firms.
T_1 = 0.67 ± 0.06, $T_{1/2}$= 74.79 ± 9.29, and T_0 =965.96 ± 148.94
Negative EBITDA: 117356 firms.
T_1 = 2.93 ± 0.49, $T_{1/2}$= 228.14 ± 31.66, and T_0 =814.21 ± 198.22.
Total active firms 588210, total created firms 116077, and total destroyed firms 70020

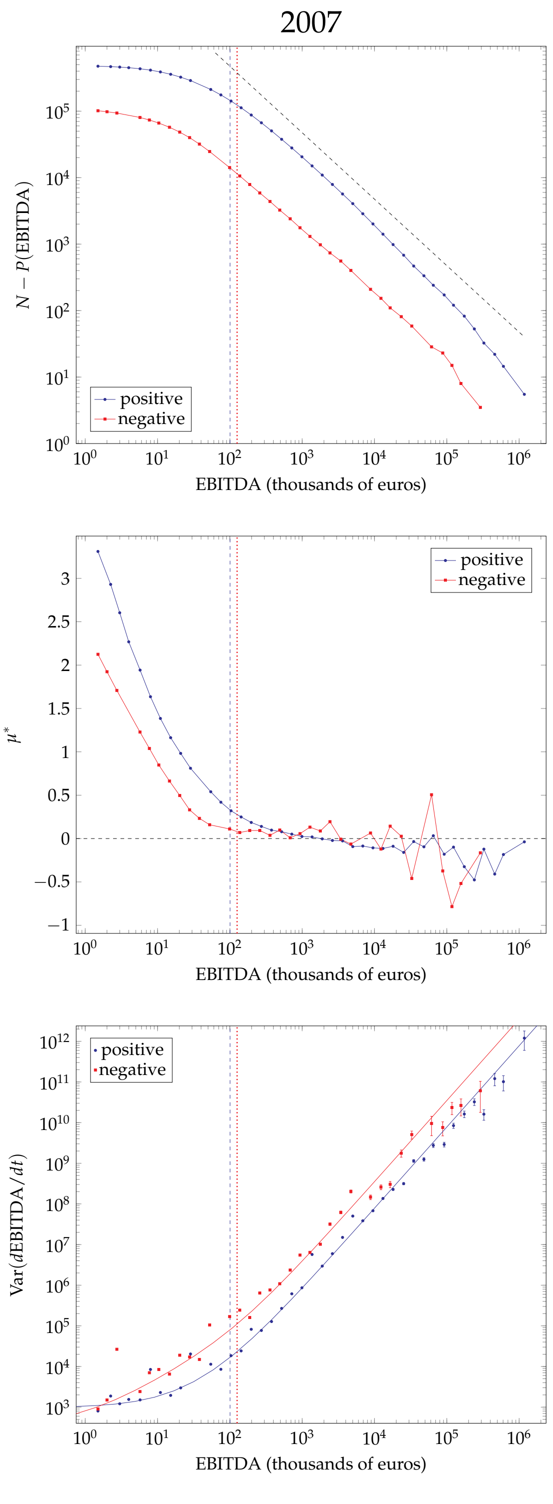

Figure 5: **Spain 2007: Rank plot, chemical potential and variance.**
Positive EBITDA: 485222 firms.
T_1 = 0.78 ± 0.15, $T_{1/2}$= 78.21 ± 19.91, and T_0 =981.09 ± 221.10
Negative EBITDA: 112675 firms.
T_1 = 3.48 ± 1.17, $T_{1/2}$= 438.48 ± 70.09, and T_0 =359.78 ± 243.03.
Total active firms 597897, total created firms 44768, and total destroyed firms 106512

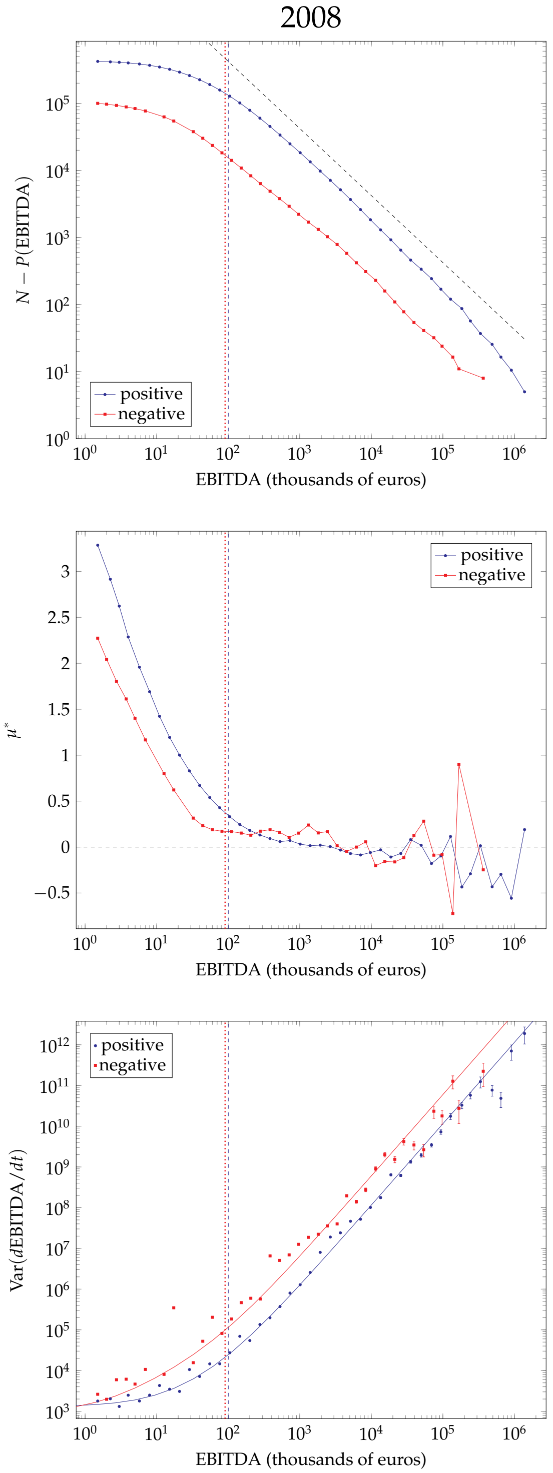

Figure 6: **Spain 2008: Rank plot, chemical potential and variance**.
Positive EBITDA: 432543 firms.
T_1 = 1.14 ± 0.14, $T_{1/2}$= 114.96 ± 17.80, and T_0 =1298.55 ± 213.37
Negative EBITDA: 109301 firms.
T_1 = 5.96 ± 2.08, $T_{1/2}$= 538.87 ± 127.37, and T_0 =895.34 ± 424.41.
Total active firms 541844, total created firms 183923, and total destroyed firms 103452

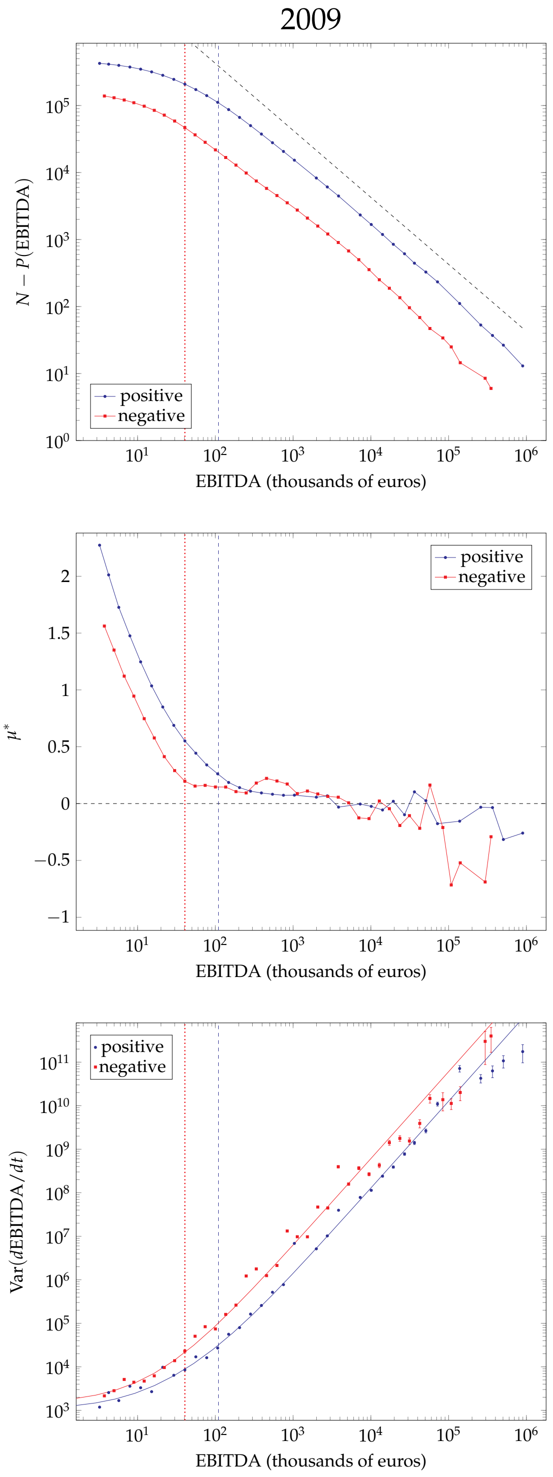

Figure 7: **Spain 2009: Rank plot, chemical potential and variance**.
Positive EBITDA: 461783 firms.
T_1 = 1.27 ± 0.19, $T_{1/2}$= 139.21 ± 20.18, and T_0 =1066.93 ± 189.10
Negative EBITDA: 171427 firms.
T_1 = 6.06 ± 0.91, $T_{1/2}$= 245.91 ± 54.91, and T_0 =1470.71 ± 461.58.
Total active firms 633210, total created firms 91586, and total destroyed firms 65814

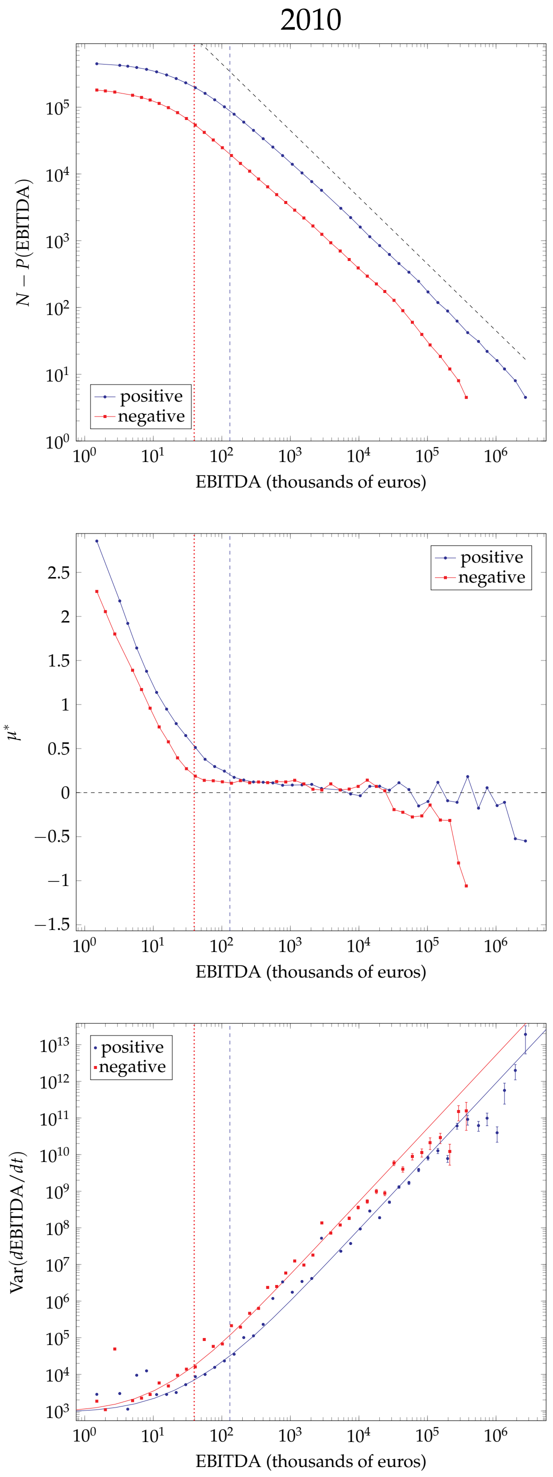

Figure 8: **Spain 2010: Rank plot, chemical potential and variance**.
Positive EBITDA: 464758 firms.
$T_1 = 0.90 \pm 0.21$, $T_{1/2} = 117.83 \pm 21.63$, and $T_0 = 902.29 \pm 252.06$
Negative EBITDA: 196737 firms.
$T_1 = 5.19 \pm 0.90$, $T_{1/2} = 205.09 \pm 44.79$, and $T_0 = 917.49 \pm 269.41$.
Total active firms 661495, total created firms 70627, and total destroyed firms 85592

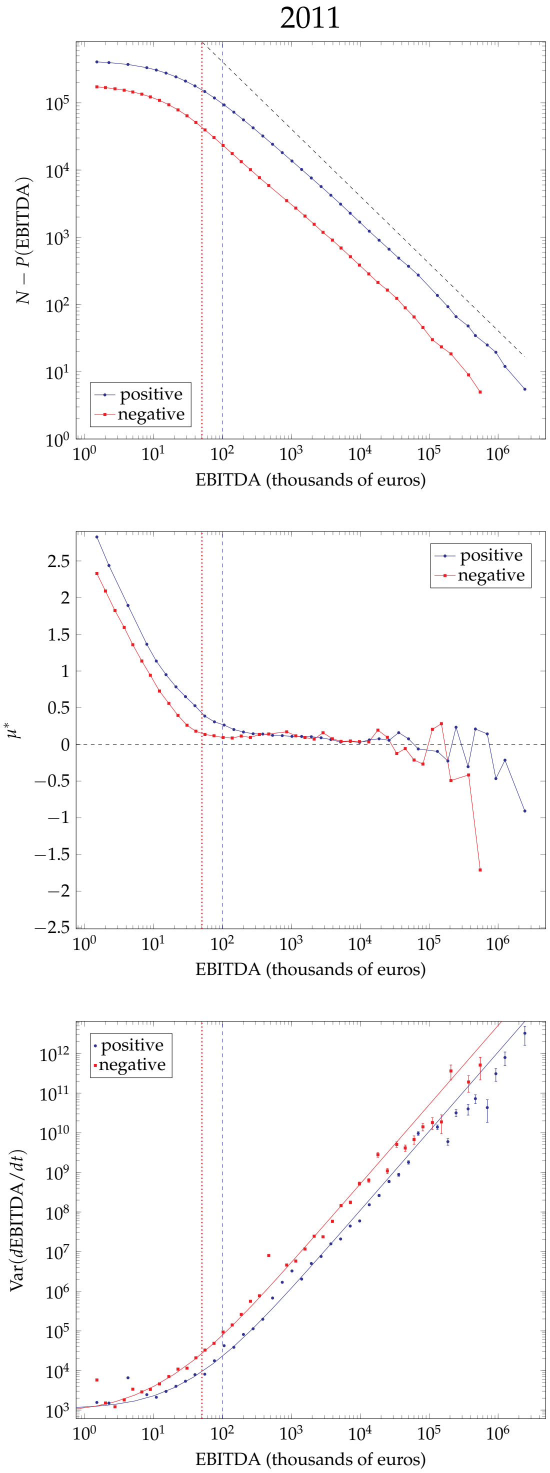

Figure 9: **Spain 2011: Rank plot, chemical potential and variance**.
Positive EBITDA: 422307 firms.
T_1 = 1.10 ± 0.14, $T_{1/2}$= 109.10 ± 14.90, and T_0 =1088.83 ± 176.94
Negative EBITDA: 187046 firms.
T_1 = 5.14 ± 0.63, $T_{1/2}$= 257.43 ± 31.94, and T_0 =877.67 ± 169.99.
Total active firms 609353, total created firms 30702, and total destroyed firms 101886

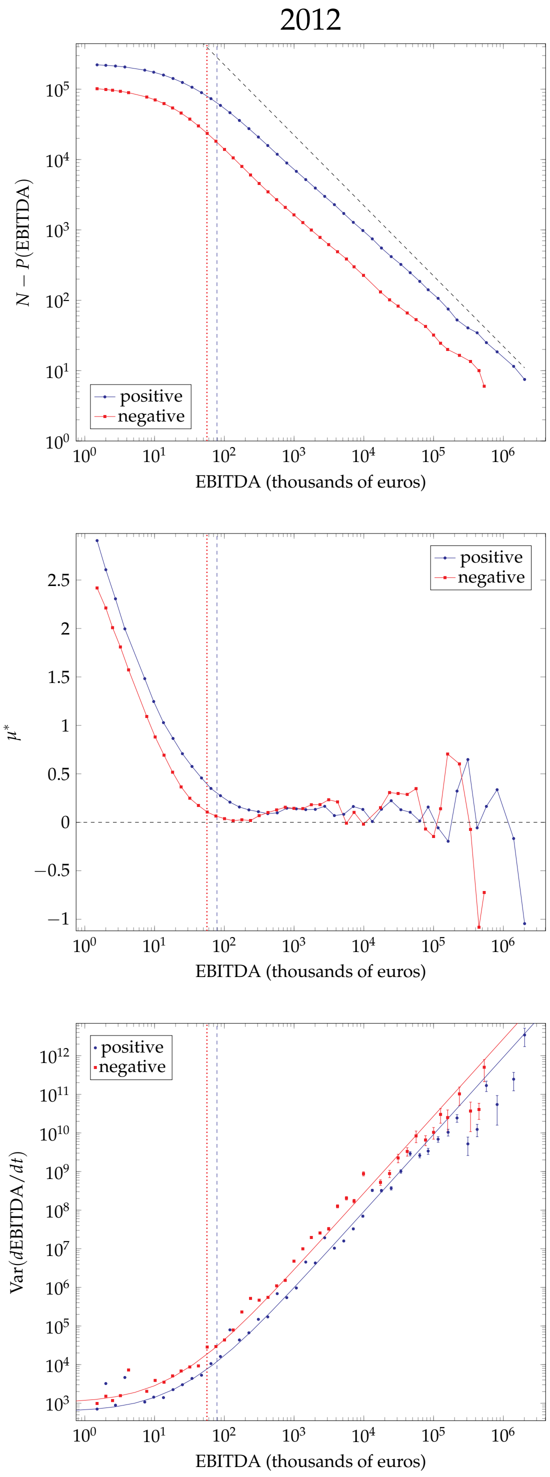

Figure 10: **Spain 2012: Rank plot, chemical potential and variance**.
Positive EBITDA: 230209 firms.
$T_1 = 0.92 \pm 0.13$, $T_{1/2}$= 71.81 ± 12.24, and T_0 =604.28 ± 136.09
Negative EBITDA: 108898 firms.
$T_1 = 2.73 \pm 0.48$, $T_{1/2}$= 154.07 ± 26.79, and T_0 =1024.32 ± 181.77.
Total active firms 339107, total created firms 6401, and total destroyed firms 328395

Andalusia

Tables of Temperatures

Positive EBITDA

Year	T_1	$T_{1/2}$	T_0	$T_{1/2}/T_1$	Num. Firms
2003	0.75 ± 0.11	47.92 ± 9.76	365.87 ± 89.53	63.5	48702
2004	0.71 ± 0.14	52.17 ± 10.51	345.06 ± 58.81	73.2	50132
2005	0.81 ± 0.10	68.89 ± 22.15	557.57 ± 171.99	85.1	53351
2006	0.69 ± 0.19	89.15 ± 21.59	484.65 ± 117.89	129.1	57098
2007	0.86 ± 0.23	74.86 ± 21.43	597.05 ± 135.27	86.9	56310
2008	0.98 ± 0.15	95.52 ± 17.24	956.99 ± 159.79	97.4	49271
2009	0.94 ± 0.08	89.20 ± 20.45	785.92 ± 169.37	95.1	56793
2010	0.74 ± 0.14	65.50 ± 14.36	671.42 ± 147.02	88.3	56713
2011	0.59 ± 0.09	77.07 ± 31.29	826.07 ± 327.31	129.9	52600
2012	0.64 ± 0.10	84.31 ± 28.32	465.48 ± 207.52	132.4	31522

Negative EBITDA

Year	T_1	$T_{1/2}$	T_0	$T_{1/2}/T_1$	Num. Firms
2003	1.81 ± 0.48	179.90 ± 119.44	388.65 ± 181.23	99.6	12308
2004	2.12 ± 0.46	228.47 ± 141.86	213.84 ± 112.89	108.0	12268
2005	2.78 ± 0.66	141.41 ± 190.76	612.31 ± 352.75	50.8	13240
2006	2.76 ± 0.74	226.72 ± 288.92	248.35 ± 157.94	82.1	14507
2007	2.85 ± 0.55	347.91 ± 299.76	583.45 ± 213.26	121.9	12919
2008	3.93 ± 0.79	247.27 ± 368.34	1707.81 ± 433.07	62.9	12536
2009	4.36 ± 0.59	175.13 ± 401.75	853.21 ± 383.58	40.2	21368
2010	3.27 ± 0.43	78.95 ± 108.85	1141.73 ± 283.37	24.2	23996
2011	2.65 ± 0.33	199.46 ± 250.77	357.58 ± 233.57	75.4	24364
2012	2.28 ± 0.68	196.53 ± 345.63	991.72 ± 317.36	86.4	15335

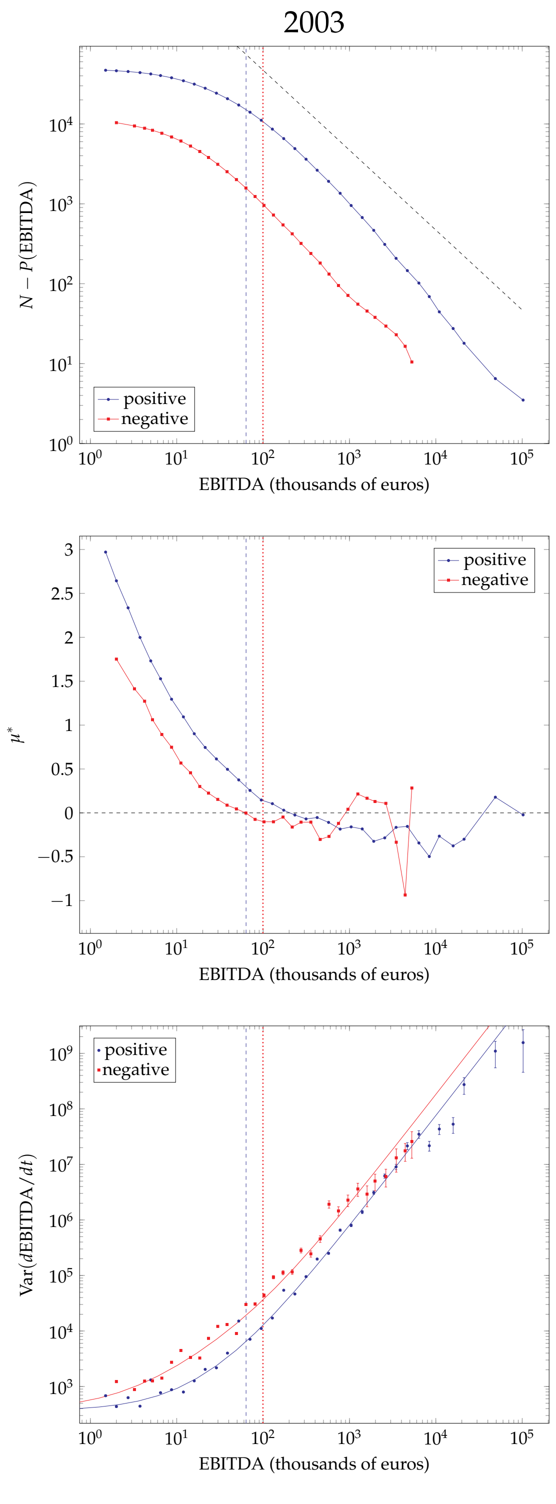

Figure 11: **Andalusia 2003: Rank plot, chemical potential and variance.**
Positive EBITDA: 48702 firms.
T_1 = 0.75 ± 0.11, $T_{1/2}$= 47.92 ± 9.76, and T_0 =365.87 ± 89.53
Negative EBITDA: 12308 firms.
T_1 = 1.81 ± 0.48, $T_{1/2}$= 179.90 ± 29.43, and T_0 =388.65 ± 181.23.
Total active firms 61010, total created firms 13946, and total destroyed firms 7775

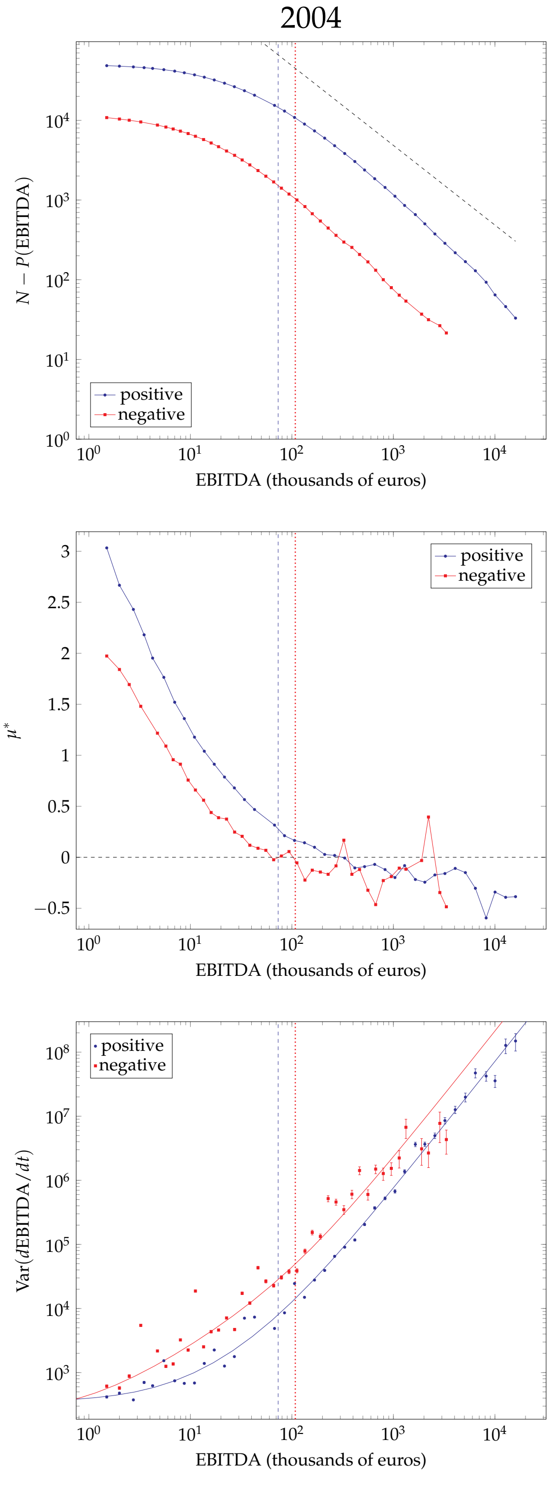

Figure 12: **Andalusia 2004: Rank plot, chemical potential and variance.**
Positive EBITDA: 50132 firms.
$T_1 = 0.71 \pm 0.14$, $T_{1/2} = 52.17 \pm 10.51$, and $T_0 = 345.06 \pm 58.81$
Negative EBITDA: 12268 firms.
$T_1 = 2.12 \pm 0.46$, $T_{1/2} = 228.47 \pm 33.24$, and $T_0 = 213.84 \pm 112.89$.
Total active firms 62400, total created firms 12869, and total destroyed firms 12552

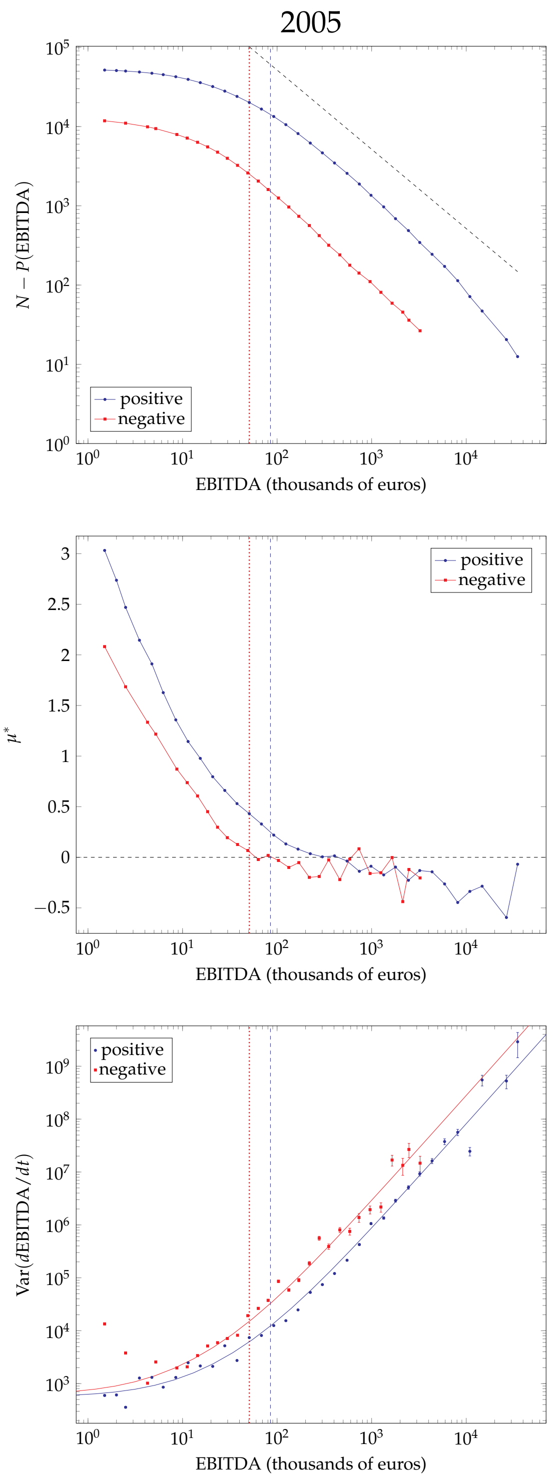

Figure 13: **Andalusia 2005: Rank plot, chemical potential and variance.**
Positive EBITDA: 53351 firms.
T_1 = 0.81 ± 0.10, $T_{1/2}$= 68.89 ± 22.15, and T_0 =557.57 ± 171.99
Negative EBITDA: 13240 firms.
T_1 = 2.78 ± 0.66, $T_{1/2}$= 141.41 ± 40.98, and T_0 =612.31 ± 352.75.
Total active firms 66591, total created firms 15214, and total destroyed firms 8755

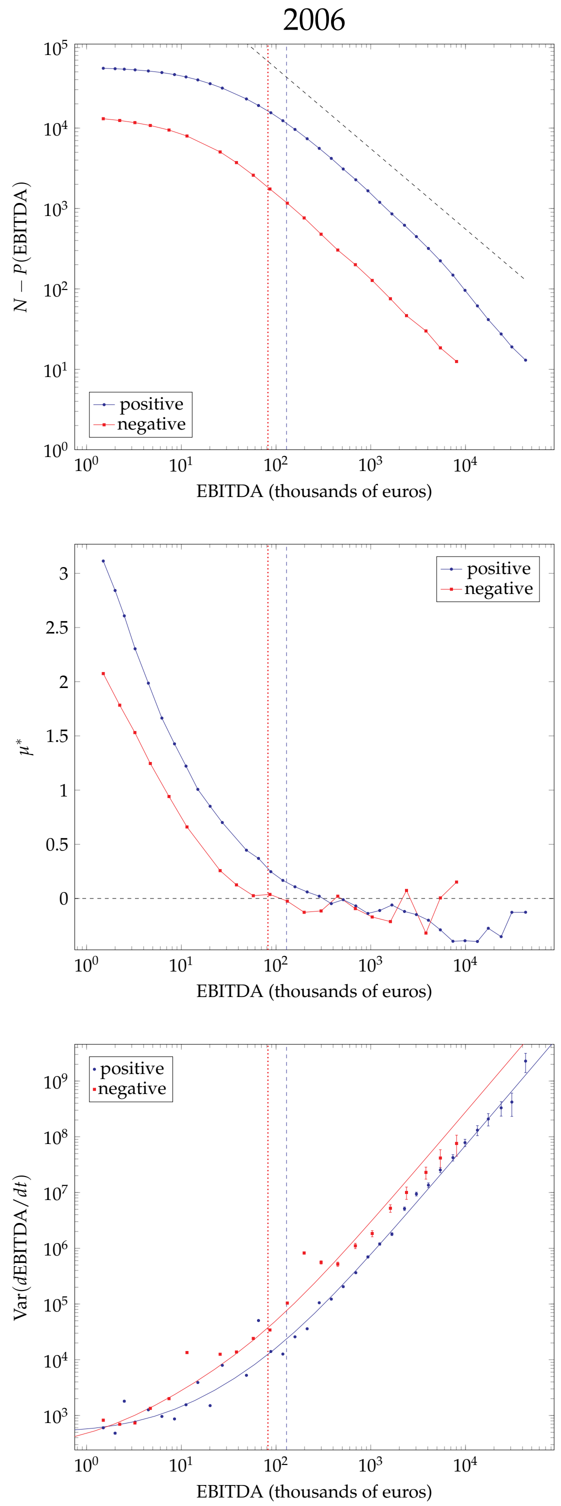

Figure 14: **Andalusia 2006: Rank plot, chemical potential and variance**.
Positive EBITDA: 57098 firms.
T_1 = 0.69 ± 0.19, $T_{1/2}$= 89.15 ± 21.59, and T_0 =484.65 ± 117.89
Negative EBITDA: 14507 firms.
T_1 = 2.76 ± 0.74, $T_{1/2}$= 226.72 ± 54.96, and T_0 =248.35 ± 157.94.
Total active firms 71605, total created firms 13893, and total destroyed firms 10198

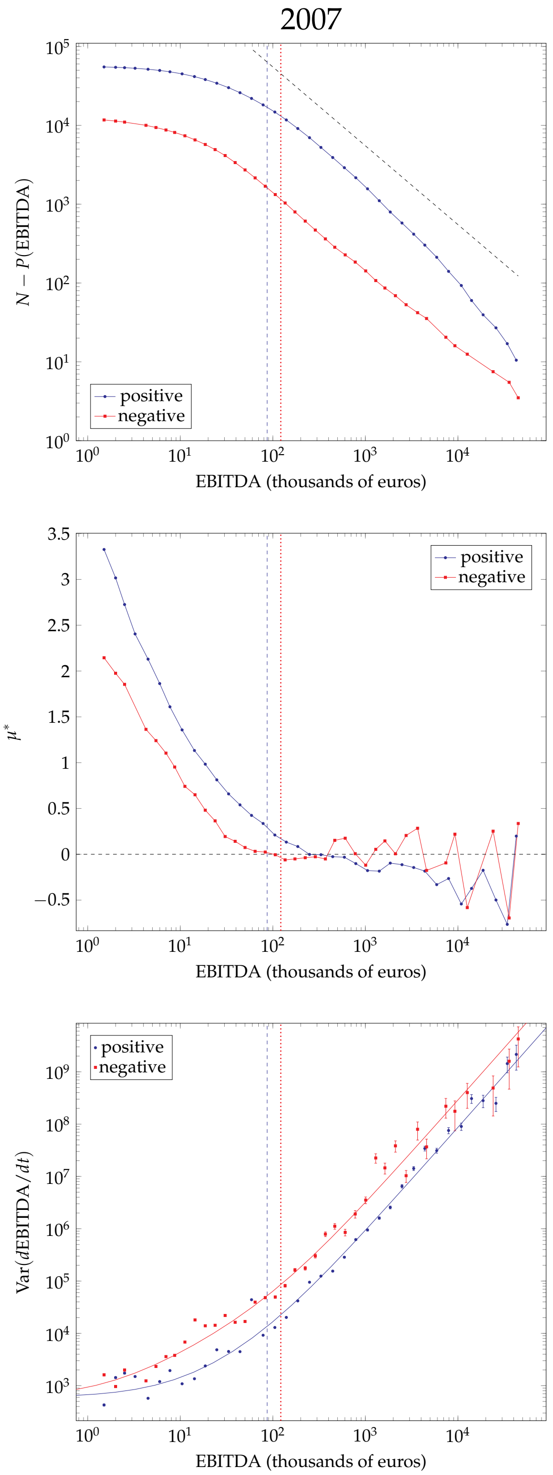

Figure 15: **Andalusia 2007: Rank plot, chemical potential and variance.**
Positive EBITDA: 56310 firms.
T_1 = 0.86 ± 0.23, $T_{1/2}$= 74.86 ± 21.43, and T_0 =597.05 ± 135.27
Negative EBITDA: 12919 firms.
T_1 = 2.85 ± 0.55, $T_{1/2}$= 347.91 ± 56.41, and T_0 =583.45 ± 213.26.
Total active firms 69229, total created firms 5861, and total destroyed firms 16469

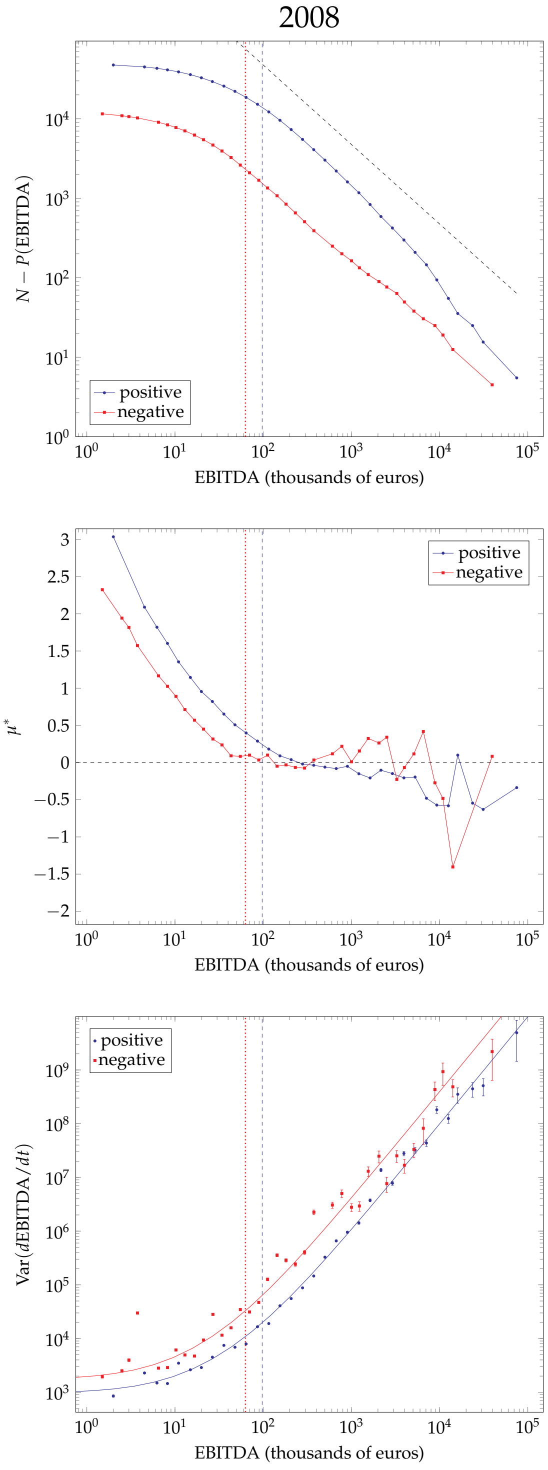

Figure 16: **Andalusia 2008: Rank plot, chemical potential and variance**.
Positive EBITDA: 49271 firms.
$T_1 = 0.98 \pm 0.15$, $T_{1/2}$= 95.52 ± 17.24, and T_0 =956.99 ± 159.79
Negative EBITDA: 12536 firms.
$T_1 = 3.93 \pm 0.79$, $T_{1/2}$= 247.27 ± 65.25, and T_0 =1707.81 ± 433.07.
Total active firms 61807, total created firms 25821, and total destroyed firms 13549

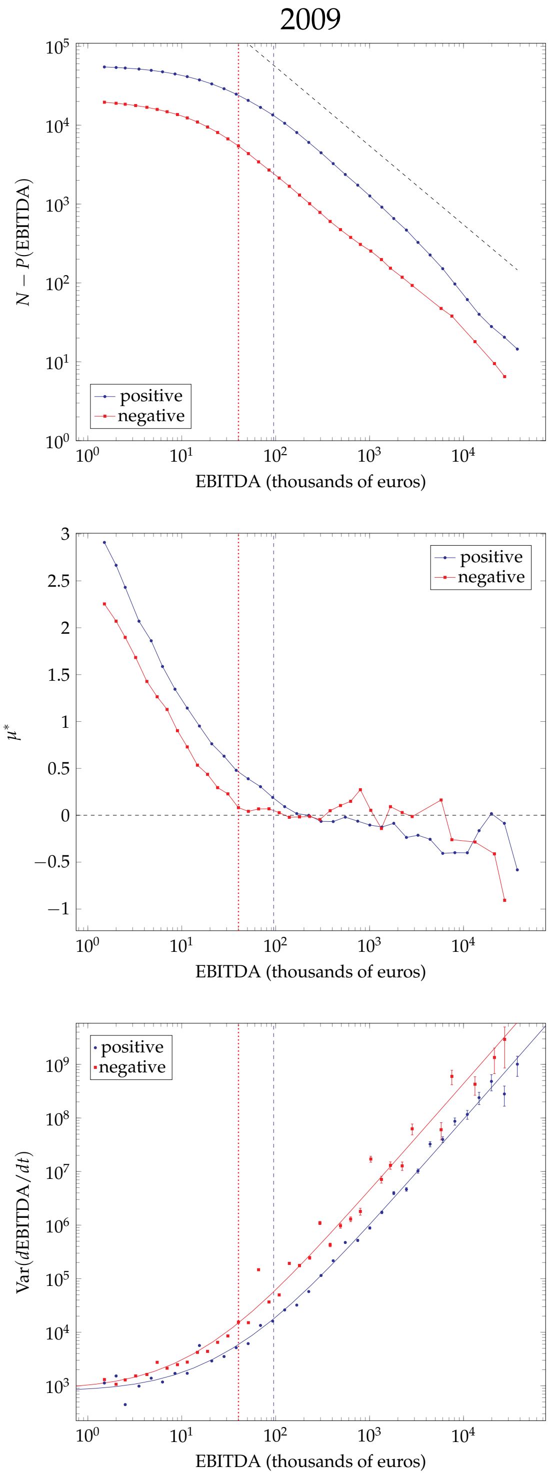

Figure 17: **Andalusia 2009: Rank plot, chemical potential and variance.**
Positive EBITDA: 56793 firms.
$T_1 = 0.94 \pm 0.08$, $T_{1/2}= 89.20 \pm 20.45$, and $T_0 = 785.92 \pm 169.37$
Negative EBITDA: 21368 firms.
$T_1 = 4.36 \pm 0.59$, $T_{1/2}= 175.13 \pm 69.39$, and $T_0 = 853.21 \pm 383.58$.
Total active firms 78161, total created firms 14171, and total destroyed firms 8834

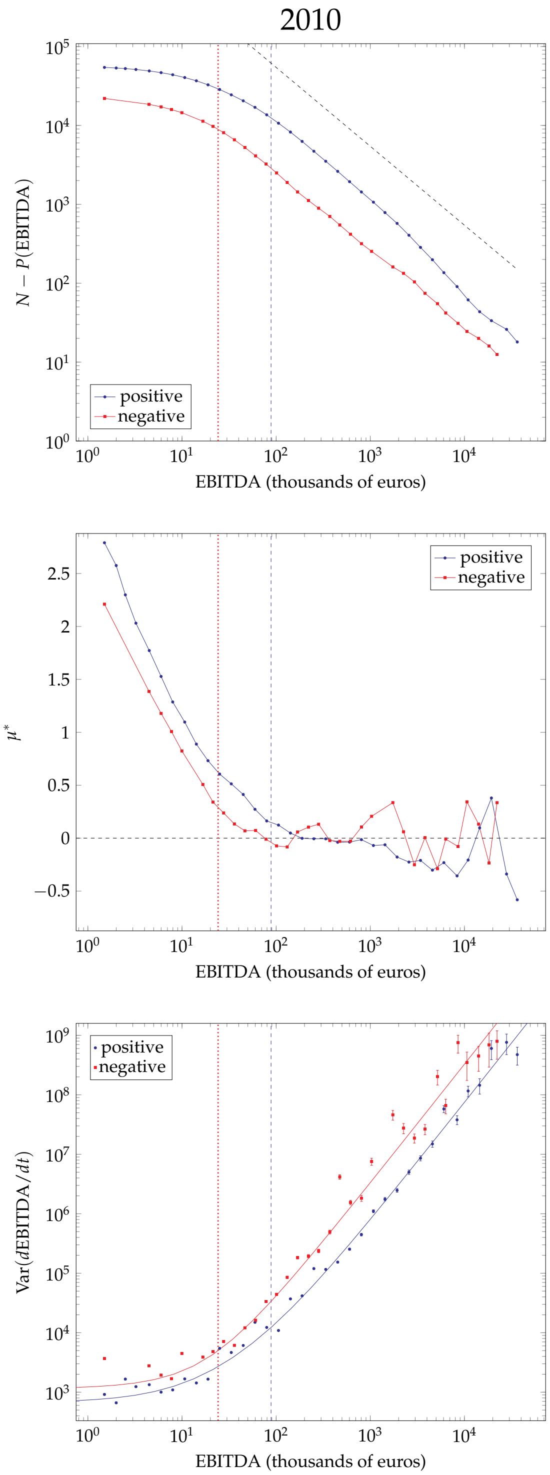

Figure 18: **Andalusia 2010: Rank plot, chemical potential and variance.**
Positive EBITDA: 56713 firms.
$T_1 = 0.74 \pm 0.14$, $T_{1/2} = 65.50 \pm 14.36$, and $T_0 = 671.42 \pm 147.02$
Negative EBITDA: 23996 firms.
$T_1 = 3.27 \pm 0.43$, $T_{1/2} = 78.95 \pm 27.56$, and $T_0 = 1141.73 \pm 283.37$.
Total active firms 80709, total created firms 10129, and total destroyed firms 11545

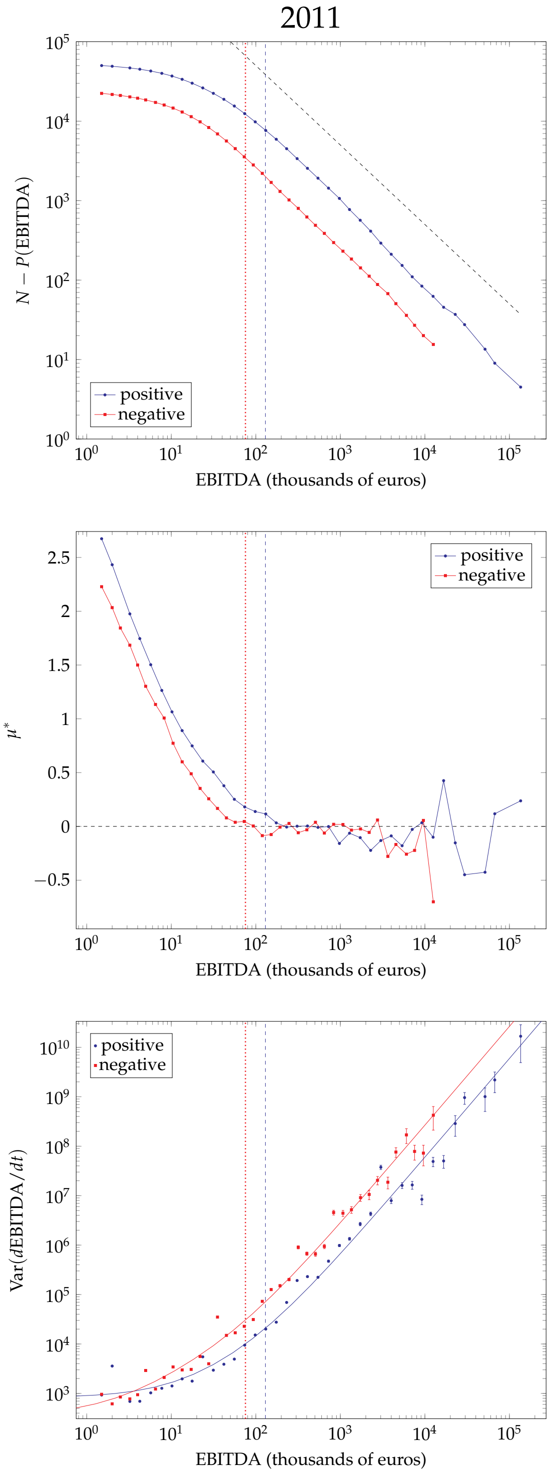

Figure 19: **Andalusia 2011: Rank plot, chemical potential and variance.**
Positive EBITDA: 52600 firms.
T_1 = 0.59 ± 0.09, $T_{1/2}$= 77.07 ± 31.29, and T_0 =826.07 ± 327.31
Negative EBITDA: 24364 firms.
T_1 = 2.65 ± 0.33, $T_{1/2}$= 199.46 ± 49.72, and T_0 =357.58 ± 233.57.
Total active firms 76964, total created firms 4730, and total destroyed firms 13879

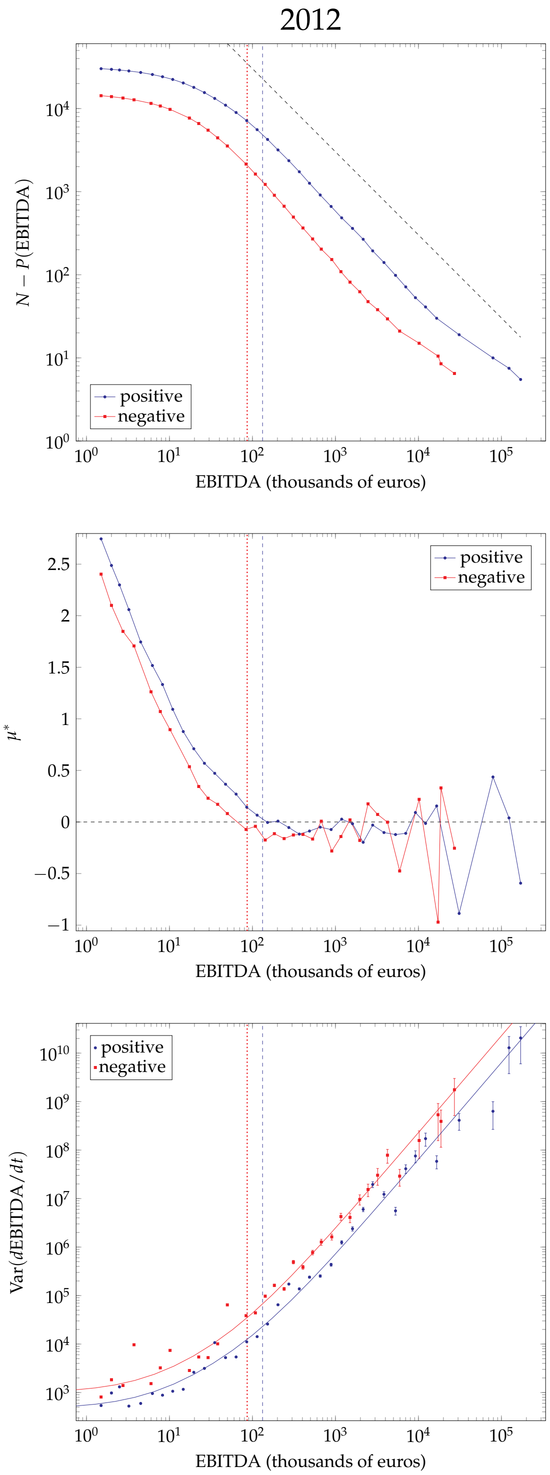

Figure 20: **Andalusia 2012: Rank plot, chemical potential and variance.**
Positive EBITDA: 31522 firms.
$T_1 = 0.64 \pm 0.10$, $T_{1/2}$= 84.31 ± 28.32, and T_0 =465.48 ± 207.52
Negative EBITDA: 15335 firms.
$T_1 = 2.28 \pm 0.68$, $T_{1/2}$= 196.53 ± 62.38, and T_0 =991.72 ± 317.36.
Total active firms 46857, total created firms 991, and total destroyed firms 35540

Aragon

Tables of Temperatures

Positive EBITDA

Year	T_1	$T_{1/2}$	T_0	$T_{1/2}/T_1$	Num. Firms
2003	0.40 ± 0.08	32.77 ± 6.52	213.25 ± 46.31	82.9	14783
2004	0.41 ± 0.06	50.36 ± 17.86	259.12 ± 132.15	122.9	16069
2005	0.49 ± 0.07	82.08 ± 19.94	185.32 ± 122.22	167.6	17717
2006	0.56 ± 0.12	54.23 ± 15.74	273.80 ± 192.97	96.7	18581
2007	0.66 ± 0.10	48.46 ± 11.34	530.42 ± 68.84	73.3	17733
2008	0.82 ± 0.17	141.01 ± 55.06	144.50 ± 363.24	171.4	16136
2009	0.76 ± 0.23	104.25 ± 30.08	670.95 ± 201.43	136.9	16849
2010	0.68 ± 0.23	91.60 ± 29.66	336.24 ± 119.05	133.8	16338
2011	0.49 ± 0.08	75.87 ± 24.28	498.30 ± 210.02	156.2	15382
2012	0.41 ± 0.07	87.57 ± 20.77	256.19 ± 133.82	211.4	10568

Negative EBITDA

Year	T_1	$T_{1/2}$	T_0	$T_{1/2}/T_1$	Num. Firms
2003	2.08 ± 1.09	101.33 ± 310.20	491.79 ± 360.28	48.8	3103
2004	2.48 ± 0.61	130.45 ± 261.26	235.41 ± 212.59	52.6	3524
2005	2.16 ± 0.88	159.52 ± 189.13	45.33 ± 107.25	73.9	4255
2006	3.39 ± 1.46	115.29 ± 357.99	389.81 ± 249.84	34.0	4364
2007	3.56 ± 0.86	137.65 ± 246.80	238.89 ± 144.51	38.6	3434
2008	4.39 ± 1.02	240.29 ± 1502.00	2709.27 ± 1646.11	54.7	3457
2009	5.32 ± 1.90	279.35 ± 922.38	1272.96 ± 560.03	52.5	5562
2010	2.77 ± 0.98	218.34 ± 407.64	309.62 ± 306.88	78.9	6422
2011	4.77 ± 1.29	152.64 ± 1112.61	1046.97 ± 901.70	32.0	6670
2012	3.52 ± 0.59	84.13 ± 393.09	726.11 ± 413.79	23.9	5052

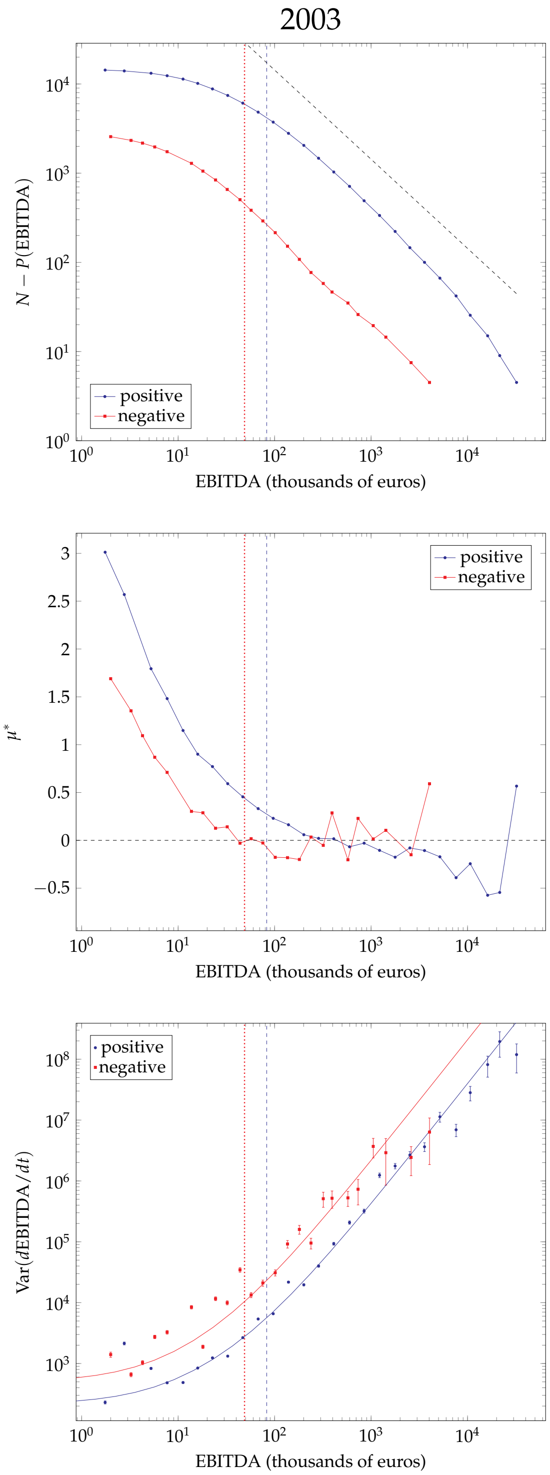

Figure 21: **Aragon 2003: Rank plot, chemical potential and variance.**
Positive EBITDA: 14783 firms.
$T_1 = 0.40 \pm 0.08$, $T_{1/2} = 32.77 \pm 6.52$, and $T_0 = 213.25 \pm 46.31$
Negative EBITDA: 3103 firms.
$T_1 = 2.08 \pm 1.09$, $T_{1/2} = 101.33 \pm 57.79$, and $T_0 = 491.79 \pm 360.28$.
Total active firms 17886, total created firms 3083, and total destroyed firms 1382

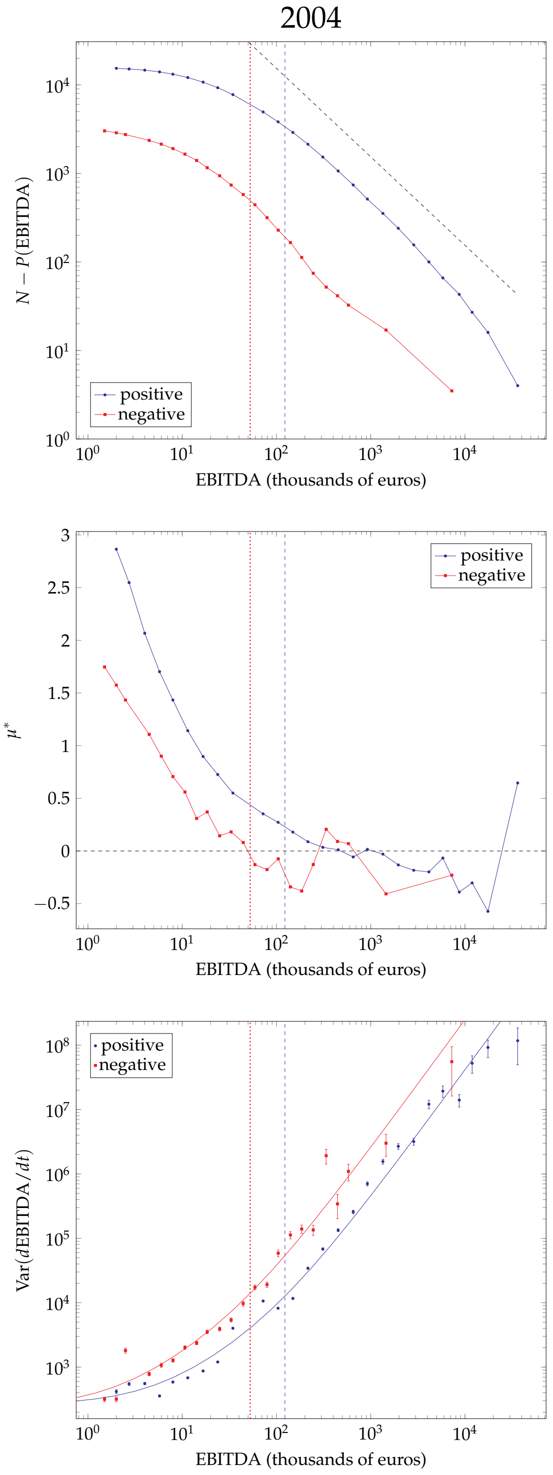

Figure 22: **Aragon 2004: Rank plot, chemical potential and variance.**
Positive EBITDA: 16069 firms.
$T_1 = 0.41 \pm 0.06$, $T_{1/2}= 50.36 \pm 17.86$, and $T_0 =259.12 \pm 132.15$
Negative EBITDA: 3524 firms.
$T_1 = 2.48 \pm 0.61$, $T_{1/2}= 130.45 \pm 51.18$, and $T_0 =235.41 \pm 212.59$.
Total active firms 19593, total created firms 3580, and total destroyed firms 1354

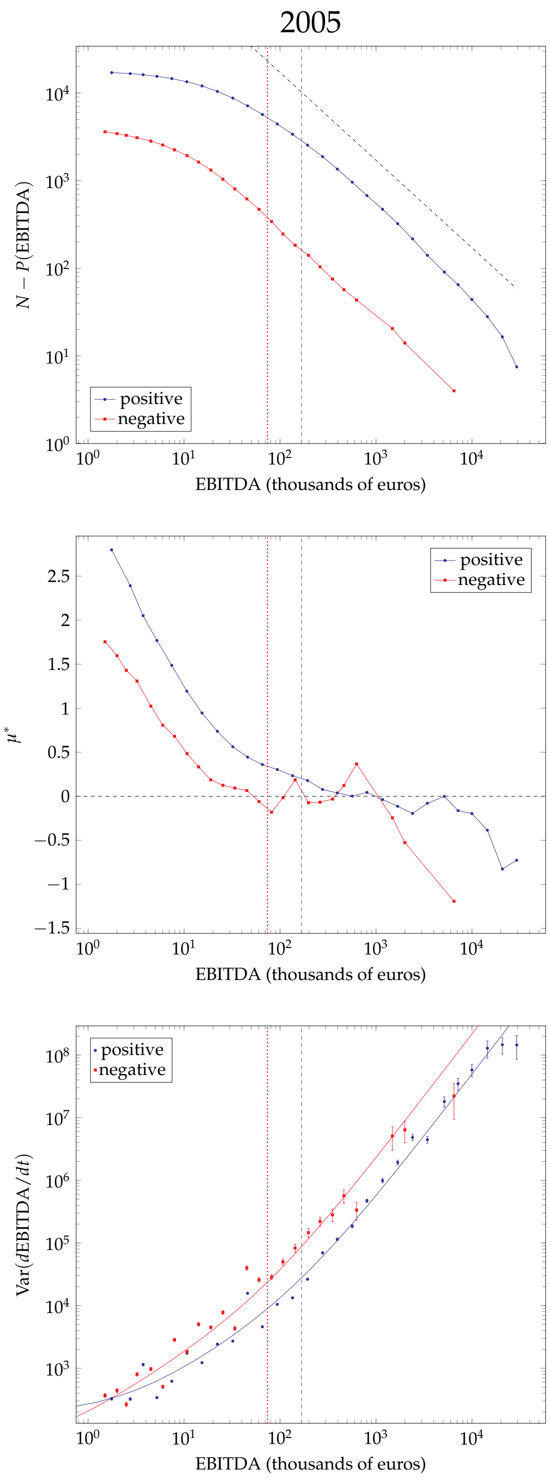

Figure 23: **Aragon 2005: Rank plot, chemical potential and variance.**
Positive EBITDA: 17717 firms.
$T_1 = 0.49 \pm 0.07$, $T_{1/2}$= 82.08 ± 19.94, and T_0 =185.32 ± 122.22
Negative EBITDA: 4255 firms.
$T_1 = 2.16 \pm 0.88$, $T_{1/2}$= 159.52 ± 40.73, and T_0 =45.33 ± 107.25.
Total active firms 21972, total created firms 2610, and total destroyed firms 1038

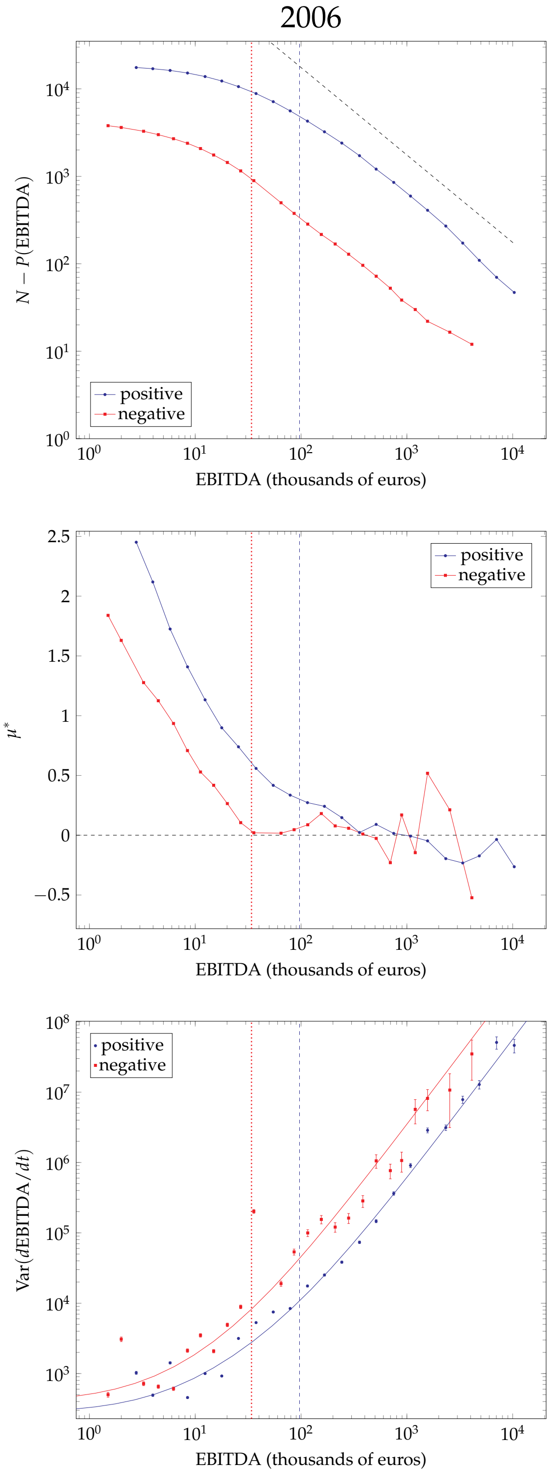

Figure 24: **Aragon 2006: Rank plot, chemical potential and variance**.
Positive EBITDA: 18581 firms.
$T_1 = 0.56 \pm 0.12$, $T_{1/2}$= 54.23 ± 15.74, and T_0 =273.80 ± 192.97
Negative EBITDA: 4364 firms.
$T_1 = 3.39 \pm 1.46$, $T_{1/2}$= 115.29 ± 63.95, and T_0 =389.81 ± 249.84.
Total active firms 22945, total created firms 2509, and total destroyed firms 1614

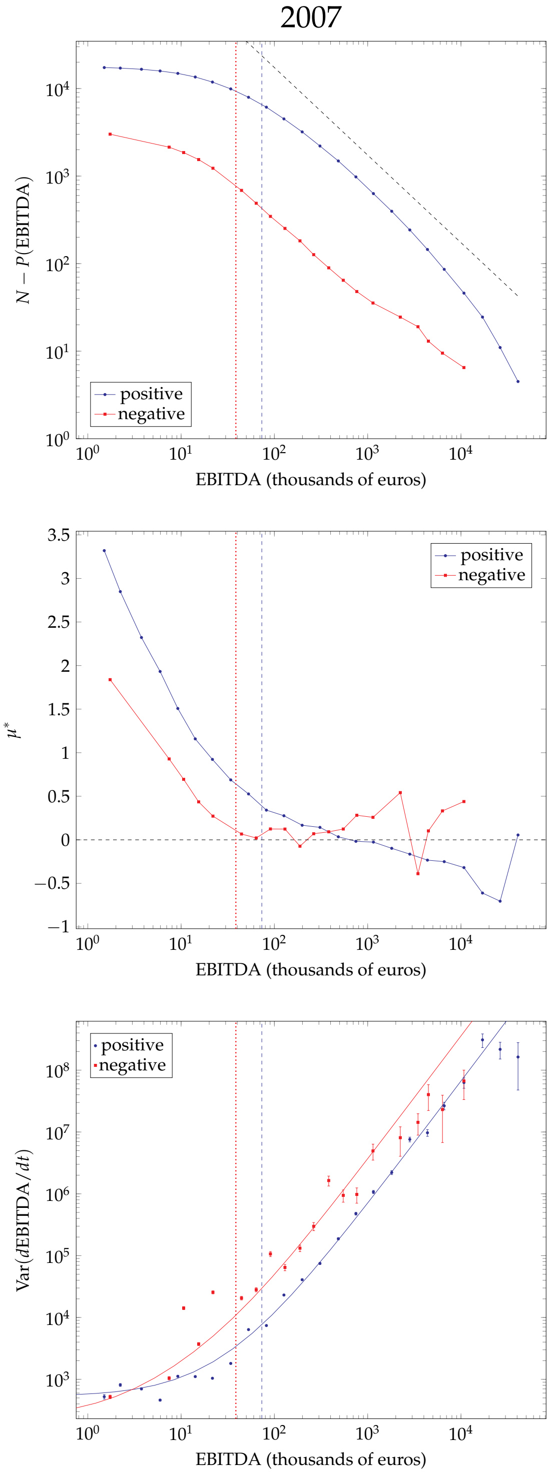

Figure 25: **Aragon 2007: Rank plot, chemical potential and variance**.
Positive EBITDA: 17733 firms.
$T_1 = 0.66 \pm 0.10$, $T_{1/2}$= 48.46 ± 11.34, and T_0 =530.42 ± 68.84
Negative EBITDA: 3434 firms.
$T_1 = 3.56 \pm 0.86$, $T_{1/2}$= 137.65 ± 49.16, and T_0 =238.89 ± 144.51.
Total active firms 21167, total created firms 1451, and total destroyed firms 4546

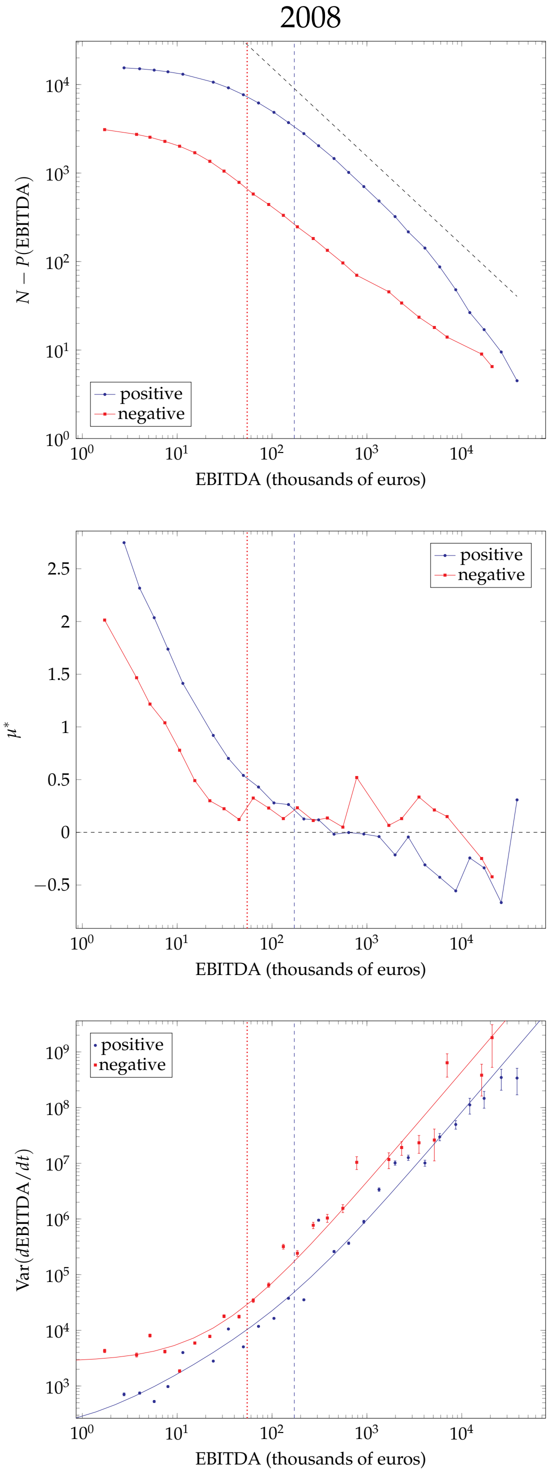

Figure 26: **Aragon 2008: Rank plot, chemical potential and variance**.
Positive EBITDA: 16136 firms.
T_1 = 0.82 ± 0.17, $T_{1/2}$= 141.01 ± 55.06, and T_0 =144.50 ± 363.24
Negative EBITDA: 3457 firms.
T_1 = 4.39 ± 1.02, $T_{1/2}$= 240.29 ± 176.30, and T_0 =2709.27 ± 1646.11.
Total active firms 19593, total created firms 4711, and total destroyed firms 3105

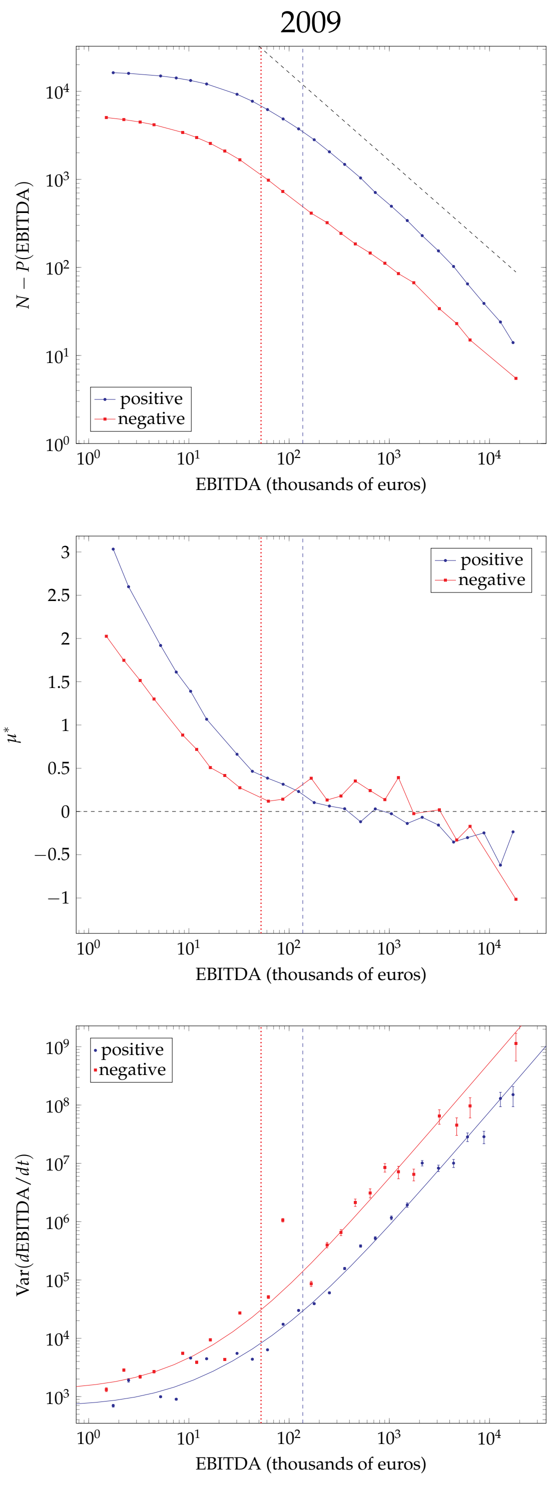

Figure 27: **Aragon 2009: Rank plot, chemical potential and variance**.
Positive EBITDA: 16849 firms.
$T_1 = 0.76 \pm 0.23$, $T_{1/2} = 104.25 \pm 30.08$, and $T_0 = 670.95 \pm 201.43$
Negative EBITDA: 5562 firms.
$T_1 = 5.32 \pm 1.90$, $T_{1/2} = 279.35 \pm 124.88$, and $T_0 = 1272.96 \pm 560.03$.
Total active firms 22411, total created firms 2229, and total destroyed firms 1774

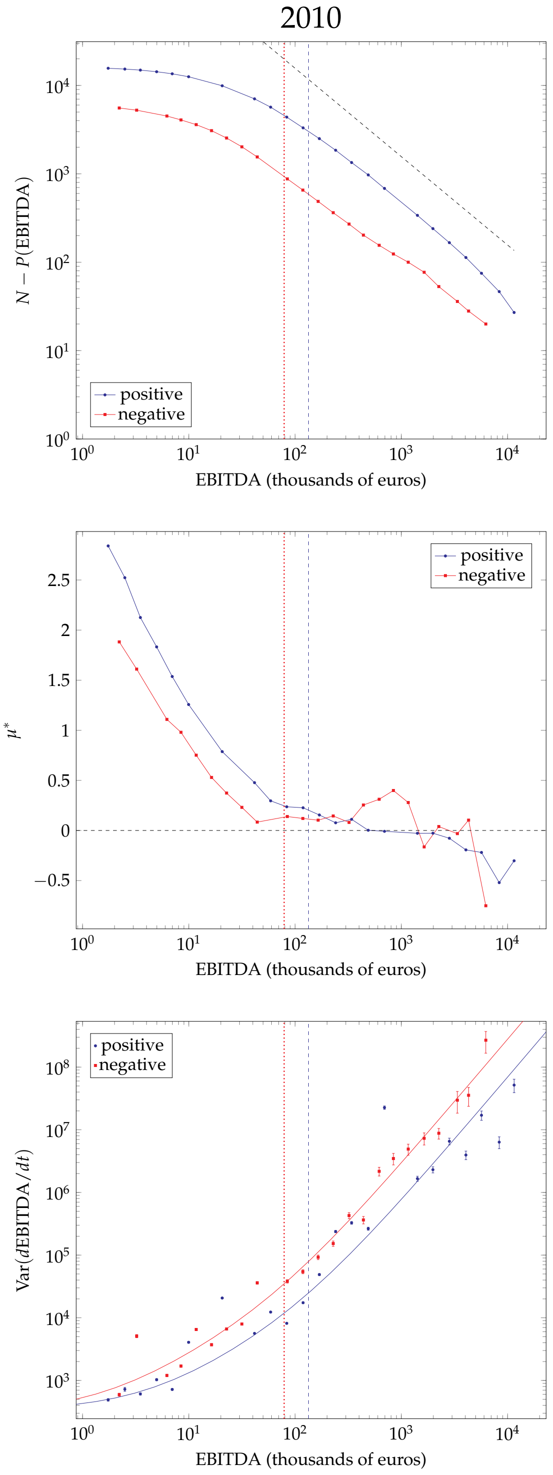

Figure 28: **Aragon 2010: Rank plot, chemical potential and variance.**
Positive EBITDA: 16338 firms.
$T_1 = 0.68 \pm 0.23$, $T_{1/2}$= 91.60 ± 29.66, and T_0 =336.24 ± 119.05
Negative EBITDA: 6422 firms.
$T_1 = 2.77 \pm 0.98$, $T_{1/2}$= 218.34 ± 70.10, and T_0 =309.62 ± 306.88.
Total active firms 22760, total created firms 1983, and total destroyed firms 1825

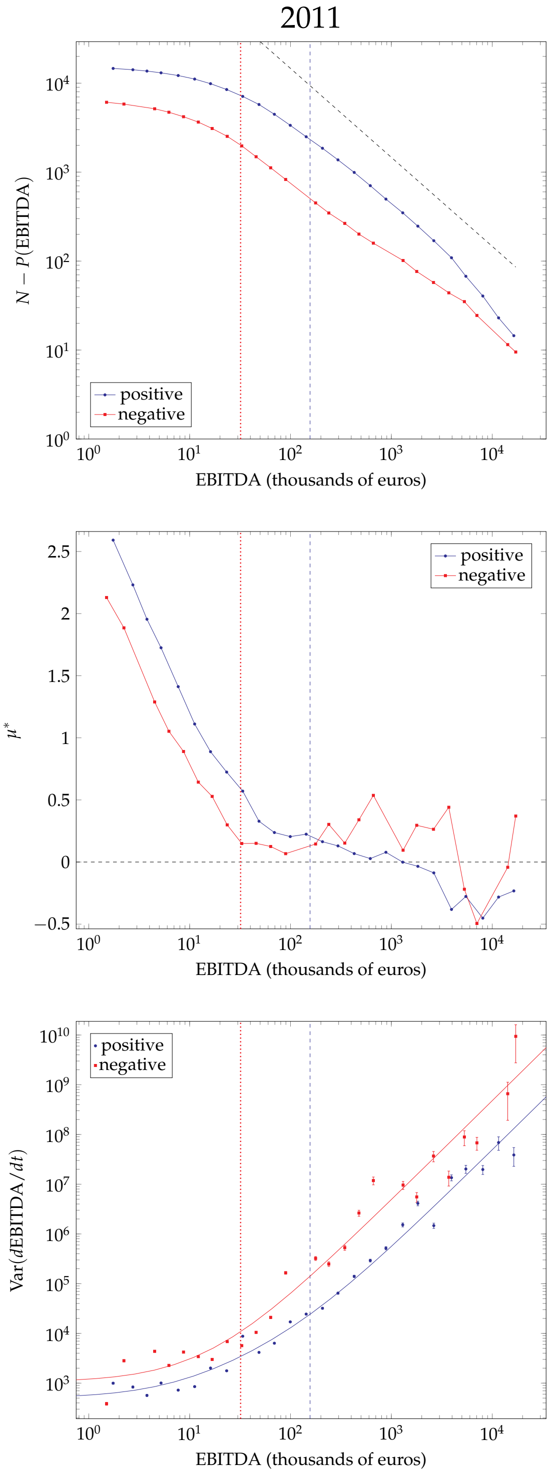

Figure 29: **Aragon 2011: Rank plot, chemical potential and variance**.
Positive EBITDA: 15382 firms.
$T_1 = 0.49 \pm 0.08$, $T_{1/2}$= 75.87 ± 24.28, and T_0 =498.30 ± 210.02
Negative EBITDA: 6670 firms.
$T_1 = 4.77 \pm 1.29$, $T_{1/2}$= 152.64 ± 142.59, and T_0 =1046.97 ± 901.70.
Total active firms 22052, total created firms 1063, and total destroyed firms 2730

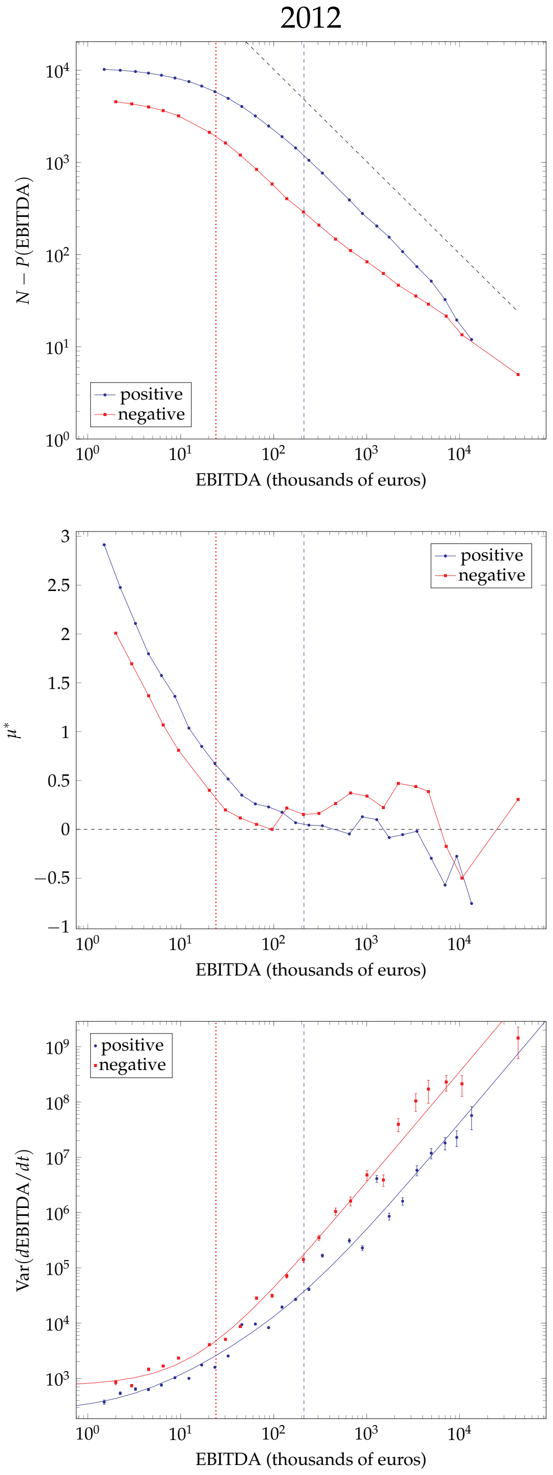

Figure 30: **Aragon 2012: Rank plot, chemical potential and variance.**
Positive EBITDA: 10568 firms.
T_1 = 0.41 ± 0.07, $T_{1/2}$= 87.57 ± 20.77, and T_0 =256.19 ± 133.82
Negative EBITDA: 5052 firms.
T_1 = 3.52 ± 0.59, $T_{1/2}$= 84.13 ± 68.33, and T_0 =726.11 ± 413.79.
Total active firms 15620, total created firms 268, and total destroyed firms 7615

Asturias

Tables of Temperatures

Positive EBITDA

Year	T_1	$T_{1/2}$	T_0	$T_{1/2}/T_1$	Num. Firms
2003	0.44 ± 0.15	25.85 ± 12.65	171.97 ± 147.62	59.4	7937
2004	0.54 ± 0.14	38.69 ± 11.54	120.36 ± 99.32	71.7	7760
2005	0.17 ± 0.07	55.57 ± 10.03	102.72 ± 122.03	319.4	8147
2006	0.55 ± 0.09	53.01 ± 10.24	118.38 ± 98.60	96.3	8683
2007	0.70 ± 0.14	23.76 ± 13.44	463.13 ± 183.31	34.0	8674
2008	0.60 ± 0.11	110.90 ± 34.75	182.00 ± 164.30	185.4	8255
2009	0.58 ± 0.11	62.41 ± 11.19	206.71 ± 104.90	108.3	9567
2010	0.36 ± 0.07	105.89 ± 28.71	268.63 ± 178.68	297.0	9436
2011	0.59 ± 0.17	82.85 ± 51.48	809.33 ± 496.68	139.7	8549
2012	0.60 ± 0.20	64.63 ± 25.95	590.66 ± 178.57	107.9	5688

Negative EBITDA

Year	T_1	$T_{1/2}$	T_0	$T_{1/2}/T_1$	Num. Firms
2003	2.88 ± 1.81	57.91 ± 481.34	545.44 ± 469.65	20.1	1984
2004	2.51 ± 0.57	115.26 ± 216.56	198.52 ± 177.70	46.0	1902
2005	2.28 ± 0.80	162.21 ± 297.20	550.88 ± 278.46	71.2	1877
2006	2.39 ± 1.07	100.20 ± 330.97	449.52 ± 334.43	42.0	2064
2007	3.38 ± 2.13	339.62 ± 1085.40	0.00 ± 377.03	100.4	1712
2008	3.28 ± 1.10	193.87 ± 1127.04	1737.19 ± 1123.24	59.1	1729
2009	3.53 ± 1.21	70.61 ± 215.23	828.35 ± 266.33	20.0	3214
2010	2.77 ± 0.60	48.23 ± 115.30	1045.07 ± 202.44	17.4	3789
2011	3.12 ± 0.44	81.67 ± 195.54	530.82 ± 216.41	26.2	4130
2012	2.22 ± 0.61	110.59 ± 168.46	261.53 ± 133.01	49.9	3223

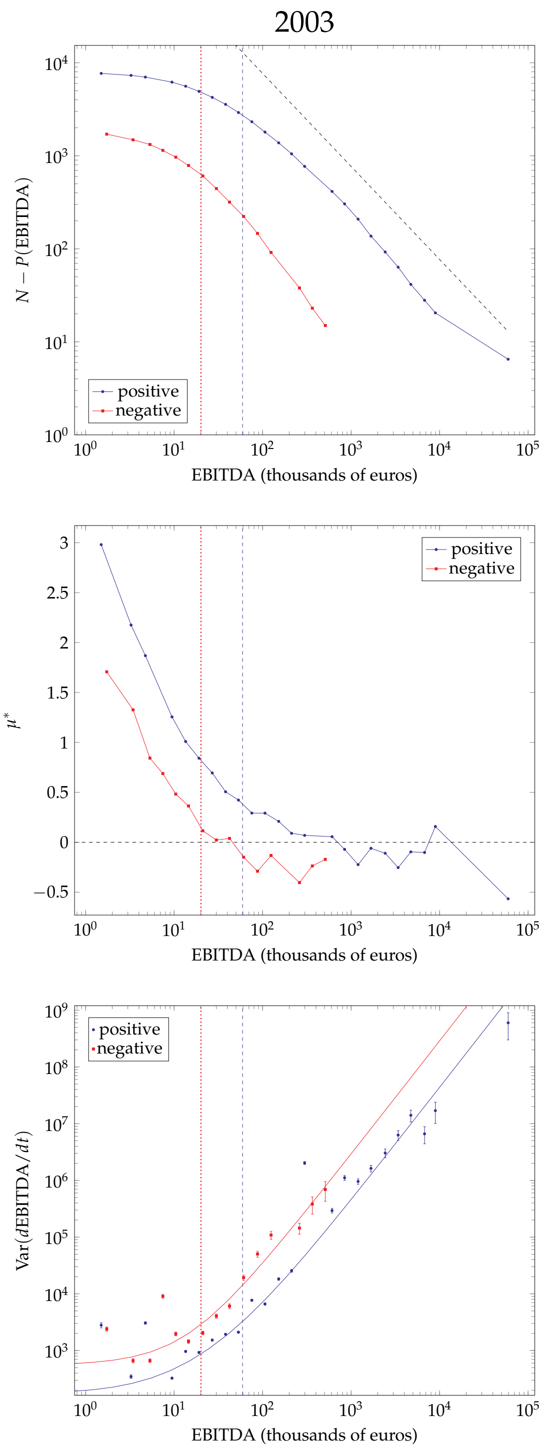

Figure 31: **Asturias 2003: Rank plot, chemical potential and variance**.
Positive EBITDA: 7937 firms.
$T_1 = 0.44 \pm 0.15$, $T_{1/2}= 25.85 \pm 12.65$, and $T_0 =171.97 \pm 147.62$
Negative EBITDA: 1984 firms.
$T_1 = 2.88 \pm 1.81$, $T_{1/2}= 57.91 \pm 78.85$, and $T_0 =545.44 \pm 469.65$.
Total active firms 9921, total created firms 1424, and total destroyed firms 1435

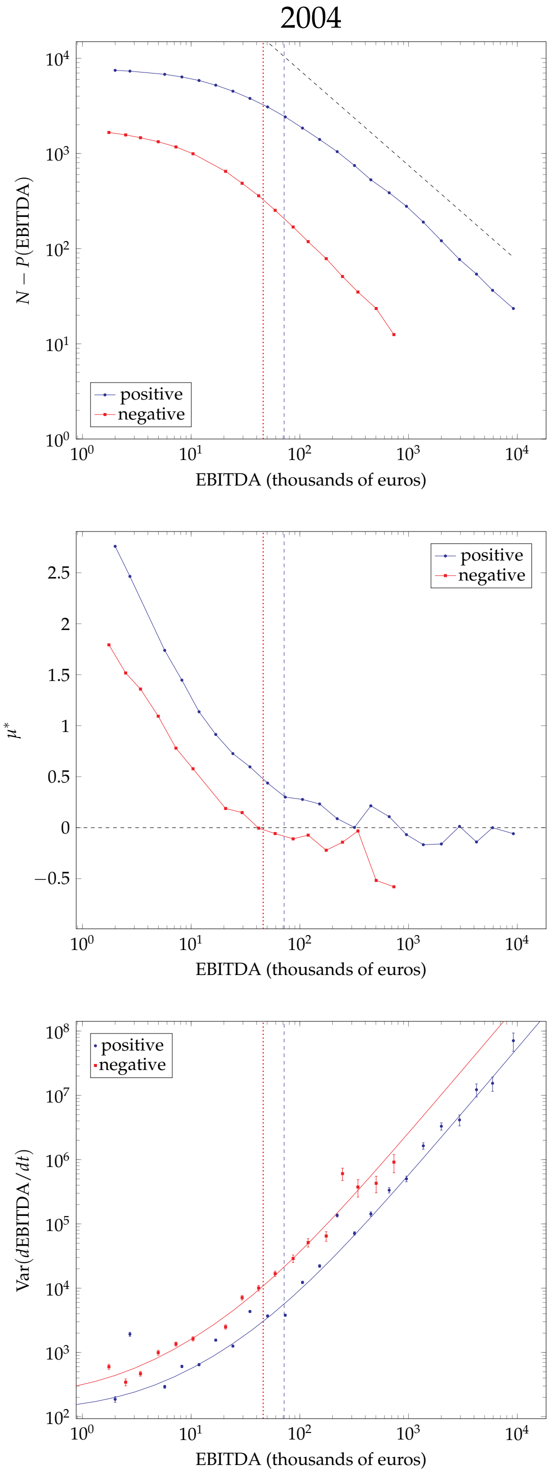

Figure 32: **Asturias 2004: Rank plot, chemical potential and variance.**
Positive EBITDA: 7760 firms.
T_1 = 0.54 ± 0.14, $T_{1/2}$= 38.69 ± 11.54, and T_0 =120.36 ± 99.32
Negative EBITDA: 1902 firms.
T_1 = 2.51 ± 0.57, $T_{1/2}$= 115.26 ± 44.82, and T_0 =198.52 ± 177.70.
Total active firms 9662, total created firms 1409, and total destroyed firms 1713

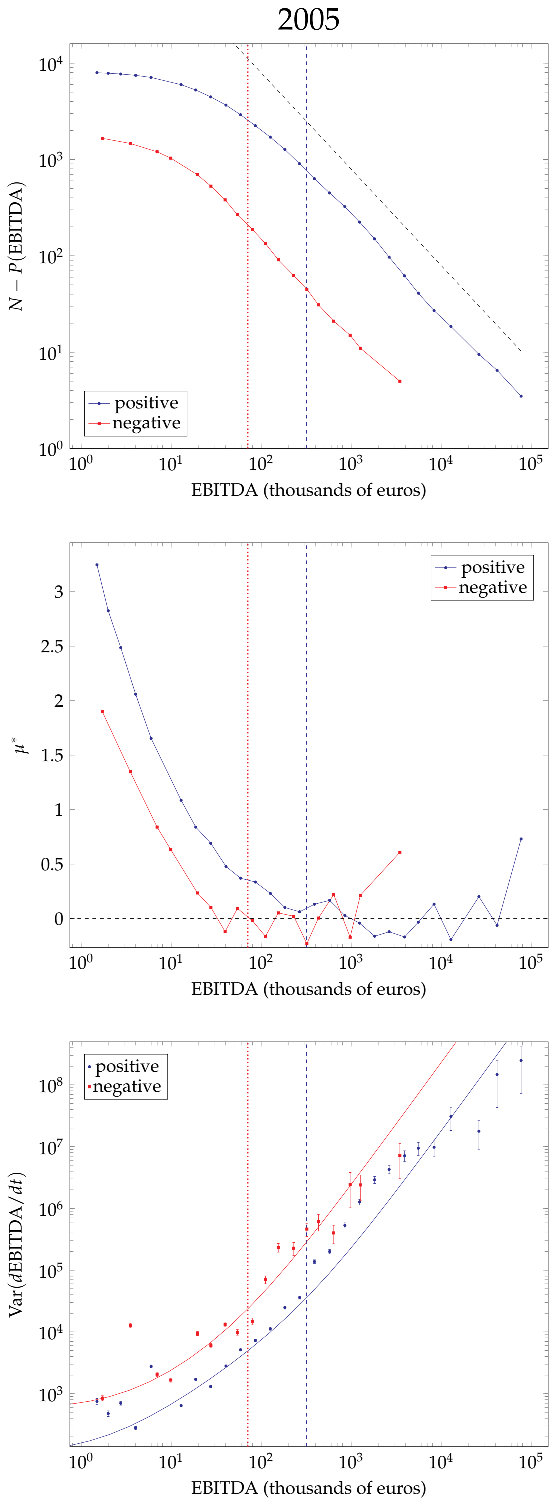

Figure 33: **Asturias 2005: Rank plot, chemical potential and variance**.
Positive EBITDA: 8147 firms.
$T_1 = 0.17 \pm 0.07$, $T_{1/2} = 55.57 \pm 10.03$, and $T_0 = 102.72 \pm 122.03$
Negative EBITDA: 1877 firms.
$T_1 = 2.28 \pm 0.80$, $T_{1/2} = 162.21 \pm 56.07$, and $T_0 = 550.88 \pm 278.46$.
Total active firms 10024, total created firms 1724, and total destroyed firms 1063

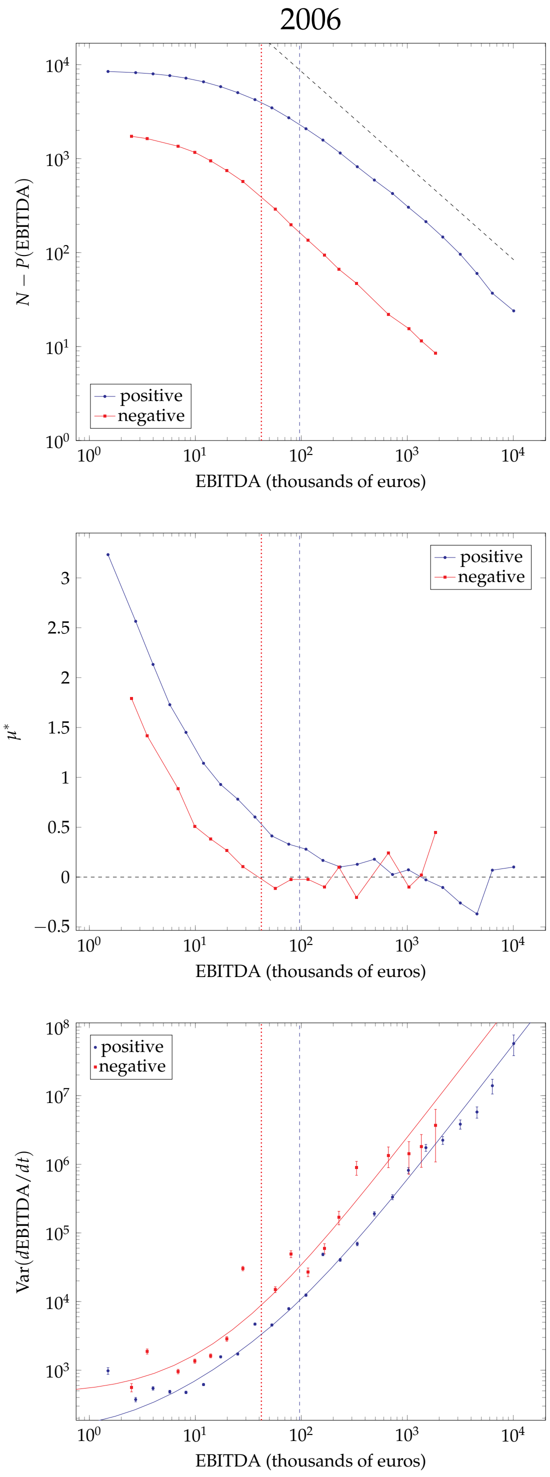

Figure 34: **Asturias 2006: Rank plot, chemical potential and variance**.
Positive EBITDA: 8683 firms.
T_1 = 0.55 ± 0.09, $T_{1/2}$= 53.01 ± 10.24, and T_0 =118.38 ± 98.60
Negative EBITDA: 2064 firms.
T_1 = 2.39 ± 1.07, $T_{1/2}$= 100.20 ± 60.50, and T_0 =449.52 ± 334.43.
Total active firms 10747, total created firms 1568, and total destroyed firms 975

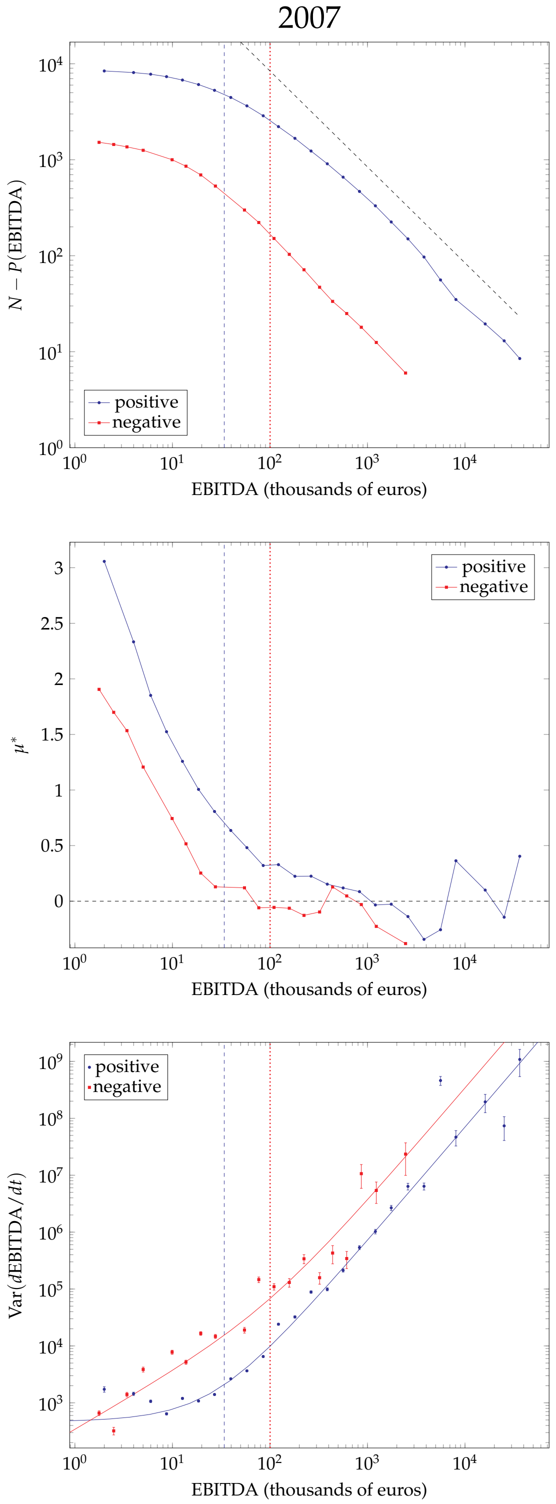

Figure 35: **Asturias 2007: Rank plot, chemical potential and variance**.
Positive EBITDA: 8674 firms.
T_1 = 0.70 ± 0.14, $T_{1/2}$= 23.76 ± 13.44, and T_0 =463.13 ± 183.31
Negative EBITDA: 1712 firms.
T_1 = 3.38 ± 2.13, $T_{1/2}$= 339.62 ± 140.12, and T_0 =0.00 ± 377.03.
Total active firms 10386, total created firms 829, and total destroyed firms 1944

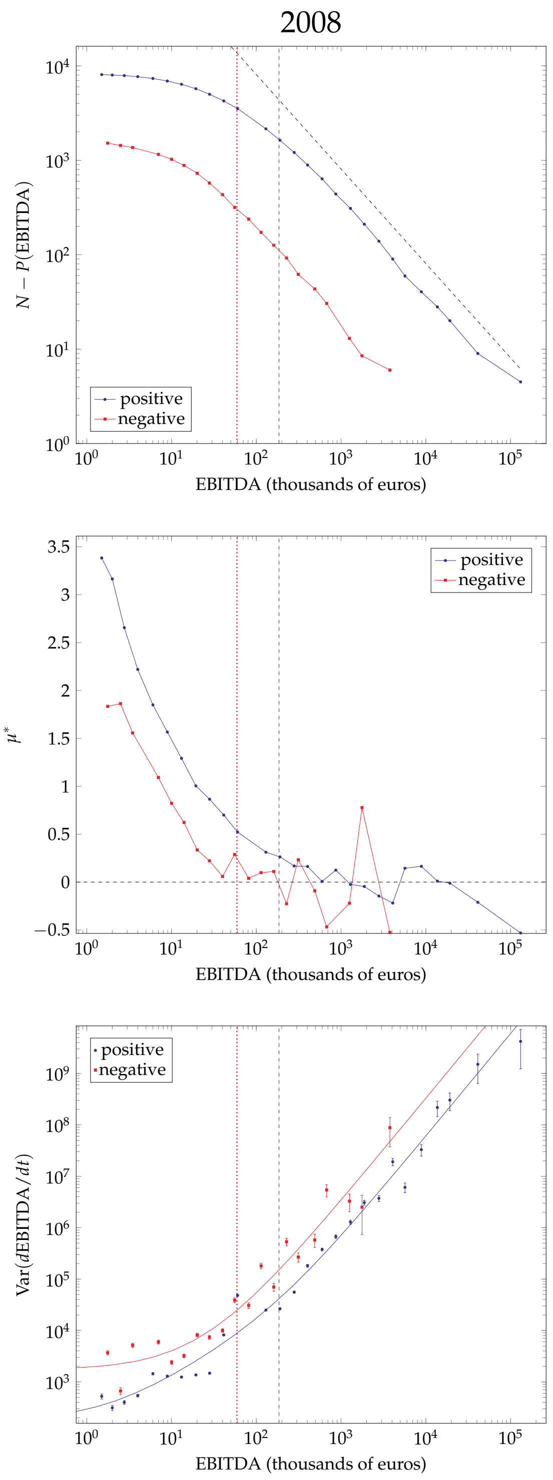

Figure 36: **Asturias 2008: Rank plot, chemical potential and variance**.
Positive EBITDA: 8255 firms.
T_1 = 0.60 ± 0.11, $T_{1/2}$= 110.90 ± 34.75, and T_0 =182.00 ± 164.30
Negative EBITDA: 1729 firms.
T_1 = 3.28 ± 1.10, $T_{1/2}$= 193.87 ± 143.90, and T_0 =1737.19 ± 1123.24.
Total active firms 9984, total created firms 3818, and total destroyed firms 1281

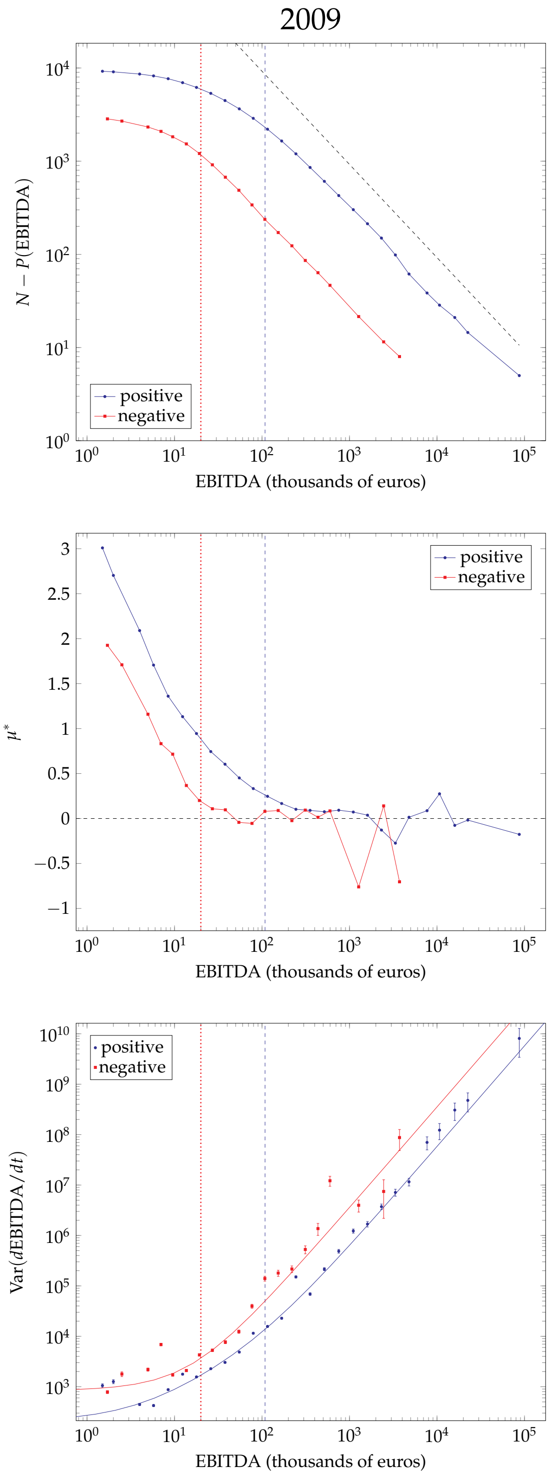

Figure 37: **Asturias 2009: Rank plot, chemical potential and variance**.
Positive EBITDA: 9567 firms.
T_1 = 0.58 ± 0.11, $T_{1/2}$= 62.41 ± 11.19, and T_0 =206.71 ± 104.90
Negative EBITDA: 3214 firms.
T_1 = 3.53 ± 1.21, $T_{1/2}$= 70.61 ± 44.63, and T_0 =828.35 ± 266.33.
Total active firms 12781, total created firms 1599, and total destroyed firms 914

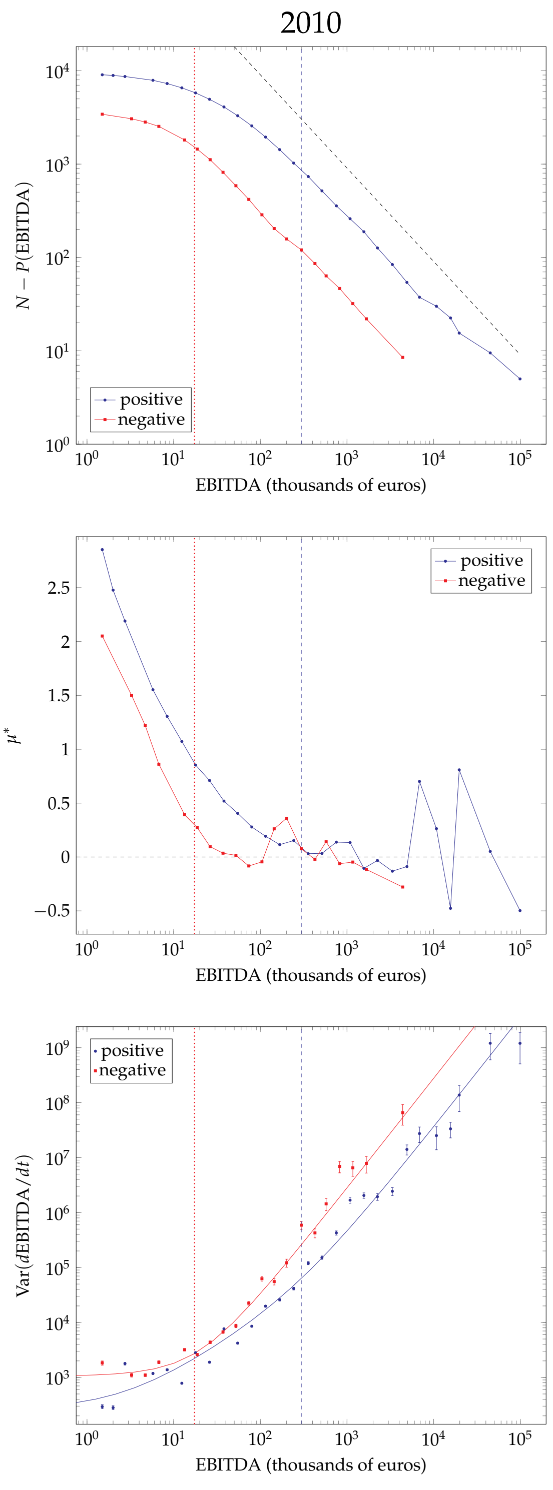

Figure 38: **Asturias 2010: Rank plot, chemical potential and variance**.
Positive EBITDA: 9436 firms.
$T_1 = 0.36 \pm 0.07$, $T_{1/2} = 105.89 \pm 28.71$, and $T_0 = 268.63 \pm 178.68$
Negative EBITDA: 3789 firms.
$T_1 = 2.77 \pm 0.60$, $T_{1/2} = 48.23 \pm 28.70$, and $T_0 = 1045.07 \pm 202.44$.
Total active firms 13225, total created firms 1004, and total destroyed firms 1134

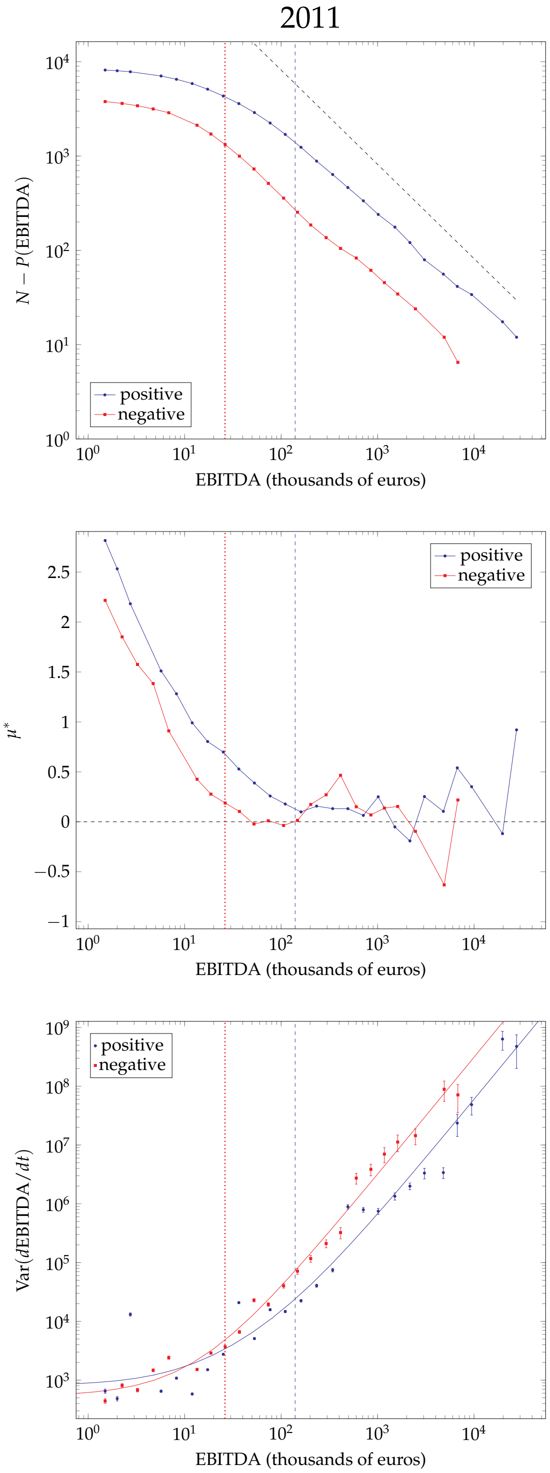

Figure 39: **Asturias 2011: Rank plot, chemical potential and variance**.
Positive EBITDA: 8549 firms.
$T_1 = 0.59 \pm 0.17$, $T_{1/2}$= 82.85 ± 51.48, and T_0 =809.33 ± 496.68
Negative EBITDA: 4130 firms.
$T_1 = 3.12 \pm 0.44$, $T_{1/2}$= 81.67 ± 41.70, and T_0 =530.82 ± 216.41.
Total active firms 12679, total created firms 640, and total destroyed firms 1595

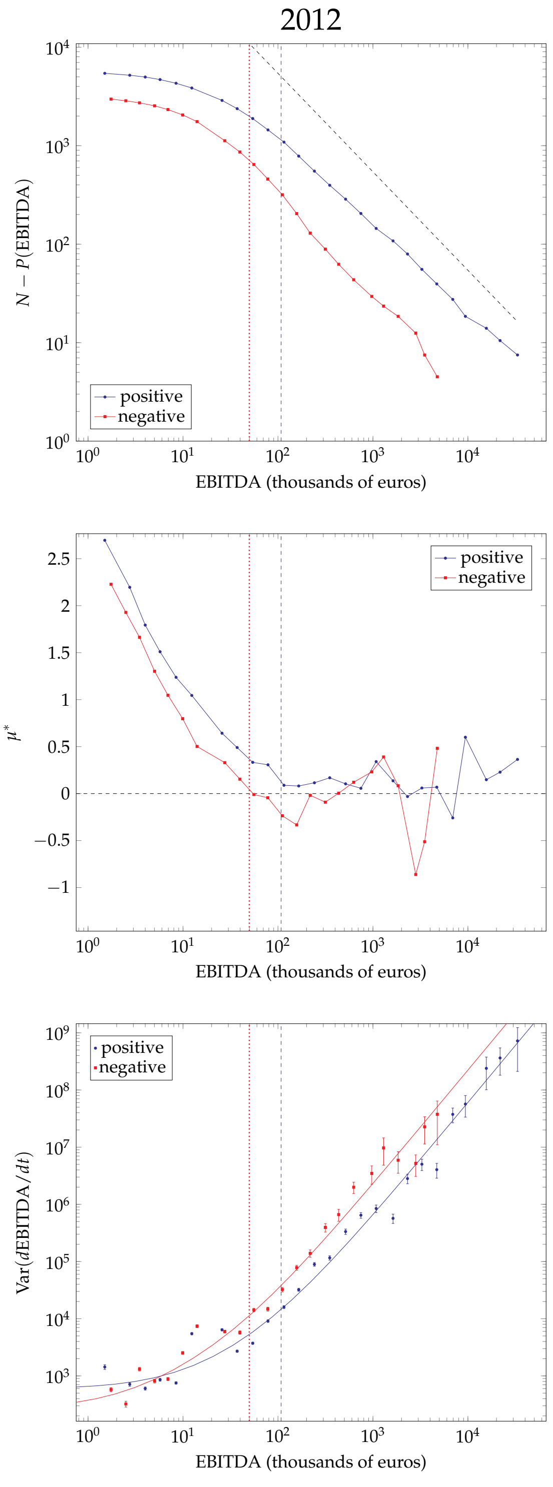

Figure 40: **Asturias 2012: Rank plot, chemical potential and variance.**
Positive EBITDA: 5688 firms.
$T_1 = 0.60 \pm 0.20$, $T_{1/2}$= 64.63 ± 25.95, and T_0 =590.66 ± 178.57
Negative EBITDA: 3223 firms.
$T_1 = 2.22 \pm 0.61$, $T_{1/2}$= 110.59 ± 37.53, and T_0 =261.53 ± 133.01.
Total active firms 8911, total created firms 159, and total destroyed firms 4483

Balearic islands

Tables of Temperatures

Positive EBITDA

Year	T_1	$T_{1/2}$	T_0	$T_{1/2}/T_1$	Num. Firms
2003	0.35 ± 0.12	67.29 ± 12.51	175.21 ± 55.51	189.9	11636
2004	0.39 ± 0.06	55.87 ± 13.96	230.93 ± 116.16	142.4	11800
2005	0.47 ± 0.12	57.26 ± 36.09	706.13 ± 356.48	120.7	12071
2006	0.59 ± 0.12	49.22 ± 25.92	462.70 ± 229.22	84.1	12450
2007	0.50 ± 0.15	52.68 ± 26.65	1373.82 ± 348.57	106.1	12081
2008	0.67 ± 0.22	68.05 ± 31.34	1286.32 ± 326.83	101.4	10794
2009	1.20 ± 0.26	115.87 ± 55.47	525.06 ± 319.26	96.7	13852
2010	0.47 ± 0.10	149.77 ± 41.24	293.81 ± 265.67	318.8	13145
2011	0.61 ± 0.13	95.07 ± 40.19	480.17 ± 307.09	155.4	12437
2012	0.59 ± 0.09	27.77 ± 17.72	1165.66 ± 250.28	47.2	5051

Negative EBITDA

Year	T_1	$T_{1/2}$	T_0	$T_{1/2}/T_1$	Num. Firms
2003	2.66 ± 1.19	115.28 ± 381.58	333.12 ± 253.94	43.3	3333
2004	1.69 ± 0.58	160.06 ± 202.71	474.74 ± 175.67	94.8	3243
2005	3.42 ± 0.65	160.83 ± 280.76	382.51 ± 238.18	47.0	3221
2006	2.86 ± 0.60	72.04 ± 353.35	1198.82 ± 486.38	25.2	3393
2007	2.01 ± 0.50	191.59 ± 192.20	672.13 ± 208.06	95.4	2875
2008	2.96 ± 1.02	256.69 ± 452.09	842.41 ± 324.93	86.8	2682
2009	2.67 ± 0.45	92.50 ± 347.42	1987.90 ± 616.93	34.7	5507
2010	2.27 ± 0.54	159.25 ± 428.95	402.66 ± 337.81	70.1	6033
2011	2.41 ± 0.55	78.20 ± 102.94	477.38 ± 192.76	32.5	5812
2012	1.80 ± 0.40	162.66 ± 388.32	1040.20 ± 462.33	90.3	2197

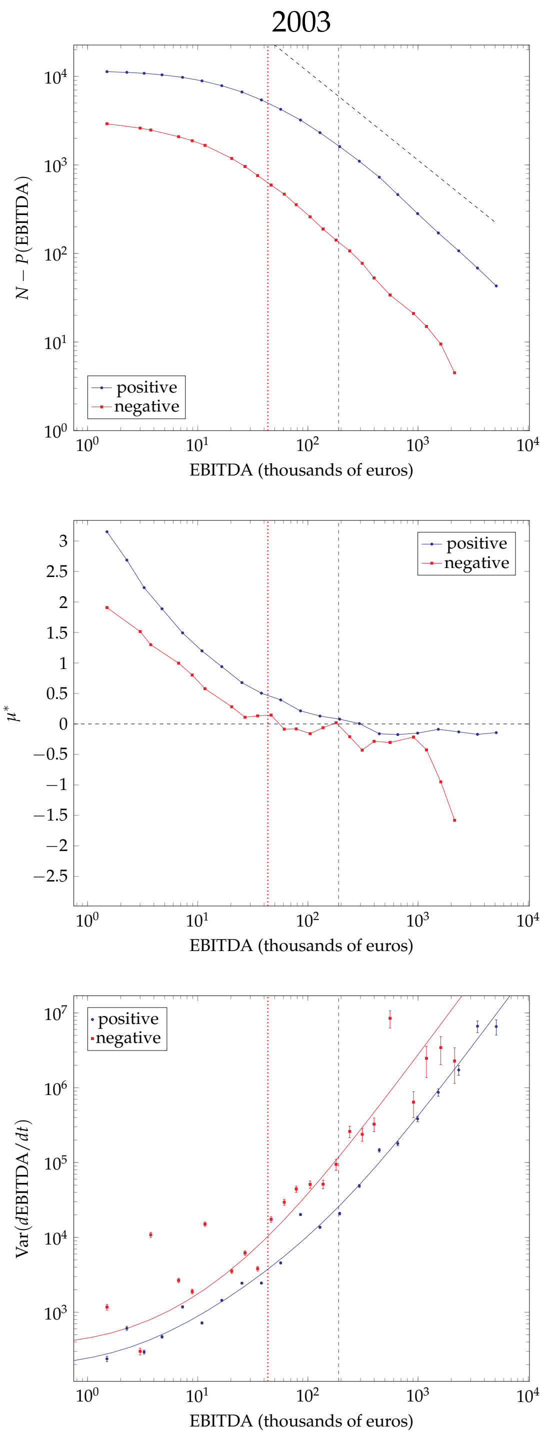

Figure 41: **Balearic islands 2003: Rank plot, chemical potential and variance**.
Positive EBITDA: 11636 firms.
T_1 = 0.35 ± 0.12, $T_{1/2}$= 67.29 ± 12.51, and T_0 =175.21 ± 55.51
Negative EBITDA: 3333 firms.
T_1 = 2.66 ± 1.19, $T_{1/2}$= 115.28 ± 66.91, and T_0 =333.12 ± 253.94.
Total active firms 14969, total created firms 3370, and total destroyed firms 1484

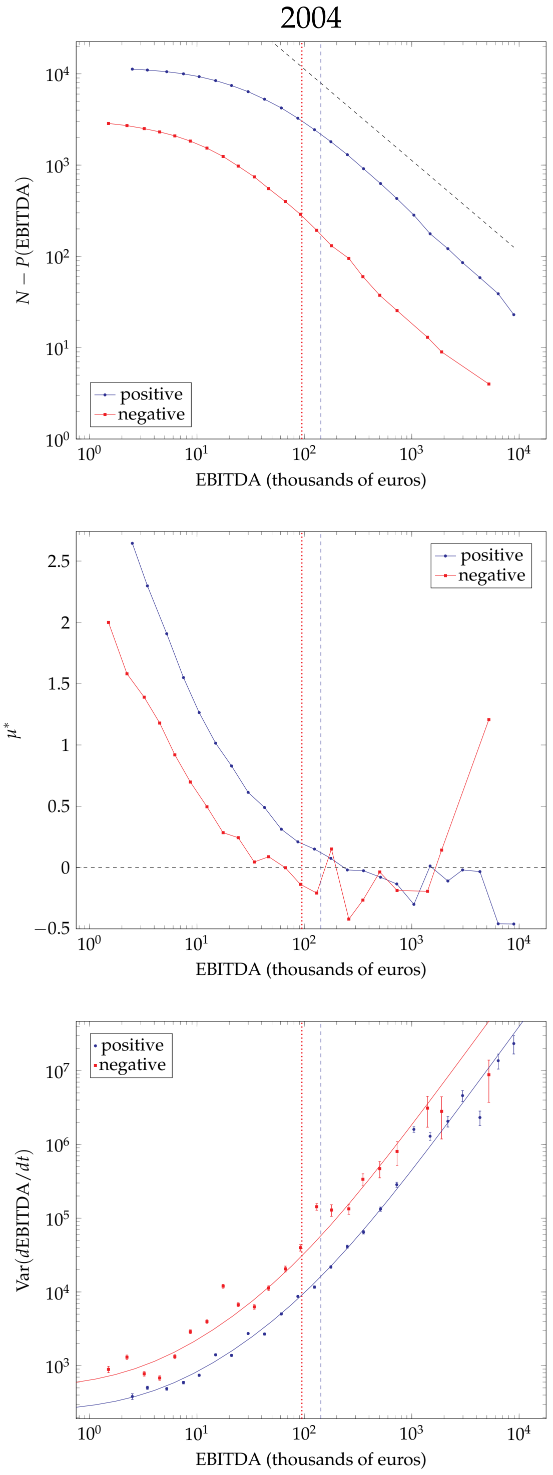

Figure 42: **Balearic islands 2004: Rank plot, chemical potential and variance**.
Positive EBITDA: 11800 firms.
$T_1 = 0.39 \pm 0.06$, $T_{1/2}= 55.87 \pm 13.96$, and $T_0 =230.93 \pm 116.16$
Negative EBITDA: 3243 firms.
$T_1 = 1.69 \pm 0.58$, $T_{1/2}= 160.06 \pm 42.78$, and $T_0 =474.74 \pm 175.67$.
Total active firms 15043, total created firms 2848, and total destroyed firms 3324

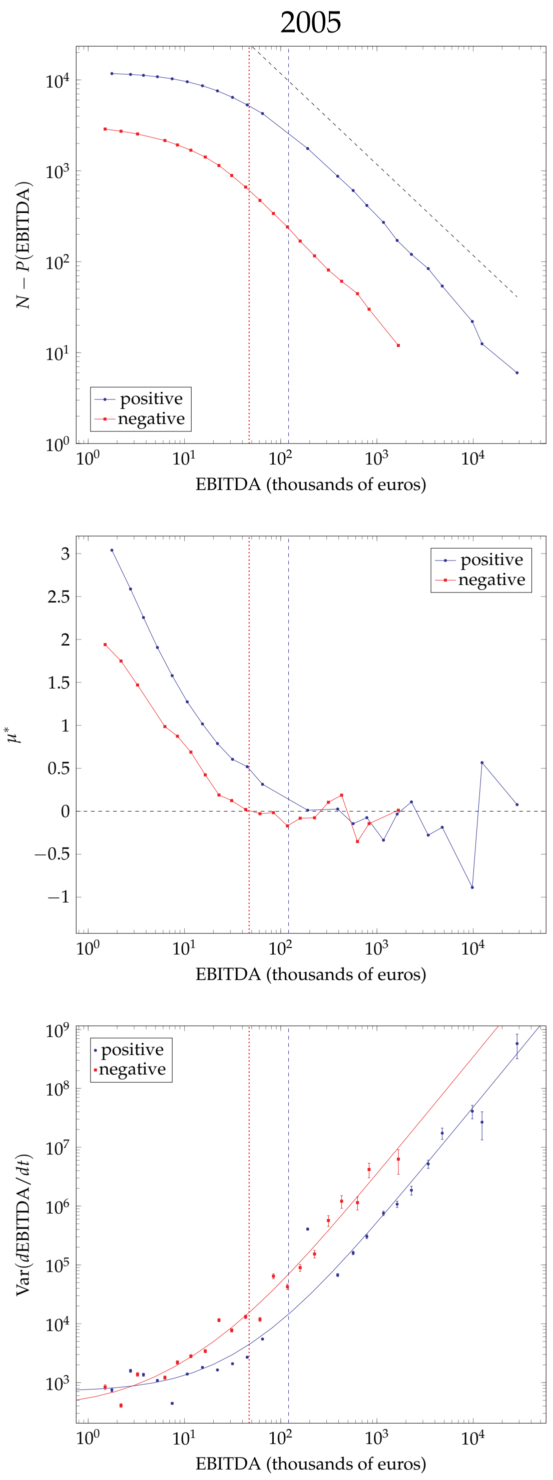

Figure 43: **Balearic islands 2005: Rank plot, chemical potential and variance.**
Positive EBITDA: 12071 firms.
T_1 = 0.47 ± 0.12, $T_{1/2}$= 57.26 ± 36.09, and T_0 =706.13 ± 356.48
Negative EBITDA: 3221 firms.
T_1 = 3.42 ± 0.65, $T_{1/2}$= 160.83 ± 53.86, and T_0 =382.51 ± 238.18.
Total active firms 15292, total created firms 2988, and total destroyed firms 2662

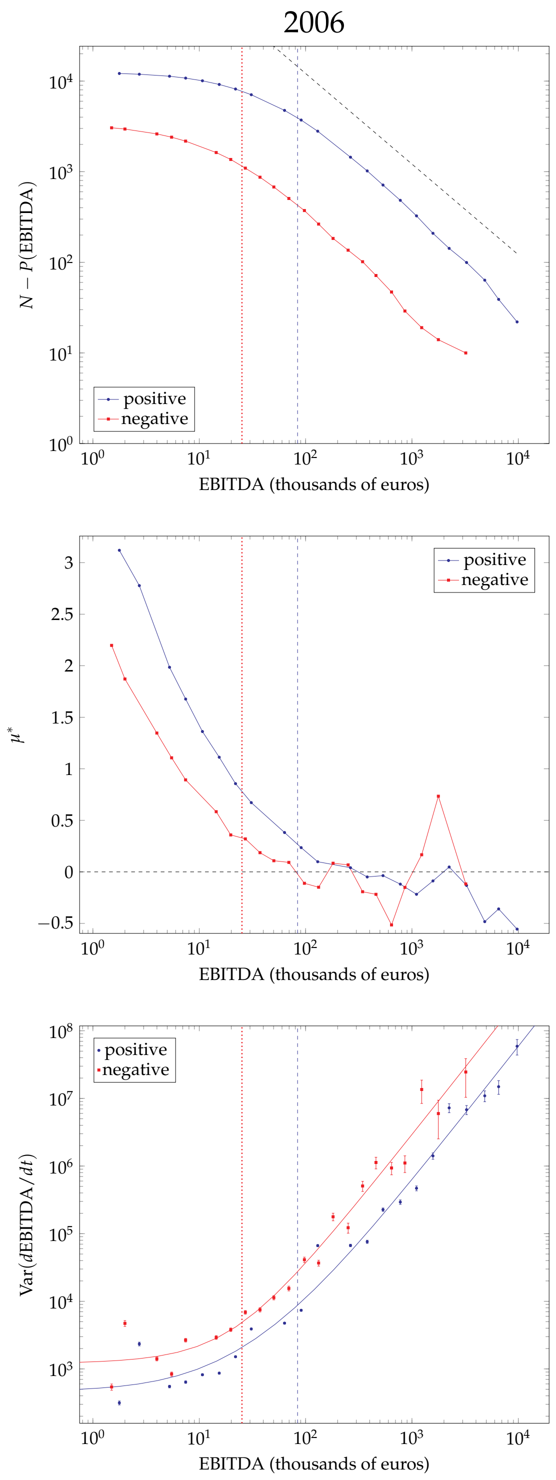

Figure 44: **Balearic islands 2006: Rank plot, chemical potential and variance**.
Positive EBITDA: 12450 firms.
$T_1 = 0.59 \pm 0.12$, $T_{1/2}$= 49.22 ± 25.92, and T_0 =462.70 ± 229.22
Negative EBITDA: 3393 firms.
$T_1 = 2.86 \pm 0.60$, $T_{1/2}$= 72.04 ± 63.37, and T_0 =1198.82 ± 486.38.
Total active firms 15843, total created firms 3075, and total destroyed firms 2426

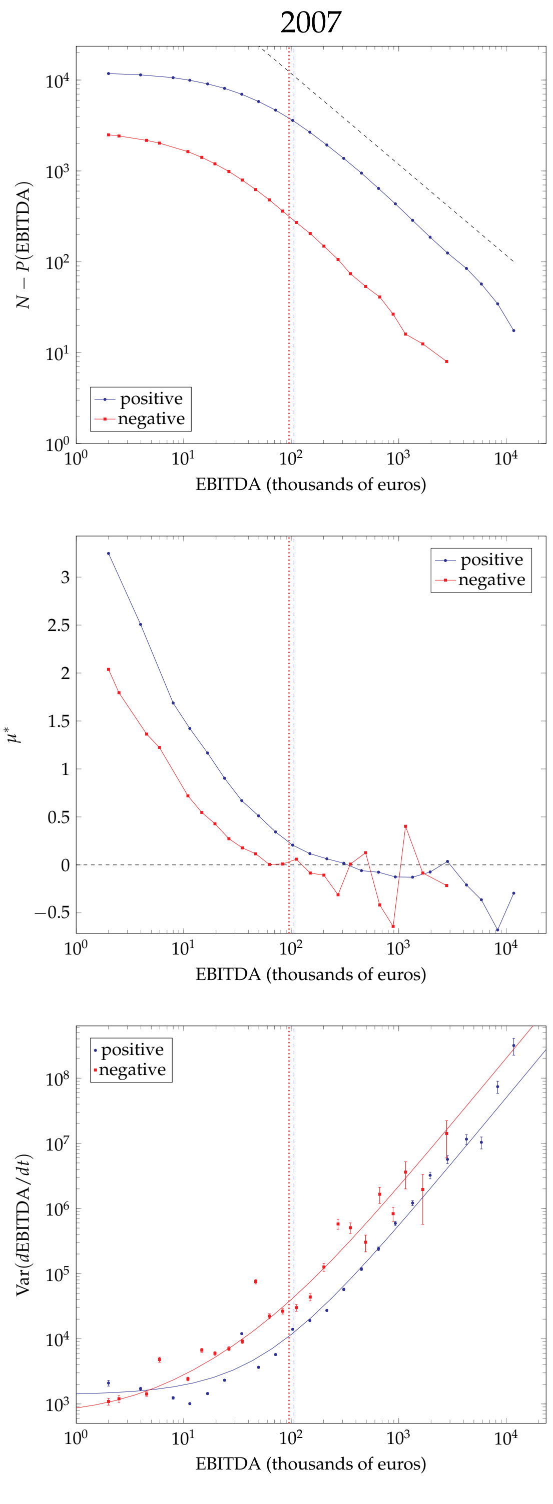

Figure 45: **Balearic islands 2007: Rank plot, chemical potential and variance.**
Positive EBITDA: 12081 firms.
$T_1 = 0.50 \pm 0.15$, $T_{1/2}$= 52.68 ± 26.65, and T_0 =1373.82 ± 348.57
Negative EBITDA: 2875 firms.
$T_1 = 2.01 \pm 0.50$, $T_{1/2}$= 191.59 ± 41.20, and T_0 =672.13 ± 208.06.
Total active firms 14956, total created firms 917, and total destroyed firms 3986

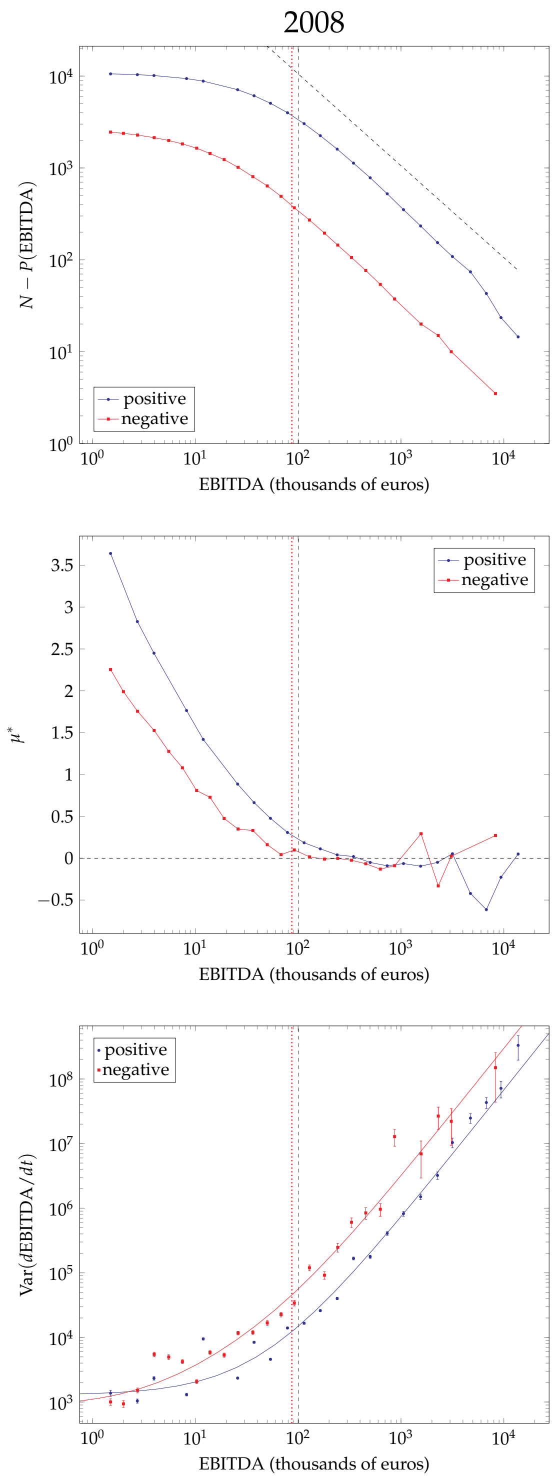

Figure 46: **Balearic islands 2008: Rank plot, chemical potential and variance.**
Positive EBITDA: 10794 firms.
T_1 = 0.67 ± 0.22, $T_{1/2}$= 68.05 ± 31.34, and T_0 =1286.32 ± 326.83
Negative EBITDA: 2682 firms.
T_1 = 2.96 ± 1.02, $T_{1/2}$= 256.69 ± 75.43, and T_0 =842.41 ± 324.93.
Total active firms 13476, total created firms 7673, and total destroyed firms 2474

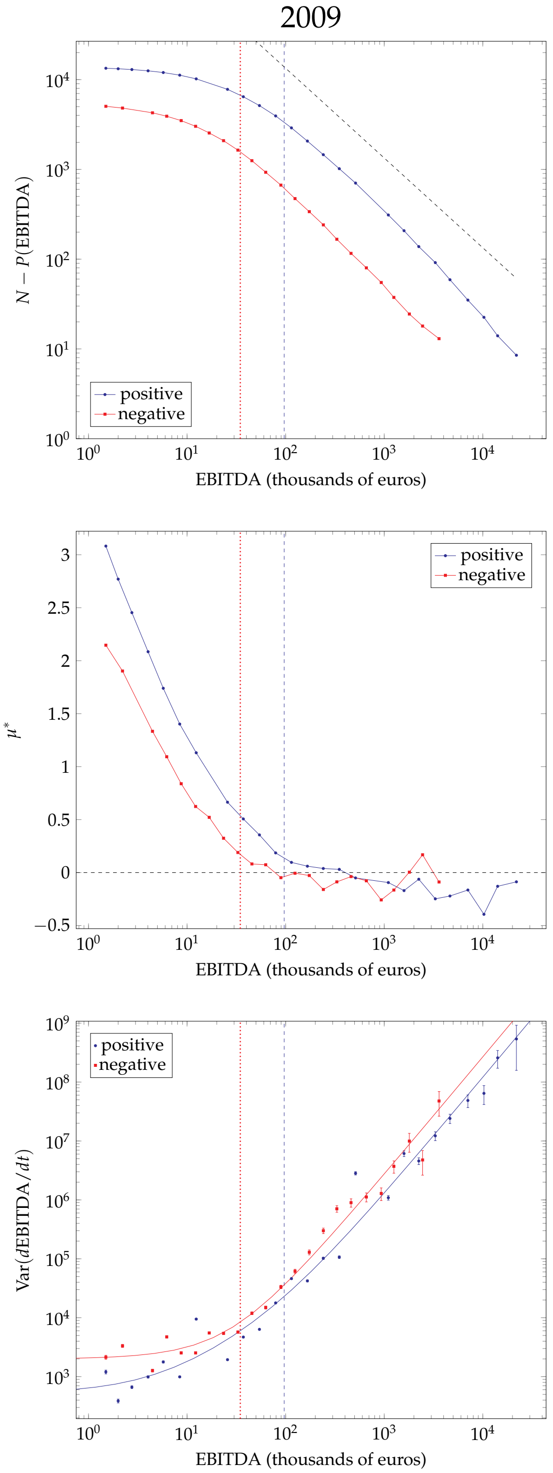

Figure 47: **Balearic islands 2009: Rank plot, chemical potential and variance**.
Positive EBITDA: 13852 firms.
T_1 = 1.20 ± 0.26, $T_{1/2}$= 115.87 ± 55.47, and T_0 =525.06 ± 319.26
Negative EBITDA: 5507 firms.
T_1 = 2.67 ± 0.45, $T_{1/2}$= 92.50 ± 62.61, and T_0 =1987.90 ± 616.93.
Total active firms 19359, total created firms 2615, and total destroyed firms 1652

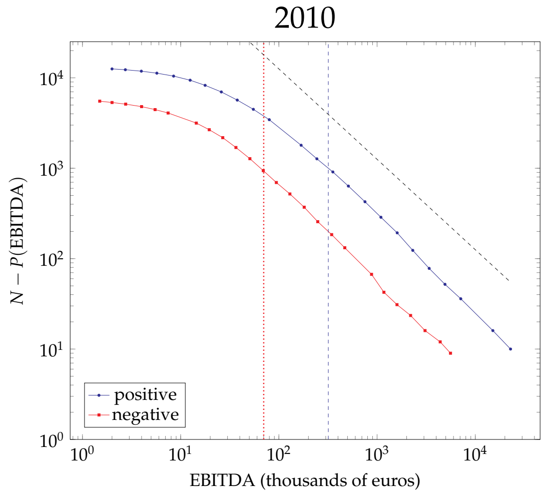

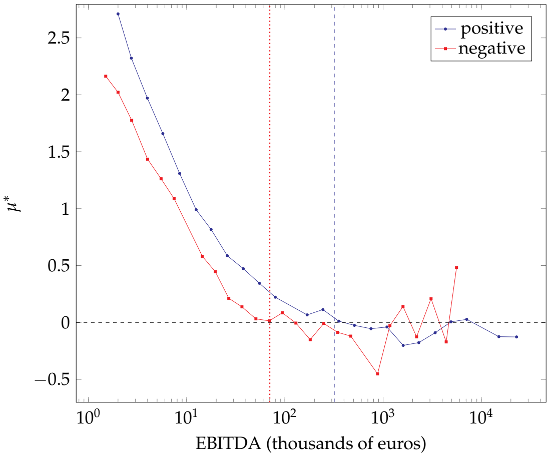

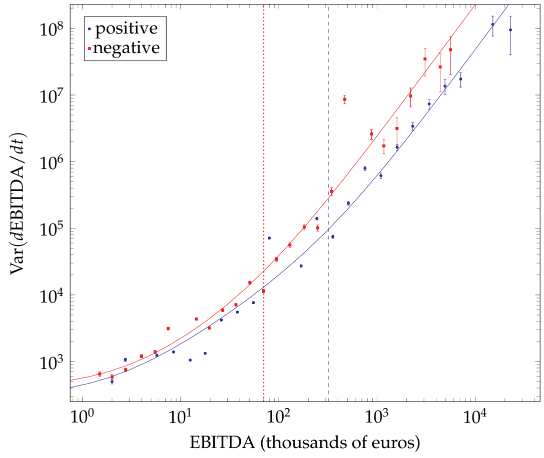

Figure 48: **Balearic islands 2010: Rank plot, chemical potential and variance.**
Positive EBITDA: 13145 firms.
T_1 = 0.47 ± 0.10, $T_{1/2}$= 149.77 ± 41.24, and T_0 =293.81 ± 265.67
Negative EBITDA: 6033 firms.
T_1 = 2.27 ± 0.54, $T_{1/2}$= 159.25 ± 72.68, and T_0 =402.66 ± 337.81.
Total active firms 19178, total created firms 1767, and total destroyed firms 2812

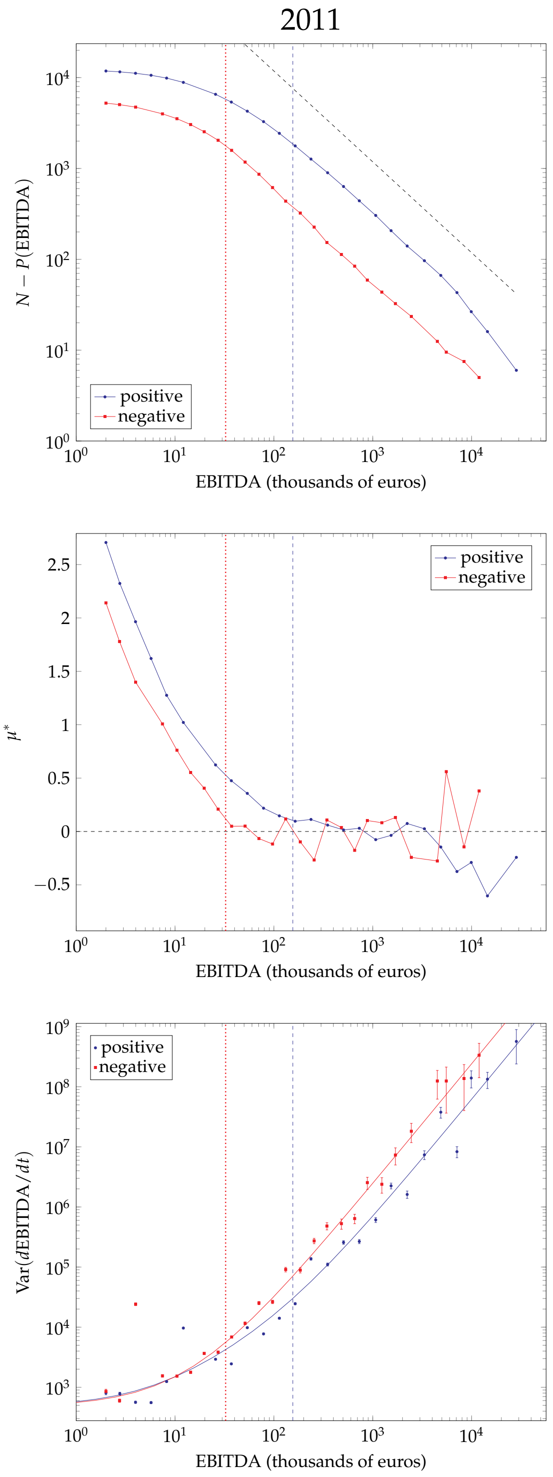

Figure 49: **Balearic islands 2011: Rank plot, chemical potential and variance.**
Positive EBITDA: 12437 firms.
$T_1 = 0.61 \pm 0.13$, $T_{1/2}$= 95.07 ± 40.19, and T_0 =480.17 ± 307.09
Negative EBITDA: 5812 firms.
$T_1 = 2.41 \pm 0.55$, $T_{1/2}$= 78.20 ± 26.49, and T_0 =477.38 ± 192.76.
Total active firms 18249, total created firms 720, and total destroyed firms 2704

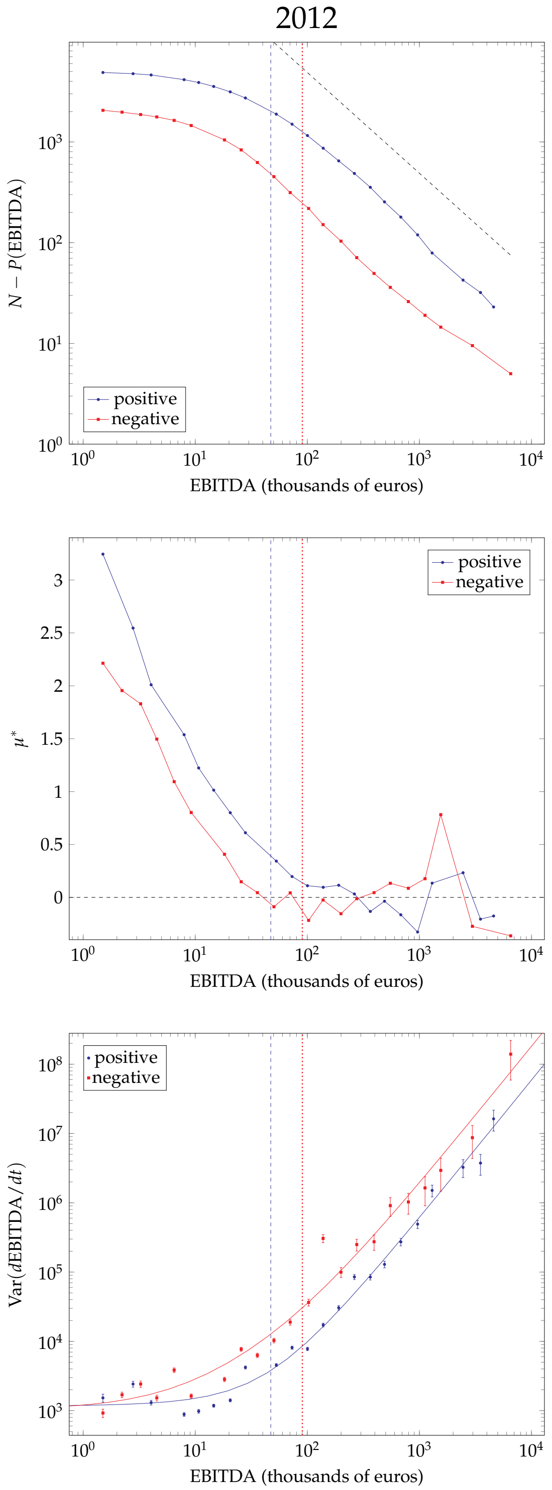

Figure 50: **Balearic islands 2012: Rank plot, chemical potential and variance.**
Positive EBITDA: 5051 firms.
T_1 = 0.59 ± 0.09, $T_{1/2}$= 27.77 ± 17.72, and T_0 =1165.66 ± 250.28
Negative EBITDA: 2197 firms.
T_1 = 1.80 ± 0.40, $T_{1/2}$= 162.66 ± 67.74, and T_0 =1040.20 ± 462.33.
Total active firms 7248, total created firms 80, and total destroyed firms 11887

Basque country

Tables of Temperatures

Positive EBITDA

Year	T_1	$T_{1/2}$	T_0	$T_{1/2}/T_1$	Num. Firms
2003	0.15 ± 0.05	81.64 ± 11.70	160.31 ± 140.66	560.8	19408
2004	0.48 ± 0.08	75.53 ± 10.10	331.88 ± 69.73	157.9	19878
2005	0.53 ± 0.12	62.88 ± 18.46	931.98 ± 193.65	118.5	20989
2006	0.50 ± 0.10	81.12 ± 13.05	293.46 ± 129.12	161.4	22313
2007	0.52 ± 0.10	57.02 ± 13.33	377.29 ± 199.63	108.8	22219
2008	0.94 ± 0.43	104.39 ± 50.47	882.63 ± 345.70	111.0	20441
2009	1.06 ± 0.15	159.64 ± 53.31	1712.80 ± 596.21	149.9	21588
2010	0.76 ± 0.18	259.13 ± 98.51	548.52 ± 571.50	342.6	22587
2011	1.00 ± 0.18	299.45 ± 91.85	1145.89 ± 757.37	298.8	20060
2012	0.53 ± 0.14	107.92 ± 51.95	587.59 ± 438.80	202.6	12961

Negative EBITDA

Year	T_1	$T_{1/2}$	T_0	$T_{1/2}/T_1$	Num. Firms
2003	1.76 ± 0.51	252.41 ± 456.99	944.56 ± 409.07	143.0	4885
2004	1.63 ± 0.49	121.61 ± 258.35	1275.44 ± 392.30	74.4	5007
2005	2.94 ± 0.73	184.49 ± 281.82	892.05 ± 291.47	62.7	5379
2006	2.04 ± 0.49	244.97 ± 225.46	535.73 ± 231.88	119.9	5887
2007	1.30 ± 0.59	343.39 ± 226.25	16.60 ± 172.48	264.2	5002
2008	2.72 ± 1.06	363.41 ± 943.17	1025.86 ± 657.45	133.6	4752
2009	4.47 ± 0.73	393.04 ± 772.78	285.74 ± 461.75	87.8	7048
2010	3.45 ± 0.72	572.11 ± 1720.21	684.85 ± 837.14	165.7	9039
2011	4.64 ± 1.28	501.47 ± 2395.14	797.80 ± 1207.66	108.0	7989
2012	2.71 ± 0.52	129.31 ± 495.08	1384.52 ± 672.09	47.7	5651

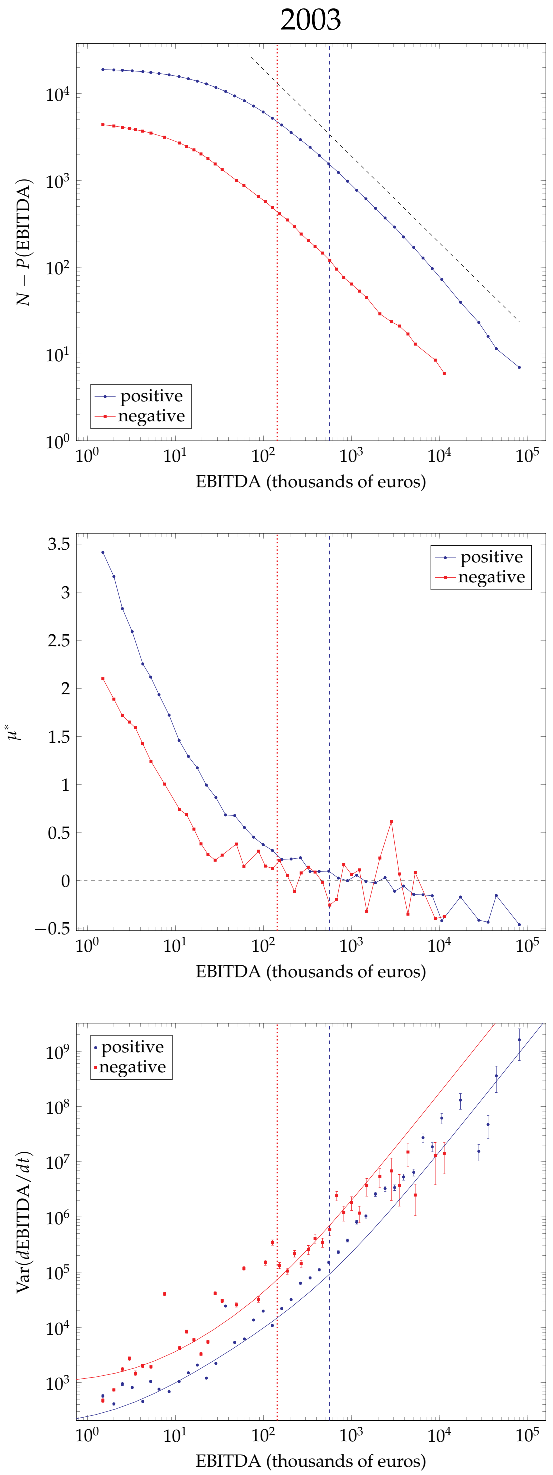

Figure 51: **Basque country 2003: Rank plot, chemical potential and variance.**
Positive EBITDA: 19408 firms.
T_1 = 0.15 ± 0.05, $T_{1/2}$= 81.64 ± 11.70, and T_0 =160.31 ± 140.66
Negative EBITDA: 4885 firms.
T_1 = 1.76 ± 0.51, $T_{1/2}$= 252.41 ± 76.00, and T_0 =944.56 ± 409.07.
Total active firms 24293, total created firms 4326, and total destroyed firms 1460

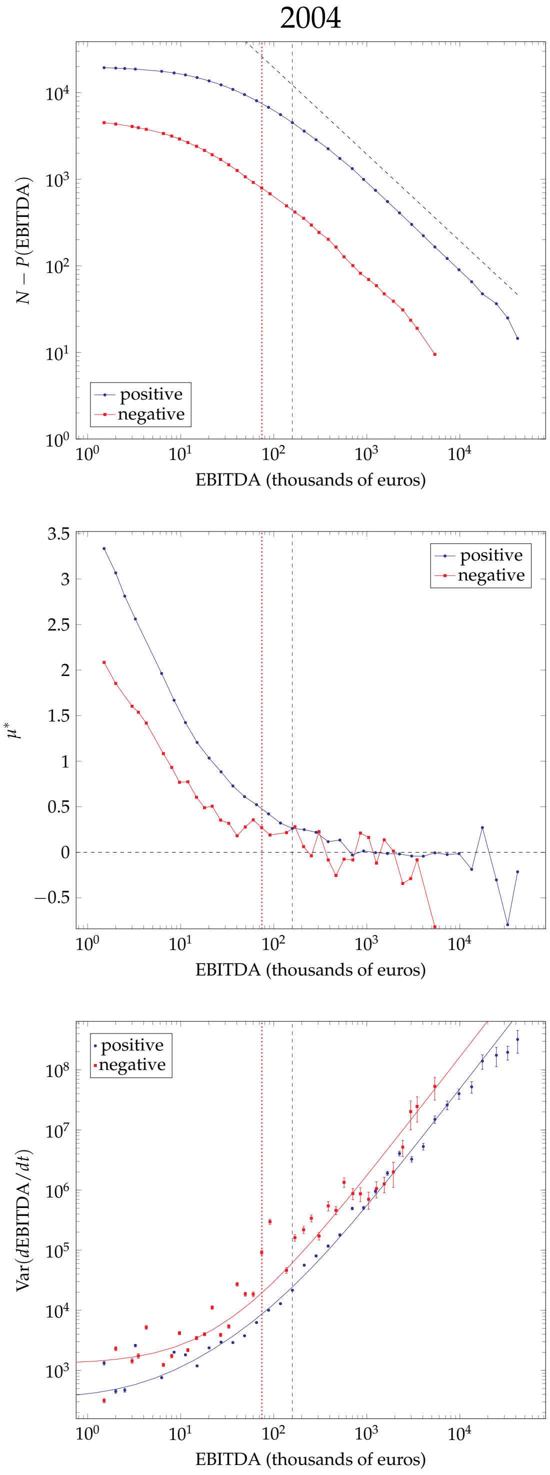

Figure 52: **Basque country 2004: Rank plot, chemical potential and variance.**
Positive EBITDA: 19878 firms.
$T_1 = 0.48 \pm 0.08$, $T_{1/2} = 75.53 \pm 10.10$, and $T_0 = 331.88 \pm 69.73$
Negative EBITDA: 5007 firms.
$T_1 = 1.63 \pm 0.49$, $T_{1/2} = 121.61 \pm 50.78$, and $T_0 = 1275.44 \pm 392.30$.
Total active firms 24885, total created firms 4249, and total destroyed firms 3688

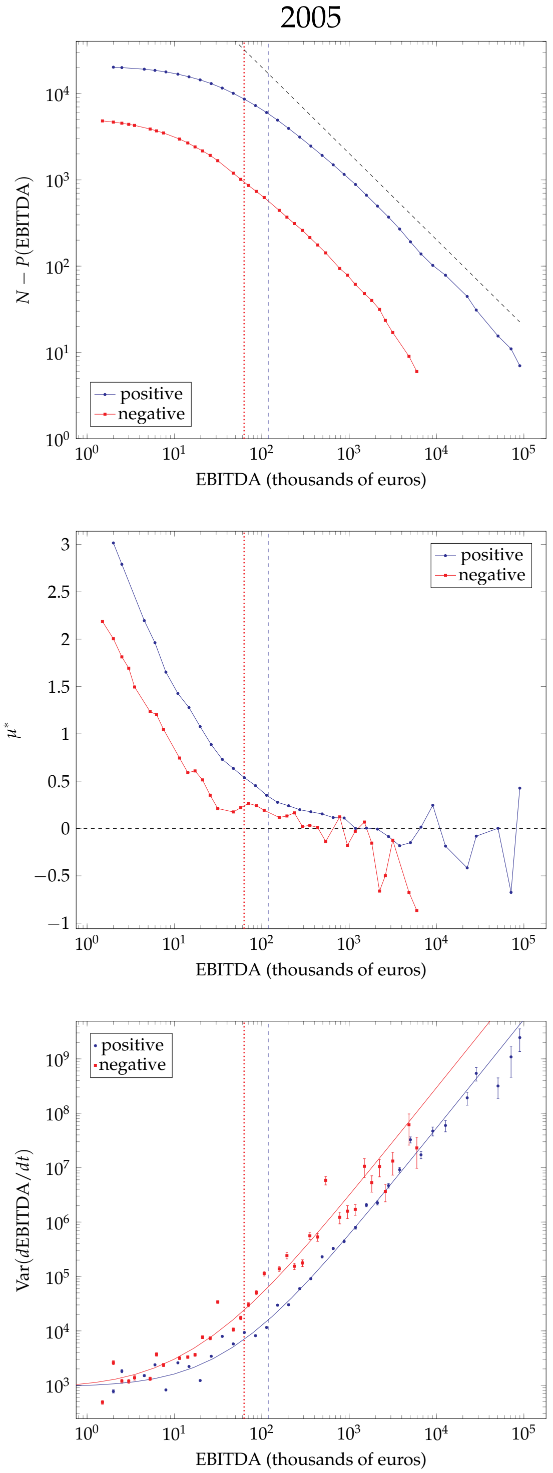

Figure 53: **Basque country 2005: Rank plot, chemical potential and variance.**
Positive EBITDA: 20989 firms.
$T_1 = 0.53 \pm 0.12$, $T_{1/2} = 62.88 \pm 18.46$, and $T_0 = 931.98 \pm 193.65$
Negative EBITDA: 5379 firms.
$T_1 = 2.94 \pm 0.73$, $T_{1/2} = 184.49 \pm 54.00$, and $T_0 = 892.05 \pm 291.47$.
Total active firms 26368, total created firms 4840, and total destroyed firms 2769

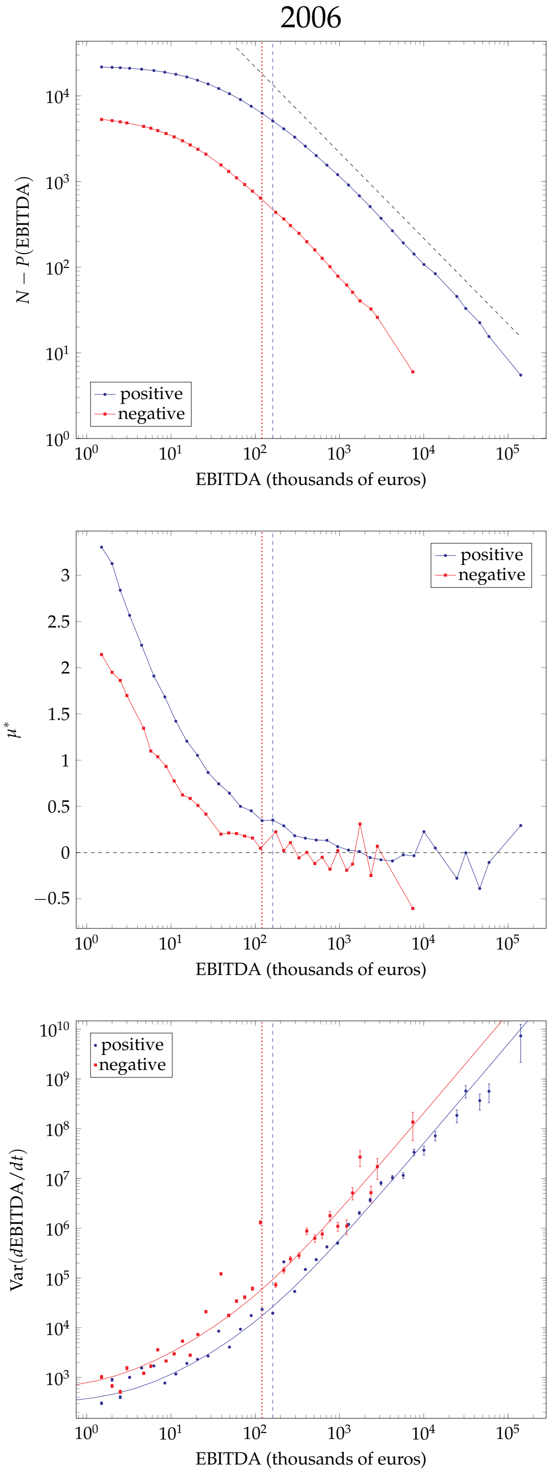

Figure 54: **Basque country 2006: Rank plot, chemical potential and variance.**
Positive EBITDA: 22313 firms.
$T_1 = 0.50 \pm 0.10$, $T_{1/2} = 81.12 \pm 13.05$, and $T_0 = 293.46 \pm 129.12$
Negative EBITDA: 5887 firms.
$T_1 = 2.04 \pm 0.49$, $T_{1/2} = 244.97 \pm 46.12$, and $T_0 = 535.73 \pm 231.88$.
Total active firms 28200, total created firms 4380, and total destroyed firms 2993

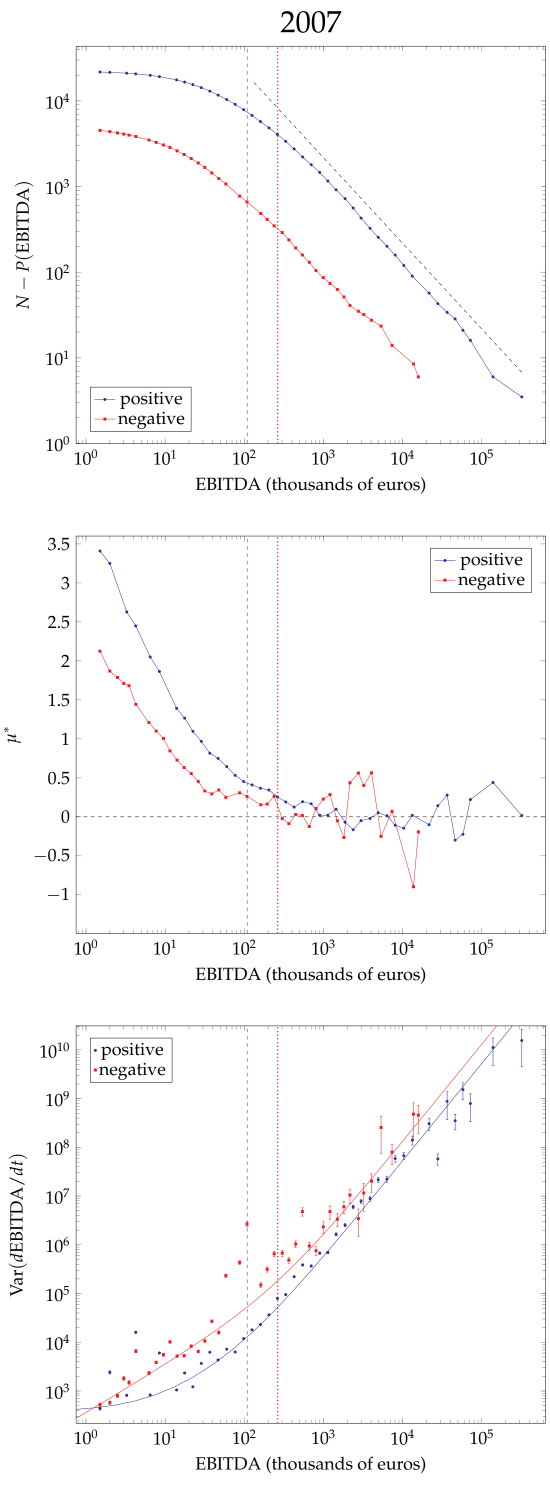

Figure 55: **Basque country 2007: Rank plot, chemical potential and variance**.
Positive EBITDA: 22219 firms.
T_1 = 0.52 ± 0.10, $T_{1/2}$= 57.02 ± 13.33, and T_0 =377.29 ± 199.63
Negative EBITDA: 5002 firms.
T_1 = 1.30 ± 0.59, $T_{1/2}$= 343.39 ± 46.23, and T_0 =16.60 ± 172.48.
Total active firms 27221, total created firms 1850, and total destroyed firms 5339

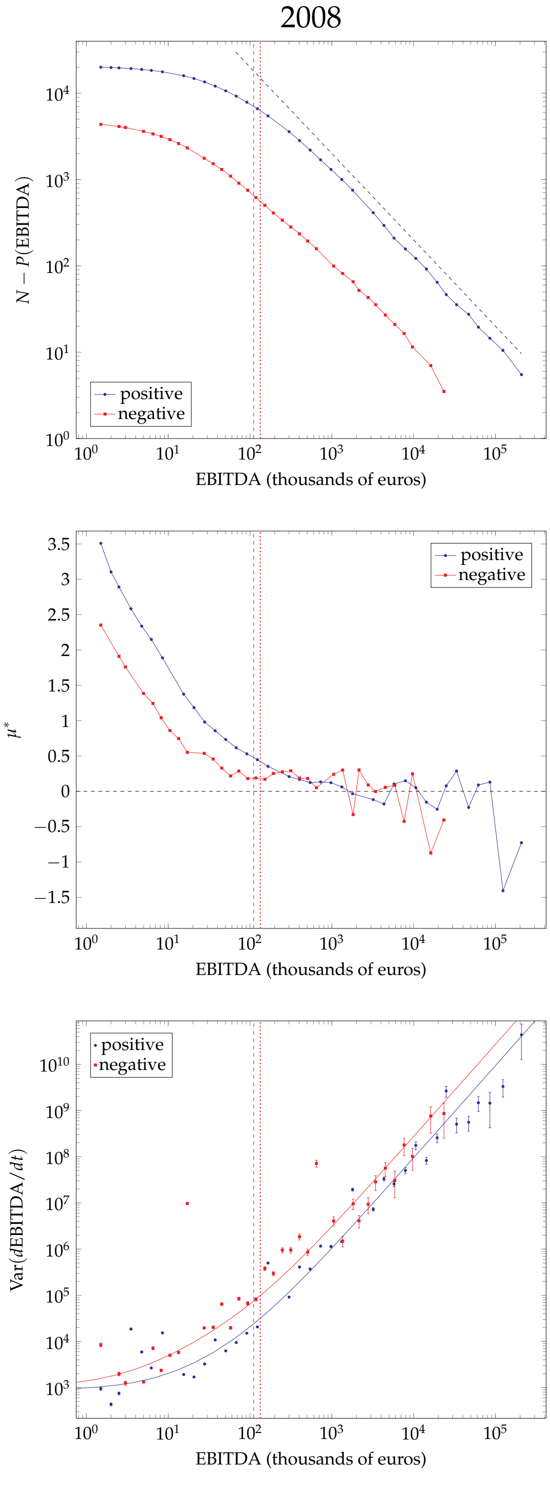

Figure 56: **Basque country 2008: Rank plot, chemical potential and variance**.
Positive EBITDA: 20441 firms.
$T_1 = 0.94 \pm 0.43$, $T_{1/2}$= 104.39 ± 50.47, and T_0 =882.63 ± 345.70
Negative EBITDA: 4752 firms.
$T_1 = 2.72 \pm 1.06$, $T_{1/2}$= 363.41 ± 126.87, and T_0 =1025.86 ± 657.45.
Total active firms 25193, total created firms 9169, and total destroyed firms 4008

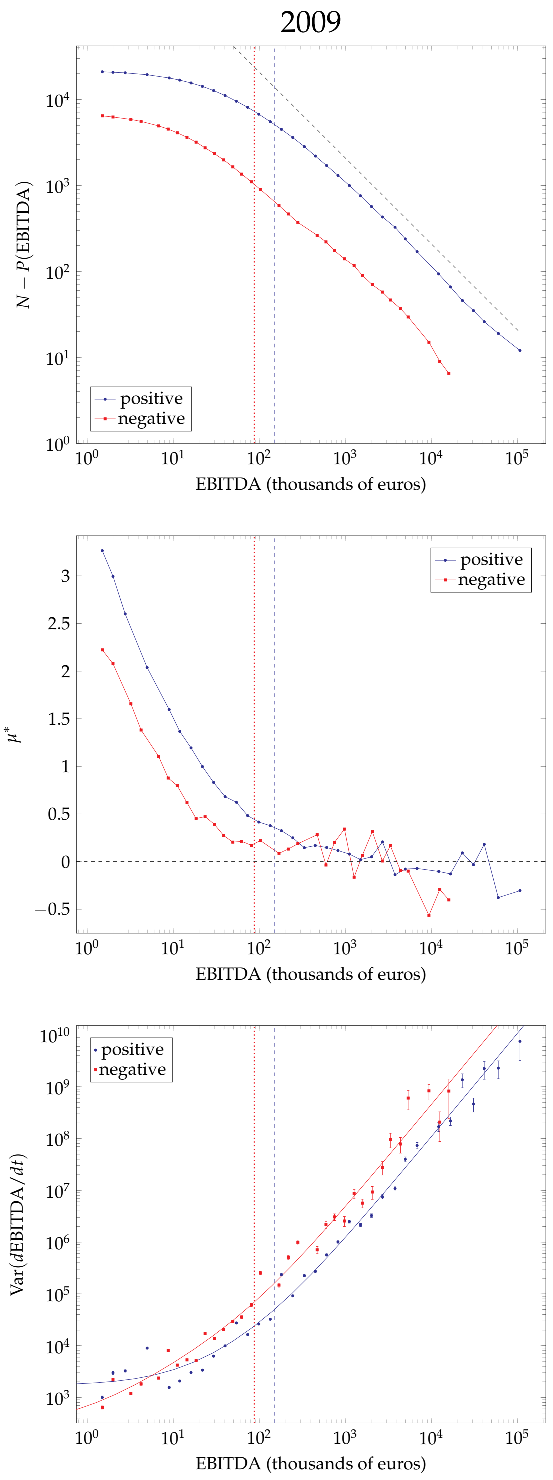

Figure 57: **Basque country 2009: Rank plot, chemical potential and variance.**
Positive EBITDA: 21588 firms.
$T_1 = 1.06 \pm 0.15$, $T_{1/2} = 159.64 \pm 53.31$, and $T_0 = 1712.80 \pm 596.21$
Negative EBITDA: 7048 firms.
$T_1 = 4.47 \pm 0.73$, $T_{1/2} = 393.04 \pm 110.20$, and $T_0 = 285.74 \pm 461.75$.
Total active firms 28636, total created firms 3851, and total destroyed firms 2860

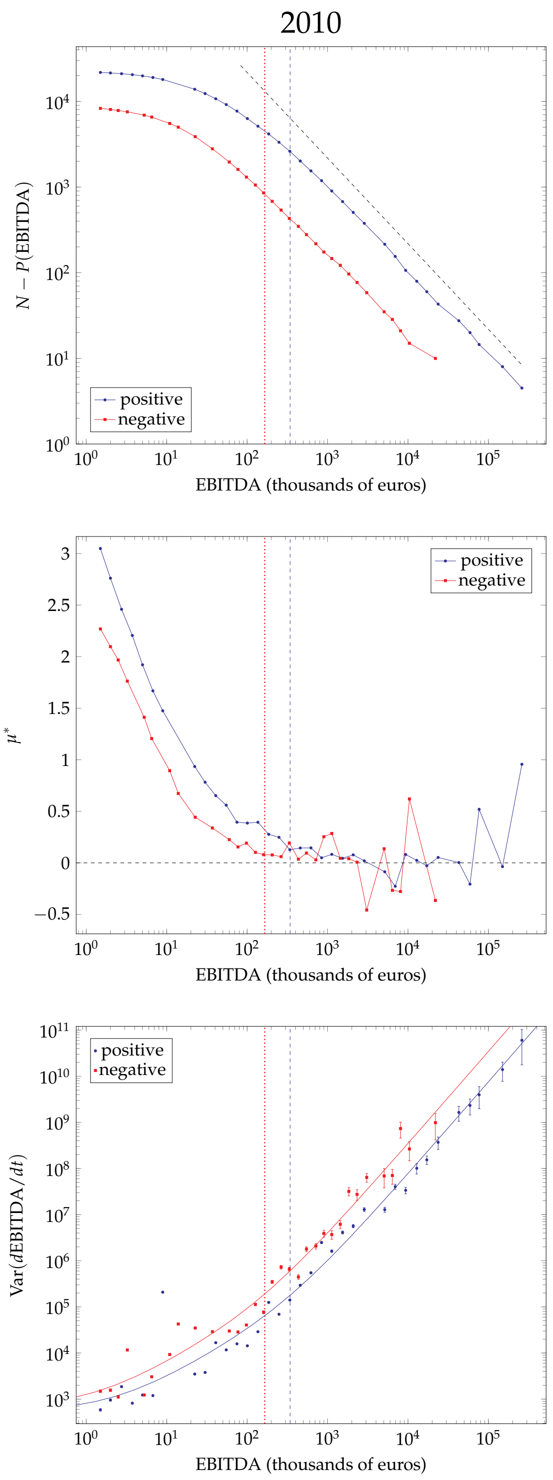

Figure 58: **Basque country 2010: Rank plot, chemical potential and variance.**
Positive EBITDA: 22587 firms.
T_1 = 0.76 ± 0.18, $T_{1/2}$= 259.13 ± 98.51, and T_0 =548.52 ± 571.50
Negative EBITDA: 9039 firms.
T_1 = 3.45 ± 0.72, $T_{1/2}$= 572.11 ± 194.04, and T_0 =684.85 ± 837.14.
Total active firms 31626, total created firms 3339, and total destroyed firms 3581

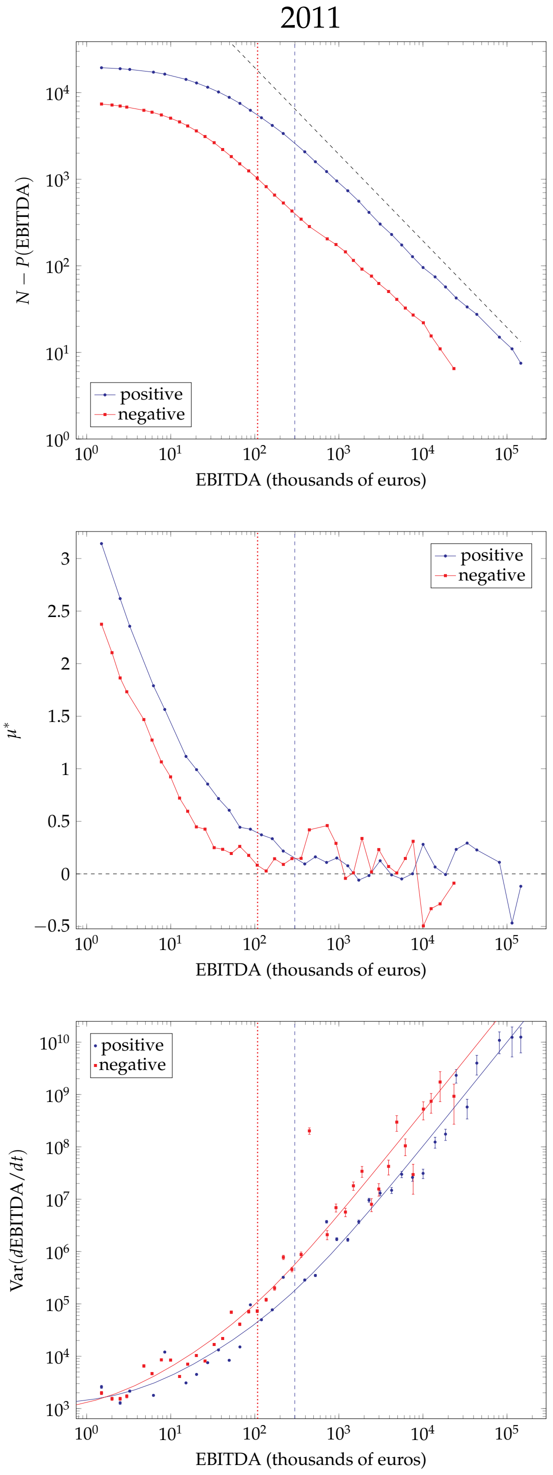

Figure 59: **Basque country 2011: Rank plot, chemical potential and variance.**
Positive EBITDA: 20060 firms.
$T_1 = 1.00 \pm 0.18$, $T_{1/2} = 299.45 \pm 91.85$, and $T_0 = 1145.89 \pm 757.37$
Negative EBITDA: 7989 firms.
$T_1 = 4.64 \pm 1.28$, $T_{1/2} = 501.47 \pm 245.22$, and $T_0 = 797.80 \pm 1207.66$.
Total active firms 28049, total created firms 1481, and total destroyed firms 4168

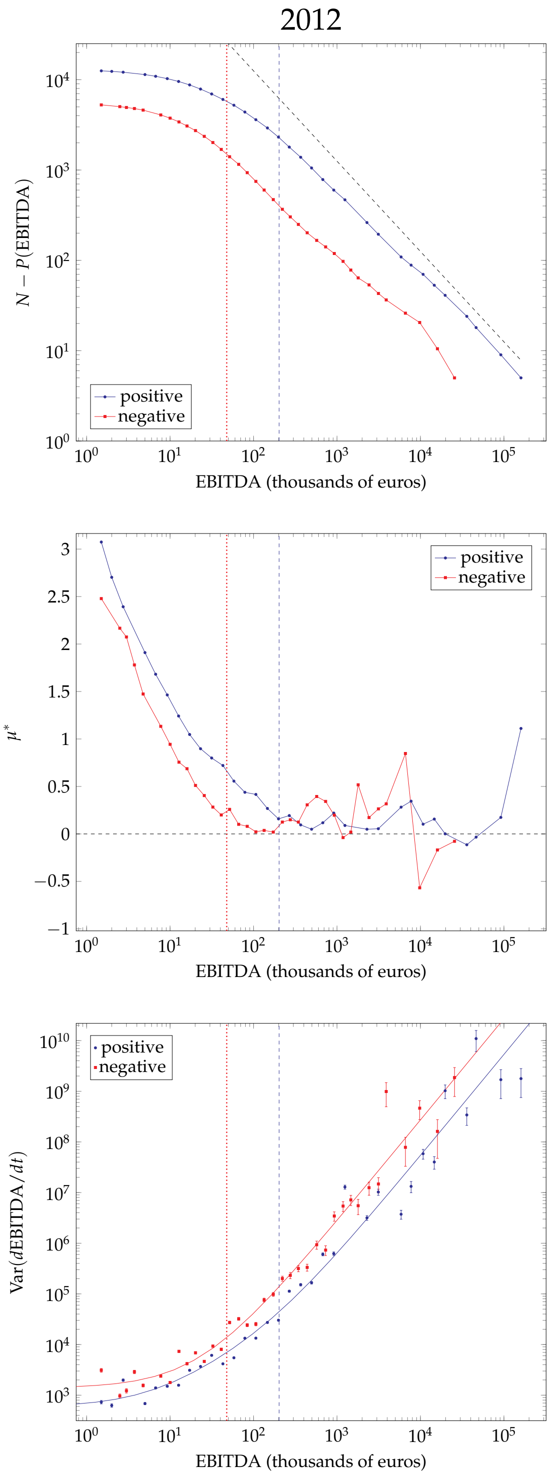

Figure 60: **Basque country 2012: Rank plot, chemical potential and variance**.
Positive EBITDA: 12961 firms.
$T_1 = 0.53 \pm 0.14$, $T_{1/2}$= 107.92 ± 51.95, and T_0 =587.59 ± 438.80
Negative EBITDA: 5651 firms.
$T_1 = 2.71 \pm 0.52$, $T_{1/2}$= 129.31 ± 80.43, and T_0 =1384.52 ± 672.09.
Total active firms 18612, total created firms 285, and total destroyed firms 13934

Canary islands

Tables of Temperatures

Positive EBITDA

Year	T_1	$T_{1/2}$	T_0	$T_{1/2}/T_1$	Num. Firms
2003	0.43 ± 0.08	174.33 ± 43.46	721.88 ± 366.43	405.1	13429
2004	0.47 ± 0.09	72.31 ± 39.58	2071.52 ± 702.03	154.3	14803
2005	0.42 ± 0.07	177.53 ± 40.99	731.02 ± 336.50	419.9	15948
2006	0.25 ± 0.09	177.38 ± 35.30	883.87 ± 324.63	704.5	16221
2007	0.51 ± 0.07	113.18 ± 34.83	1498.80 ± 506.68	223.4	15918
2008	0.71 ± 0.11	156.65 ± 48.25	2422.03 ± 669.92	221.9	13784
2009	0.57 ± 0.09	234.59 ± 48.96	518.09 ± 319.66	409.1	15869
2010	0.49 ± 0.09	202.86 ± 46.46	965.62 ± 375.45	414.3	15248
2011	0.55 ± 0.11	238.63 ± 55.84	829.66 ± 404.99	431.5	14625
2012	0.55 ± 0.07	72.99 ± 23.44	1262.61 ± 323.57	133.6	8495

Negative EBITDA

Year	T_1	$T_{1/2}$	T_0	$T_{1/2}/T_1$	Num. Firms
2003	1.94 ± 0.69	345.50 ± 761.26	0.09 ± 393.50	177.9	3207
2004	1.97 ± 0.44	266.48 ± 328.24	233.26 ± 258.96	135.6	3713
2005	2.11 ± 0.32	191.48 ± 240.75	1121.99 ± 370.79	90.8	4287
2006	1.07 ± 0.35	322.59 ± 369.10	1286.67 ± 493.09	301.7	4258
2007	1.54 ± 0.80	680.71 ± 1104.81	335.43 ± 505.28	440.8	4042
2008	2.75 ± 0.61	584.56 ± 1219.73	694.11 ± 762.06	212.9	3833
2009	2.10 ± 0.39	427.70 ± 606.10	110.60 ± 493.98	203.2	6463
2010	2.45 ± 0.37	148.93 ± 388.38	2126.10 ± 752.73	60.9	6806
2011	2.61 ± 0.58	202.43 ± 469.52	791.80 ± 486.88	77.5	6288
2012	1.92 ± 0.62	157.43 ± 255.60	975.86 ± 491.90	81.9	3573

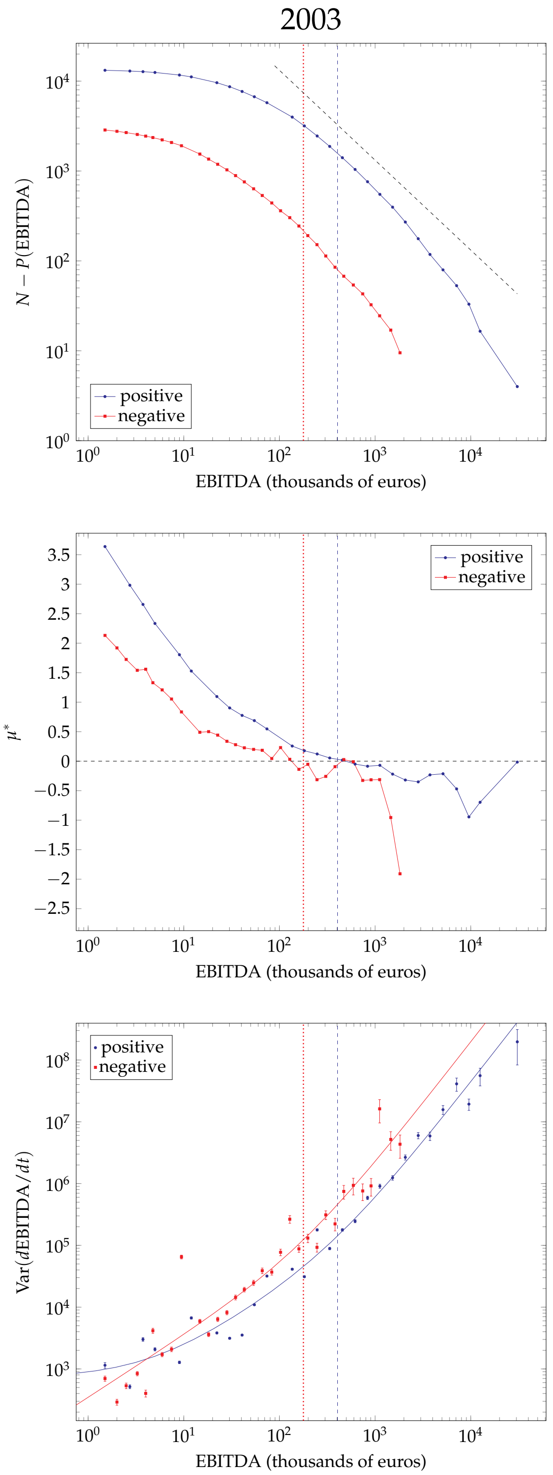

Figure 61: **Canary islands 2003: Rank plot, chemical potential and variance**.
Positive EBITDA: 13429 firms.
T_1 = 0.43 ± 0.08, $T_{1/2}$= 174.33 ± 43.46, and T_0 =721.88 ± 366.43
Negative EBITDA: 3207 firms.
T_1 = 1.94 ± 0.69, $T_{1/2}$= 345.50 ± 109.03, and T_0 =0.09 ± 393.50.
Total active firms 16636, total created firms 4650, and total destroyed firms 1584

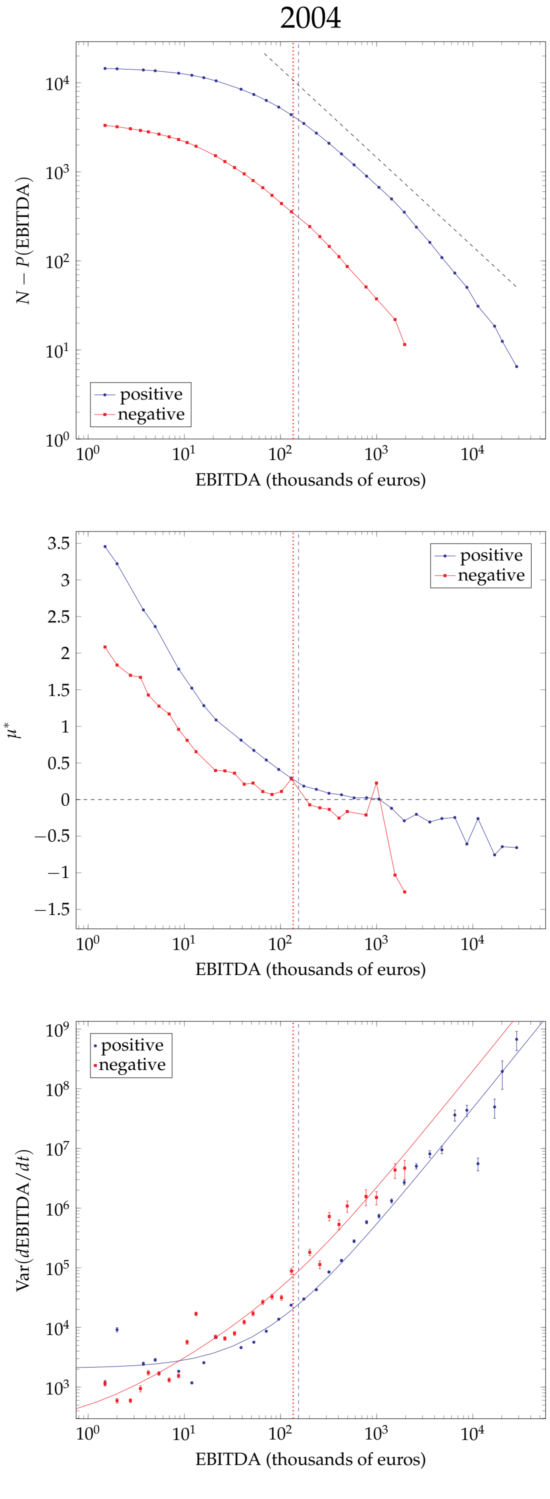

Figure 62: **Canary islands 2004: Rank plot, chemical potential and variance**.
Positive EBITDA: 14803 firms.
T_1 = 0.47 ± 0.09, $T_{1/2}$= 72.31 ± 39.58, and T_0 =2071.52 ± 702.03
Negative EBITDA: 3713 firms.
T_1 = 1.97 ± 0.44, $T_{1/2}$= 266.48 ± 60.15, and T_0 =233.26 ± 258.96.
Total active firms 18516, total created firms 4574, and total destroyed firms 2690

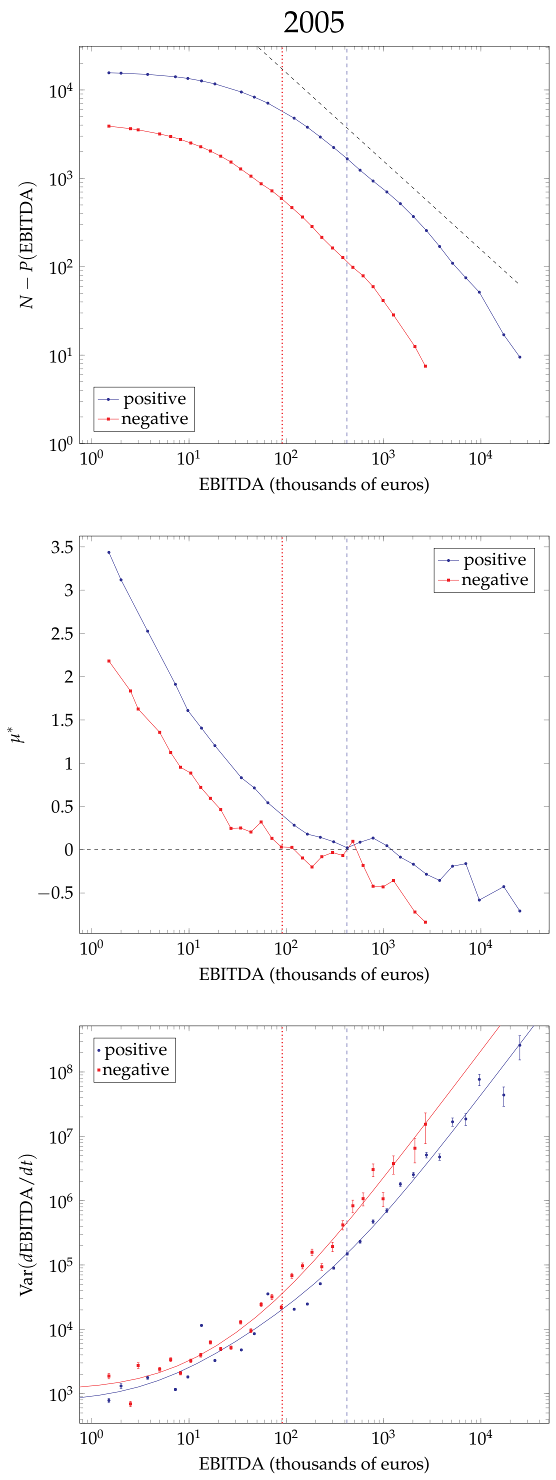

Figure 63: **Canary islands 2005: Rank plot, chemical potential and variance**.
Positive EBITDA: 15948 firms.
T_1 = 0.42 ± 0.07, $T_{1/2}$= 177.53 ± 40.99, and T_0 =731.02 ± 336.50
Negative EBITDA: 4287 firms.
T_1 = 2.11 ± 0.32, $T_{1/2}$= 191.48 ± 48.31, and T_0 =1121.99 ± 370.79.
Total active firms 20235, total created firms 3825, and total destroyed firms 2937

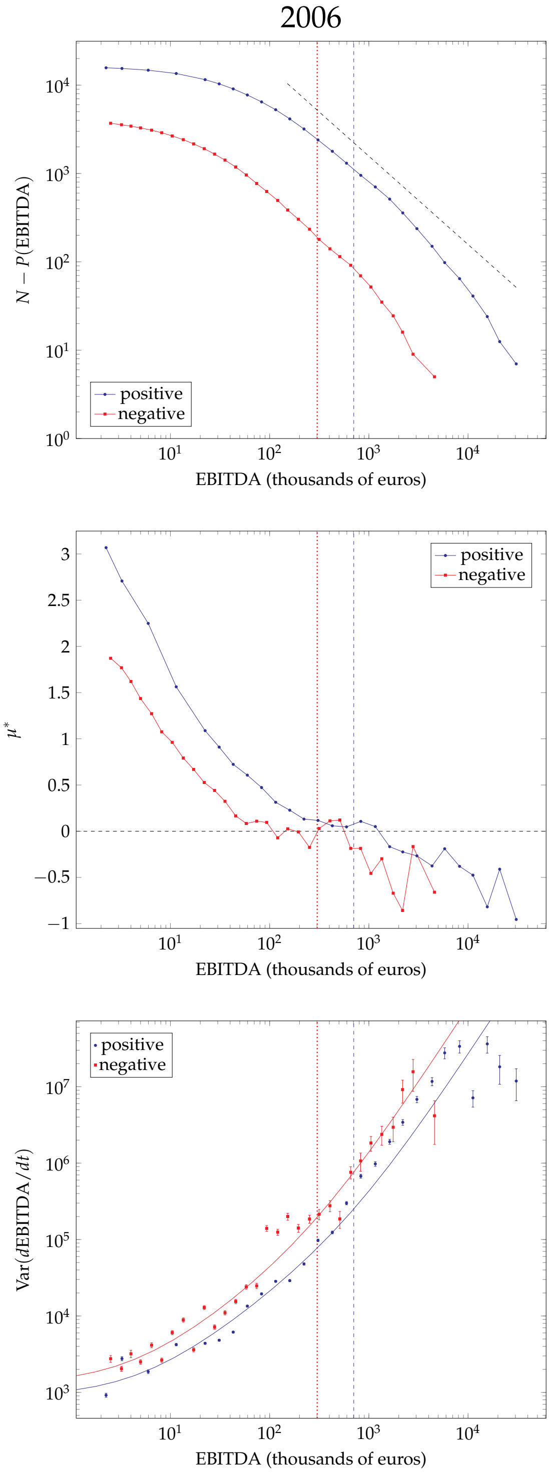

Figure 64: **Canary islands 2006: Rank plot, chemical potential and variance.**
Positive EBITDA: 16221 firms.
$T_1 = 0.25 \pm 0.09$, $T_{1/2} = 177.38 \pm 35.30$, and $T_0 = 883.87 \pm 324.63$
Negative EBITDA: 4258 firms.
$T_1 = 1.07 \pm 0.35$, $T_{1/2} = 322.59 \pm 65.35$, and $T_0 = 1286.67 \pm 493.09$.
Total active firms 20479, total created firms 3826, and total destroyed firms 3608

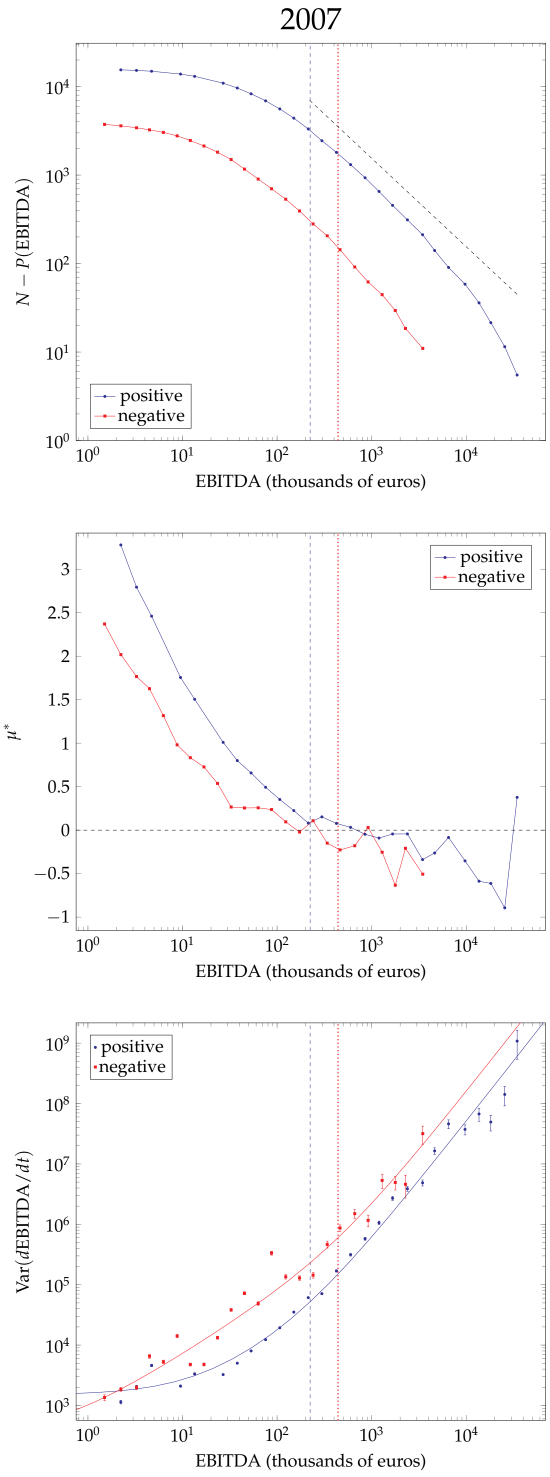

Figure 65: **Canary islands 2007: Rank plot, chemical potential and variance.**
Positive EBITDA: 15918 firms.
T_1 = 0.51 ± 0.07, $T_{1/2}$= 113.18 ± 34.83, and T_0 =1498.80 ± 506.68
Negative EBITDA: 4042 firms.
T_1 = 1.54 ± 0.80, $T_{1/2}$= 680.71 ± 141.88, and T_0 =335.43 ± 505.28.
Total active firms 19960, total created firms 1441, and total destroyed firms 4351

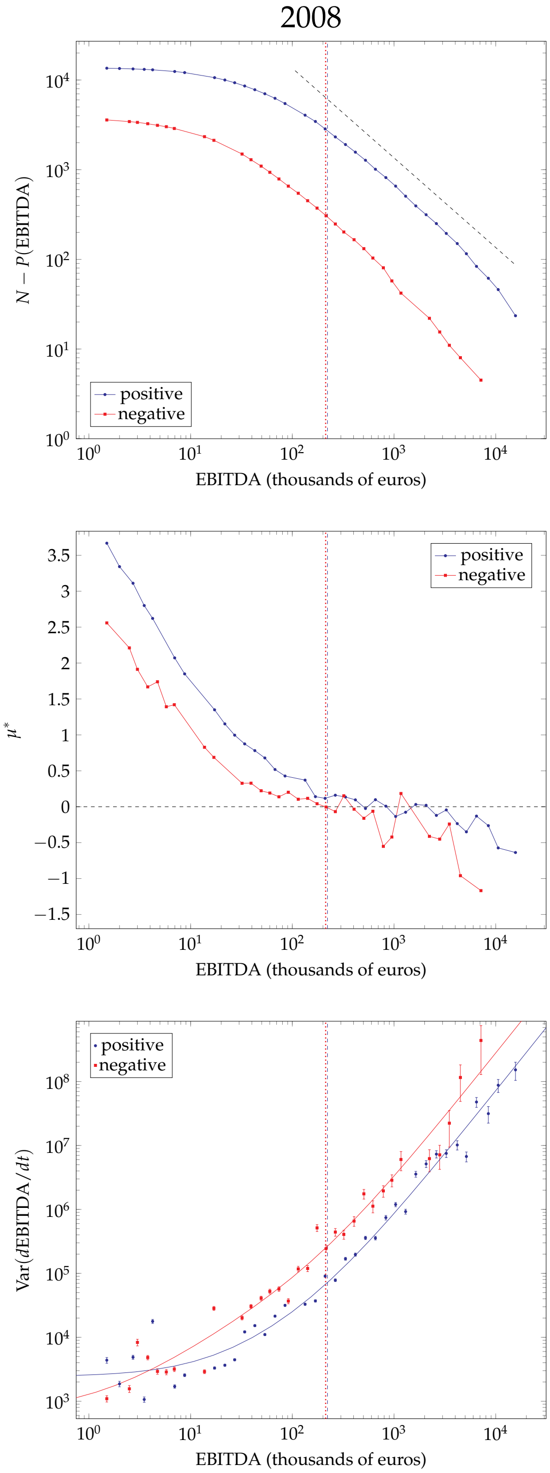

Figure 66: **Canary islands 2008: Rank plot, chemical potential and variance.**
Positive EBITDA: 13784 firms.
$T_1 = 0.71 \pm 0.11$, $T_{1/2}$= 156.65 ± 48.25, and T_0 =2422.03 ± 669.92
Negative EBITDA: 3833 firms.
$T_1 = 2.75 \pm 0.61$, $T_{1/2}$= 584.56 ± 152.17, and T_0 =694.11 ± 762.06.
Total active firms 17617, total created firms 7695, and total destroyed firms 3858

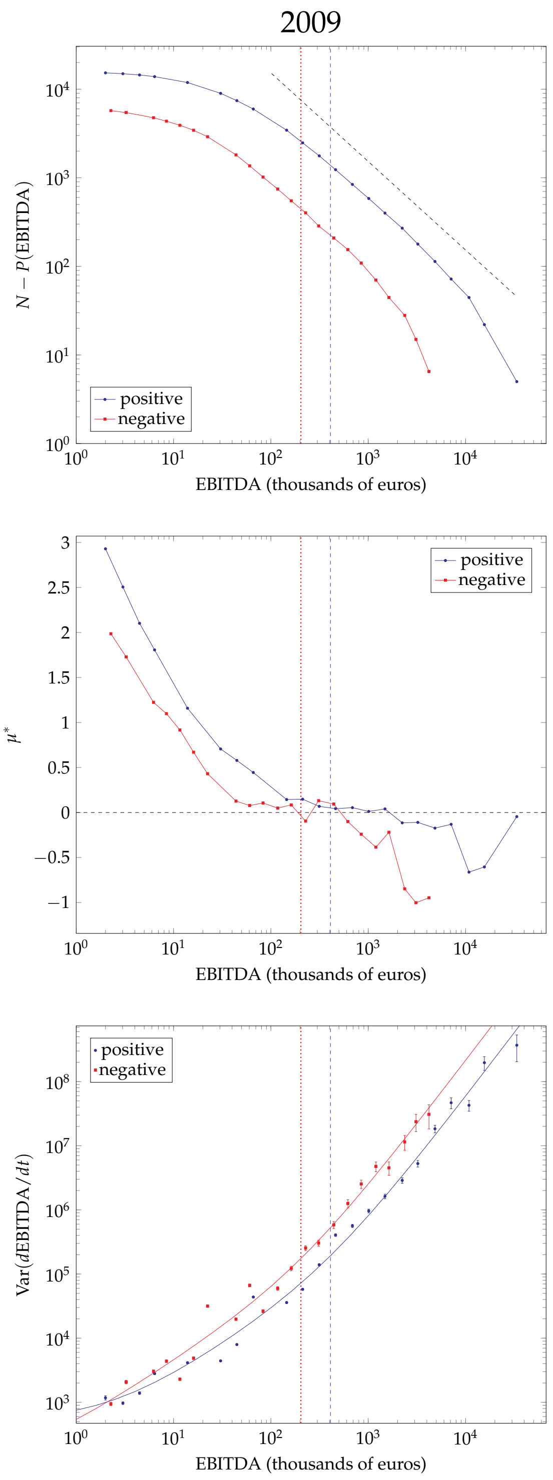

Figure 67: **Canary islands 2009: Rank plot, chemical potential and variance**.
Positive EBITDA: 15869 firms.
T_1 = 0.57 ± 0.09, $T_{1/2}$= 234.59 ± 48.96, and T_0 =518.09 ± 319.66
Negative EBITDA: 6463 firms.
T_1 = 2.10 ± 0.39, $T_{1/2}$= 427.70 ± 92.80, and T_0 =110.60 ± 493.98.
Total active firms 22332, total created firms 3091, and total destroyed firms 2868

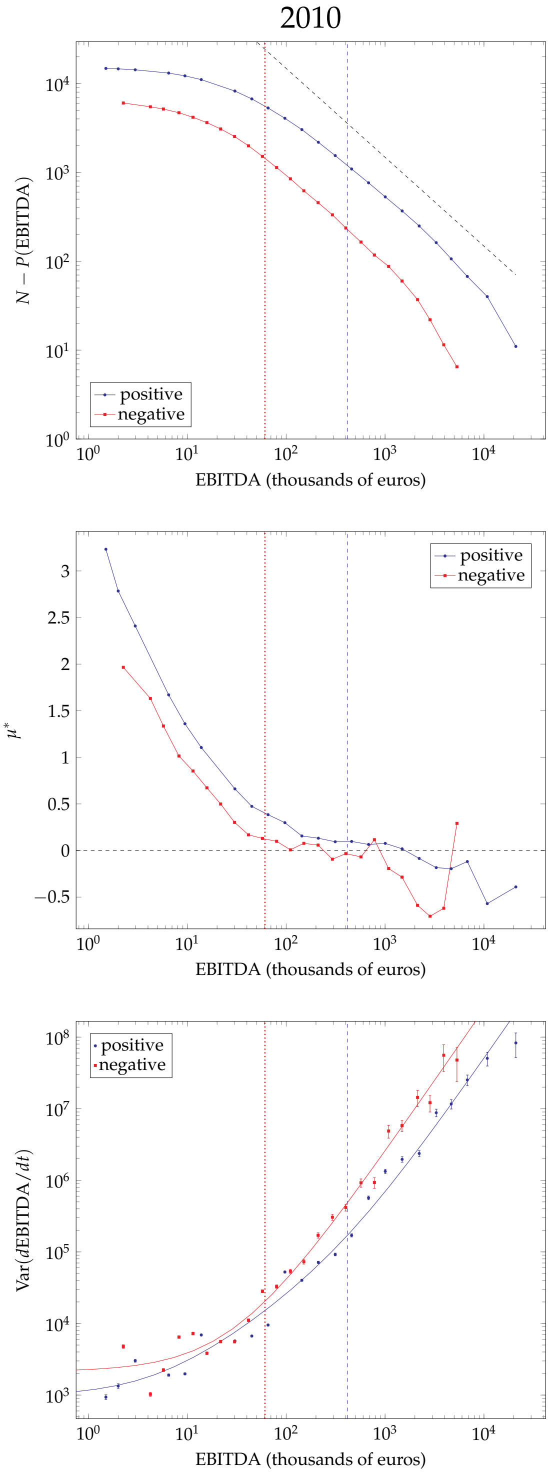

Figure 68: **Canary islands 2010: Rank plot, chemical potential and variance.**
Positive EBITDA: 15248 firms.
T_1 = 0.49 ± 0.09, $T_{1/2}$= 202.86 ± 46.46, and T_0 =965.62 ± 375.45
Negative EBITDA: 6806 firms.
T_1 = 2.45 ± 0.37, $T_{1/2}$= 148.93 ± 67.75, and T_0 =2126.10 ± 752.73.
Total active firms 22054, total created firms 2384, and total destroyed firms 3359

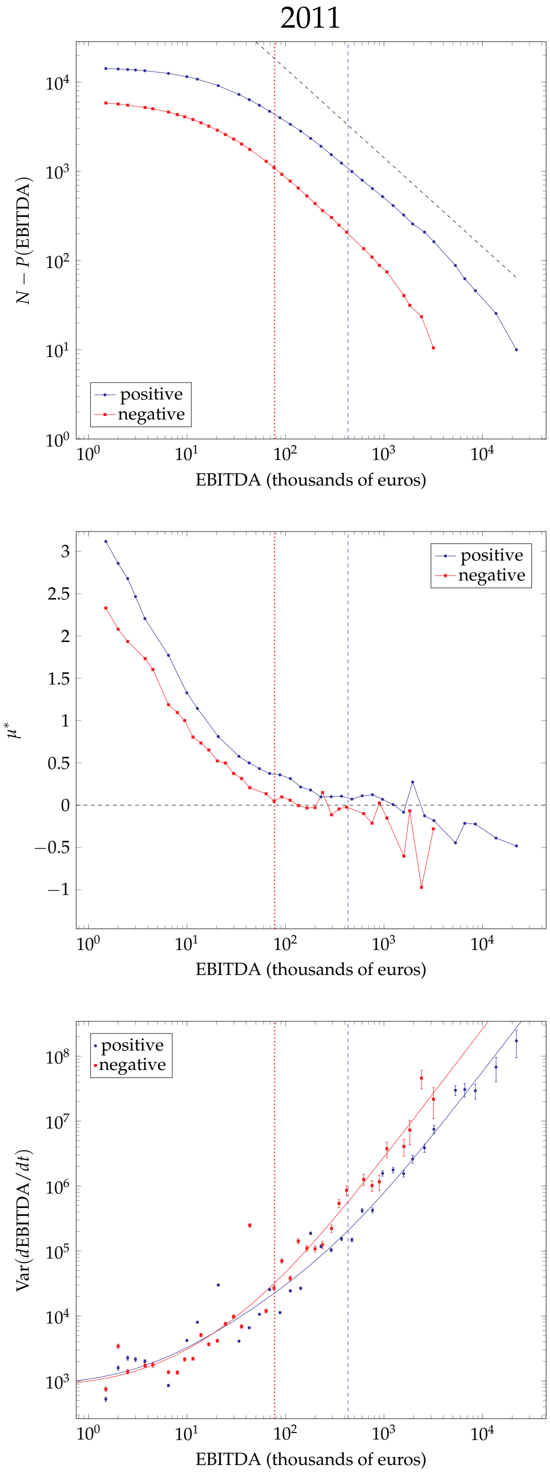

Figure 69: **Canary islands 2011: Rank plot, chemical potential and variance**.
Positive EBITDA: 14625 firms.
T_1 = 0.55 ± 0.11, $T_{1/2}$= 238.63 ± 55.84, and T_0 =829.66 ± 404.99
Negative EBITDA: 6288 firms.
T_1 = 2.61 ± 0.58, $T_{1/2}$= 202.43 ± 77.47, and T_0 =791.80 ± 486.88.
Total active firms 20913, total created firms 1107, and total destroyed firms 3549

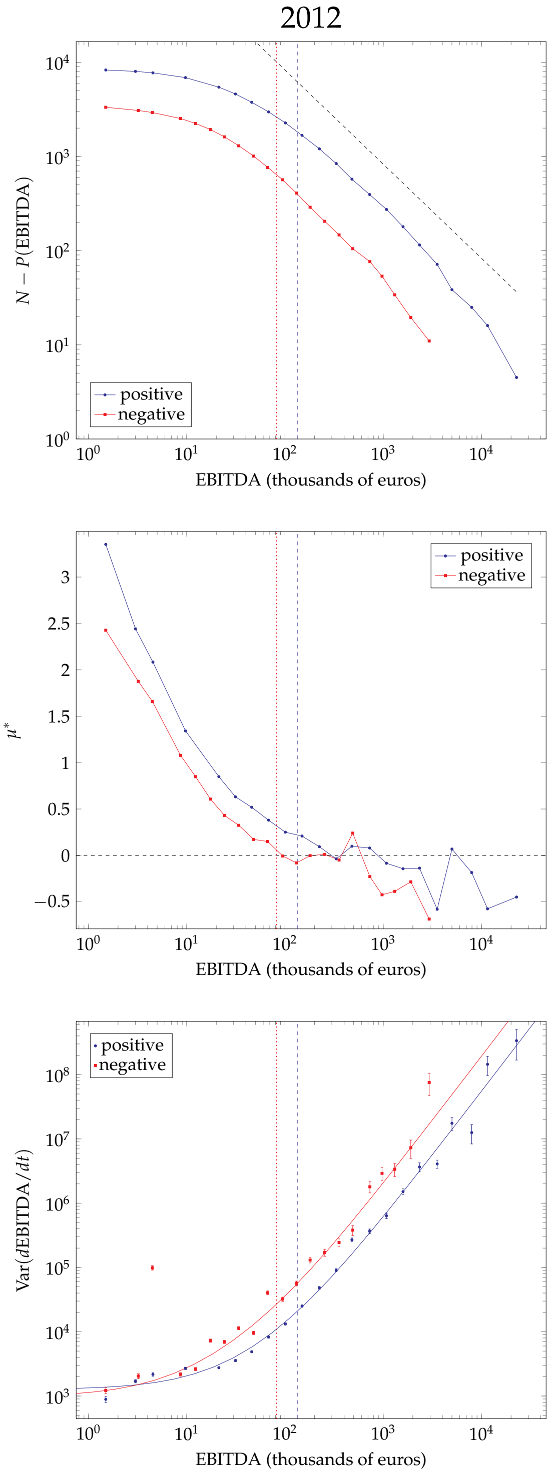

Figure 70: **Canary islands 2012: Rank plot, chemical potential and variance.**
Positive EBITDA: 8495 firms.
$T_1 = 0.55 \pm 0.07$, $T_{1/2}$= 72.99 ± 23.44, and T_0 =1262.61 ± 323.57
Negative EBITDA: 3573 firms.
$T_1 = 1.92 \pm 0.62$, $T_{1/2}$= 157.43 ± 50.40, and T_0 =975.86 ± 491.90.
Total active firms 12068, total created firms 229, and total destroyed firms 10080

Cantabria

Tables of Temperatures

Positive EBITDA

Year	T_1	$T_{1/2}$	T_0	$T_{1/2}/T_1$	Num. Firms
2003	0.33 ± 0.08	41.88 ± 9.49	216.80 ± 121.36	127.3	3553
2004	0.28 ± 0.09	92.41 ± 41.01	474.94 ± 317.33	331.3	3475
2005	0.42 ± 0.11	23.20 ± 28.63	1132.77 ± 458.60	55.5	3536
2006	0.32 ± 0.07	51.34 ± 21.86	510.36 ± 298.15	158.6	4011
2007	0.33 ± 0.09	86.44 ± 38.73	419.42 ± 321.35	260.0	4073
2008	0.55 ± 0.18	99.11 ± 90.14	2546.79 ± 1571.95	180.1	3769
2009	0.56 ± 0.10	90.33 ± 31.57	937.77 ± 346.81	161.4	3937
2010	0.64 ± 0.11	35.76 ± 11.89	376.68 ± 138.91	55.7	4106
2011	0.86 ± 0.20	59.86 ± 50.05	1549.23 ± 713.91	69.5	3374
2012	0.59 ± 0.22	75.46 ± 36.86	218.10 ± 227.09	127.9	791

Negative EBITDA

Year	T_1	$T_{1/2}$	T_0	$T_{1/2}/T_1$	Num. Firms
2003	2.41 ± 0.78	151.23 ± 210.89	540.79 ± 292.93	62.6	675
2004	2.45 ± 0.82	116.10 ± 937.12	2373.94 ± 1344.72	47.3	647
2005	1.38 ± 0.74	49.91 ± 246.79	1589.95 ± 432.50	36.2	653
2006	1.70 ± 0.44	96.08 ± 264.65	935.49 ± 396.49	56.5	748
2007	2.66 ± 1.27	240.10 ± 908.41	0.00 ± 369.60	90.2	691
2008	2.89 ± 2.93	184.48 ± 4830.26	3488.32 ± 4165.15	63.7	659
2009	3.68 ± 2.56	89.37 ± 1429.48	4217.63 ± 1477.89	24.3	1108
2010	2.05 ± 0.90	189.12 ± 220.29	6.79 ± 204.56	92.4	1441
2011	4.66 ± 1.09	205.97 ± 790.94	1294.66 ± 666.53	44.2	1299
2012	1.05 ± 1.05	92.85 ± 997.56	652.20 ± 1333.54	88.5	414

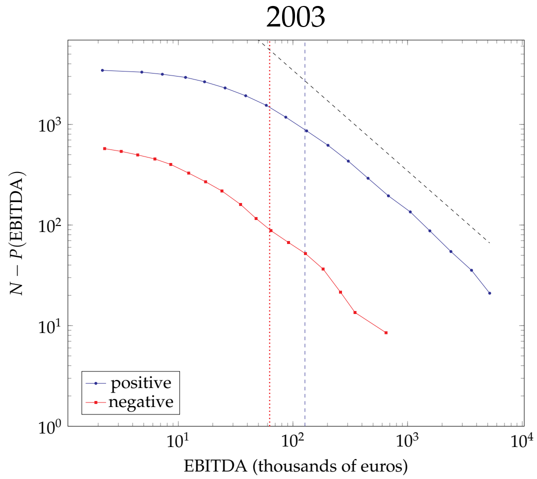

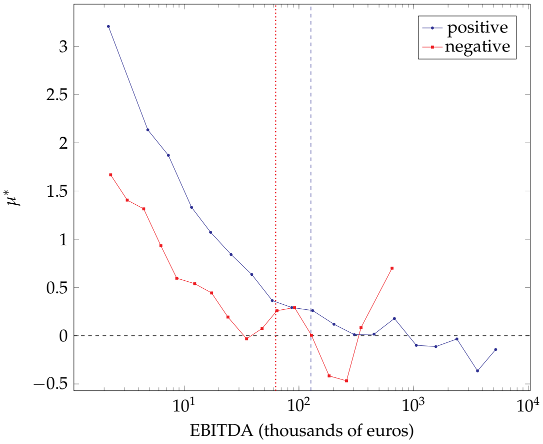

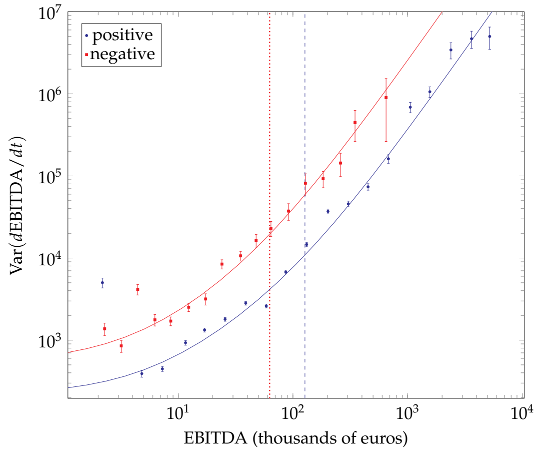

Figure 71: **Cantabria 2003: Rank plot, chemical potential and variance.**
Positive EBITDA: 3553 firms.
T_1 = 0.33 ± 0.08, $T_{1/2}$= 41.88 ± 9.49, and T_0 =216.80 ± 121.36
Negative EBITDA: 675 firms.
T_1 = 2.41 ± 0.78, $T_{1/2}$= 151.23 ± 43.99, and T_0 =540.79 ± 292.93.
Total active firms 4228, total created firms 811, and total destroyed firms 298

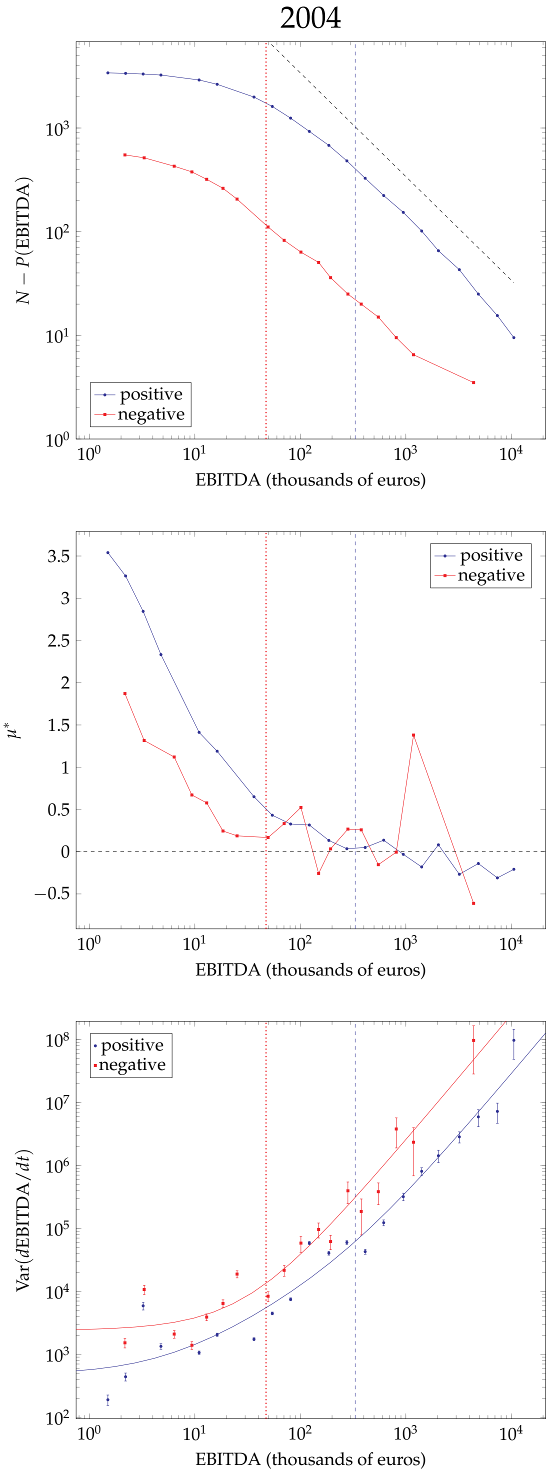

Figure 72: **Cantabria 2004: Rank plot, chemical potential and variance.**
Positive EBITDA: 3475 firms.
T_1 = 0.28 ± 0.09, $T_{1/2}$= 92.41 ± 41.01, and T_0 =474.94 ± 317.33
Negative EBITDA: 647 firms.
T_1 = 2.45 ± 0.82, $T_{1/2}$= 116.10 ± 126.29, and T_0 =2373.94 ± 1344.72.
Total active firms 4122, total created firms 777, and total destroyed firms 935

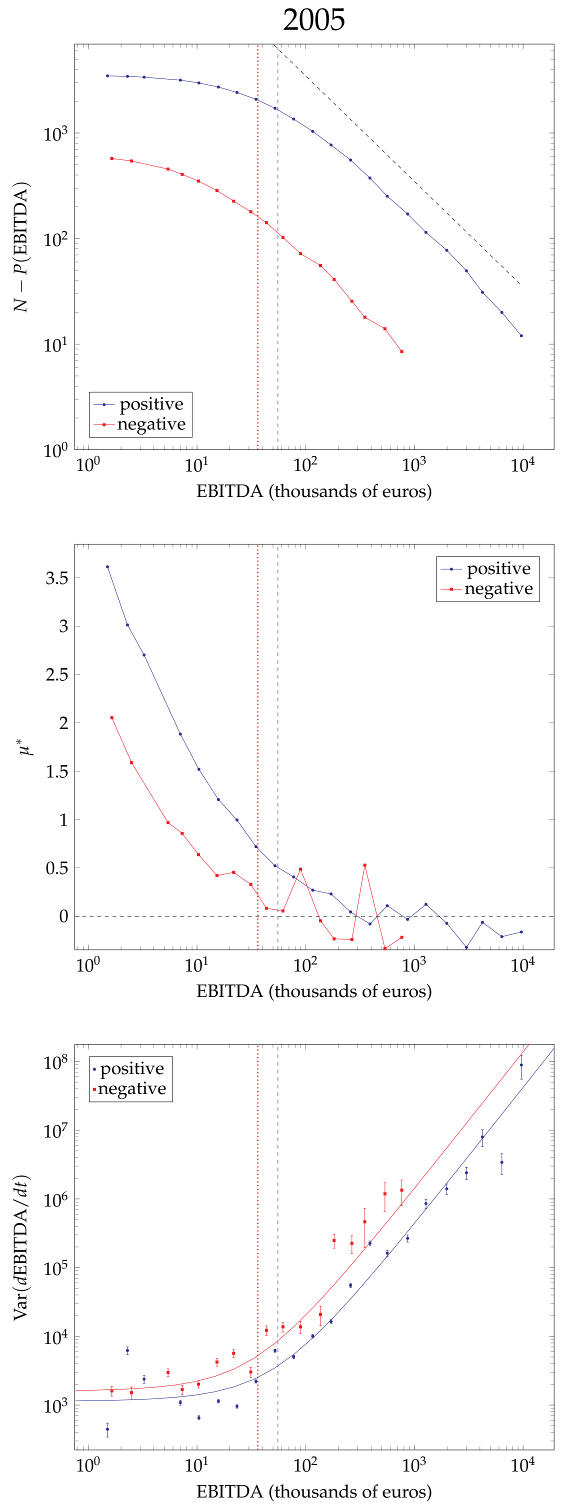

Figure 73: **Cantabria 2005: Rank plot, chemical potential and variance.**
Positive EBITDA: 3536 firms.
$T_1 = 0.42 \pm 0.11$, $T_{1/2} = 23.20 \pm 28.63$, and $T_0 = 1132.77 \pm 458.60$
Negative EBITDA: 653 firms.
$T_1 = 1.38 \pm 0.74$, $T_{1/2} = 49.91 \pm 49.16$, and $T_0 = 1589.95 \pm 432.50$.
Total active firms 4189, total created firms 1076, and total destroyed firms 692

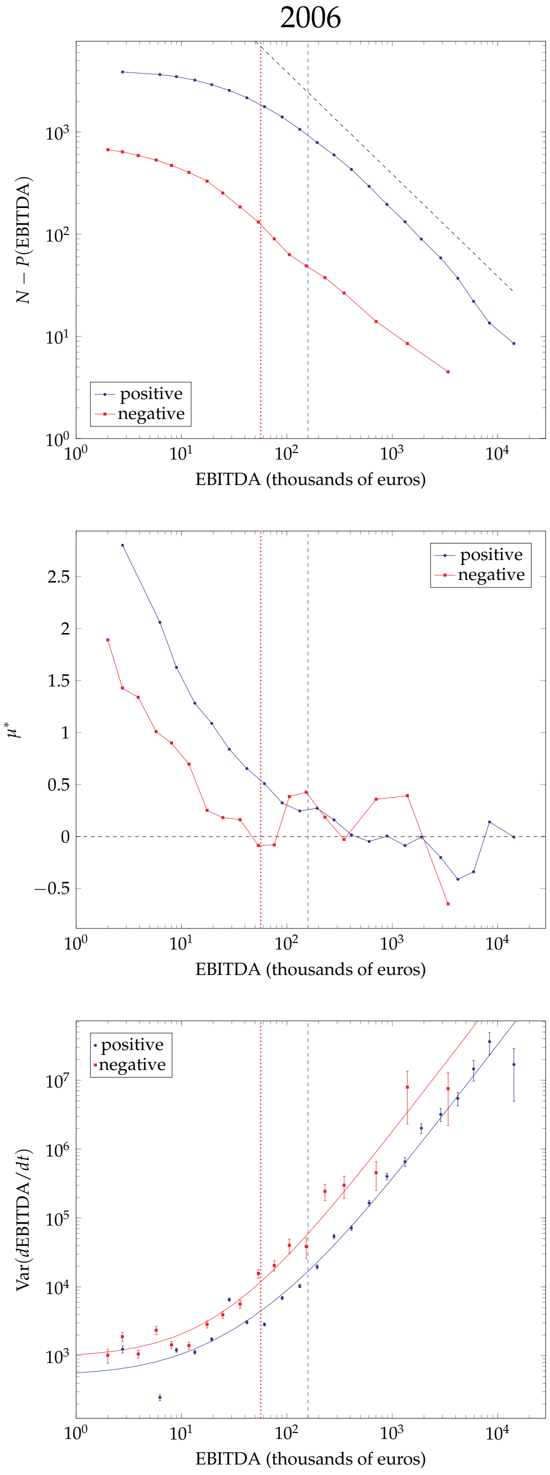

Figure 74: **Cantabria 2006: Rank plot, chemical potential and variance.**
Positive EBITDA: 4011 firms.
$T_1 = 0.32 \pm 0.07$, $T_{1/2}$= 51.34 ± 21.86, and T_0 =510.36 ± 298.15
Negative EBITDA: 748 firms.
$T_1 = 1.70 \pm 0.44$, $T_{1/2}$= 96.08 ± 51.65, and T_0 =935.49 ± 396.49.
Total active firms 4759, total created firms 1025, and total destroyed firms 492

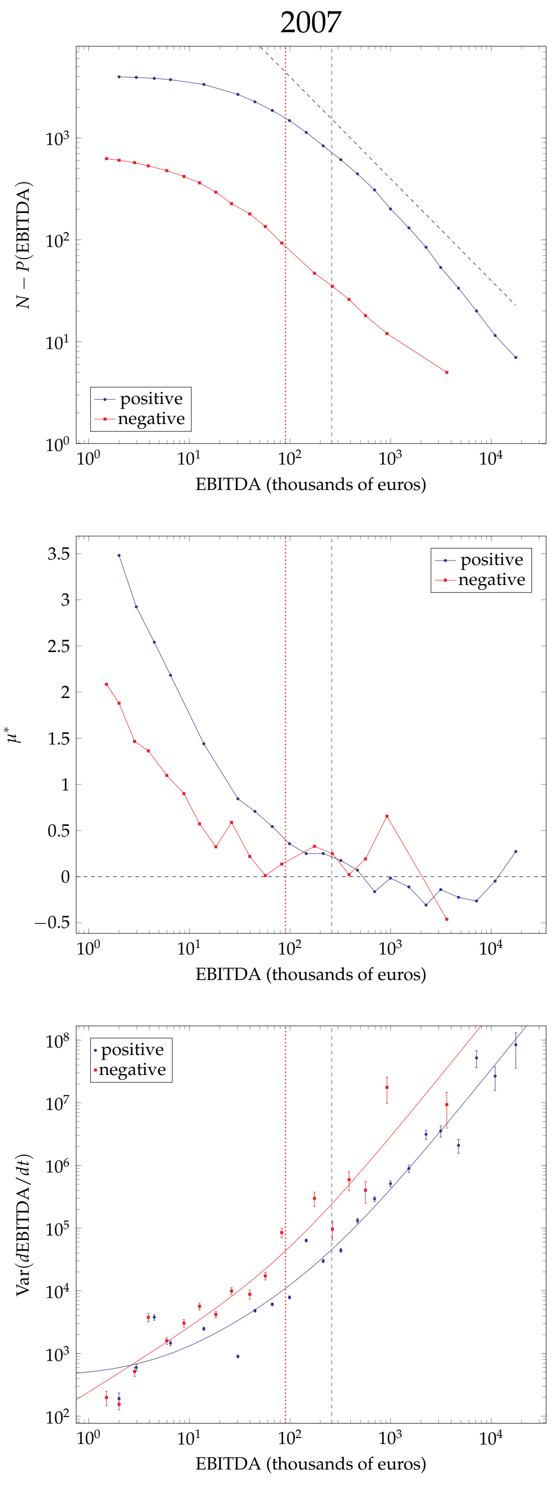

Figure 75: **Cantabria 2007: Rank plot, chemical potential and variance.**
Positive EBITDA: 4073 firms.
T_1 = 0.33 ± 0.09, $T_{1/2}$= 86.44 ± 38.73, and T_0 =419.42 ± 321.35
Negative EBITDA: 691 firms.
T_1 = 2.66 ± 1.27, $T_{1/2}$= 240.10 ± 123.54, and T_0 =0.00 ± 369.60.
Total active firms 4764, total created firms 411, and total destroyed firms 1047

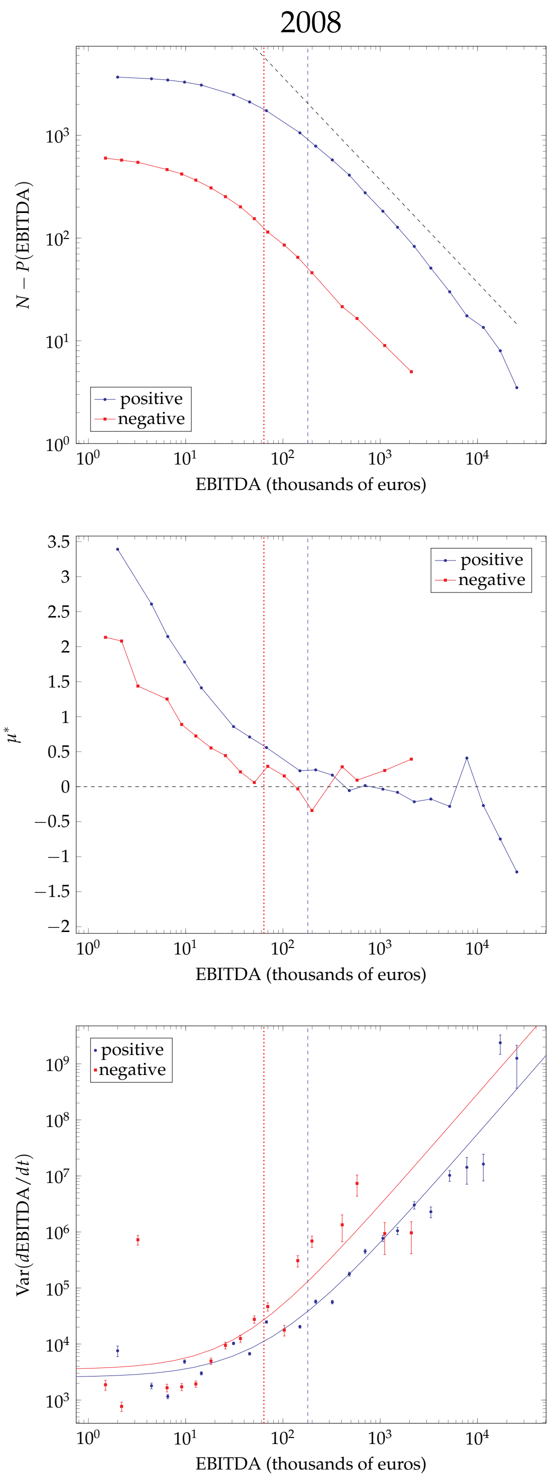

Figure 76: **Cantabria 2008: Rank plot, chemical potential and variance.**
Positive EBITDA: 3769 firms.
$T_1 = 0.55 \pm 0.18$, $T_{1/2} = 99.11 \pm 90.14$, and $T_0 = 2546.79 \pm 1571.95$
Negative EBITDA: 659 firms.
$T_1 = 2.89 \pm 2.93$, $T_{1/2} = 184.48 \pm 402.68$, and $T_0 = 3488.32 \pm 4165.15$.
Total active firms 4428, total created firms 2159, and total destroyed firms 756

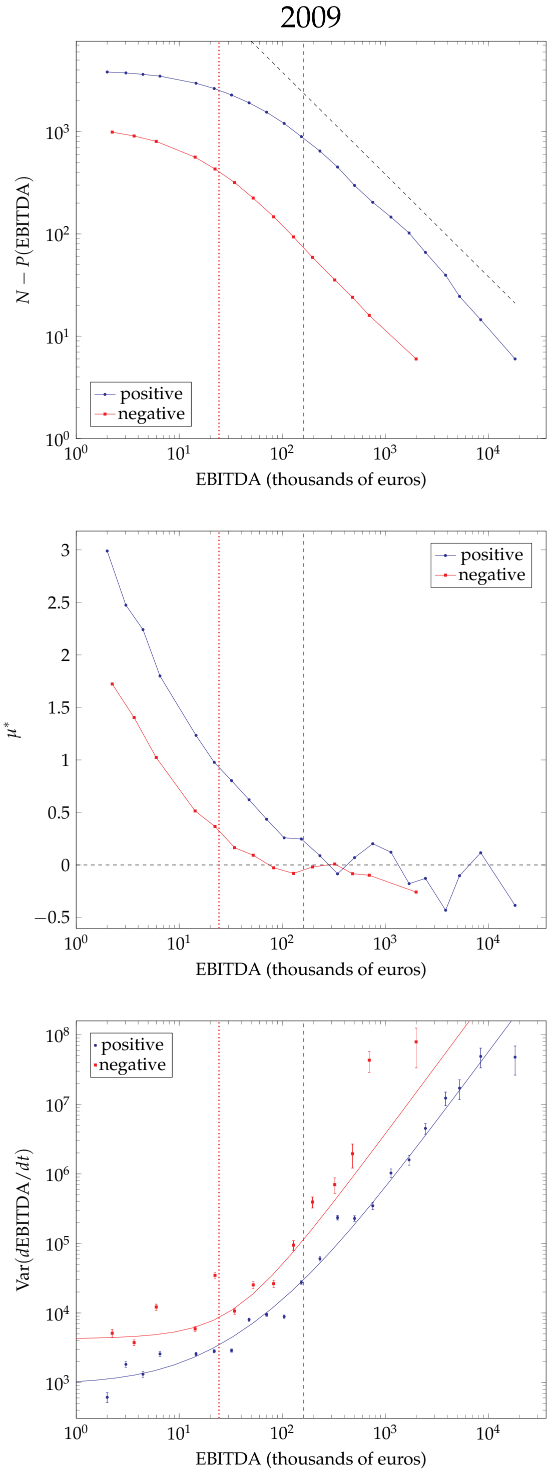

Figure 77: **Cantabria 2009: Rank plot, chemical potential and variance.**
Positive EBITDA: 3937 firms.
$T_1 = 0.56 \pm 0.10$, $T_{1/2} = 90.33 \pm 31.57$, and $T_0 = 937.77 \pm 346.81$
Negative EBITDA: 1108 firms.
$T_1 = 3.68 \pm 2.56$, $T_{1/2} = 89.37 \pm 170.23$, and $T_0 = 4217.63 \pm 1477.89$.
Total active firms 5045, total created firms 675, and total destroyed firms 485

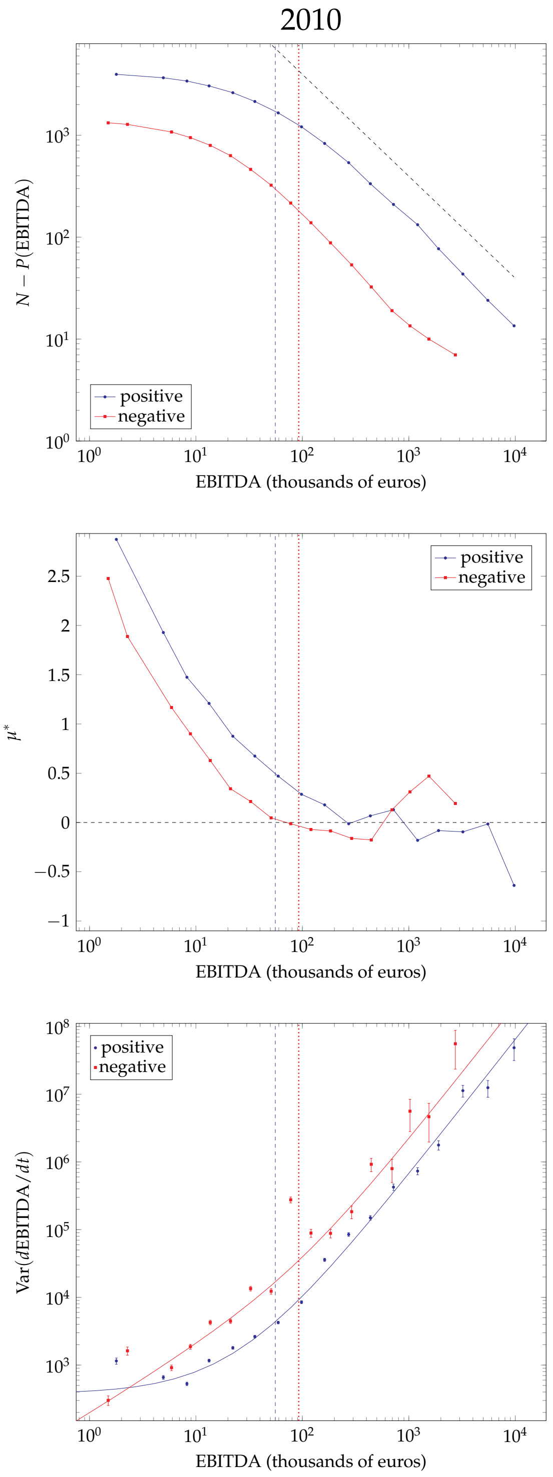

Figure 78: **Cantabria 2010: Rank plot, chemical potential and variance.**
Positive EBITDA: 4106 firms.
$T_1 = 0.64 \pm 0.11$, $T_{1/2} = 35.76 \pm 11.89$, and $T_0 = 376.68 \pm 138.91$
Negative EBITDA: 1441 firms.
$T_1 = 2.05 \pm 0.90$, $T_{1/2} = 189.12 \pm 45.37$, and $T_0 = 6.79 \pm 204.56$.
Total active firms 5547, total created firms 704, and total destroyed firms 1208

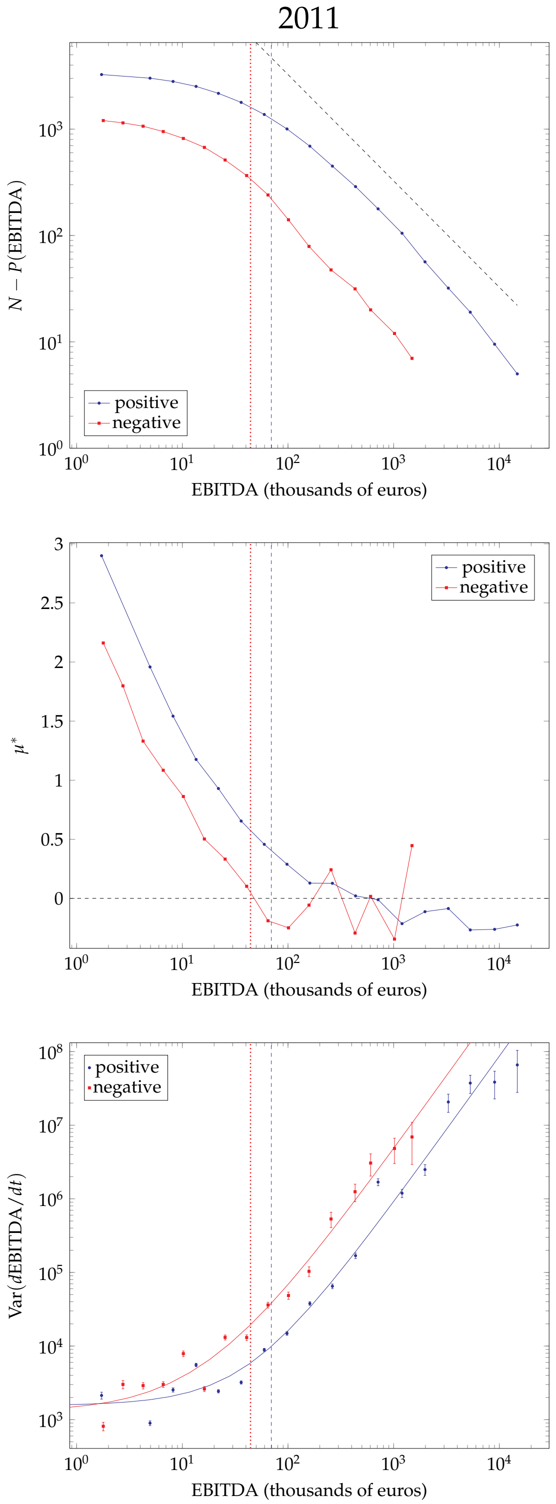

Figure 79: **Cantabria 2011: Rank plot, chemical potential and variance.**
Positive EBITDA: 3374 firms.
$T_1 = 0.86 \pm 0.20$, $T_{1/2} = 59.86 \pm 50.05$, and $T_0 = 1549.23 \pm 713.91$
Negative EBITDA: 1299 firms.
$T_1 = 4.66 \pm 1.09$, $T_{1/2} = 205.97 \pm 112.02$, and $T_0 = 1294.66 \pm 666.53$.
Total active firms 4673, total created firms 245, and total destroyed firms 1015

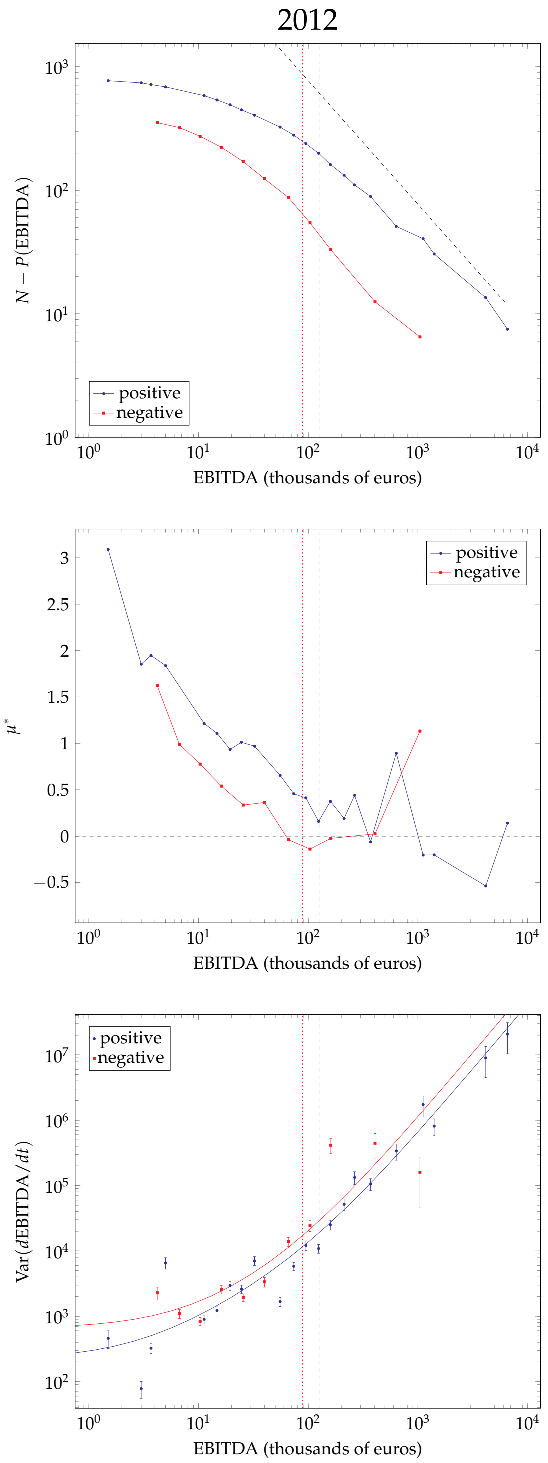

Figure 80: **Cantabria 2012: Rank plot, chemical potential and variance.**
Positive EBITDA: 791 firms.
$T_1 = 0.59 \pm 0.22$, $T_{1/2}$= 75.46 ± 36.86, and T_0 =218.10 ± 227.09
Negative EBITDA: 414 firms.
$T_1 = 1.05 \pm 1.05$, $T_{1/2}$= 92.85 ± 132.00, and T_0 =652.20 ± 1333.54.
Total active firms 1205, total created firms 24, and total destroyed firms 4319

Castile and Leon

Tables of Temperatures

Positive EBITDA

Year	T_1	$T_{1/2}$	T_0	$T_{1/2}/T_1$	Num. Firms
2003	0.31 ± 0.05	35.63 ± 14.21	457.71 ± 153.07	116.0	20859
2004	0.44 ± 0.05	23.50 ± 4.84	241.06 ± 52.41	53.5	21830
2005	0.44 ± 0.08	31.86 ± 5.78	202.75 ± 31.25	71.7	22848
2006	0.39 ± 0.10	36.87 ± 9.67	313.71 ± 62.79	94.1	23941
2007	0.57 ± 0.15	41.97 ± 11.10	164.16 ± 73.58	74.0	23346
2008	0.80 ± 0.10	41.48 ± 21.83	959.14 ± 245.10	51.7	21761
2009	0.85 ± 0.12	62.58 ± 11.75	551.67 ± 185.87	73.7	23026
2010	0.32 ± 0.14	79.42 ± 18.88	297.07 ± 177.15	250.4	24145
2011	0.67 ± 0.13	166.94 ± 50.81	363.52 ± 293.23	249.2	21393
2012	0.28 ± 0.10	58.92 ± 14.34	328.57 ± 87.88	207.2	9818

Negative EBITDA

Year	T_1	$T_{1/2}$	T_0	$T_{1/2}/T_1$	Num. Firms
2003	1.44 ± 0.45	114.35 ± 408.42	1861.15 ± 796.98	79.7	4216
2004	1.44 ± 0.38	131.19 ± 56.50	165.40 ± 70.79	91.1	4329
2005	2.12 ± 0.54	112.05 ± 168.75	975.87 ± 223.78	52.9	4534
2006	1.49 ± 0.69	110.31 ± 273.75	739.48 ± 321.00	73.9	4886
2007	3.52 ± 0.92	256.31 ± 327.07	766.67 ± 284.21	72.9	4256
2008	8.31 ± 2.28	95.18 ± 1357.76	1380.04 ± 1059.91	11.5	4071
2009	4.39 ± 0.99	270.28 ± 237.49	233.28 ± 187.86	61.6	6309
2010	2.80 ± 0.43	148.01 ± 287.46	643.05 ± 333.22	52.9	8019
2011	3.87 ± 1.61	366.03 ± 800.98	376.87 ± 389.60	94.7	7519
2012	2.56 ± 0.85	74.39 ± 219.32	672.49 ± 257.52	29.1	4499

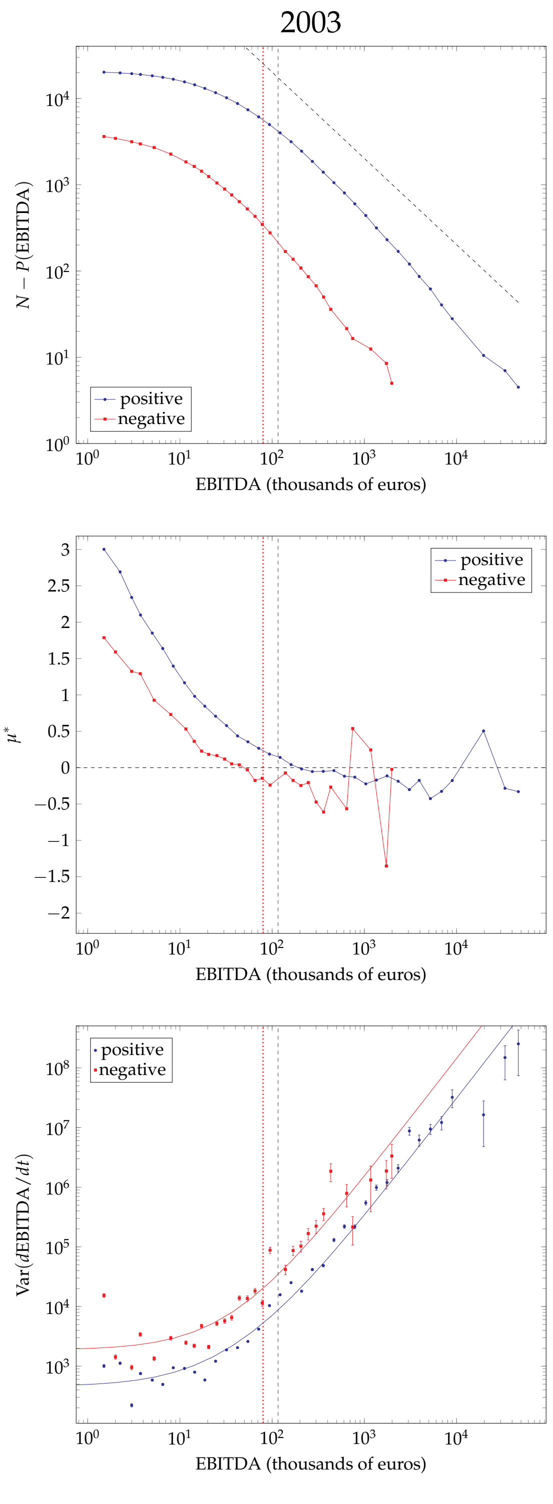

Figure 81: **Castile and Leon 2003: Rank plot, chemical potential and variance**.
Positive EBITDA: 20859 firms.
T_1 = 0.31 ± 0.05, $T_{1/2}$= 35.63 ± 14.21, and T_0 =457.71 ± 153.07
Negative EBITDA: 4216 firms.
T_1 = 1.44 ± 0.45, $T_{1/2}$= 114.35 ± 70.20, and T_0 =1861.15 ± 796.98.
Total active firms 25075, total created firms 4568, and total destroyed firms 935

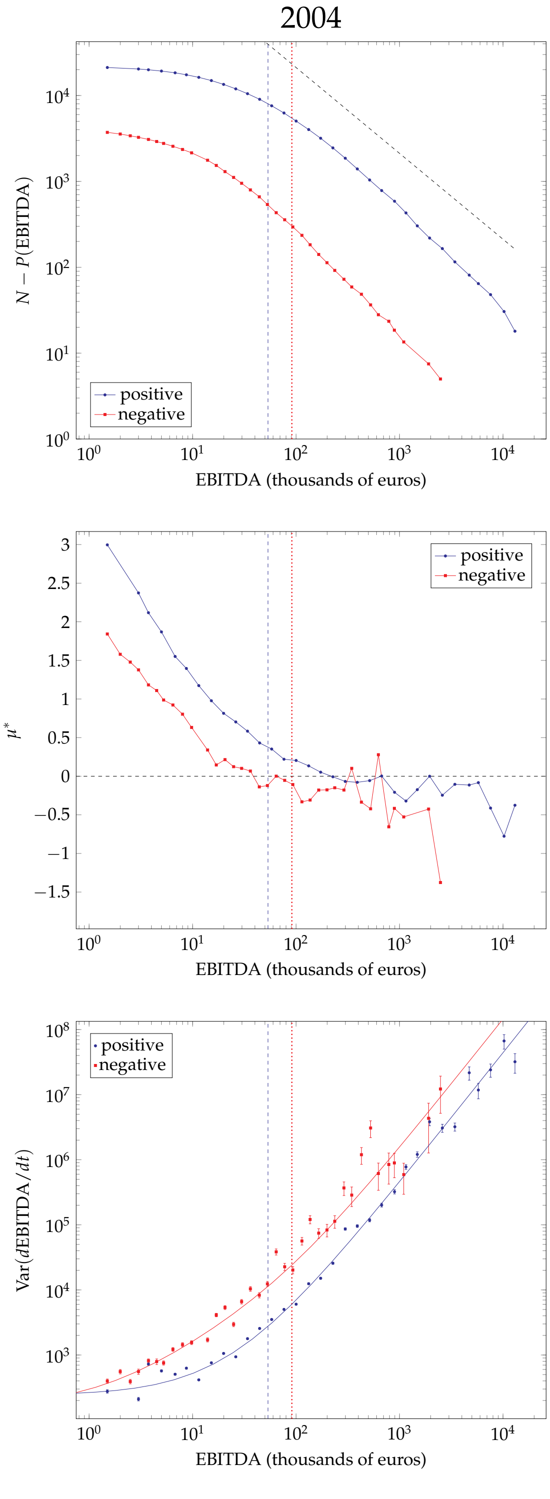

Figure 82: **Castile and Leon 2004: Rank plot, chemical potential and variance.**
Positive EBITDA: 21830 firms.
T_1 = 0.44 ± 0.05, $T_{1/2}$= 23.50 ± 4.84, and T_0 =241.06 ± 52.41
Negative EBITDA: 4329 firms.
T_1 = 1.44 ± 0.38, $T_{1/2}$= 131.19 ± 17.33, and T_0 =165.40 ± 70.79.
Total active firms 26159, total created firms 3780, and total destroyed firms 3436

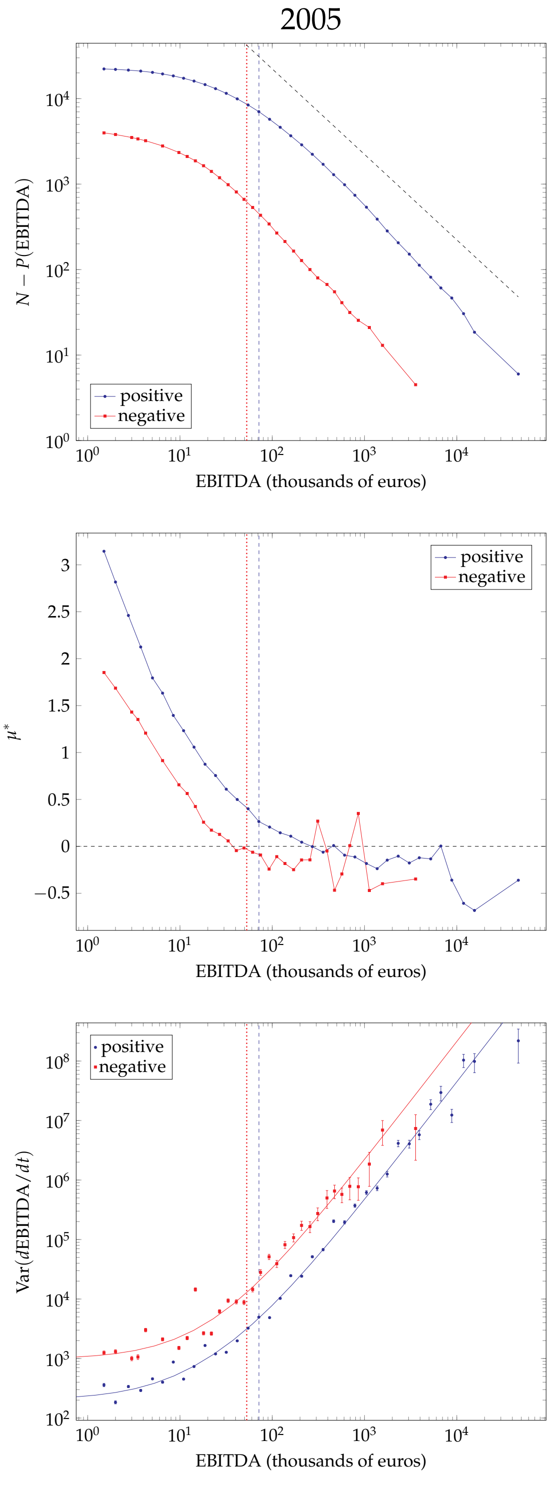

Figure 83: **Castile and Leon 2005: Rank plot, chemical potential and variance.**
Positive EBITDA: 22848 firms.
T_1 = 0.44 ± 0.08, $T_{1/2}$= 31.86 ± 5.78, and T_0 =202.75 ± 31.25
Negative EBITDA: 4534 firms.
T_1 = 2.12 ± 0.54, $T_{1/2}$= 112.05 ± 37.57, and T_0 =975.87 ± 223.78.
Total active firms 27382, total created firms 4457, and total destroyed firms 2560

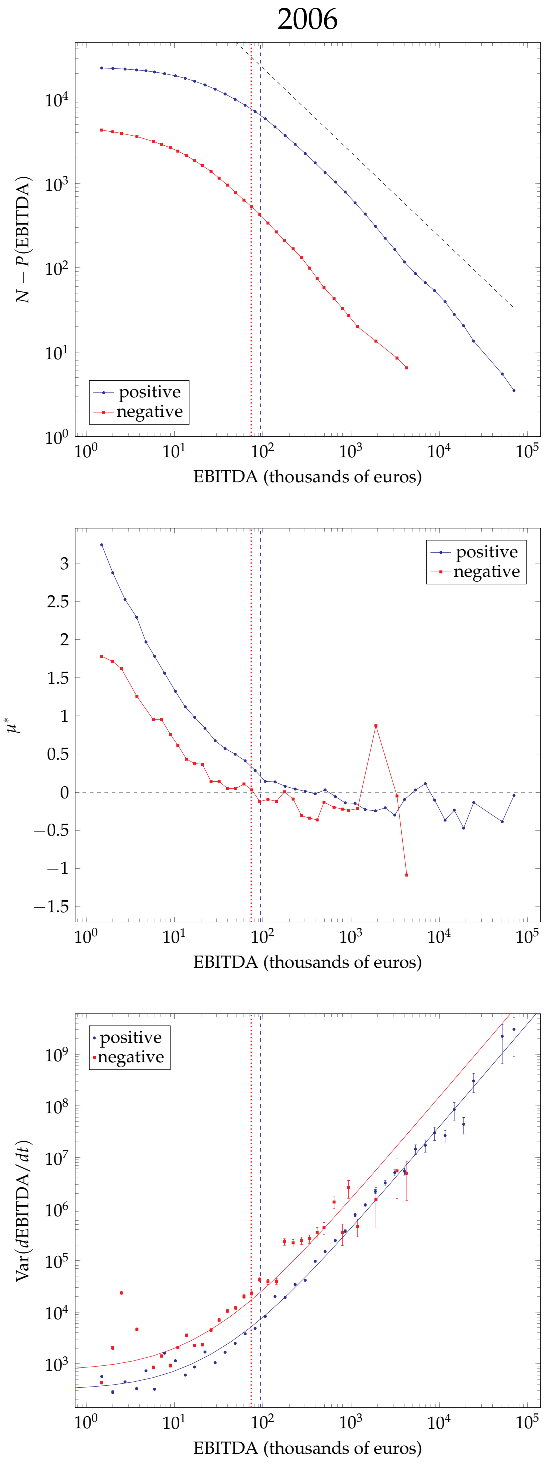

Figure 84: **Castile and Leon 2006: Rank plot, chemical potential and variance.**
Positive EBITDA: 23941 firms.
$T_1 = 0.39 \pm 0.10$, $T_{1/2}$= 36.87 ± 9.67, and T_0 =313.71 ± 62.79
Negative EBITDA: 4886 firms.
$T_1 = 1.49 \pm 0.69$, $T_{1/2}$= 110.31 ± 52.90, and T_0 =739.48 ± 321.00.
Total active firms 28827, total created firms 4225, and total destroyed firms 3008

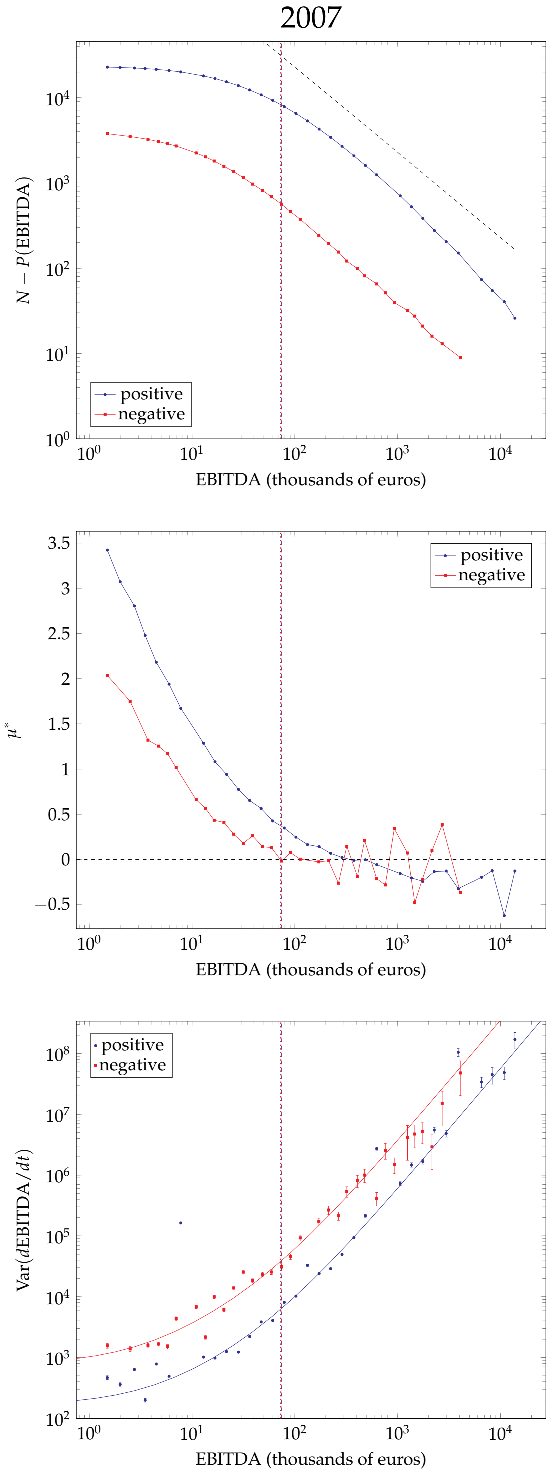

Figure 85: **Castile and Leon 2007: Rank plot, chemical potential and variance.**
Positive EBITDA: 23346 firms.
$T_1 = 0.57 \pm 0.15$, $T_{1/2} = 41.97 \pm 11.10$, and $T_0 = 164.16 \pm 73.58$
Negative EBITDA: 4256 firms.
$T_1 = 3.52 \pm 0.92$, $T_{1/2} = 256.31 \pm 60.00$, and $T_0 = 766.67 \pm 284.21$.
Total active firms 27602, total created firms 1958, and total destroyed firms 5611

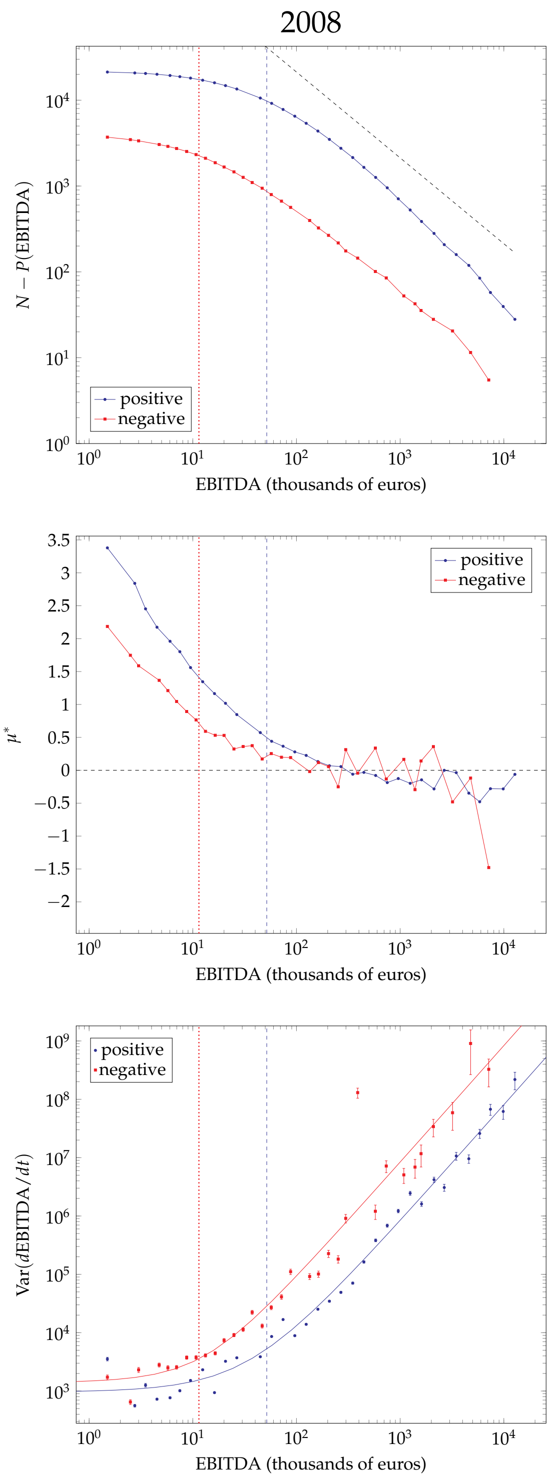

Figure 86: **Castile and Leon 2008: Rank plot, chemical potential and variance**.
Positive EBITDA: 21761 firms.
$T_1 = 0.80 \pm 0.10$, $T_{1/2} = 41.48 \pm 21.83$, and $T_0 = 959.14 \pm 245.10$
Negative EBITDA: 4071 firms.
$T_1 = 8.31 \pm 2.28$, $T_{1/2} = 95.18 \pm 164.15$, and $T_0 = 1380.04 \pm 1059.91$.
Total active firms 25832, total created firms 9137, and total destroyed firms 3778

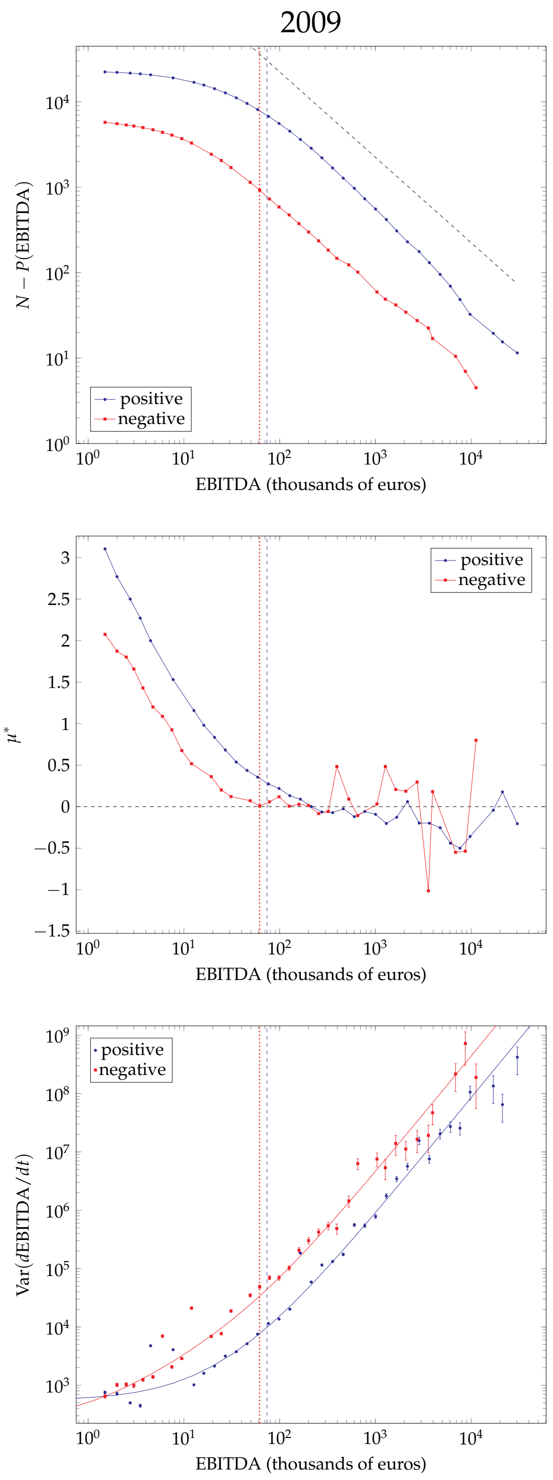

Figure 87: **Castile and Leon 2009: Rank plot, chemical potential and variance.**
Positive EBITDA: 23026 firms.
$T_1 = 0.85 \pm 0.12$, $T_{1/2} = 62.58 \pm 11.75$, and $T_0 = 551.67 \pm 185.87$
Negative EBITDA: 6309 firms.
$T_1 = 4.39 \pm 0.99$, $T_{1/2} = 270.28 \pm 47.84$, and $T_0 = 233.28 \pm 187.86$.
Total active firms 29335, total created firms 3774, and total destroyed firms 2755

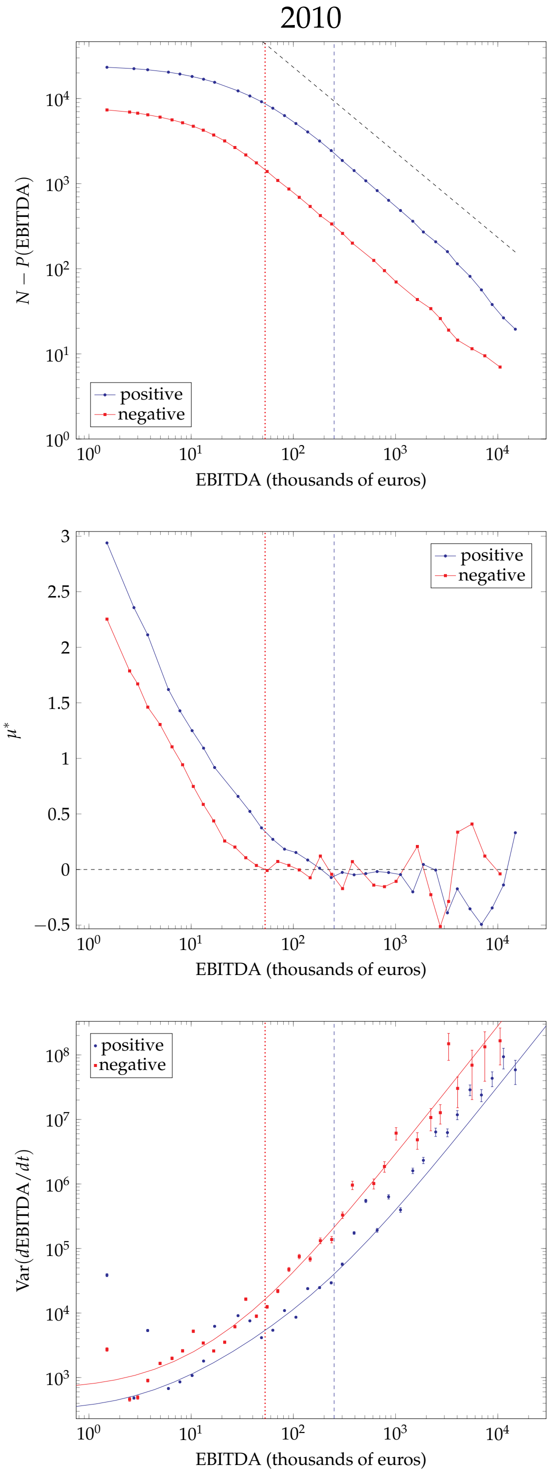

Figure 88: **Castile and Leon 2010: Rank plot, chemical potential and variance.**
Positive EBITDA: 24145 firms.
$T_1 = 0.32 \pm 0.14$, $T_{1/2}$= 79.42 ± 18.88, and T_0 =297.07 ± 177.15
Negative EBITDA: 8019 firms.
$T_1 = 2.80 \pm 0.43$, $T_{1/2}$= 148.01 ± 54.76, and T_0 =643.05 ± 333.22.
Total active firms 32164, total created firms 3133, and total destroyed firms 3639

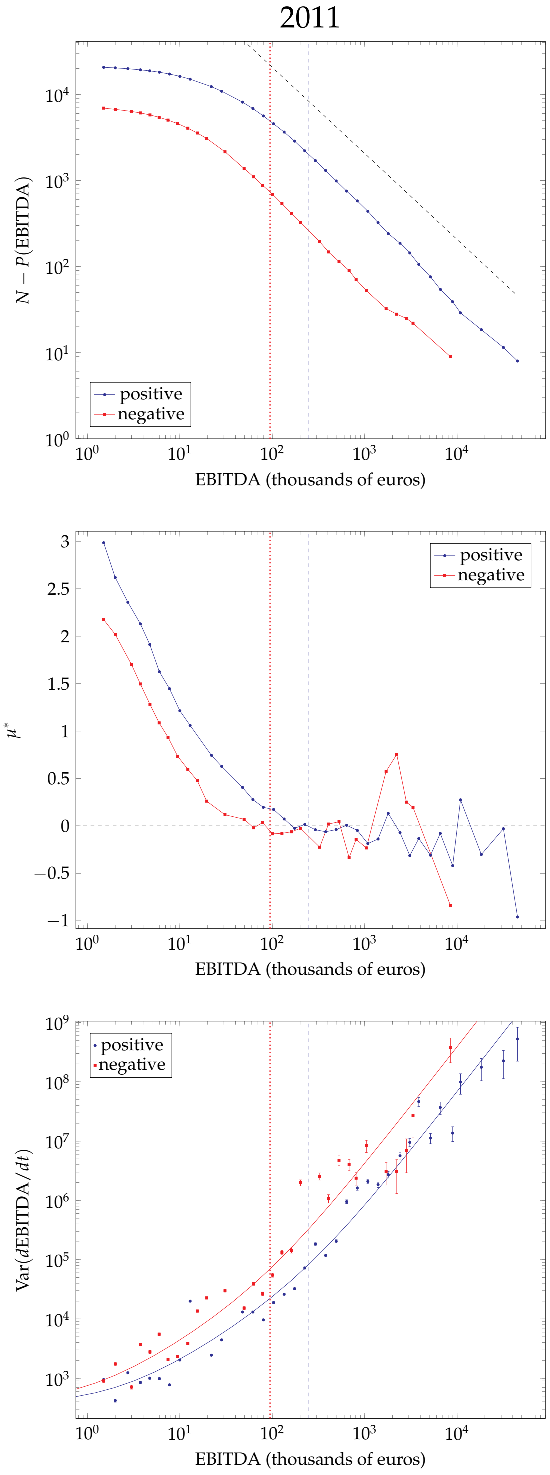

Figure 89: **Castile and Leon 2011: Rank plot, chemical potential and variance.**
Positive EBITDA: 21393 firms.
T_1 = 0.67 ± 0.13, $T_{1/2}$= 166.94 ± 50.81, and T_0 =363.52 ± 293.23
Negative EBITDA: 7519 firms.
T_1 = 3.87 ± 1.61, $T_{1/2}$= 366.03 ± 113.02, and T_0 =376.87 ± 389.60.
Total active firms 28912, total created firms 1298, and total destroyed firms 3888

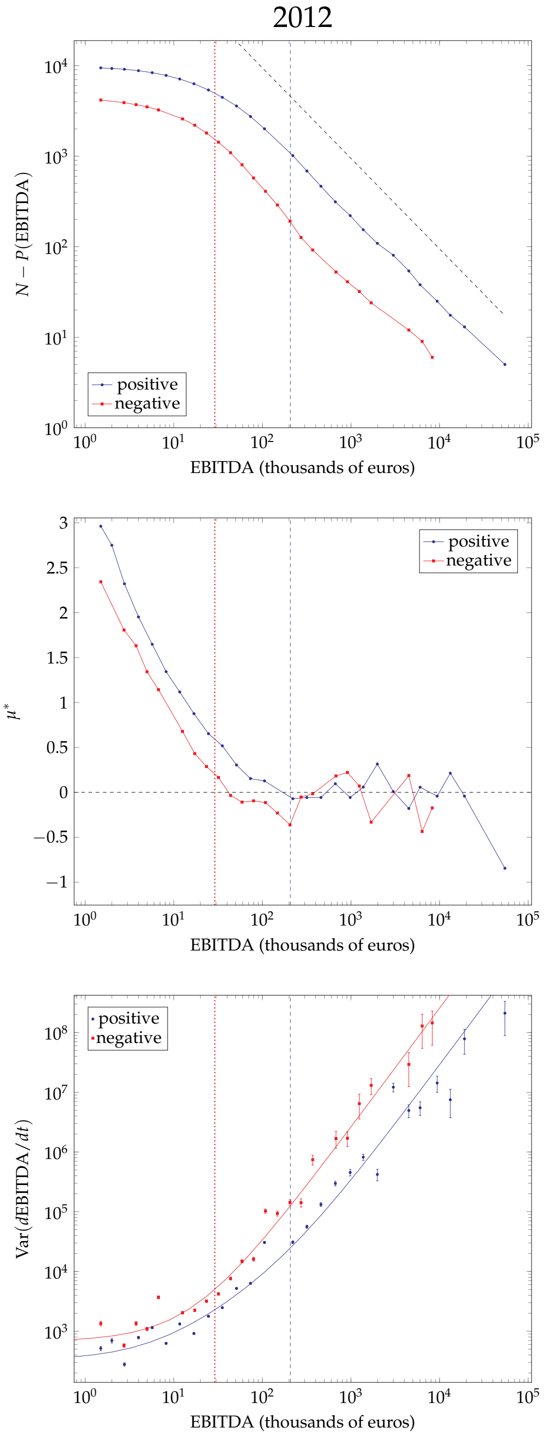

Figure 90: **Castile and Leon 2012: Rank plot, chemical potential and variance.**
Positive EBITDA: 9818 firms.
T_1 = 0.28 ± 0.10, $T_{1/2}$= 58.92 ± 14.34, and T_0 =328.57 ± 87.88
Negative EBITDA: 4499 firms.
T_1 = 2.56 ± 0.85, $T_{1/2}$= 74.39 ± 45.23, and T_0 =672.49 ± 257.52.
Total active firms 14317, total created firms 254, and total destroyed firms 18659

Castile-La Mancha

Tables of Temperatures

Positive EBITDA

Year	T_1	$T_{1/2}$	T_0	$T_{1/2}/T_1$	Num. Firms
2003	0.31 ± 0.17	58.09 ± 19.95	178.18 ± 81.30	188.0	17315
2004	0.44 ± 0.07	23.50 ± 4.78	146.76 ± 52.87	54.0	17471
2005	0.51 ± 0.09	19.51 ± 5.06	224.22 ± 41.02	38.0	18584
2006	0.51 ± 0.08	24.64 ± 6.73	456.04 ± 78.08	48.1	20087
2007	0.63 ± 0.10	43.57 ± 18.02	437.30 ± 143.34	68.8	21278
2008	0.81 ± 0.18	35.09 ± 12.80	494.16 ± 90.97	43.3	19014
2009	0.88 ± 0.21	70.23 ± 20.14	687.10 ± 124.23	79.8	18793
2010	0.72 ± 0.13	38.95 ± 20.70	543.04 ± 183.35	53.8	19699
2011	0.73 ± 0.12	63.57 ± 24.81	640.80 ± 234.29	86.9	16739
2012	0.41 ± 0.09	44.74 ± 15.47	302.95 ± 118.68	109.1	7095

Negative EBITDA

Year	T_1	$T_{1/2}$	T_0	$T_{1/2}/T_1$	Num. Firms
2003	2.27 ± 0.94	96.73 ± 274.97	511.55 ± 329.63	42.7	3183
2004	1.57 ± 0.52	112.26 ± 129.04	176.21 ± 123.71	71.4	3046
2005	1.01 ± 0.25	204.14 ± 103.35	494.41 ± 135.88	202.5	3389
2006	2.19 ± 0.53	131.74 ± 219.63	1500.59 ± 340.09	60.1	3640
2007	3.45 ± 0.88	165.62 ± 763.43	1552.31 ± 812.22	48.0	3486
2008	5.50 ± 2.21	91.38 ± 1003.09	2637.24 ± 948.74	16.6	3462
2009	3.72 ± 0.75	224.29 ± 250.05	1053.95 ± 252.82	60.3	5344
2010	3.85 ± 0.54	106.53 ± 239.54	794.93 ± 283.20	27.7	7084
2011	4.44 ± 0.84	69.47 ± 166.62	1131.32 ± 268.59	15.7	6474
2012	3.58 ± 1.05	204.50 ± 615.53	0.09 ± 306.36	57.2	3443

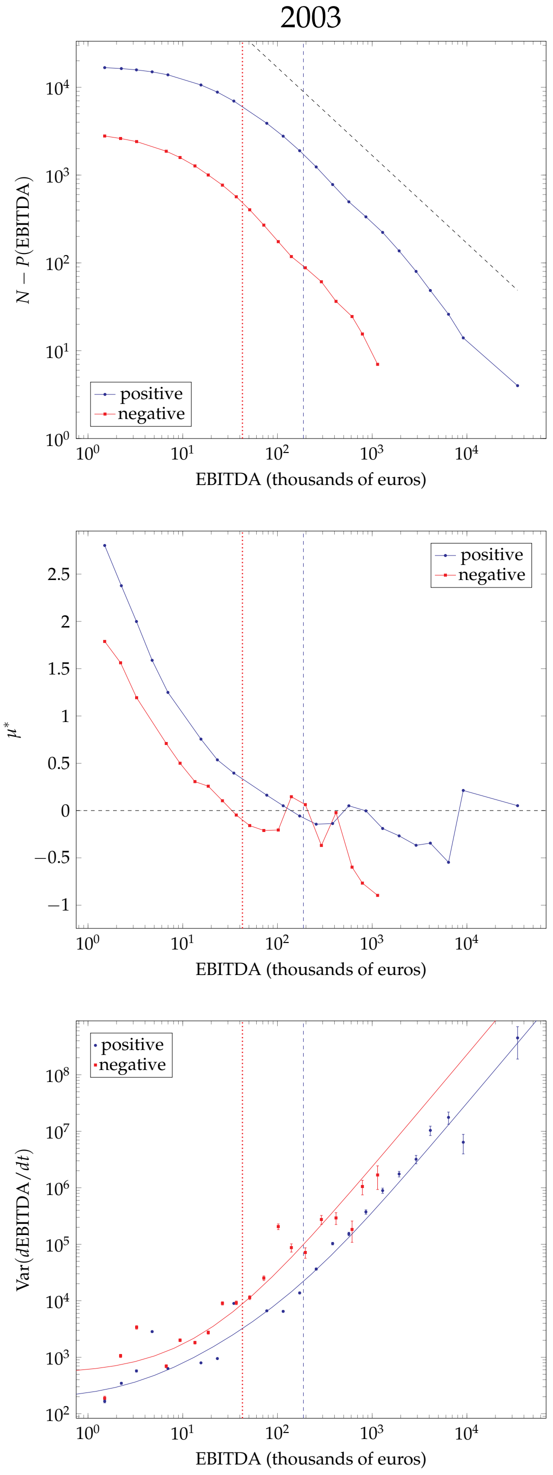

Figure 91: **Castile-La Mancha 2003: Rank plot, chemical potential and variance.**
Positive EBITDA: 17315 firms.
T_1 = 0.31 ± 0.17, $T_{1/2}$= 58.09 ± 19.95, and T_0 =178.18 ± 81.30
Negative EBITDA: 3183 firms.
T_1 = 2.27 ± 0.94, $T_{1/2}$= 96.73 ± 53.07, and T_0 =511.55 ± 329.63.
Total active firms 20498, total created firms 3711, and total destroyed firms 953

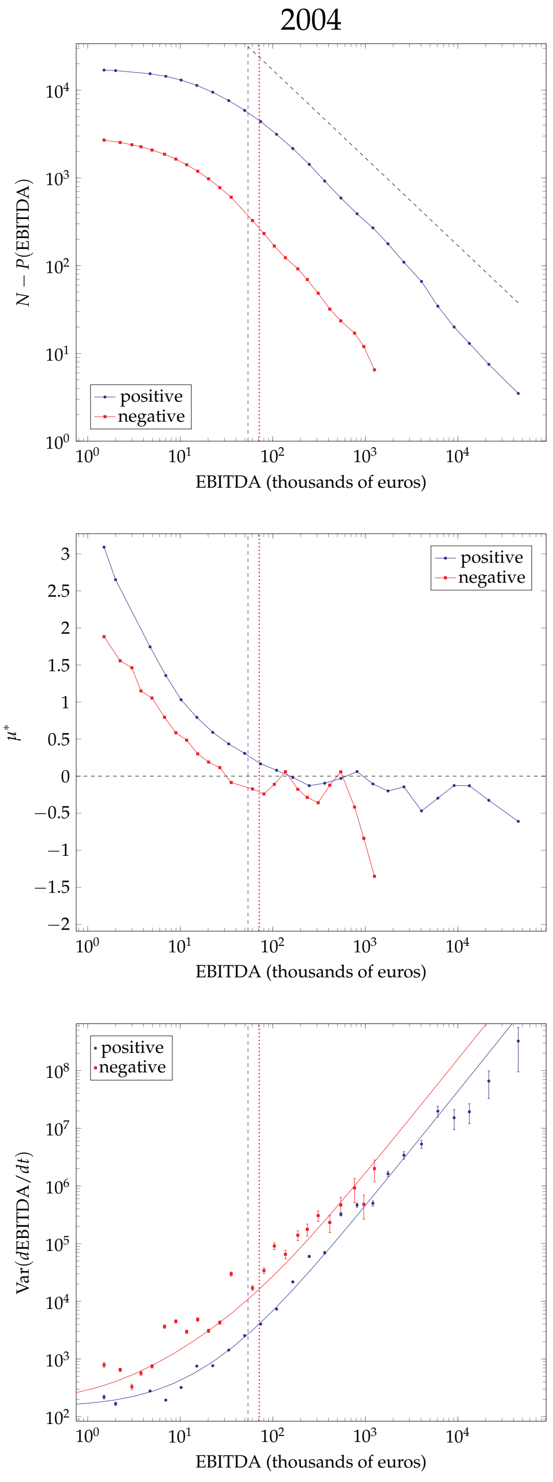

Figure 92: **Castile-La Mancha 2004: Rank plot, chemical potential and variance**.
Positive EBITDA: 17471 firms.
T_1 = 0.44 ± 0.07, $T_{1/2}$= 23.50 ± 4.78, and T_0 =146.76 ± 52.87
Negative EBITDA: 3046 firms.
T_1 = 1.57 ± 0.52, $T_{1/2}$= 112.26 ± 31.08, and T_0 =176.21 ± 123.71.
Total active firms 20517, total created firms 3841, and total destroyed firms 3733

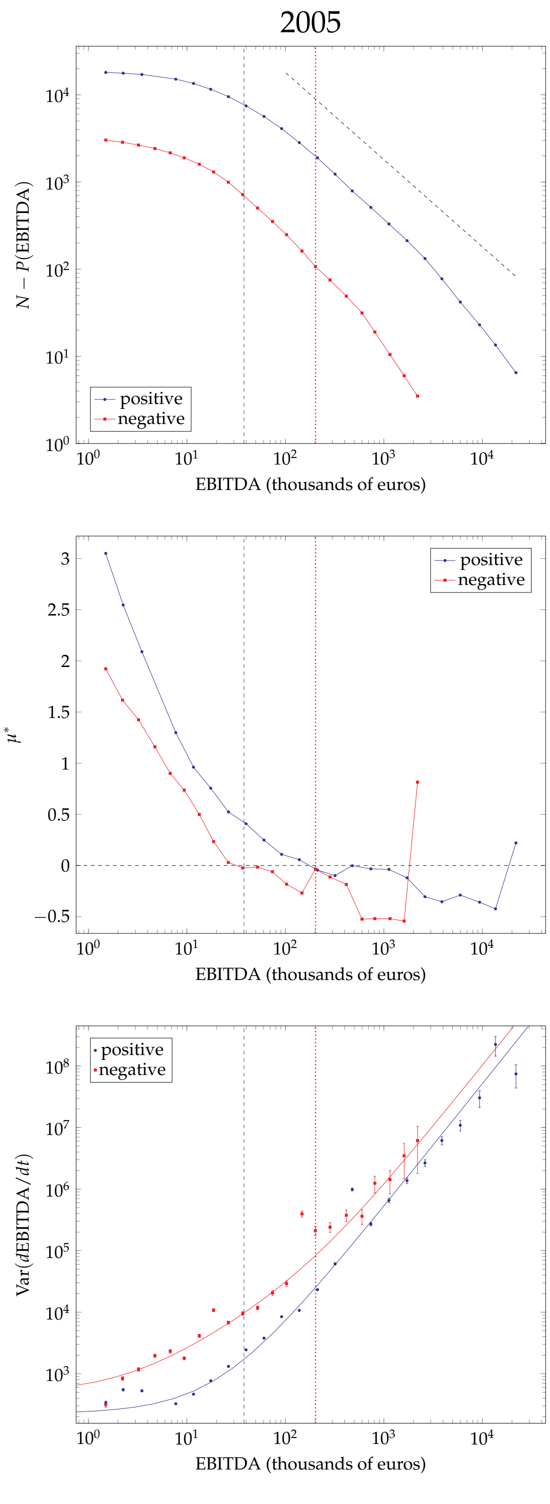

Figure 93: **Castile-La Mancha 2005: Rank plot, chemical potential and variance.**
Positive EBITDA: 18584 firms.
$T_1 = 0.51 \pm 0.09$, $T_{1/2} = 19.51 \pm 5.06$, and $T_0 = 224.22 \pm 41.02$
Negative EBITDA: 3389 firms.
$T_1 = 1.01 \pm 0.25$, $T_{1/2} = 204.14 \pm 26.57$, and $T_0 = 494.41 \pm 135.88$.
Total active firms 21973, total created firms 4109, and total destroyed firms 2398

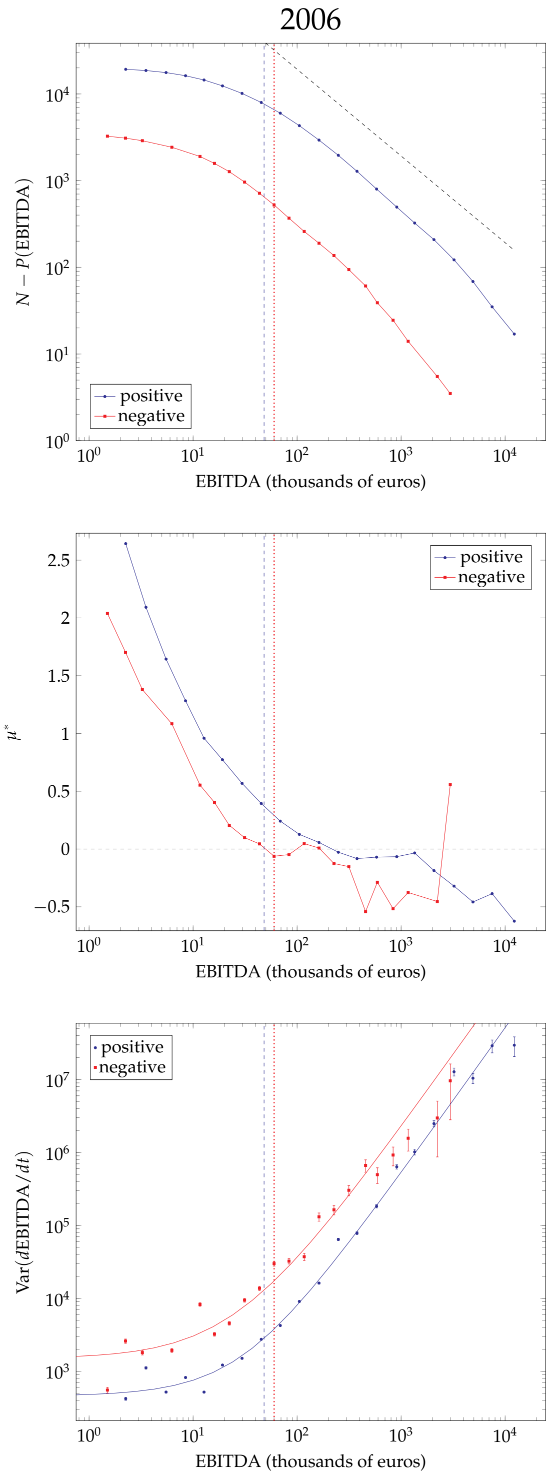

Figure 94: **Castile-La Mancha 2006: Rank plot, chemical potential and variance.**
Positive EBITDA: 20087 firms.
$T_1 = 0.51 \pm 0.08$, $T_{1/2} = 24.64 \pm 6.73$, and $T_0 = 456.04 \pm 78.08$
Negative EBITDA: 3640 firms.
$T_1 = 2.19 \pm 0.53$, $T_{1/2} = 131.74 \pm 45.27$, and $T_0 = 1500.59 \pm 340.09$.
Total active firms 23727, total created firms 4706, and total destroyed firms 2346

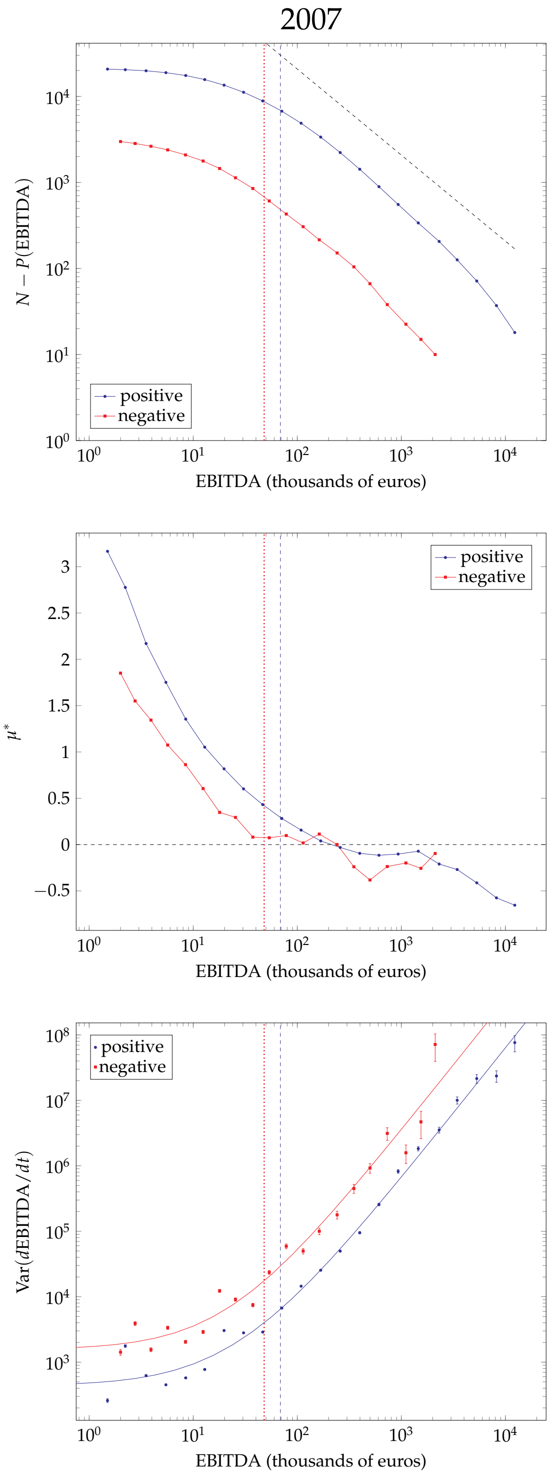

Figure 95: **Castile-La Mancha 2007: Rank plot, chemical potential and variance.**
Positive EBITDA: 21278 firms.
$T_1 = 0.63 \pm 0.10$, $T_{1/2}$= 43.57 ± 18.02, and T_0 =437.30 ± 143.34
Negative EBITDA: 3486 firms.
$T_1 = 3.45 \pm 0.88$, $T_{1/2}$= 165.62 ± 109.25, and T_0 =1552.31 ± 812.22.
Total active firms 24764, total created firms 1847, and total destroyed firms 3687

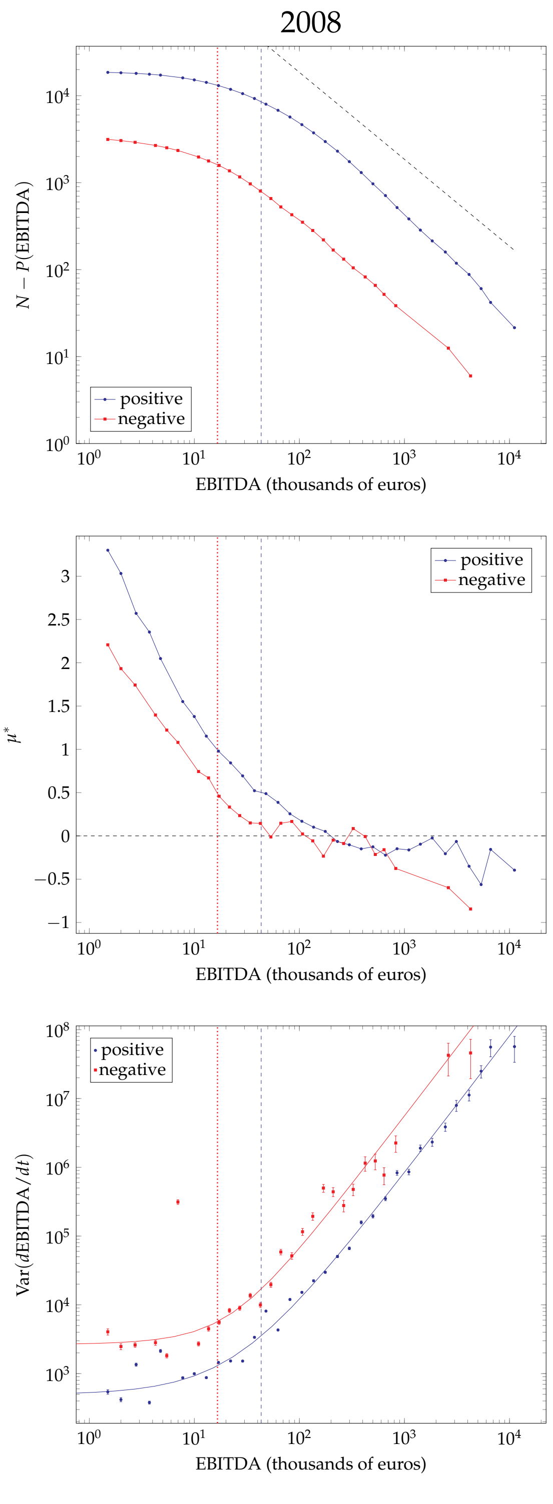

Figure 96: **Castile-La Mancha 2008: Rank plot, chemical potential and variance.**
Positive EBITDA: 19014 firms.
$T_1 = 0.81 \pm 0.18$, $T_{1/2} = 35.09 \pm 12.80$, and $T_0 = 494.16 \pm 90.97$
Negative EBITDA: 3462 firms.
$T_1 = 5.50 \pm 2.21$, $T_{1/2} = 91.38 \pm 132.52$, and $T_0 = 2637.24 \pm 948.74$.
Total active firms 22476, total created firms 6783, and total destroyed firms 4215

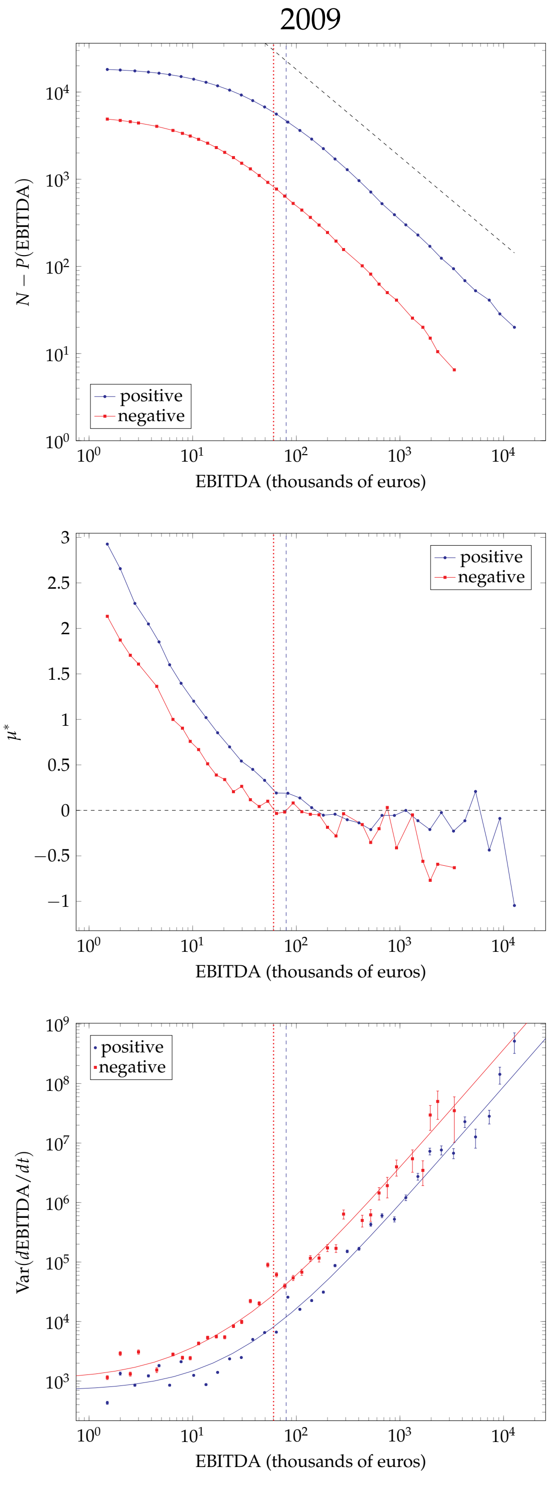

Figure 97: **Castile-La Mancha 2009: Rank plot, chemical potential and variance.**
Positive EBITDA: 18793 firms.
T_1 = 0.88 ± 0.21, $T_{1/2}$= 70.23 ± 20.14, and T_0 =687.10 ± 124.23
Negative EBITDA: 5344 firms.
T_1 = 3.72 ± 0.75, $T_{1/2}$= 224.29 ± 49.62, and T_0 =1053.95 ± 252.82.
Total active firms 24137, total created firms 3572, and total destroyed firms 2872

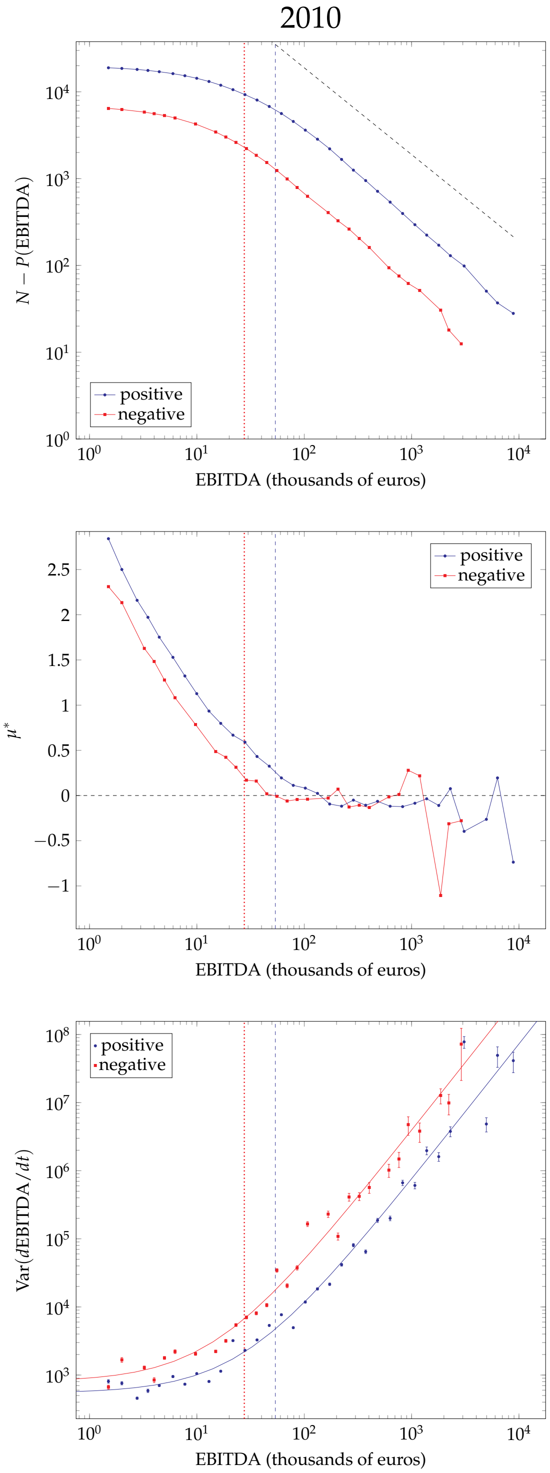

Figure 98: **Castile-La Mancha 2010: Rank plot, chemical potential and variance.**
Positive EBITDA: 19699 firms.
$T_1 = 0.72 \pm 0.13$, $T_{1/2} = 38.95 \pm 20.70$, and $T_0 = 543.04 \pm 183.35$
Negative EBITDA: 7084 firms.
$T_1 = 3.85 \pm 0.54$, $T_{1/2} = 106.53 \pm 48.14$, and $T_0 = 794.93 \pm 283.20$.
Total active firms 26783, total created firms 3797, and total destroyed firms 2989

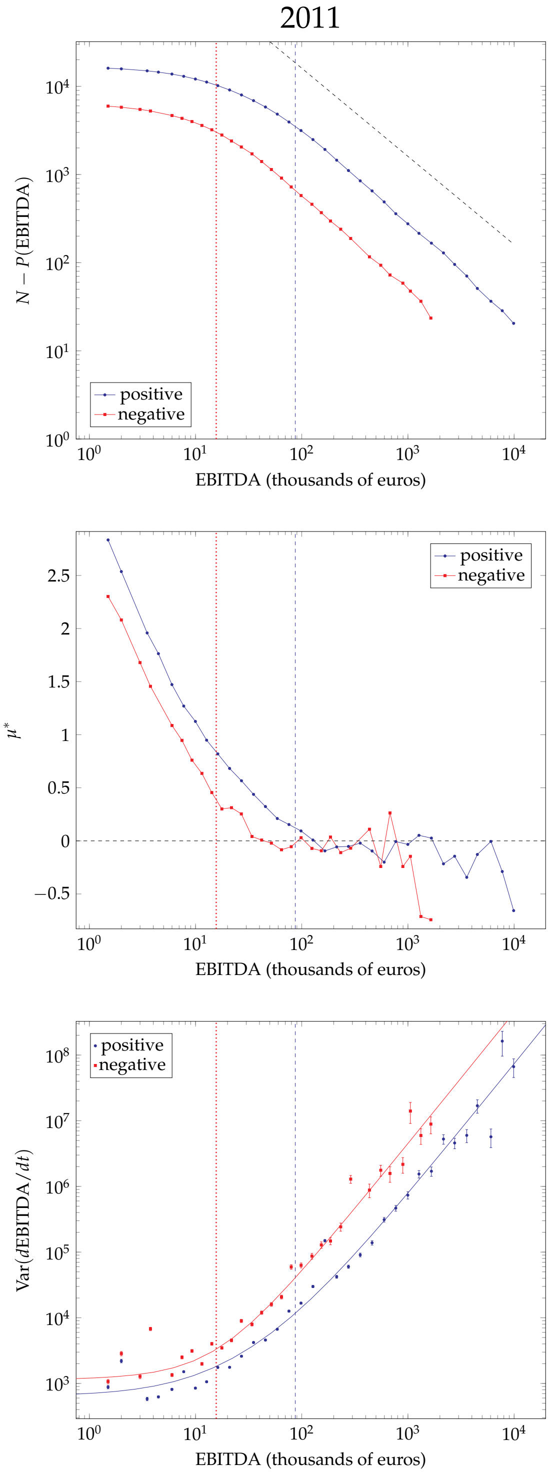

Figure 99: **Castile-La Mancha 2011: Rank plot, chemical potential and variance**.
Positive EBITDA: 16739 firms.
T_1 = 0.73 ± 0.12, $T_{1/2}$= 63.57 ± 24.81, and T_0 =640.80 ± 234.29
Negative EBITDA: 6474 firms.
T_1 = 4.44 ± 0.84, $T_{1/2}$= 69.47 ± 37.24, and T_0 =1131.32 ± 268.59.
Total active firms 23213, total created firms 1212, and total destroyed firms 4199

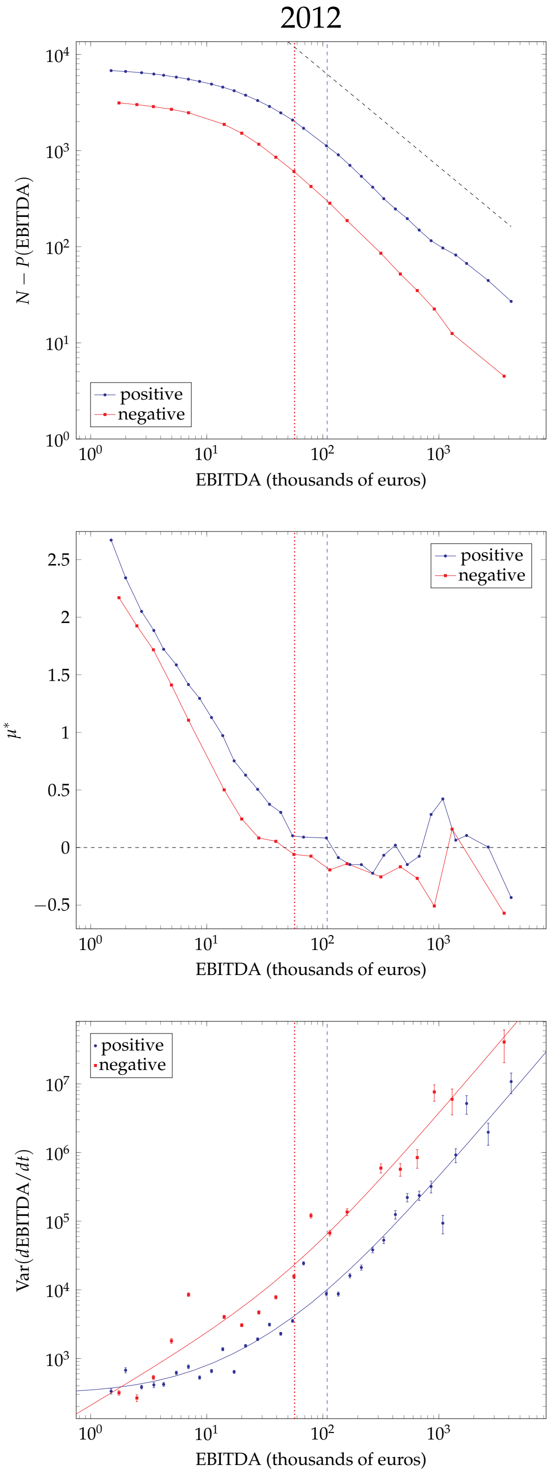

Figure 100: **Castile-La Mancha 2012: Rank plot, chemical potential and variance.**
Positive EBITDA: 7095 firms.
$T_1 = 0.41 \pm 0.09$, $T_{1/2} = 44.74 \pm 15.47$, and $T_0 = 302.95 \pm 118.68$
Negative EBITDA: 3443 firms.
$T_1 = 3.58 \pm 1.05$, $T_{1/2} = 204.50 \pm 93.82$, and $T_0 = 0.09 \pm 306.36$.
Total active firms 10538, total created firms 235, and total destroyed firms 17288

Catalonia

Tables of Temperatures

Positive EBITDA

Year	T_1	$T_{1/2}$	T_0	$T_{1/2}/T_1$	Num. Firms
2003	0.31 ± 0.04	83.48 ± 19.13	512.72 ± 168.87	268.6	91748
2004	0.39 ± 0.06	51.16 ± 22.70	1090.99 ± 336.70	132.9	93147
2005	0.49 ± 0.06	59.20 ± 9.21	534.85 ± 158.65	120.9	96104
2006	0.45 ± 0.04	92.28 ± 15.30	501.92 ± 121.58	205.7	100103
2007	0.51 ± 0.09	170.30 ± 59.84	1216.80 ± 575.70	336.0	102128
2008	0.96 ± 0.12	133.55 ± 40.81	1159.67 ± 369.78	139.8	89672
2009	1.15 ± 0.14	114.69 ± 39.41	834.58 ± 291.96	99.8	97832
2010	0.58 ± 0.09	115.03 ± 44.73	1648.23 ± 614.71	198.3	95302
2011	0.61 ± 0.07	132.49 ± 29.03	332.28 ± 176.74	216.0	90294
2012	0.50 ± 0.09	60.10 ± 39.72	2076.04 ± 766.90	119.9	49057

Negative EBITDA

Year	T_1	$T_{1/2}$	T_0	$T_{1/2}/T_1$	Num. Firms
2003	2.37 ± 0.31	259.84 ± 257.29	60.26 ± 189.38	109.8	21739
2004	2.62 ± 0.37	277.53 ± 257.87	544.37 ± 246.96	106.0	21780
2005	1.58 ± 0.28	262.52 ± 139.81	591.26 ± 182.18	166.5	22515
2006	1.58 ± 0.23	374.82 ± 403.31	330.24 ± 328.92	237.3	23536
2007	2.54 ± 0.61	842.74 ± 2576.69	0.15 ± 966.27	331.9	22162
2008	4.94 ± 0.85	668.16 ± 1750.20	146.01 ± 698.49	135.3	21846
2009	3.03 ± 0.50	424.12 ± 869.34	767.03 ± 636.91	140.2	36525
2010	2.73 ± 0.50	560.82 ± 1334.20	285.91 ± 737.44	205.6	38469
2011	2.28 ± 0.36	372.76 ± 673.85	0.00 ± 343.02	163.4	37913
2012	3.00 ± 0.36	113.15 ± 332.09	1788.23 ± 550.56	37.8	20407

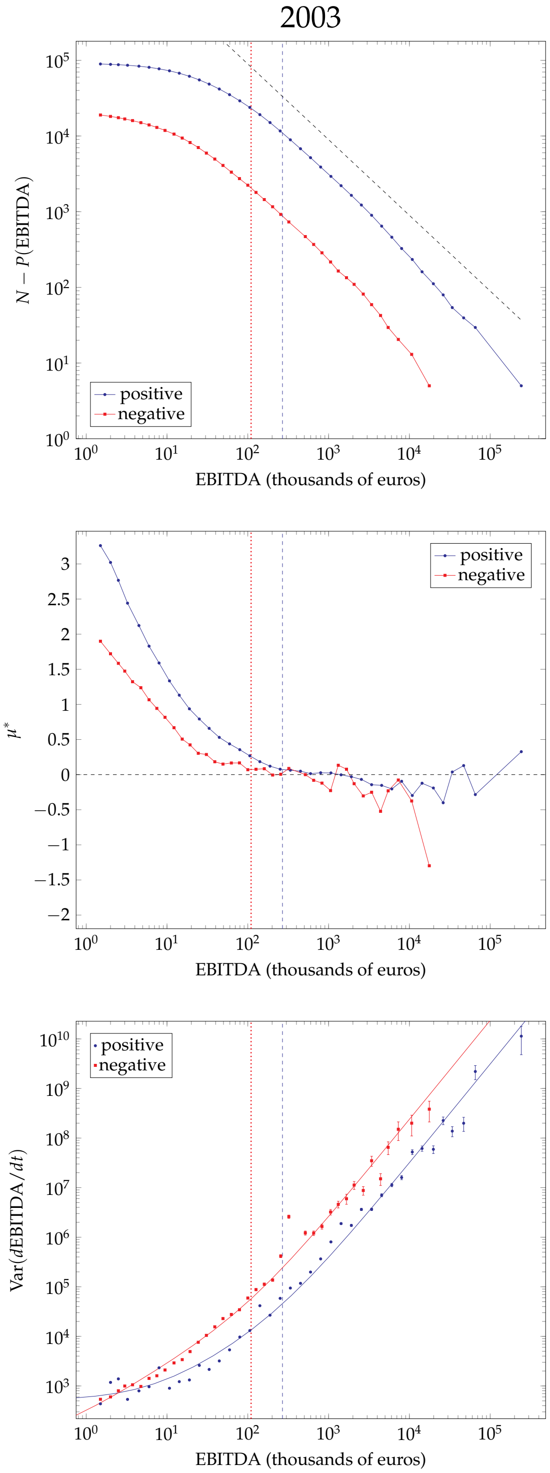

Figure 101: **Catalonia 2003: Rank plot, chemical potential and variance**.
Positive EBITDA: 91748 firms.
$T_1 = 0.31 \pm 0.04$, $T_{1/2}$= 83.48 ± 19.13, and T_0 =512.72 ± 168.87
Negative EBITDA: 21739 firms.
$T_1 = 2.37 \pm 0.31$, $T_{1/2}$= 259.84 ± 50.63, and T_0 =60.26 ± 189.38.
Total active firms 113487, total created firms 19149, and total destroyed firms 11889

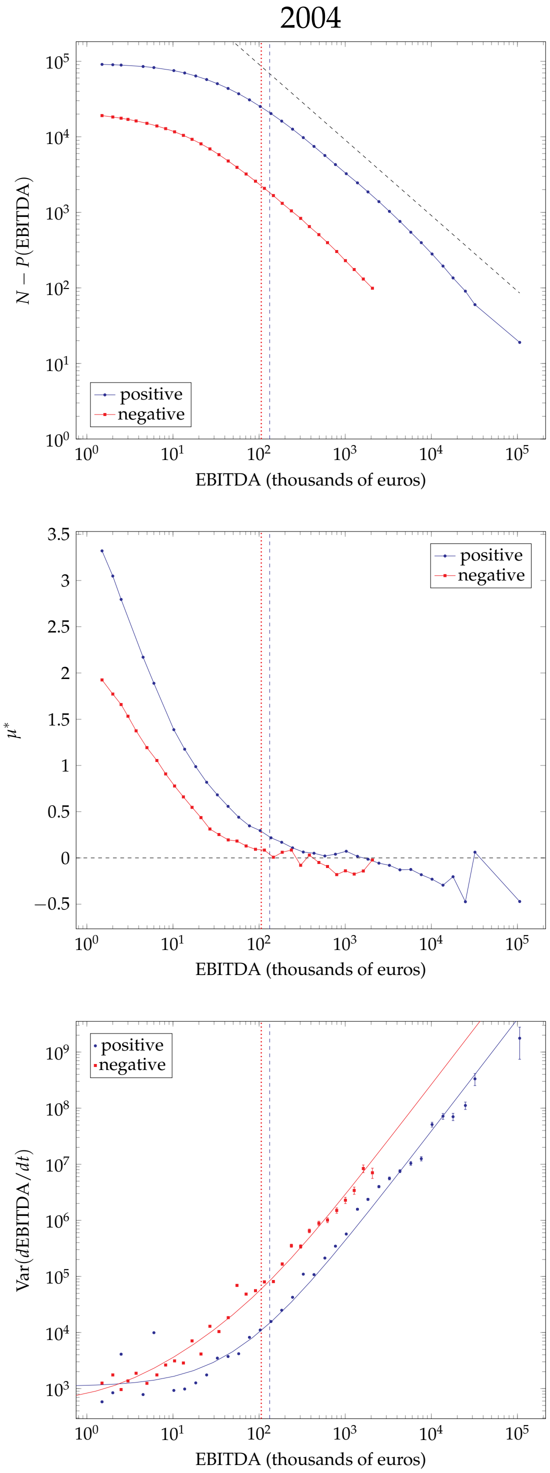

Figure 102: **Catalonia 2004: Rank plot, chemical potential and variance**.
Positive EBITDA: 93147 firms.
$T_1 = 0.39 \pm 0.06$, $T_{1/2}$= 51.16 ± 22.70, and T_0 =1090.99 ± 336.70
Negative EBITDA: 21780 firms.
$T_1 = 2.62 \pm 0.37$, $T_{1/2}$= 277.53 ± 50.71, and T_0 =544.37 ± 246.96.
Total active firms 114927, total created firms 18122, and total destroyed firms 17707

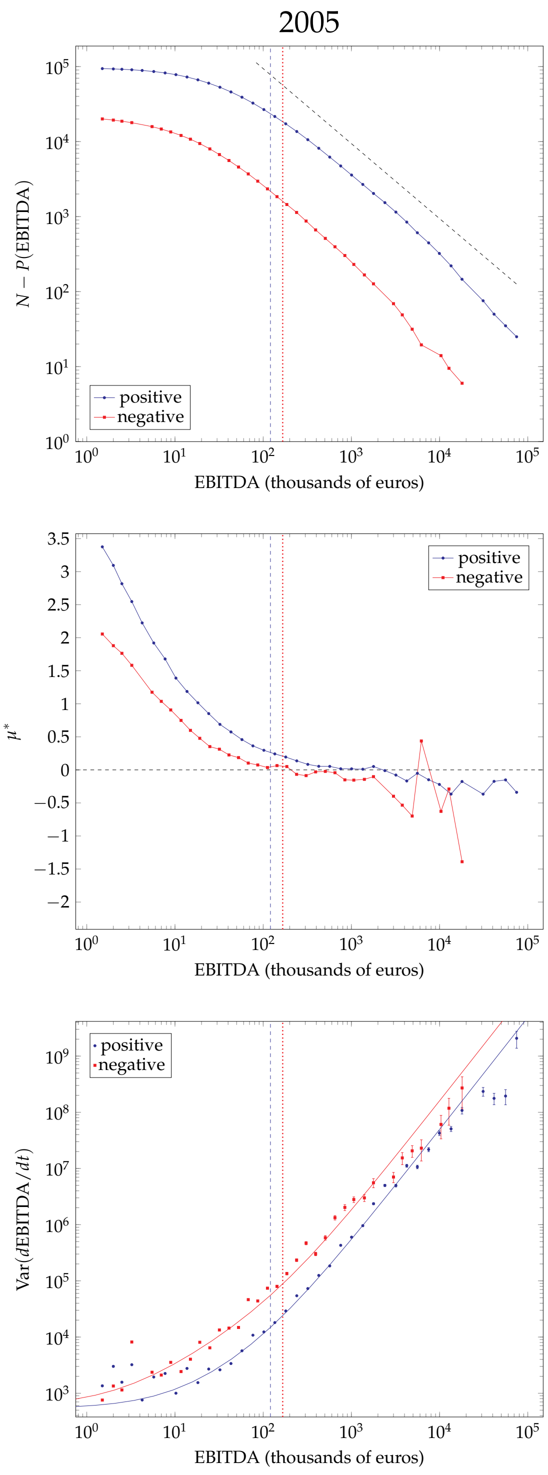

Figure 103: **Catalonia 2005: Rank plot, chemical potential and variance.**
Positive EBITDA: 96104 firms.
$T_1 = 0.49 \pm 0.06$, $T_{1/2}$= 59.20 ± 9.21, and T_0 =534.85 ± 158.65
Negative EBITDA: 22515 firms.
$T_1 = 1.58 \pm 0.28$, $T_{1/2}$= 262.52 ± 32.90, and T_0 =591.26 ± 182.18.
Total active firms 118619, total created firms 19163, and total destroyed firms 14694

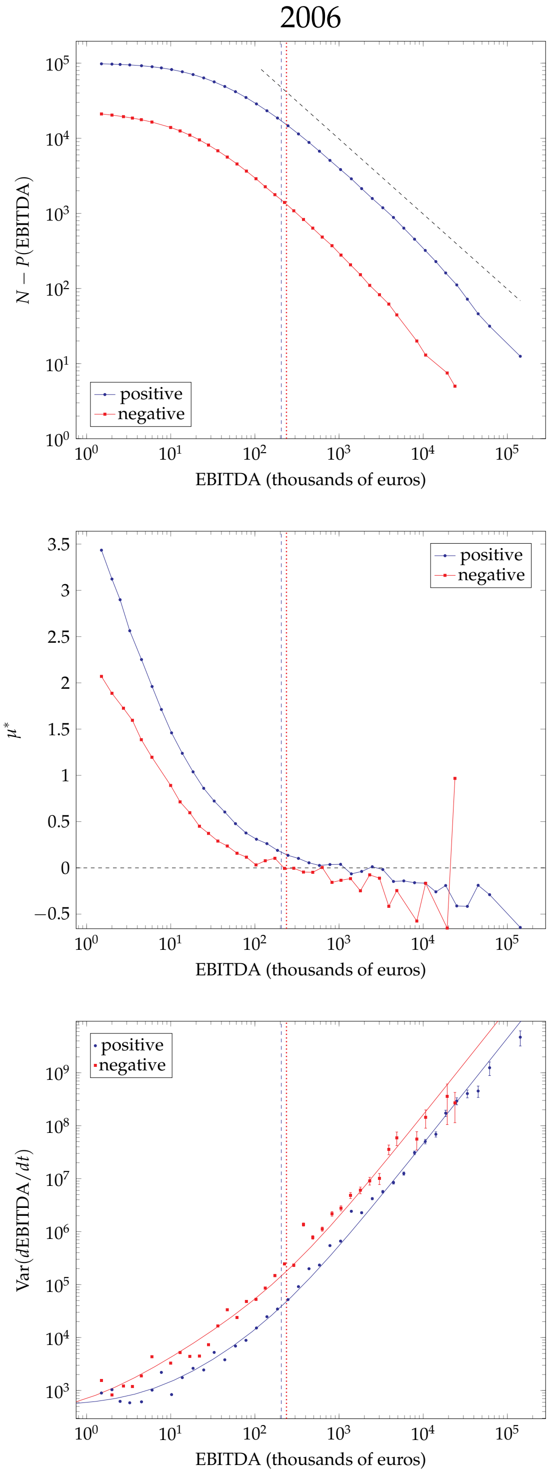

Figure 104: **Catalonia 2006: Rank plot, chemical potential and variance**.
Positive EBITDA: 100103 firms.
T_1 = 0.45 ± 0.04, $T_{1/2}$= 92.28 ± 15.30, and T_0 =501.92 ± 121.58
Negative EBITDA: 23536 firms.
T_1 = 1.58 ± 0.23, $T_{1/2}$= 374.82 ± 69.58, and T_0 =330.24 ± 328.92.
Total active firms 123639, total created firms 21992, and total destroyed firms 14069

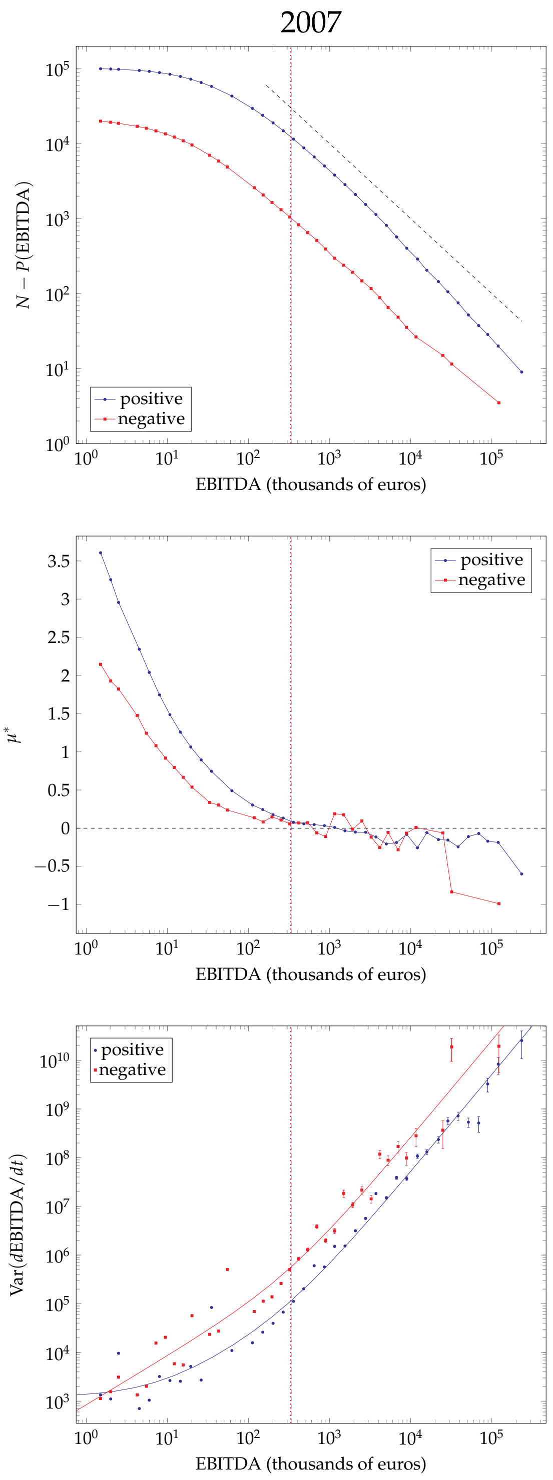

Figure 105: **Catalonia 2007: Rank plot, chemical potential and variance**.
Positive EBITDA: 102128 firms.
T_1 = 0.51 ± 0.09, $T_{1/2}$= 170.30 ± 59.84, and T_0 =1216.80 ± 575.70
Negative EBITDA: 22162 firms.
T_1 = 2.54 ± 0.61, $T_{1/2}$= 842.74 ± 258.22, and T_0 =0.15 ± 966.27.
Total active firms 124290, total created firms 7803, and total destroyed firms 21609

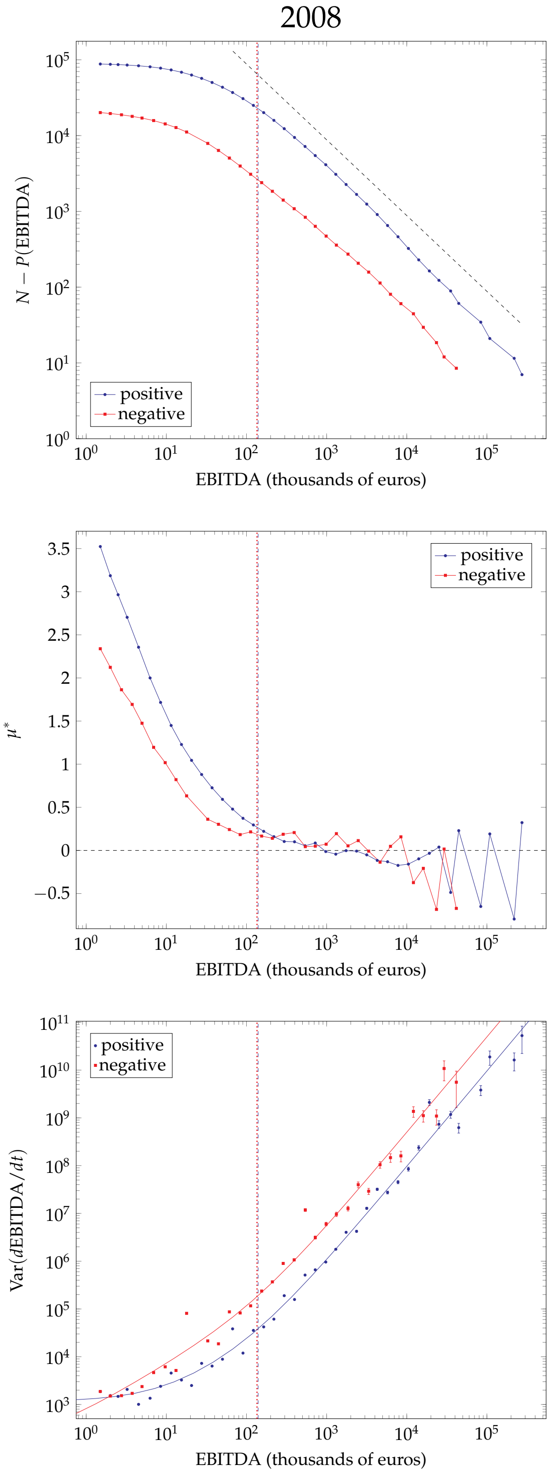

Figure 106: **Catalonia 2008: Rank plot, chemical potential and variance**.
Positive EBITDA: 89672 firms.
T_1 = 0.96 ± 0.12, $T_{1/2}$= 133.55 ± 40.81, and T_0 =1159.67 ± 369.78
Negative EBITDA: 21846 firms.
T_1 = 4.94 ± 0.85, $T_{1/2}$= 668.16 ± 196.43, and T_0 =146.01 ± 698.49.
Total active firms 111518, total created firms 37093, and total destroyed firms 20904

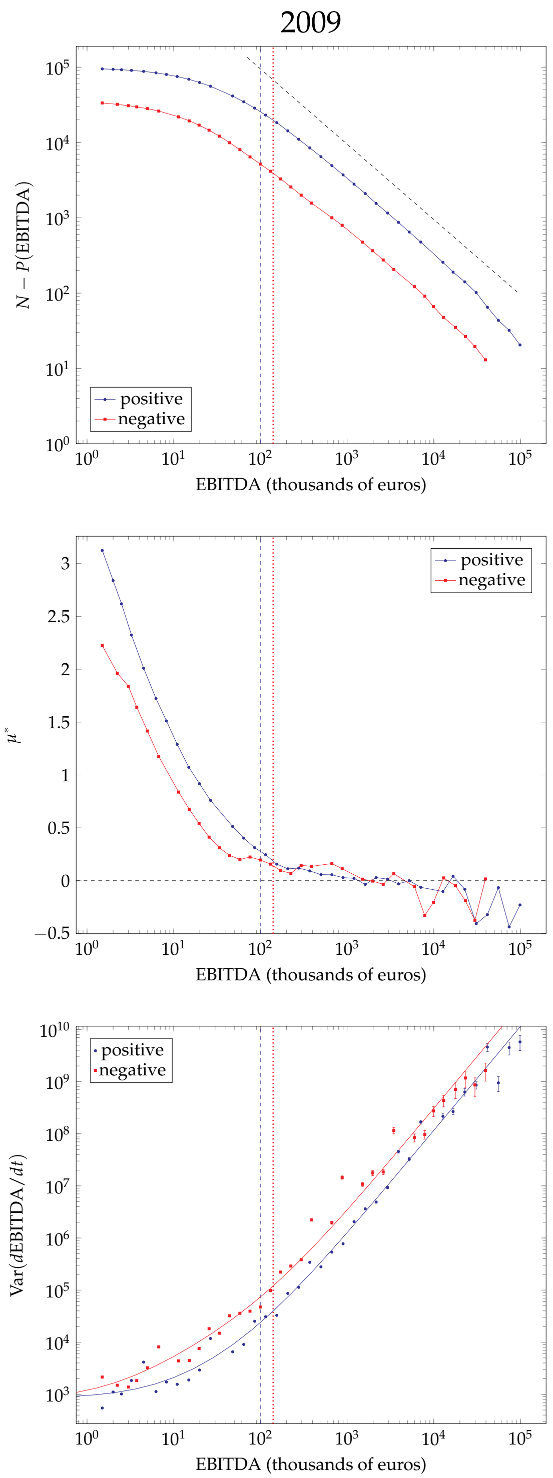

Figure 107: **Catalonia 2009: Rank plot, chemical potential and variance**.
Positive EBITDA: 97832 firms.
T_1 = 1.15 ± 0.14, $T_{1/2}$= 114.69 ± 39.41, and T_0 =834.58 ± 291.96
Negative EBITDA: 36525 firms.
T_1 = 3.03 ± 0.50, $T_{1/2}$= 424.12 ± 119.76, and T_0 =767.03 ± 636.91.
Total active firms 134357, total created firms 18293, and total destroyed firms 13524

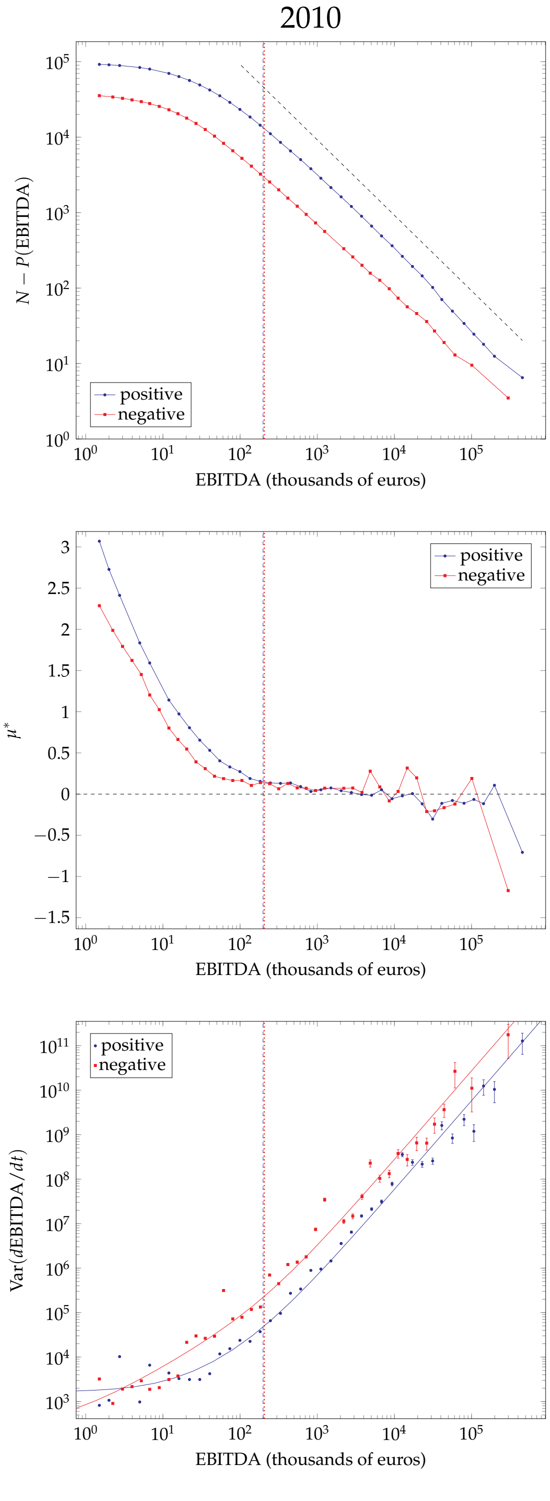

Figure 108: **Catalonia 2010: Rank plot, chemical potential and variance**.
Positive EBITDA: 95302 firms.
$T_1 = 0.58 \pm 0.09$, $T_{1/2}$= 115.03 ± 44.73, and T_0 =1648.23 ± 614.71
Negative EBITDA: 38469 firms.
$T_1 = 2.73 \pm 0.50$, $T_{1/2}$= 560.82 ± 162.13, and T_0 =285.91 ± 737.44.
Total active firms 133771, total created firms 12989, and total destroyed firms 18921

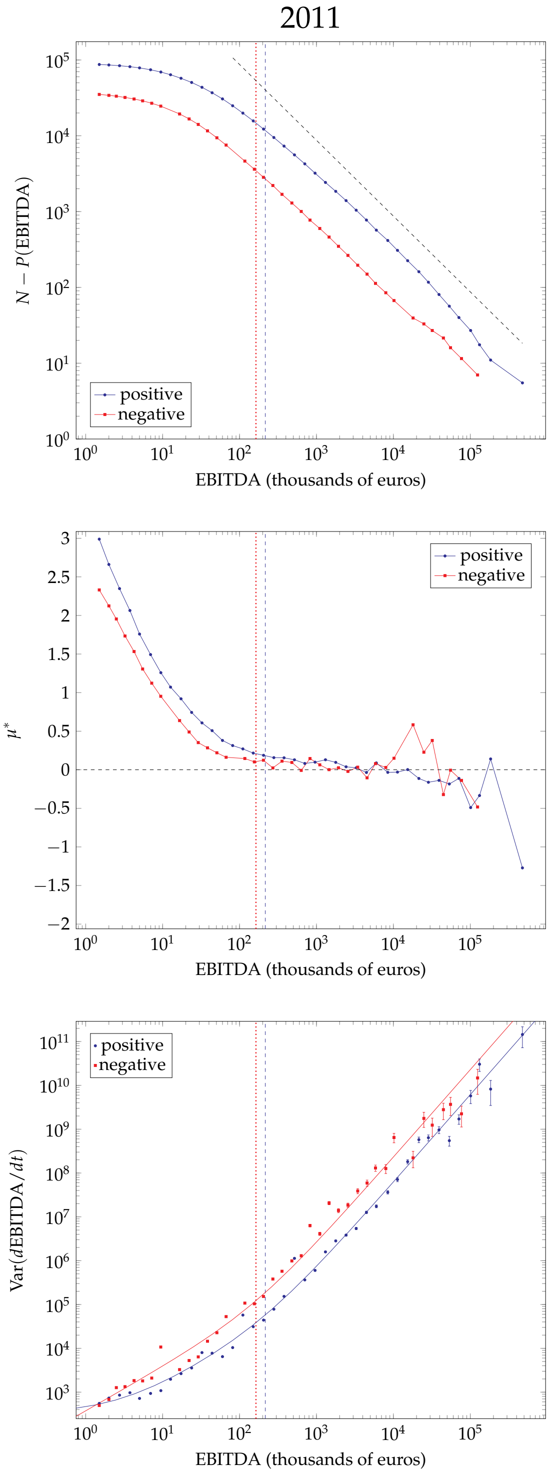

Figure 109: **Catalonia 2011: Rank plot, chemical potential and variance**.
Positive EBITDA: 90294 firms.
T_1 = 0.61 ± 0.07, $T_{1/2}$= 132.49 ± 29.03, and T_0 =332.28 ± 176.74
Negative EBITDA: 37913 firms.
T_1 = 2.28 ± 0.36, $T_{1/2}$= 372.76 ± 100.02, and T_0 =0.00 ± 343.02.
Total active firms 128207, total created firms 5458, and total destroyed firms 18703

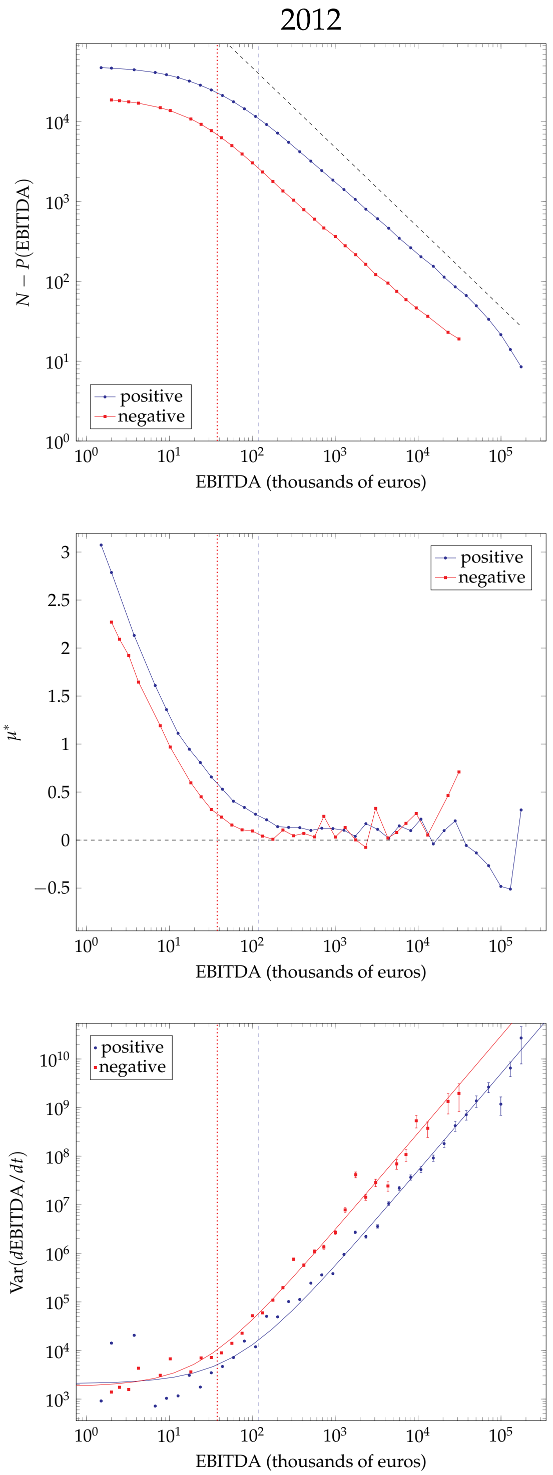

Figure 110: **Catalonia 2012: Rank plot, chemical potential and variance**.
Positive EBITDA: 49057 firms.
T_1 = 0.50 ± 0.09, $T_{1/2}$= 60.10 ± 39.72, and T_0 =2076.04 ± 766.90
Negative EBITDA: 20407 firms.
T_1 = 3.00 ± 0.36, $T_{1/2}$= 113.15 ± 60.65, and T_0 =1788.23 ± 550.56.
Total active firms 69464, total created firms 1164 19163, and total destroyed firms 65090 14694

Extremadura

Tables of Temperatures

Positive EBITDA

Year	T_1	$T_{1/2}$	T_0	$T_{1/2}/T_1$	Num. Firms
2003	0.44 ± 0.12	14.92 ± 9.76	364.16 ± 87.07	33.7	7186
2004	0.54 ± 0.19	44.04 ± 12.30	126.42 ± 56.07	82.1	7582
2005	0.33 ± 0.06	53.19 ± 15.90	226.61 ± 102.99	161.1	7825
2006	0.45 ± 0.10	32.49 ± 7.44	343.62 ± 47.17	71.5	8027
2007	0.43 ± 0.07	30.41 ± 6.85	128.97 ± 72.52	71.3	8285
2008	0.81 ± 0.15	37.50 ± 20.96	359.75 ± 144.80	46.5	7281
2009	0.68 ± 0.21	157.34 ± 61.34	144.96 ± 300.80	231.4	7457
2010	0.62 ± 0.14	80.84 ± 31.60	351.66 ± 209.75	130.7	8285
2011	0.70 ± 0.23	76.80 ± 20.12	205.87 ± 82.07	109.3	7137
2012	0.29 ± 0.22	72.10 ± 20.57	50.72 ± 77.34	250.0	3135

Negative EBITDA

Year	T_1	$T_{1/2}$	T_0	$T_{1/2}/T_1$	Num. Firms
2003	1.89 ± 0.85	135.01 ± 245.58	533.65 ± 202.59	71.4	1437
2004	1.54 ± 0.83	170.43 ± 224.51	0.00 ± 108.91	110.9	1472
2005	1.68 ± 0.97	165.07 ± 743.66	468.30 ± 621.78	98.3	1634
2006	1.84 ± 0.72	161.84 ± 275.98	456.68 ± 282.47	87.9	1595
2007	2.08 ± 1.11	163.13 ± 648.73	1246.21 ± 600.39	78.6	1562
2008	6.37 ± 1.89	127.10 ± 1074.18	1075.66 ± 849.65	19.9	1375
2009	5.72 ± 1.46	217.28 ± 1151.73	1046.29 ± 784.39	38.0	2029
2010	4.13 ± 1.00	123.39 ± 437.87	266.08 ± 309.65	29.9	2558
2011	3.30 ± 1.07	232.58 ± 487.53	759.81 ± 482.19	70.4	2360
2012	1.31 ± 0.41	119.22 ± 118.53	166.84 ± 108.47	91.2	1311

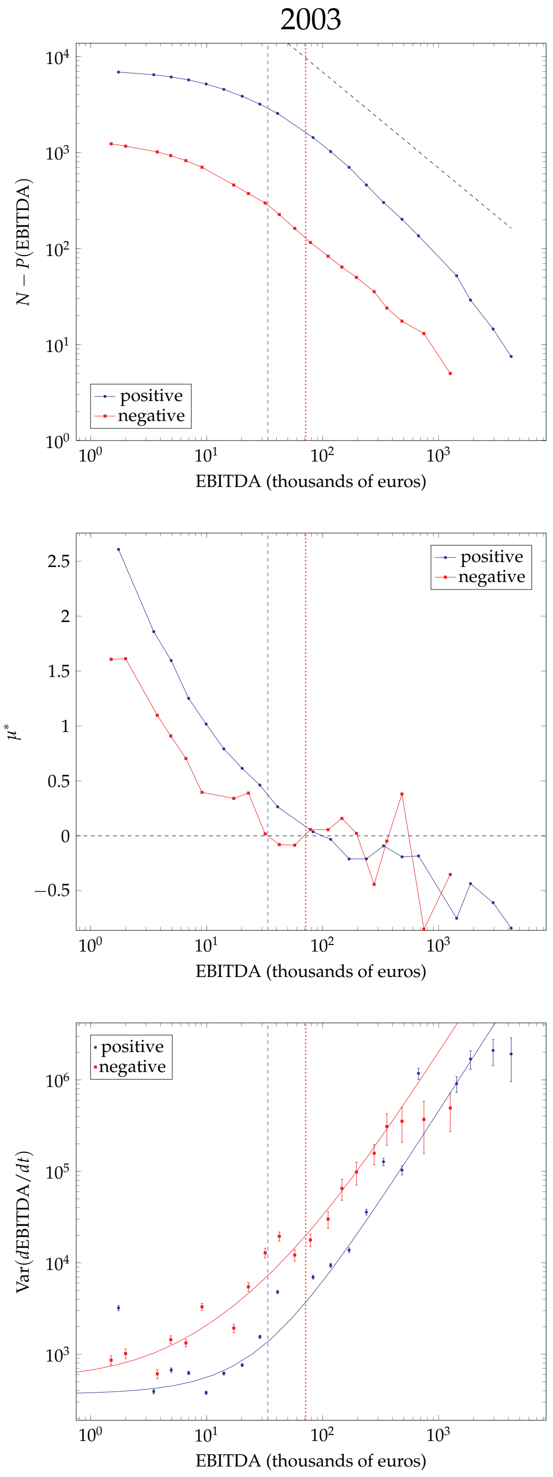

Figure 111: **Extremadura 2003: Rank plot, chemical potential and variance.**
Positive EBITDA: 7186 firms.
$T_1 = 0.44 \pm 0.12$, $T_{1/2} = 14.92 \pm 9.76$, and $T_0 = 364.16 \pm 87.07$
Negative EBITDA: 1437 firms.
$T_1 = 1.89 \pm 0.85$, $T_{1/2} = 135.01 \pm 48.99$, and $T_0 = 533.65 \pm 202.59$.
Total active firms 8623, total created firms 1070, and total destroyed firms 318

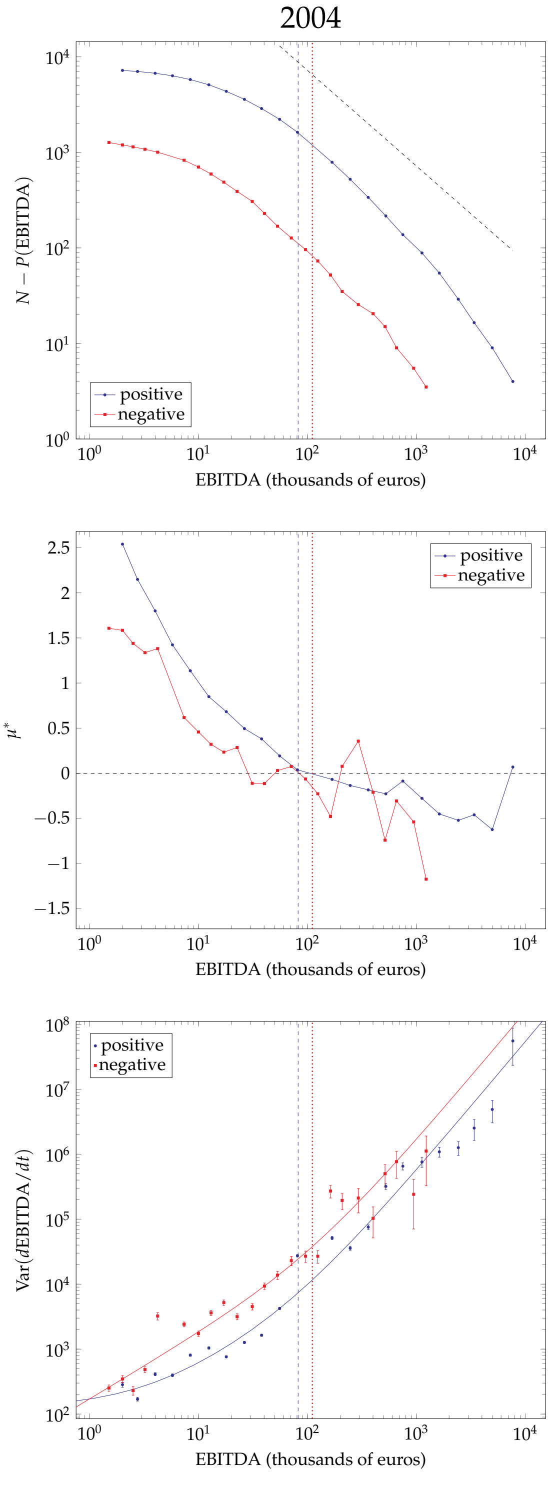

Figure 112: **Extremadura 2004: Rank plot, chemical potential and variance.**
Positive EBITDA: 7582 firms.
T_1 = 0.54 ± 0.19, $T_{1/2}$= 44.04 ± 12.30, and T_0 =126.42 ± 56.07
Negative EBITDA: 1472 firms.
T_1 = 1.54 ± 0.83, $T_{1/2}$= 170.43 ± 45.98, and T_0 =0.00 ± 108.91.
Total active firms 9054, total created firms 1082, and total destroyed firms 589

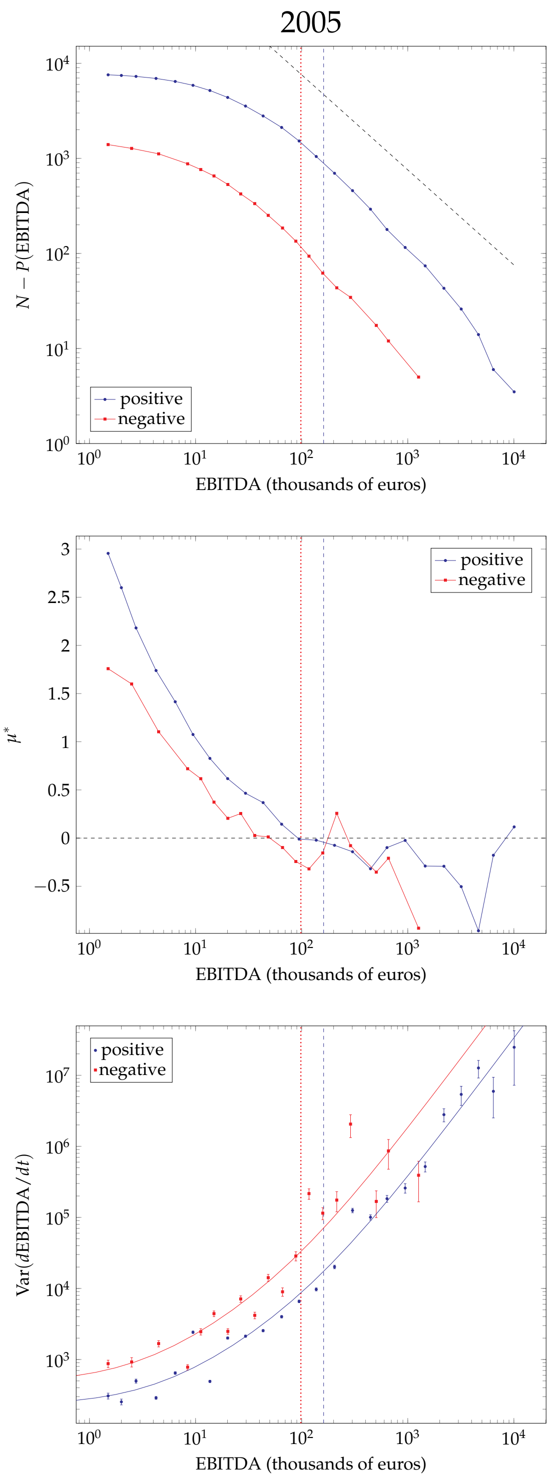

Figure 113: **Extremadura 2005: Rank plot, chemical potential and variance.**
Positive EBITDA: 7825 firms.
$T_1 = 0.33 \pm 0.06$, $T_{1/2} = 53.19 \pm 15.90$, and $T_0 = 226.61 \pm 102.99$
Negative EBITDA: 1634 firms.
$T_1 = 1.68 \pm 0.97$, $T_{1/2} = 165.07 \pm 107.24$, and $T_0 = 468.30 \pm 621.78$.
Total active firms 9459, total created firms 1076, and total destroyed firms 678

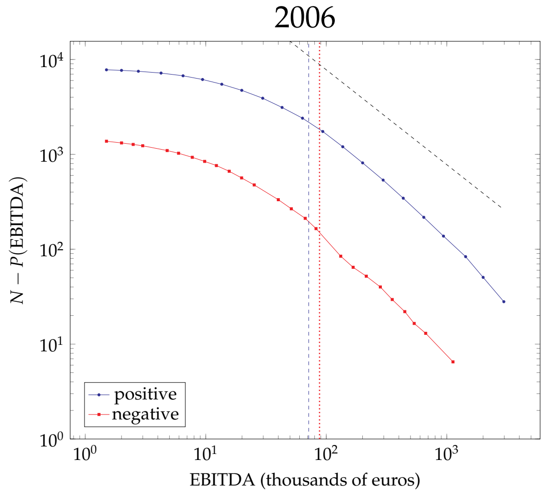

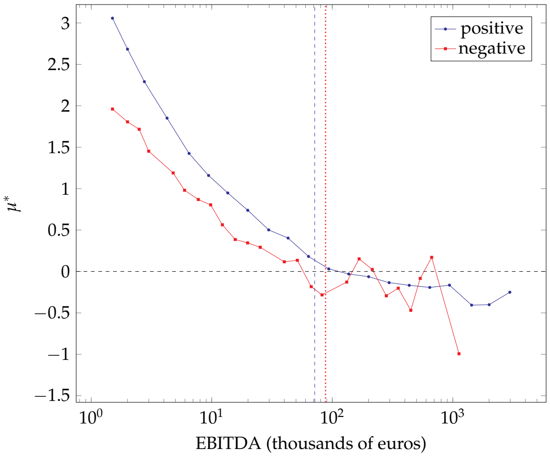

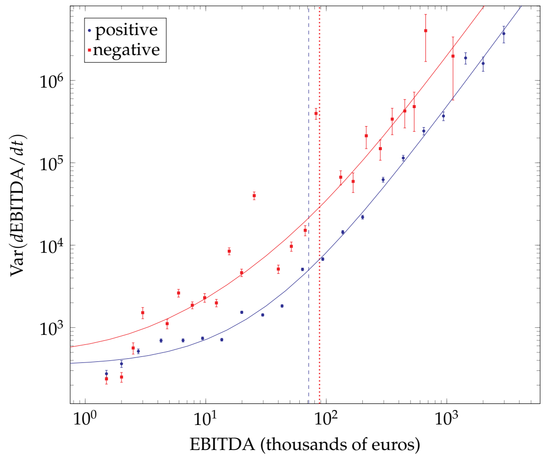

Figure 114: **Extremadura 2006: Rank plot, chemical potential and variance.**
Positive EBITDA: 8027 firms.
T_1 = 0.45 ± 0.10, $T_{1/2}$= 32.49 ± 7.44, and T_0 =343.62 ± 47.17
Negative EBITDA: 1595 firms.
T_1 = 1.84 ± 0.72, $T_{1/2}$= 161.84 ± 53.21, and T_0 =456.68 ± 282.47.
Total active firms 9622, total created firms 1491, and total destroyed firms 917

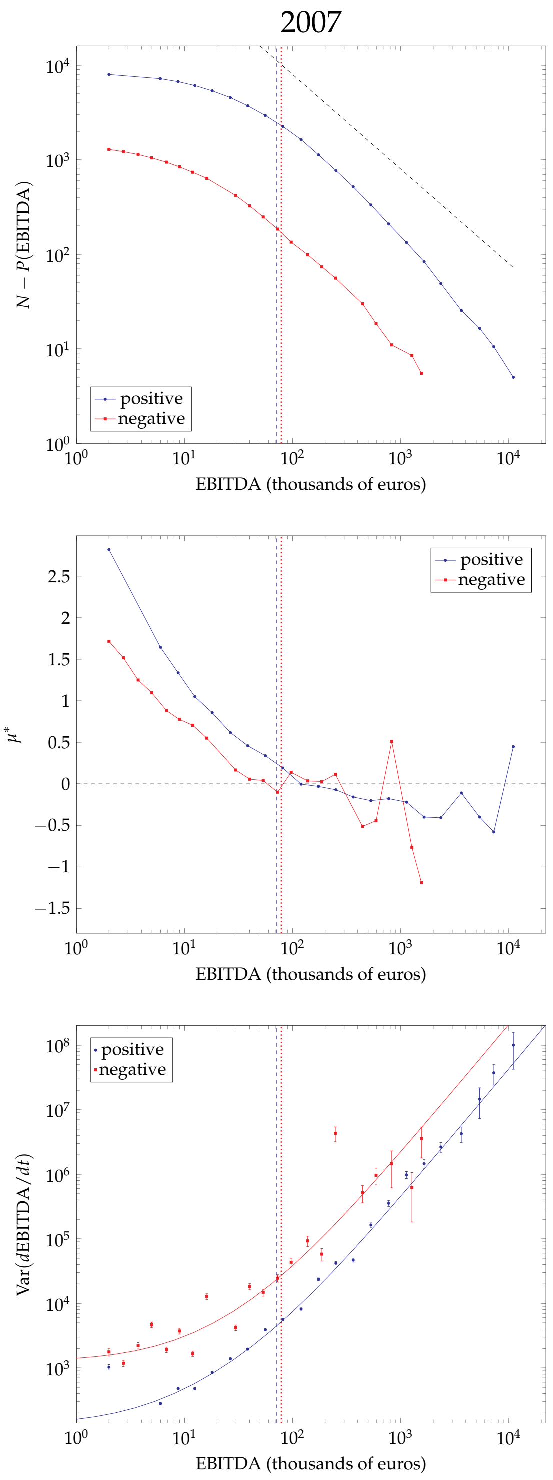

Figure 115: **Extremadura 2007: Rank plot, chemical potential and variance.**
Positive EBITDA: 8285 firms.
T_1 = 0.43 ± 0.07, $T_{1/2}$= 30.41 ± 6.85, and T_0 =128.97 ± 72.52
Negative EBITDA: 1562 firms.
T_1 = 2.08 ± 1.11, $T_{1/2}$= 163.13 ± 97.37, and T_0 =1246.21 ± 600.39.
Total active firms 9847, total created firms 697, and total destroyed firms 1267

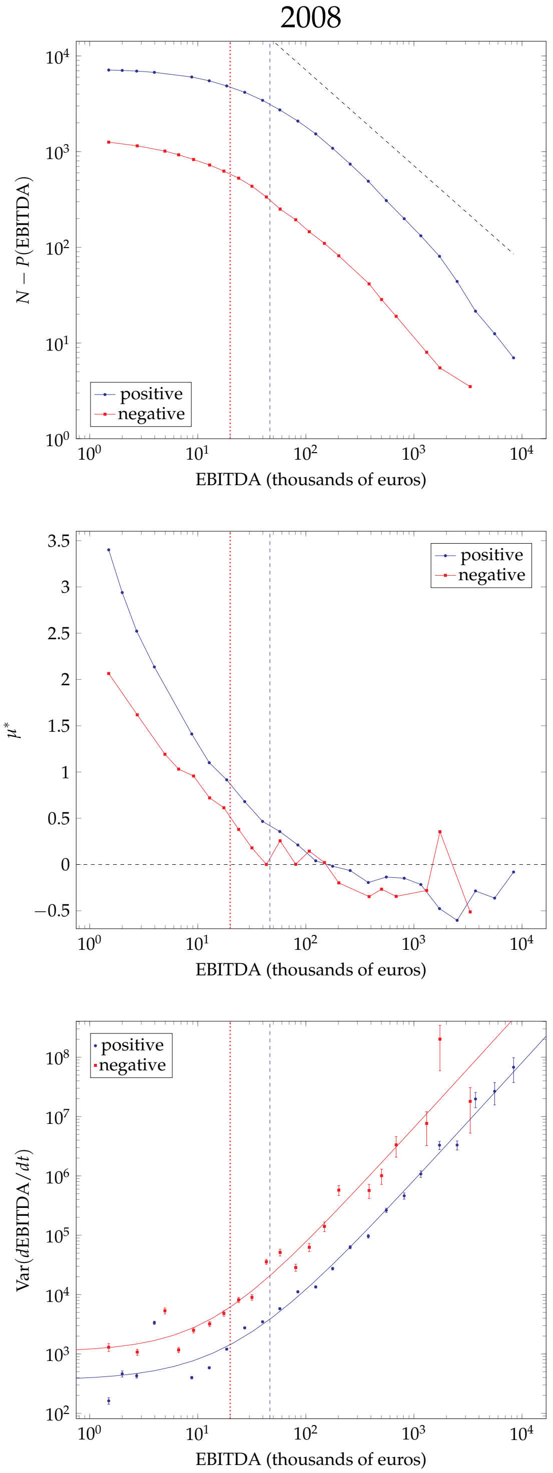

Figure 116: **Extremadura 2008: Rank plot, chemical potential and variance.**
Positive EBITDA: 7281 firms.
$T_1 = 0.81 \pm 0.15$, $T_{1/2}$= 37.50 ± 20.96, and T_0 =359.75 ± 144.80
Negative EBITDA: 1375 firms.
$T_1 = 6.37 \pm 1.89$, $T_{1/2}$= 127.10 ± 139.09, and T_0 =1075.66 ± 849.65.
Total active firms 8656, total created firms 2122, and total destroyed firms 1948

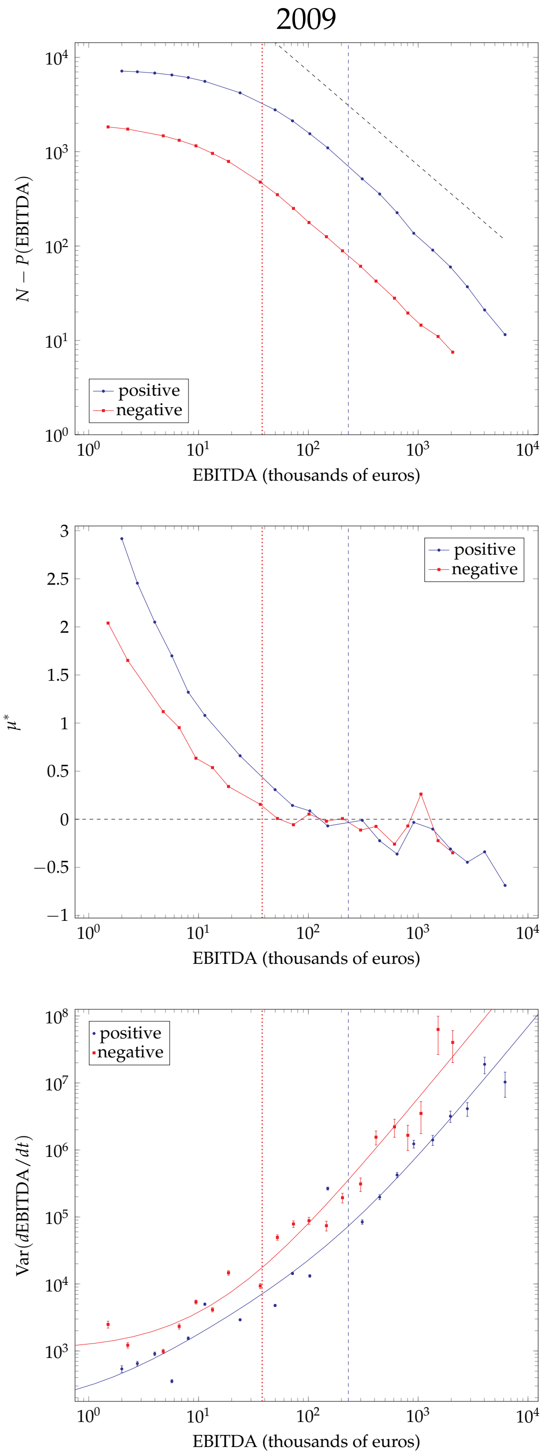

Figure 117: **Extremadura 2009: Rank plot, chemical potential and variance**.
Positive EBITDA: 7457 firms.
T_1 = 0.68 ± 0.21, $T_{1/2}$= 157.34 ± 61.34, and T_0 =144.96 ± 300.80
Negative EBITDA: 2029 firms.
T_1 = 5.72 ± 1.46, $T_{1/2}$= 217.28 ± 146.12, and T_0 =1046.29 ± 784.39.
Total active firms 9486, total created firms 1634, and total destroyed firms 732

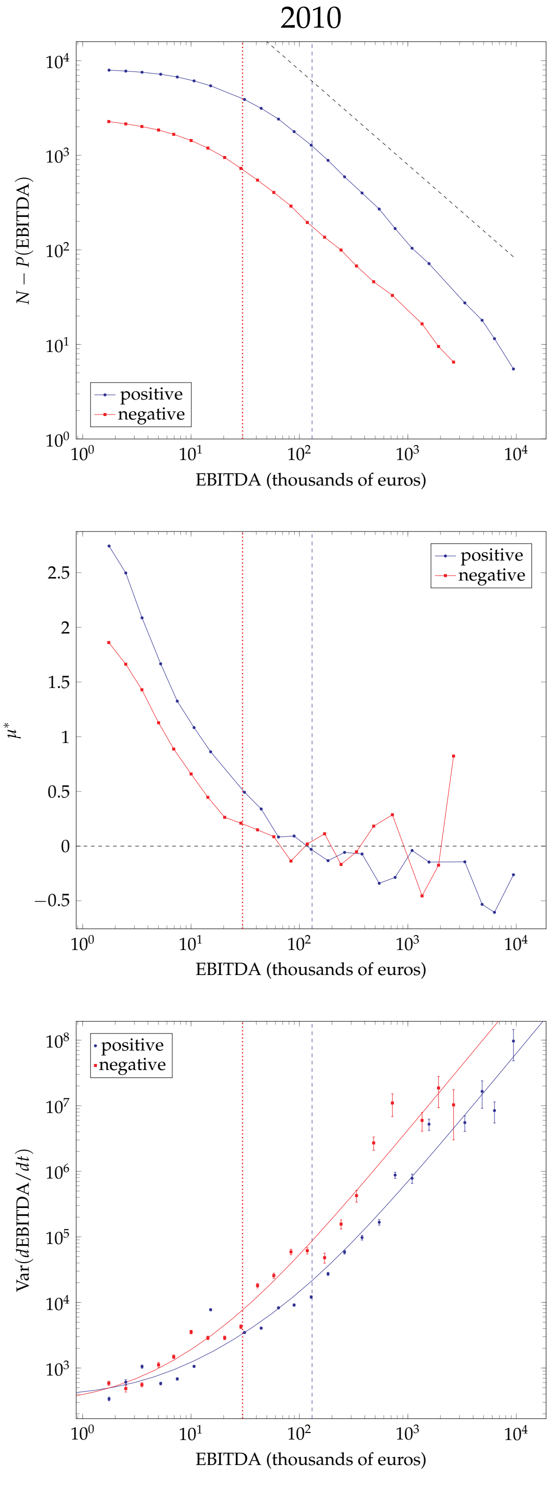

Figure 118: **Extremadura 2010: Rank plot, chemical potential and variance.**
Positive EBITDA: 8285 firms.
$T_1 = 0.62 \pm 0.14$, $T_{1/2} = 80.84 \pm 31.60$, and $T_0 = 351.66 \pm 209.75$
Negative EBITDA: 2558 firms.
$T_1 = 4.13 \pm 1.00$, $T_{1/2} = 123.39 \pm 73.74$, and $T_0 = 266.08 \pm 309.65$.
Total active firms 10843, total created firms 1175, and total destroyed firms 805

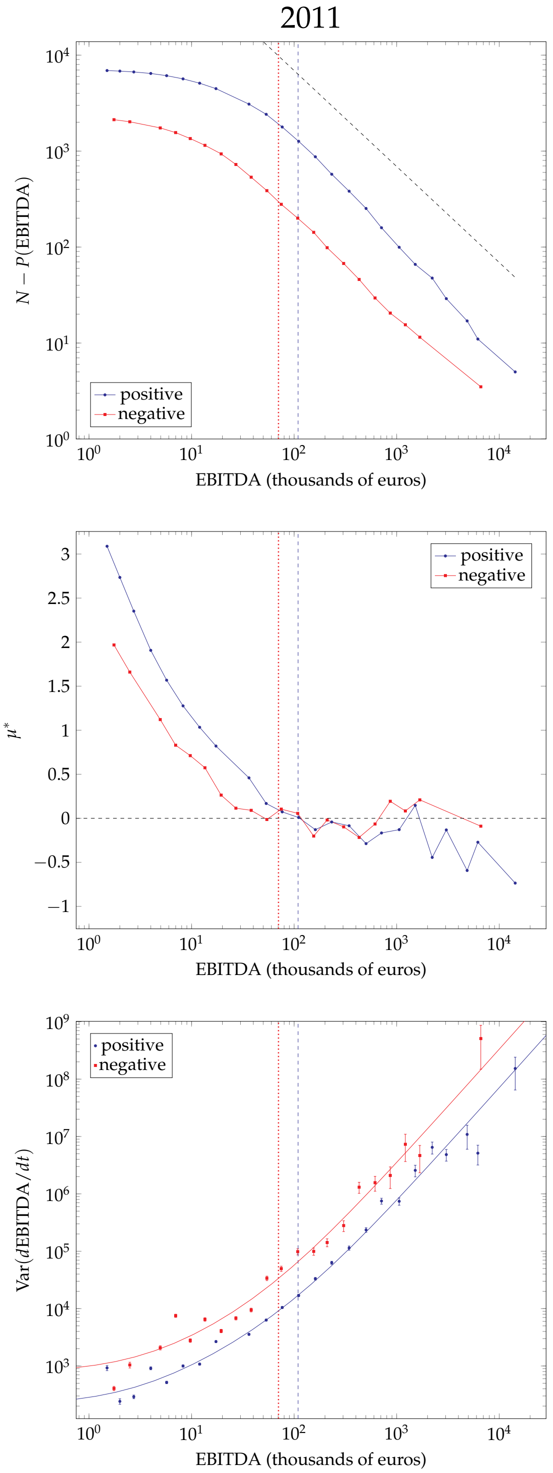

Figure 119: **Extremadura 2011: Rank plot, chemical potential and variance.**
Positive EBITDA: 7137 firms.
$T_1 = 0.70 \pm 0.23$, $T_{1/2}$= 76.80 ± 20.12, and T_0 =205.87 ± 82.07
Negative EBITDA: 2360 firms.
$T_1 = 3.30 \pm 1.07$, $T_{1/2}$= 232.58 ± 79.56, and T_0 =759.81 ± 482.19.
Total active firms 9497, total created firms 486, and total destroyed firms 1584

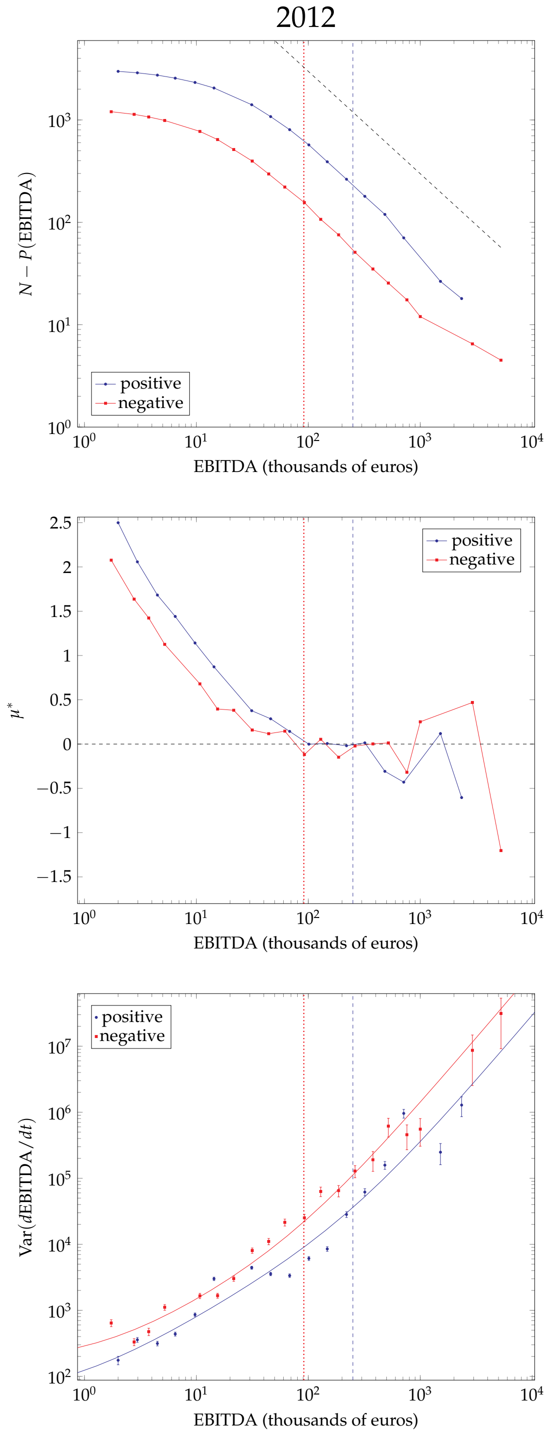

Figure 120: **Extremadura 2012: Rank plot, chemical potential and variance.**
Positive EBITDA: 3135 firms.
$T_1 = 0.29 \pm 0.22$, $T_{1/2}$= 72.10 ± 20.57, and T_0 =50.72 ± 77.34
Negative EBITDA: 1311 firms.
$T_1 = 1.31 \pm 0.41$, $T_{1/2}$= 119.22 ± 29.27, and T_0 =166.84 ± 108.47.
Total active firms 4446, total created firms 86, and total destroyed firms 6571

Galicia

Tables of Temperatures

Positive EBITDA

Year	T_1	$T_{1/2}$	T_0	$T_{1/2}/T_1$	Num. Firms
2003	0.20 ± 0.05	69.89 ± 25.77	315.68 ± 242.48	354.9	24528
2004	0.43 ± 0.08	31.69 ± 6.98	118.56 ± 65.73	73.4	25688
2005	0.48 ± 0.06	25.87 ± 4.66	197.16 ± 45.44	53.8	27216
2006	0.31 ± 0.07	82.04 ± 30.64	371.19 ± 232.66	264.1	29185
2007	0.51 ± 0.06	58.58 ± 14.35	78.13 ± 66.90	114.9	35512
2008	0.77 ± 0.10	57.59 ± 25.10	869.88 ± 285.95	74.6	34380
2009	0.67 ± 0.10	113.71 ± 30.23	180.44 ± 158.94	169.8	32216
2010	0.44 ± 0.05	70.96 ± 15.72	272.35 ± 109.91	161.9	33708
2011	0.52 ± 0.09	95.88 ± 37.32	725.39 ± 390.32	183.6	29566
2012	0.33 ± 0.10	86.17 ± 34.72	150.55 ± 218.81	263.5	20251

Negative EBITDA

Year	T_1	$T_{1/2}$	T_0	$T_{1/2}/T_1$	Num. Firms
2003	2.11 ± 0.45	193.50 ± 236.69	0.03 ± 156.16	91.6	5344
2004	2.26 ± 0.80	179.02 ± 235.27	218.43 ± 190.21	79.4	5509
2005	2.37 ± 0.74	132.82 ± 143.82	492.93 ± 203.75	56.1	5926
2006	2.89 ± 0.66	136.66 ± 346.00	418.19 ± 322.52	47.3	6740
2007	1.94 ± 0.43	276.75 ± 400.46	0.12 ± 248.77	142.4	9015
2008	3.26 ± 0.84	221.47 ± 800.67	349.11 ± 494.66	68.0	9000
2009	4.21 ± 0.52	285.24 ± 335.63	107.84 ± 211.33	67.7	10933
2010	2.08 ± 0.43	241.71 ± 480.45	31.31 ± 278.67	116.1	13500
2011	3.51 ± 0.56	263.11 ± 451.63	0.15 ± 248.74	74.9	12371
2012	2.67 ± 0.36	53.60 ± 148.17	549.50 ± 227.51	20.1	10958

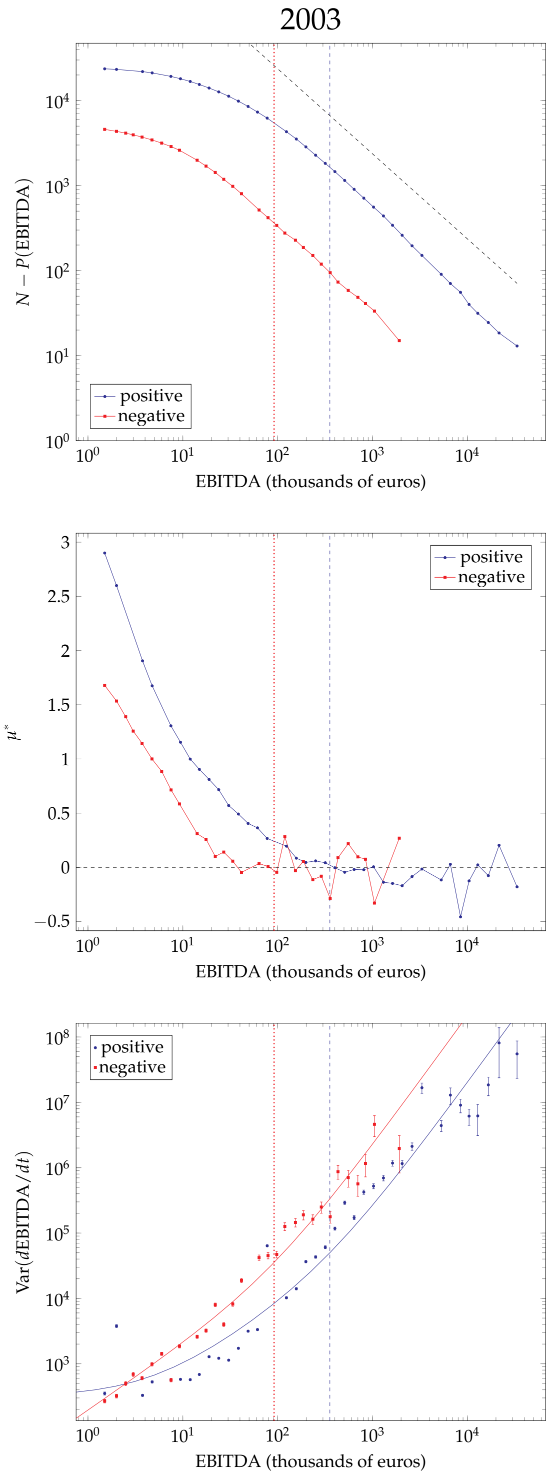

Figure 121: **Galicia 2003: Rank plot, chemical potential and variance.**
Positive EBITDA: 24528 firms.
$T_1 = 0.20 \pm 0.05$, $T_{1/2} = 69.89 \pm 25.77$, and $T_0 = 315.68 \pm 242.48$
Negative EBITDA: 5344 firms.
$T_1 = 2.11 \pm 0.45$, $T_{1/2} = 193.50 \pm 47.73$, and $T_0 = 0.03 \pm 156.16$.
Total active firms 29872, total created firms 4595, and total destroyed firms 2639

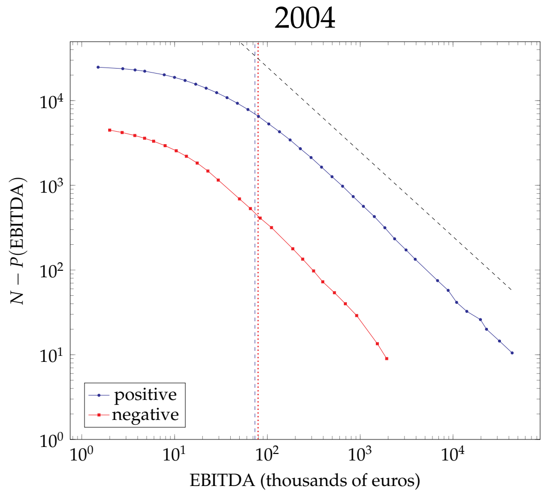

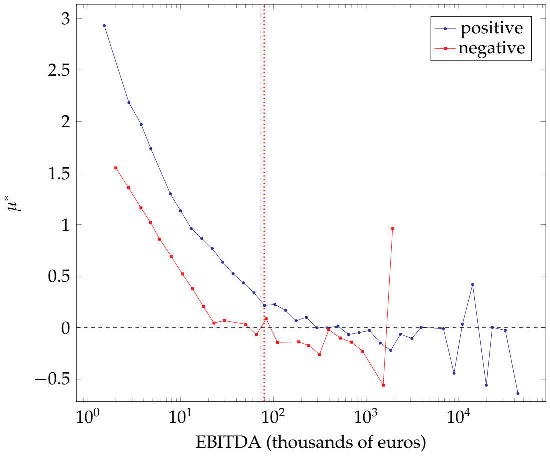

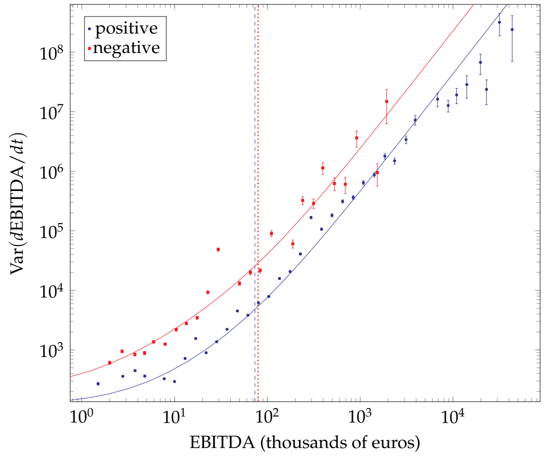

Figure 122: **Galicia 2004: Rank plot, chemical potential and variance.**
Positive EBITDA: 25688 firms.
$T_1 = 0.43 \pm 0.08$, $T_{1/2} = 31.69 \pm 6.98$, and $T_0 = 118.56 \pm 65.73$
Negative EBITDA: 5509 firms.
$T_1 = 2.26 \pm 0.80$, $T_{1/2} = 179.02 \pm 47.53$, and $T_0 = 218.43 \pm 190.21$.
Total active firms 31197, total created firms 5572, and total destroyed firms 3251

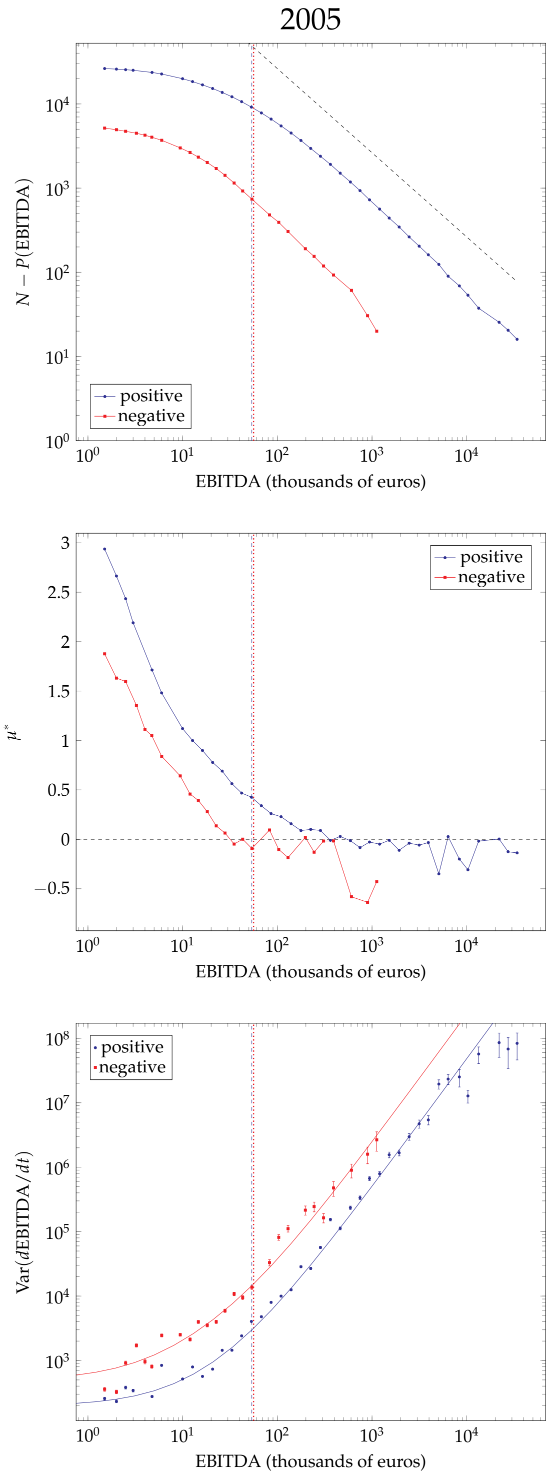

Figure 123: **Galicia 2005: Rank plot, chemical potential and variance**.
Positive EBITDA: 27216 firms.
T_1 = 0.48 ± 0.06, $T_{1/2}$= 25.87 ± 4.66, and T_0 =197.16 ± 45.44
Negative EBITDA: 5926 firms.
T_1 = 2.37 ± 0.74, $T_{1/2}$= 132.82 ± 33.56, and T_0 =492.93 ± 203.75.
Total active firms 33142, total created firms 4992, and total destroyed firms 3632

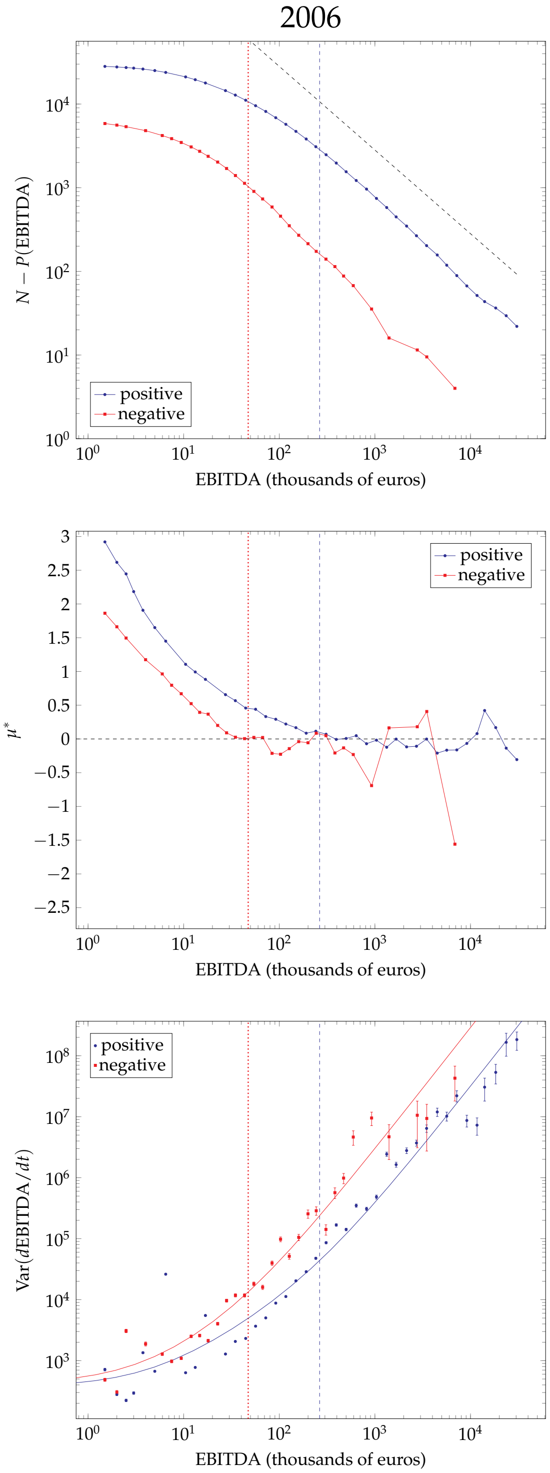

Figure 124: **Galicia 2006: Rank plot, chemical potential and variance**.
Positive EBITDA: 29185 firms.
T_1 = 0.31 ± 0.07, $T_{1/2}$= 82.04 ± 30.64, and T_0 =371.19 ± 232.66
Negative EBITDA: 6740 firms.
T_1 = 2.89 ± 0.66, $T_{1/2}$= 136.66 ± 62.43, and T_0 =418.19 ± 322.52.
Total active firms 35925, total created firms 12428, and total destroyed firms 2145

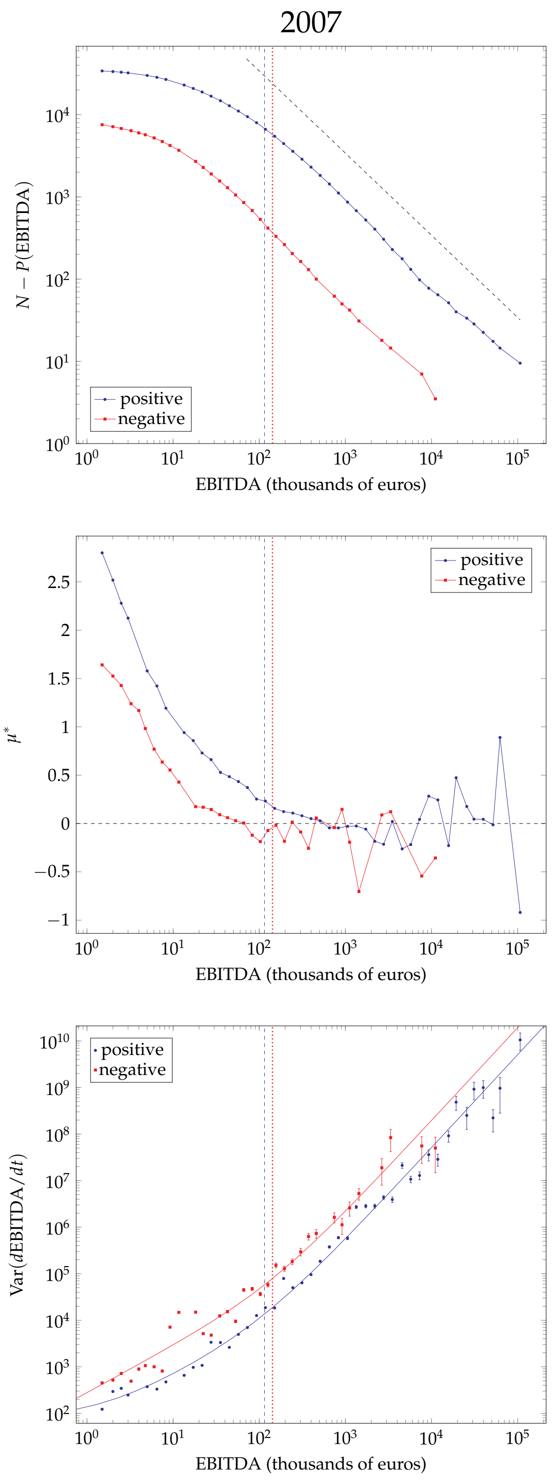

Figure 125: **Galicia 2007: Rank plot, chemical potential and variance.**
Positive EBITDA: 35512 firms.
$T_1 = 0.51 \pm 0.06$, $T_{1/2} = 58.58 \pm 14.35$, and $T_0 = 78.13 \pm 66.90$
Negative EBITDA: 9015 firms.
$T_1 = 1.94 \pm 0.43$, $T_{1/2} = 276.75 \pm 69.23$, and $T_0 = 0.12 \pm 248.77$.
Total active firms 44527, total created firms 4646, and total destroyed firms 2930

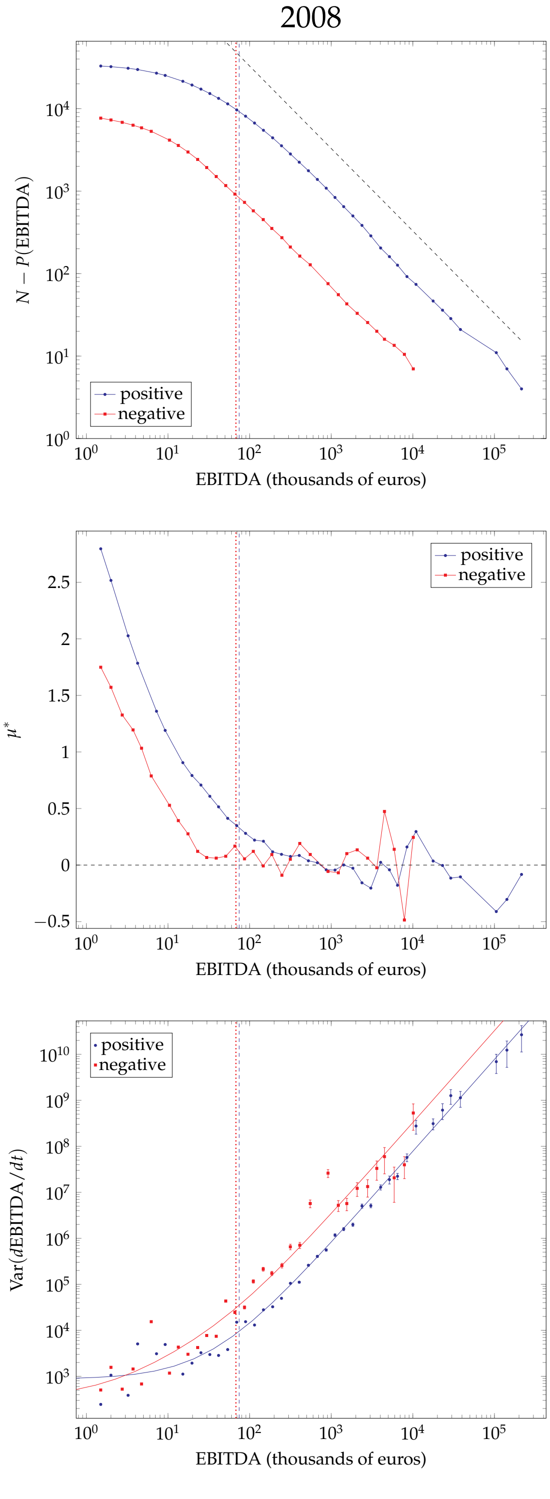

Figure 126: **Galicia 2008: Rank plot, chemical potential and variance**.
Positive EBITDA: 34380 firms.
T_1 = 0.77 ± 0.10, $T_{1/2}$= 57.59 ± 25.10, and T_0 =869.88 ± 285.95
Negative EBITDA: 9000 firms.
T_1 = 3.26 ± 0.84, $T_{1/2}$= 221.47 ± 112.99, and T_0 =349.11 ± 494.66.
Total active firms 43380, total created firms 5462, and total destroyed firms 6155

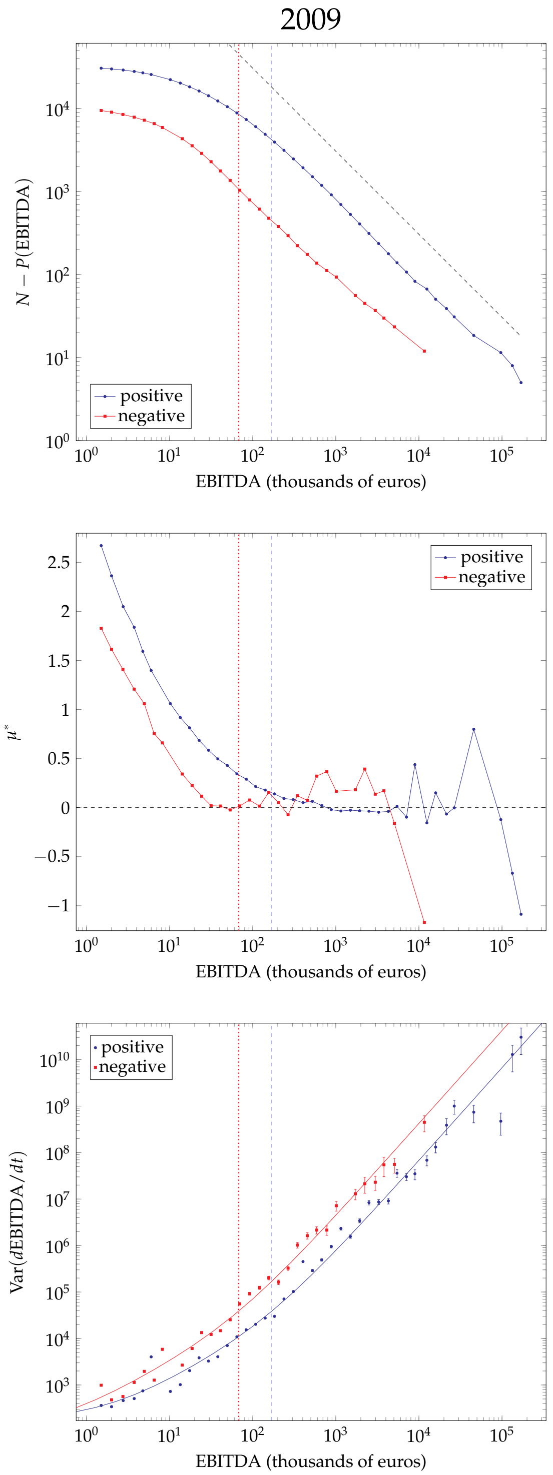

Figure 127: **Galicia 2009: Rank plot, chemical potential and variance.**
Positive EBITDA: 32216 firms.
$T_1 = 0.67 \pm 0.10$, $T_{1/2}$= 113.71 ± 30.23, and T_0 =180.44 ± 158.94
Negative EBITDA: 10933 firms.
$T_1 = 4.21 \pm 0.52$, $T_{1/2}$= 285.24 ± 61.10, and T_0 =107.84 ± 211.33.
Total active firms 43149, total created firms 5353, and total destroyed firms 3229

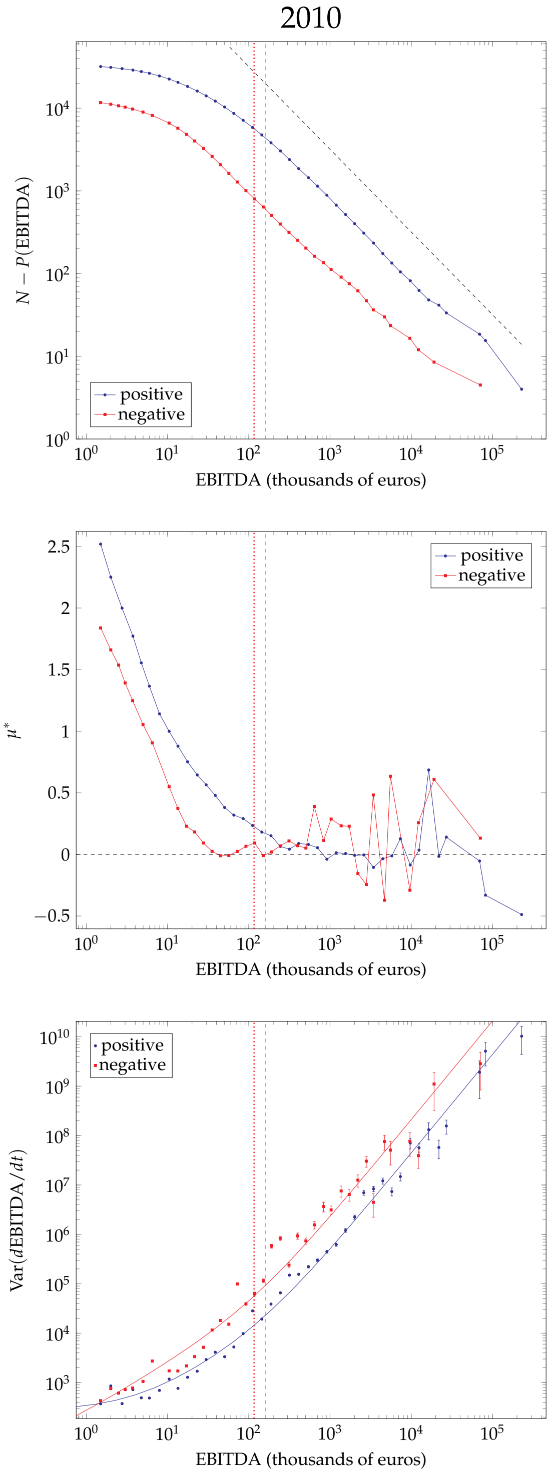

Figure 128: **Galicia 2010: Rank plot, chemical potential and variance.**
Positive EBITDA: 33708 firms.
$T_1 = 0.44 \pm 0.05$, $T_{1/2} = 70.96 \pm 15.72$, and $T_0 = 272.35 \pm 109.91$
Negative EBITDA: 13500 firms.
$T_1 = 2.08 \pm 0.43$, $T_{1/2} = 241.71 \pm 78.74$, and $T_0 = 31.31 \pm 278.67$.
Total active firms 47208, total created firms 3940, and total destroyed firms 3347

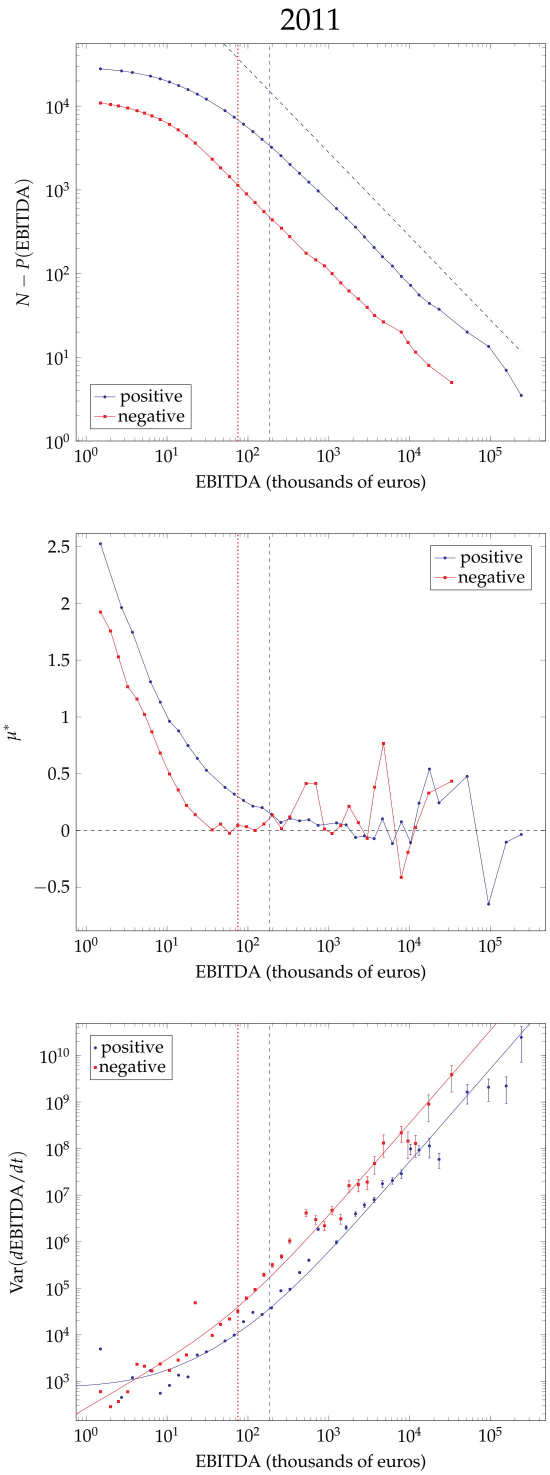

Figure 129: **Galicia 2011: Rank plot, chemical potential and variance.**
Positive EBITDA: 29566 firms.
T_1 = 0.52 ± 0.09, $T_{1/2}$= 95.88 ± 37.32, and T_0 =725.39 ± 390.32
Negative EBITDA: 12371 firms.
T_1 = 3.51 ± 0.56, $T_{1/2}$= 263.11 ± 75.37, and T_0 =0.15 ± 248.74.
Total active firms 41937, total created firms 2183, and total destroyed firms 6564

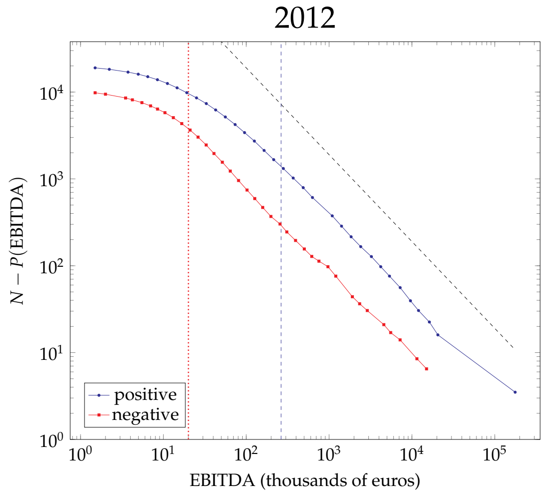

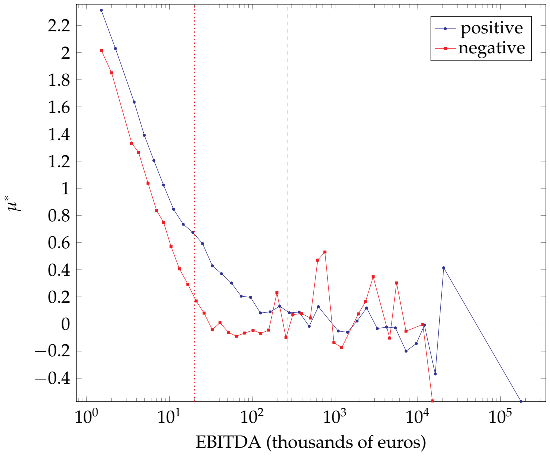

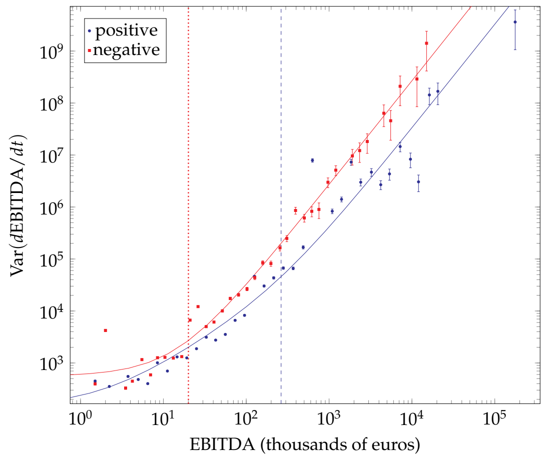

Figure 130: **Galicia 2012: Rank plot, chemical potential and variance.**
Positive EBITDA: 20251 firms.
$T_1 = 0.33 \pm 0.10$, $T_{1/2} = 86.17 \pm 34.72$, and $T_0 = 150.55 \pm 218.81$
Negative EBITDA: 10958 firms.
$T_1 = 2.67 \pm 0.36$, $T_{1/2} = 53.60 \pm 34.27$, and $T_0 = 549.50 \pm 227.51$.
Total active firms 31209, total created firms 747, and total destroyed firms 16415

La Rioja

Tables of Temperatures

Positive EBITDA

Year	T_1	$T_{1/2}$	T_0	$T_{1/2}/T_1$	Num. Firms
2003	0.24 ± 0.04	24.45 ± 5.62	154.81 ± 67.97	102.3	3067
2004	0.43 ± 0.08	16.09 ± 8.15	300.94 ± 115.71	37.3	3247
2005	0.52 ± 0.11	44.16 ± 25.37	505.63 ± 242.45	84.3	3571
2006	0.55 ± 0.09	62.16 ± 19.13	89.19 ± 85.17	113.0	3696
2007	0.45 ± 0.10	51.87 ± 25.13	339.24 ± 203.75	114.8	3505
2008	0.56 ± 0.11	44.52 ± 13.29	526.28 ± 192.17	79.4	3068
2009	0.82 ± 0.18	78.97 ± 47.63	1761.79 ± 627.01	96.7	3120
2010	0.40 ± 0.27	67.77 ± 43.30	856.31 ± 451.11	168.3	3395
2011	0.22 ± 0.08	170.10 ± 50.40	494.38 ± 406.84	779.5	3048
2012	0.24 ± 0.07	61.82 ± 10.45	257.44 ± 146.59	256.9	1768

Negative EBITDA

Year	T_1	$T_{1/2}$	T_0	$T_{1/2}/T_1$	Num. Firms
2003	0.40 ± 0.40	109.84 ± 171.22	166.04 ± 212.49	274.2	583
2004	1.27 ± 0.71	62.86 ± 161.17	592.68 ± 244.70	49.5	605
2005	5.05 ± 3.15	222.71 ± 2003.79	653.03 ± 924.12	44.1	739
2006	1.78 ± 1.13	294.05 ± 1305.96	0.00 ± 829.28	164.9	691
2007	2.84 ± 0.70	175.02 ± 292.19	81.44 ± 236.25	61.5	603
2008	1.24 ± 0.70	66.77 ± 281.67	1028.34 ± 721.60	53.7	554
2009	5.06 ± 2.66	403.36 ± 2579.00	350.30 ± 906.02	79.7	891
2010	0.48 ± 0.26	176.27 ± 268.16	834.30 ± 425.40	365.2	1273
2011	3.74 ± 1.02	316.52 ± 796.47	160.73 ± 448.16	84.6	1140
2012	2.22 ± 0.53	101.79 ± 326.45	789.15 ± 404.82	45.9	739

2003

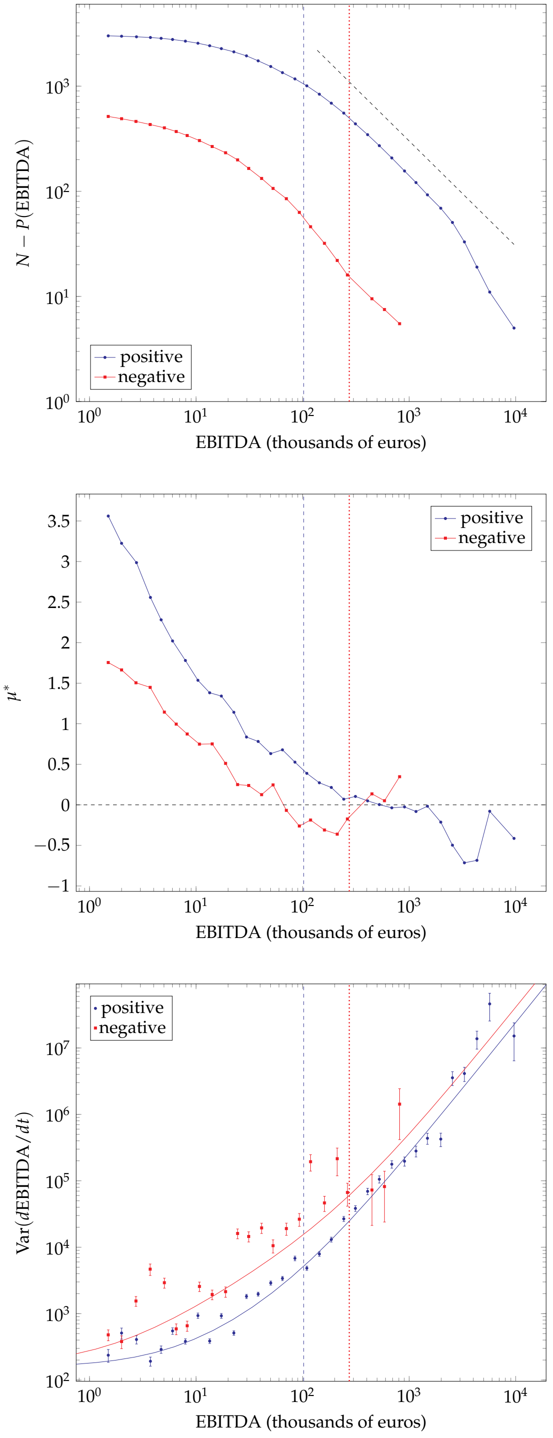

Figure 131: **La Rioja 2003: Rank plot, chemical potential and variance**.
Positive EBITDA: 3067 firms.
T_1 = 0.24 ± 0.04, $T_{1/2}$= 24.45 ± 5.62, and T_0 =154.81 ± 67.97
Negative EBITDA: 583 firms.
T_1 = 0.40 ± 0.40, $T_{1/2}$= 109.84 ± 37.96, and T_0 =166.04 ± 212.49.
Total active firms 3650, total created firms 579, and total destroyed firms 123

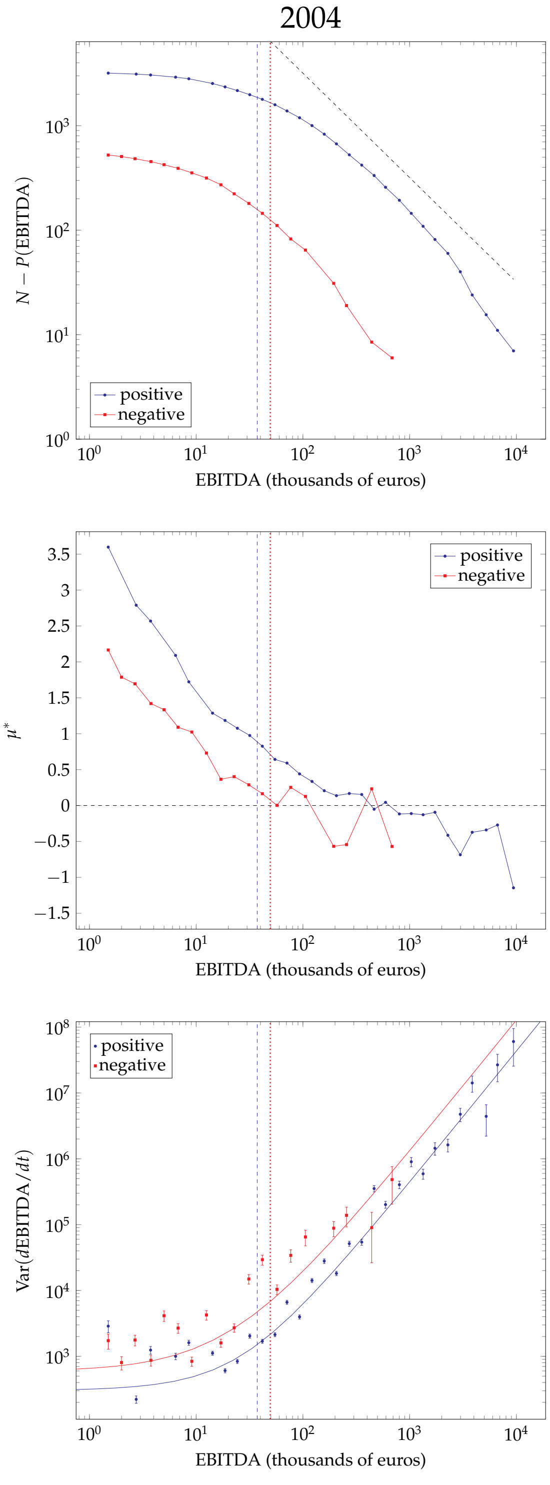

Figure 132: **La Rioja 2004: Rank plot, chemical potential and variance.**
Positive EBITDA: 3247 firms.
T_1 = 0.43 ± 0.08, $T_{1/2}$= 16.09 ± 8.15, and T_0 =300.94 ± 115.71
Negative EBITDA: 605 firms.
T_1 = 1.27 ± 0.71, $T_{1/2}$= 62.86 ± 36.37, and T_0 =592.68 ± 244.70.
Total active firms 3852, total created firms 790, and total destroyed firms 372

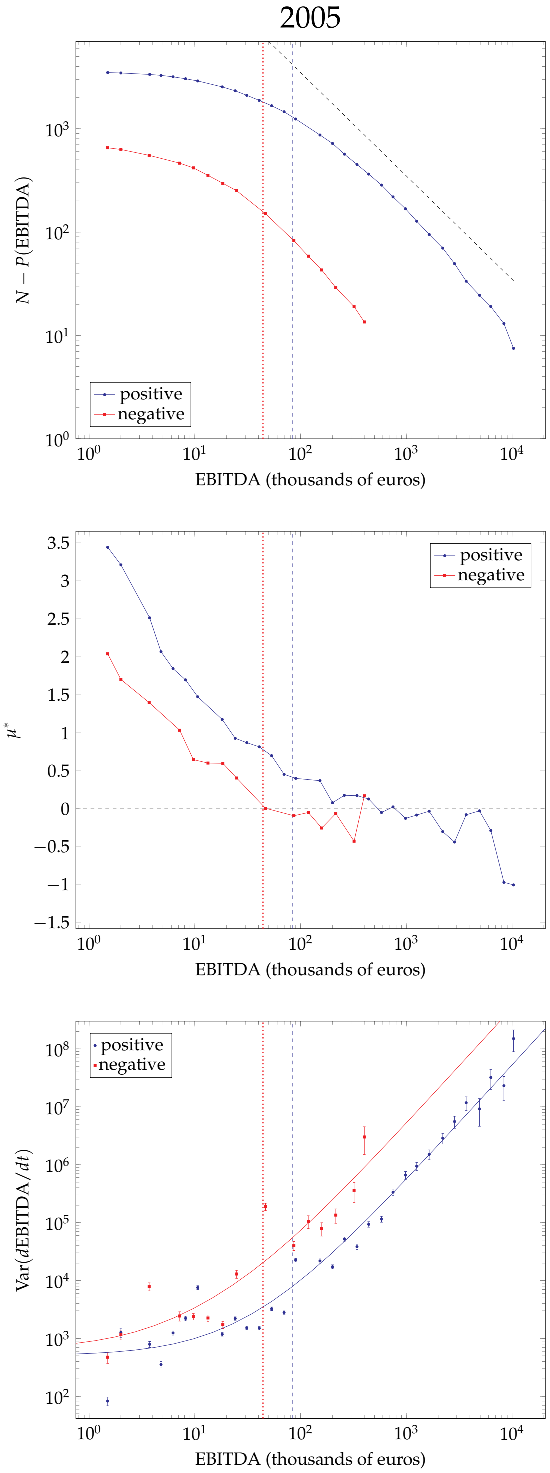

Figure 133: **La Rioja 2005: Rank plot, chemical potential and variance**.
Positive EBITDA: 3571 firms.
T_1 = 0.52 ± 0.11, $T_{1/2}$= 44.16 ± 25.37, and T_0 =505.63 ± 242.45
Negative EBITDA: 739 firms.
T_1 = 5.05 ± 3.15, $T_{1/2}$= 222.71 ± 216.15, and T_0 =653.03 ± 924.12.
Total active firms 4310, total created firms 589, and total destroyed firms 321

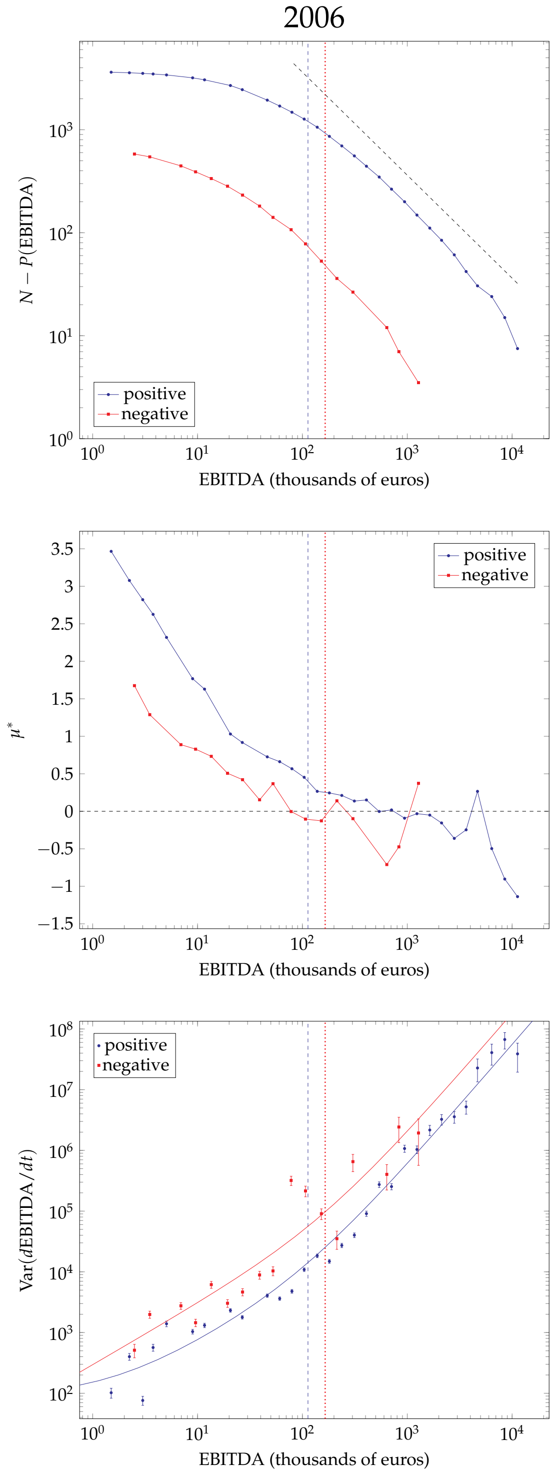

Figure 134: **La Rioja 2006: Rank plot, chemical potential and variance**.
Positive EBITDA: 3696 firms.
T_1 = 0.55 ± 0.09, $T_{1/2}$= 62.16 ± 19.13, and T_0 =89.19 ± 85.17
Negative EBITDA: 691 firms.
T_1 = 1.78 ± 1.13, $T_{1/2}$= 294.05 ± 159.70, and T_0 =0.00 ± 829.28.
Total active firms 4387, total created firms 544, and total destroyed firms 495

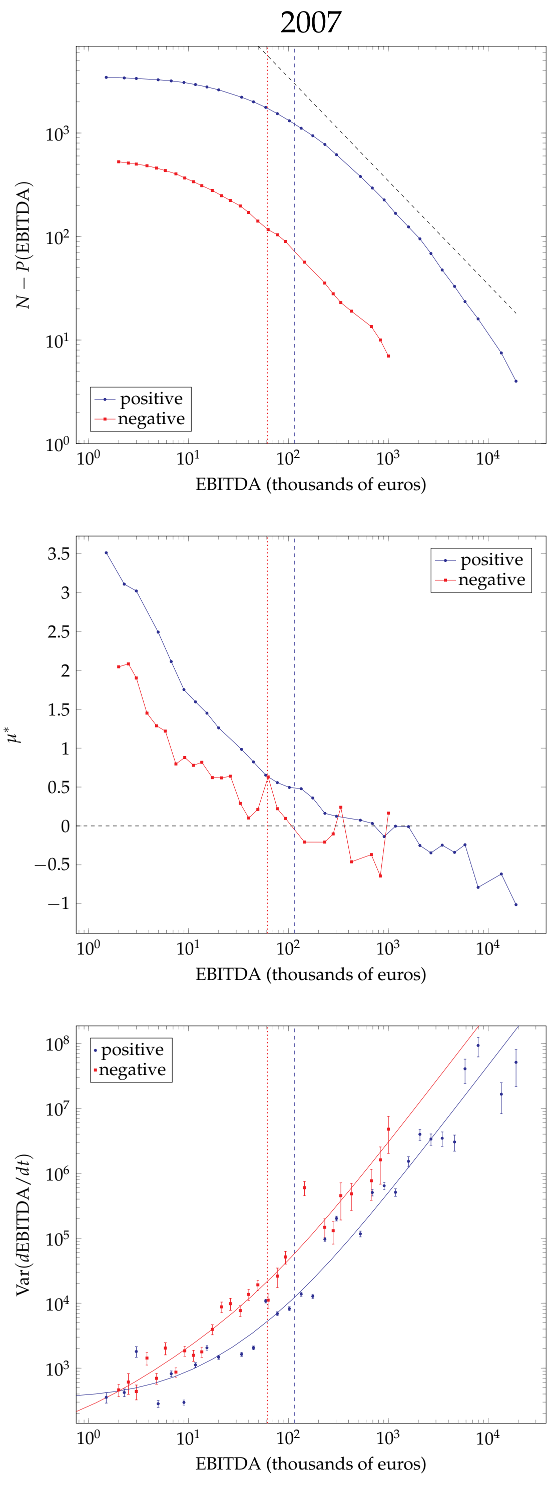

Figure 135: **La Rioja 2007: Rank plot, chemical potential and variance.**
Positive EBITDA: 3505 firms.
T_1 = 0.45 ± 0.10, $T_{1/2}$= 51.87 ± 25.13, and T_0 =339.24 ± 203.75
Negative EBITDA: 603 firms.
T_1 = 2.84 ± 0.70, $T_{1/2}$= 175.02 ± 55.40, and T_0 =81.44 ± 236.25.
Total active firms 4108, total created firms 240, and total destroyed firms 860

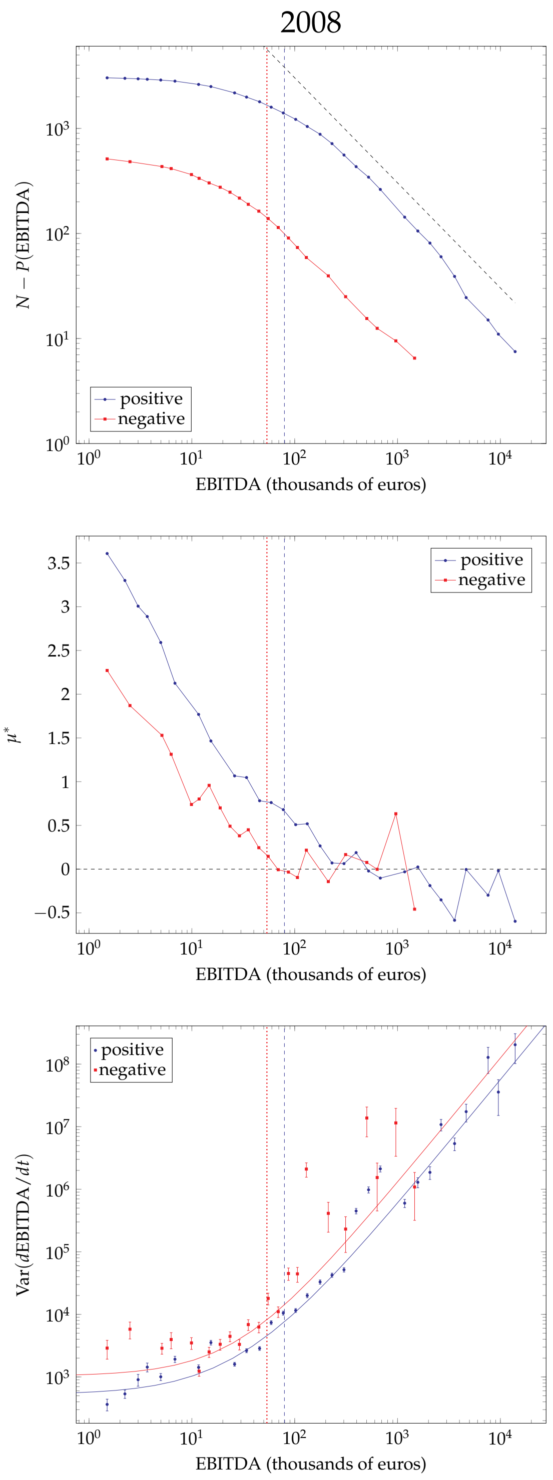

Figure 136: **La Rioja 2008: Rank plot, chemical potential and variance**.
Positive EBITDA: 3068 firms.
$T_1 = 0.56 \pm 0.11$, $T_{1/2} = 44.52 \pm 13.29$, and $T_0 = 526.28 \pm 192.17$
Negative EBITDA: 554 firms.
$T_1 = 1.24 \pm 0.70$, $T_{1/2} = 66.77 \pm 53.98$, and $T_0 = 1028.34 \pm 721.60$.
Total active firms 3622, total created firms 975, and total destroyed firms 766

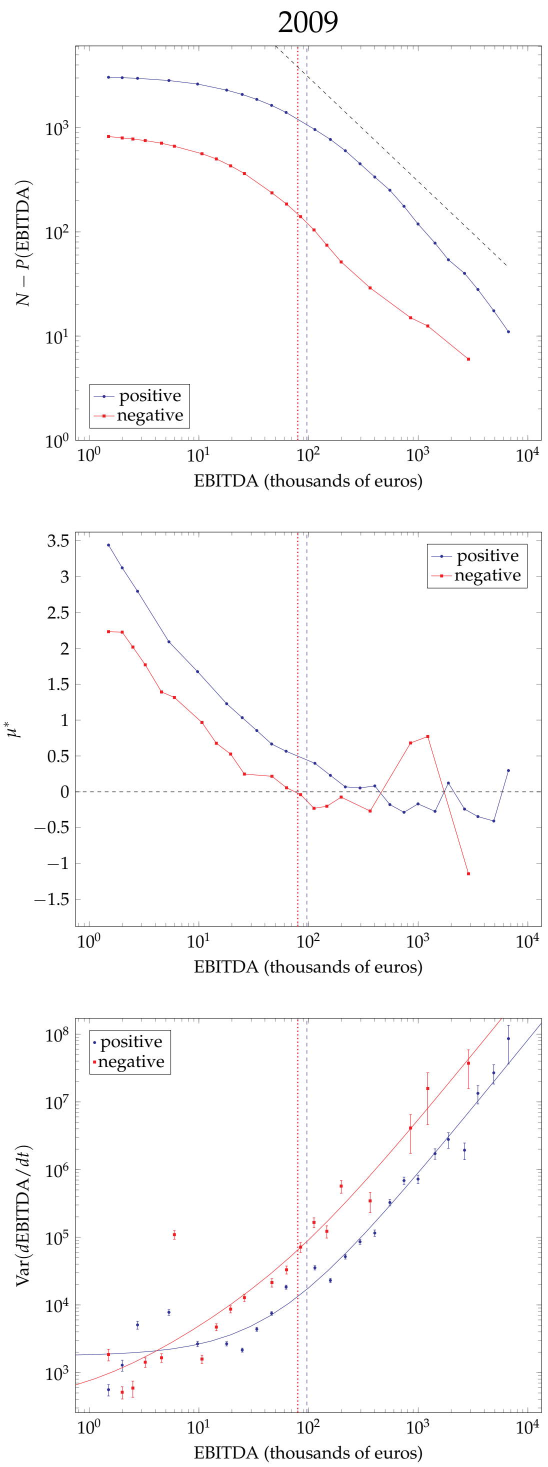

Figure 137: **La Rioja 2009: Rank plot, chemical potential and variance.**
Positive EBITDA: 3120 firms.
T_1 = 0.82 ± 0.18, $T_{1/2}$= 78.97 ± 47.63, and T_0 =1761.79 ± 627.01
Negative EBITDA: 891 firms.
T_1 = 5.06 ± 2.66, $T_{1/2}$= 403.36 ± 258.38, and T_0 =350.30 ± 906.02.
Total active firms 4011, total created firms 787, and total destroyed firms 289

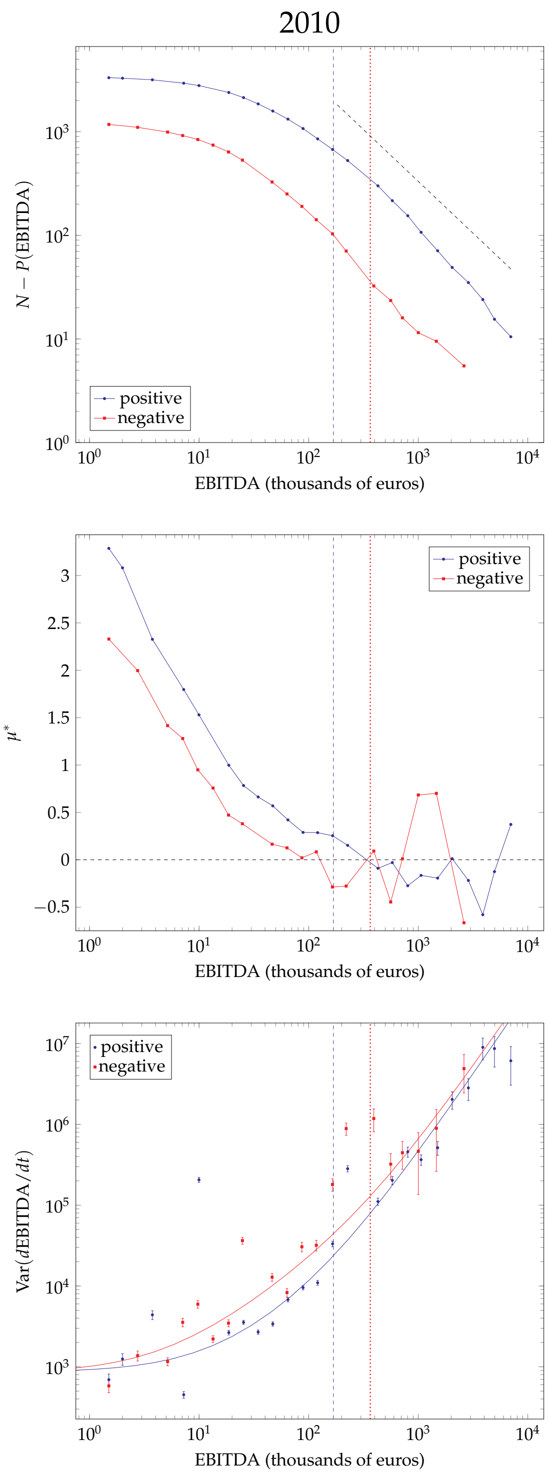

Figure 138: **La Rioja 2010: Rank plot, chemical potential and variance.**
Positive EBITDA: 3395 firms.
$T_1 = 0.40 \pm 0.27$, $T_{1/2}$= 67.77 ± 43.30, and T_0 =856.31 ± 451.11
Negative EBITDA: 1273 firms.
$T_1 = 0.48 \pm 0.26$, $T_{1/2}$= 176.27 ± 52.14, and T_0 =834.30 ± 425.40.
Total active firms 4668, total created firms 537, and total destroyed firms 385

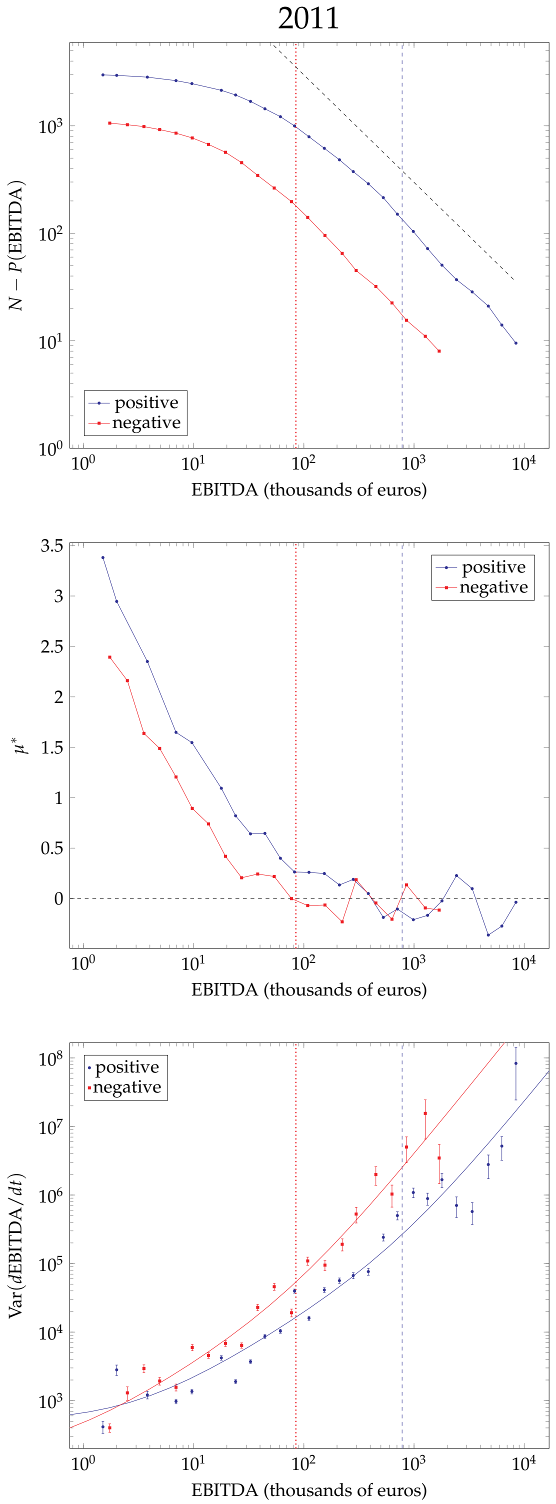

Figure 139: **La Rioja 2011: Rank plot, chemical potential and variance.**
Positive EBITDA: 3048 firms.
T_1 = 0.22 ± 0.08, $T_{1/2}$= 170.10 ± 50.40, and T_0 =494.38 ± 406.84
Negative EBITDA: 1140 firms.
T_1 = 3.74 ± 1.02, $T_{1/2}$= 316.52 ± 112.57, and T_0 =160.73 ± 448.16.
Total active firms 4188, total created firms 196, and total destroyed firms 566

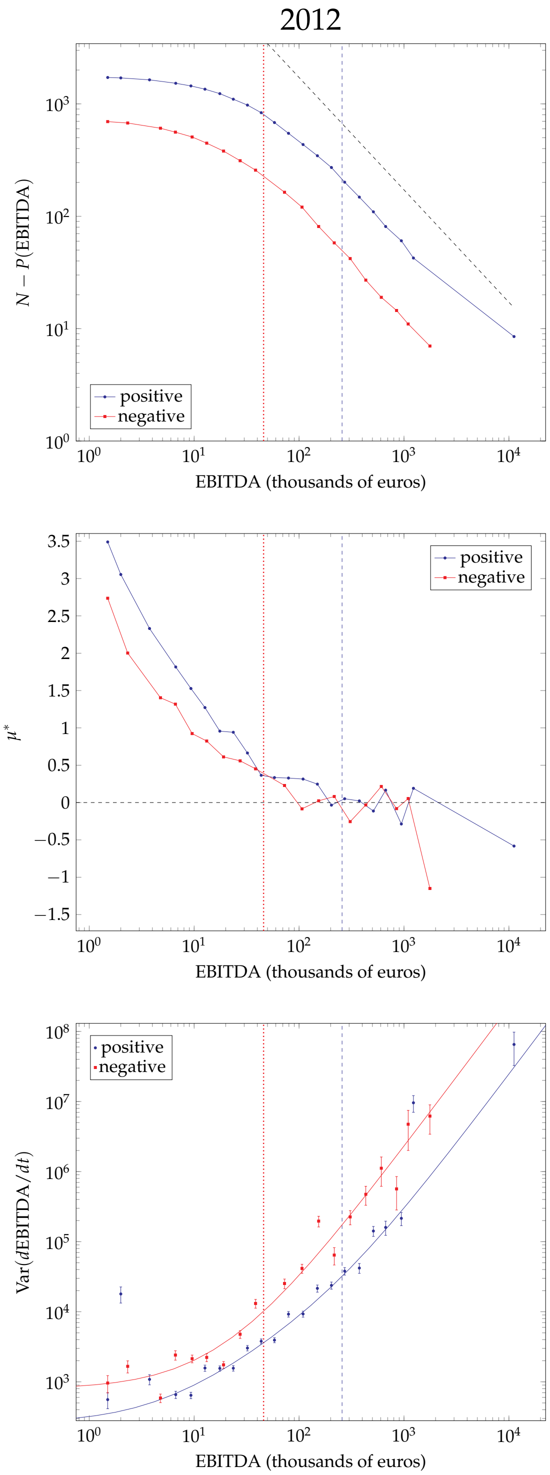

Figure 140: **La Rioja 2012: Rank plot, chemical potential and variance.**
Positive EBITDA: 1768 firms.
T_1 = 0.24 ± 0.07, $T_{1/2}$= 61.82 ± 10.45, and T_0 =257.44 ± 146.59
Negative EBITDA: 739 firms.
T_1 = 2.22 ± 0.53, $T_{1/2}$= 101.79 ± 59.91, and T_0 =789.15 ± 404.82.
Total active firms 2507, total created firms 49, and total destroyed firms 2363

Madrid

Tables of Temperatures

Positive EBITDA

Year	T_1	$T_{1/2}$	T_0	$T_{1/2}/T_1$	Num. Firms
2003	0.68 ± 0.20	125.51 ± 27.00	484.04 ± 142.12	183.4	63272
2004	0.53 ± 0.15	123.53 ± 29.11	799.15 ± 191.81	232.5	63896
2005	0.63 ± 0.14	110.16 ± 27.70	1231.92 ± 246.83	176.3	67493
2006	0.57 ± 0.13	168.40 ± 26.73	953.22 ± 194.24	296.0	69605
2007	0.97 ± 0.26	189.47 ± 45.20	1305.34 ± 306.15	195.3	73353
2008	1.08 ± 0.17	348.30 ± 110.05	773.62 ± 758.01	322.1	64799
2009	1.52 ± 0.26	310.08 ± 105.75	467.32 ± 551.69	204.6	71911
2010	0.96 ± 0.23	376.93 ± 167.96	1019.90 ± 1181.77	390.8	71282
2011	0.63 ± 0.15	357.38 ± 127.46	742.67 ± 824.96	566.2	65043
2012	1.06 ± 0.23	343.96 ± 136.82	361.32 ± 630.33	323.4	25097

Negative EBITDA

Year	T_1	$T_{1/2}$	T_0	$T_{1/2}/T_1$	Num. Firms
2003	1.88 ± 0.58	338.90 ± 484.72	753.66 ± 378.79	180.7	24052
2004	2.39 ± 0.65	246.56 ± 367.00	924.63 ± 305.82	103.4	23852
2005	2.14 ± 0.73	239.58 ± 507.74	1149.98 ± 447.93	111.9	24932
2006	1.76 ± 0.41	275.23 ± 282.15	1107.67 ± 278.38	156.2	25407
2007	3.23 ± 0.84	516.15 ± 511.70	0.00 ± 216.98	159.8	24975
2008	5.32 ± 1.18	1409.10 ± 6440.70	3.21 ± 2016.03	265.1	23474
2009	5.46 ± 1.01	405.25 ± 1426.41	450.60 ± 666.17	74.2	36135
2010	4.02 ± 0.75	501.86 ± 1407.53	129.98 ± 667.84	124.7	38530
2011	3.73 ± 0.62	359.53 ± 1472.01	2883.12 ± 1608.58	96.4	36191
2012	3.31 ± 0.56	382.08 ± 873.14	60.62 ± 422.50	115.3	13874

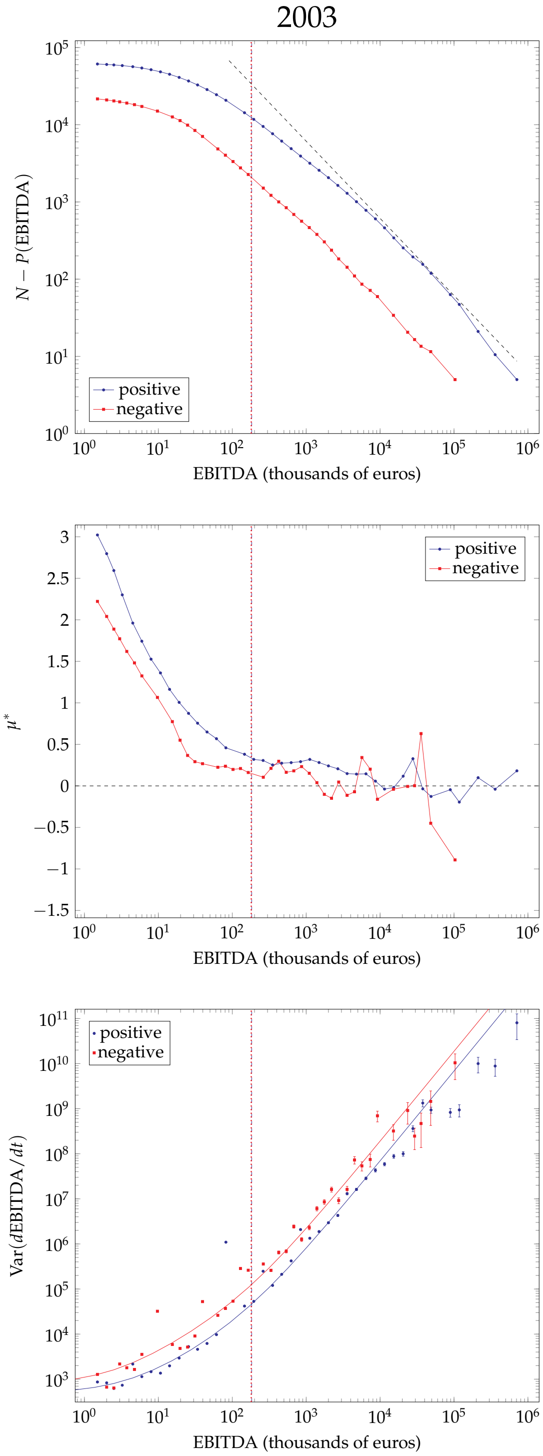

Figure 141: **Madrid 2003: Rank plot, chemical potential and variance.**
Positive EBITDA: 63272 firms.
$T_1 = 0.68 \pm 0.20$, $T_{1/2} = 125.51 \pm 27.00$, and $T_0 = 484.04 \pm 142.12$
Negative EBITDA: 24052 firms.
$T_1 = 1.88 \pm 0.58$, $T_{1/2} = 338.90 \pm 79.24$, and $T_0 = 753.66 \pm 378.79$.
Total active firms 87324, total created firms 15614, and total destroyed firms 9516

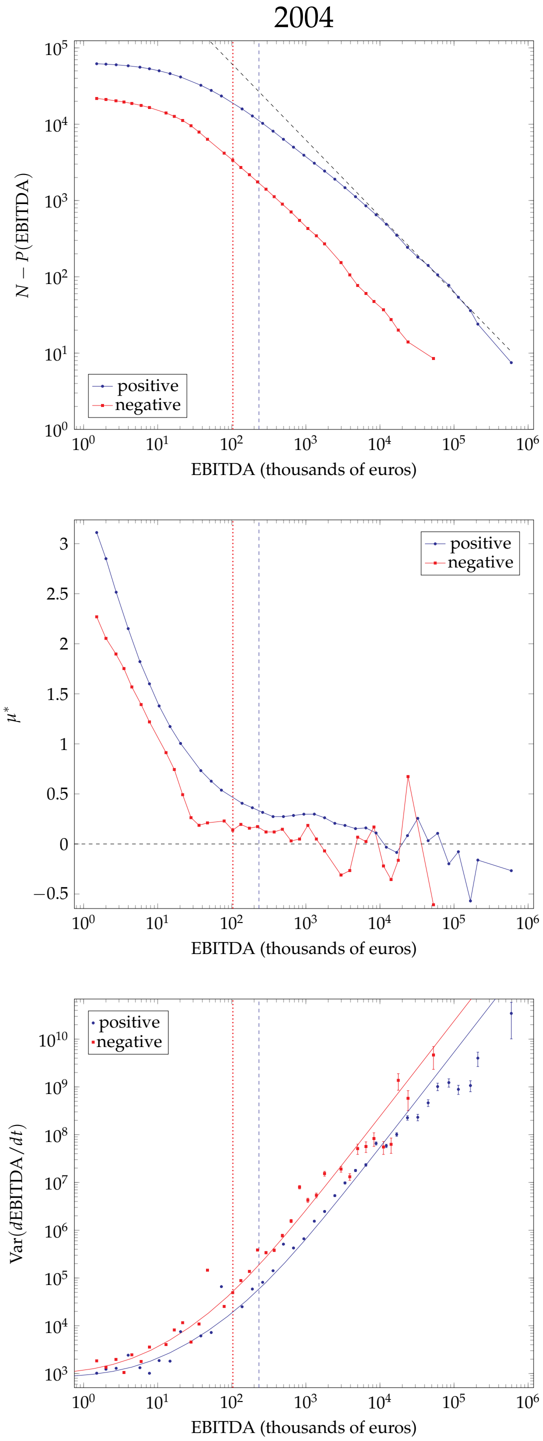

Figure 142: **Madrid 2004: Rank plot, chemical potential and variance**.
Positive EBITDA: 63896 firms.
T_1 = 0.53 ± 0.15, $T_{1/2}$= 123.53 ± 29.11, and T_0 =799.15 ± 191.81
Negative EBITDA: 23852 firms.
T_1 = 2.39 ± 0.65, $T_{1/2}$= 246.56 ± 65.09, and T_0 =924.63 ± 305.82.
Total active firms 87748, total created firms 16803, and total destroyed firms 15308

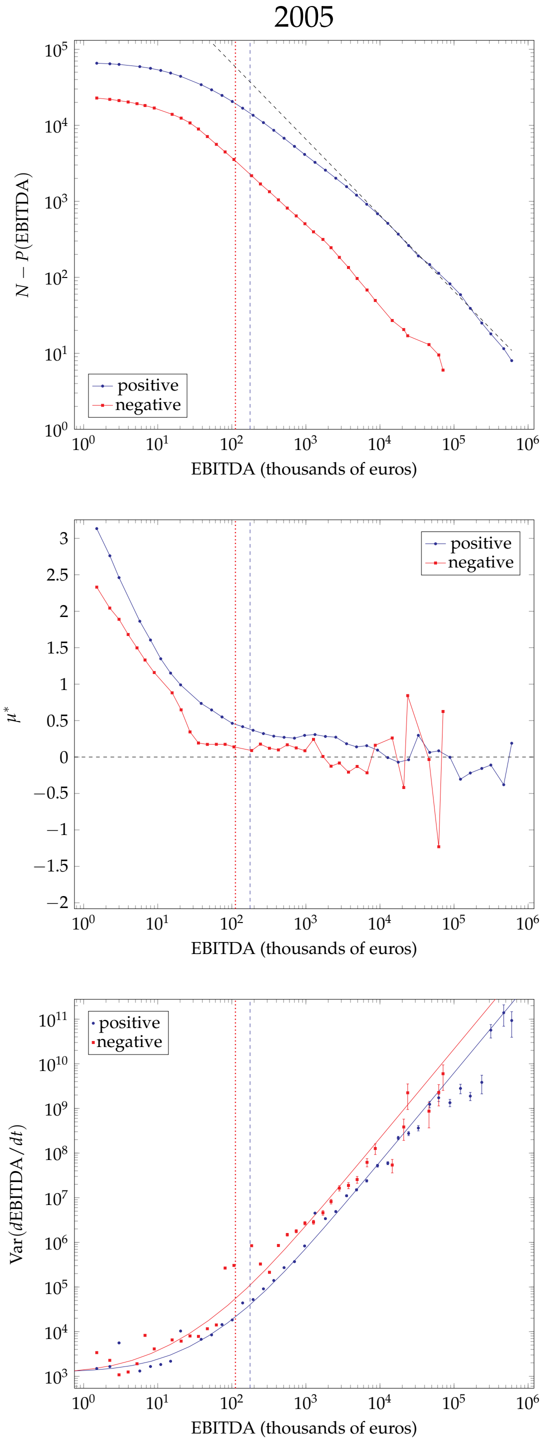

Figure 143: **Madrid 2005: Rank plot, chemical potential and variance**.
Positive EBITDA: 67493 firms.
$T_1 = 0.63 \pm 0.14$, $T_{1/2}$= 110.16 ± 27.70, and T_0 =1231.92 ± 246.83
Negative EBITDA: 24932 firms.
$T_1 = 2.14 \pm 0.73$, $T_{1/2}$= 239.58 ± 81.88, and T_0 =1149.98 ± 447.93.
Total active firms 92425, total created firms 15692, and total destroyed firms 12340

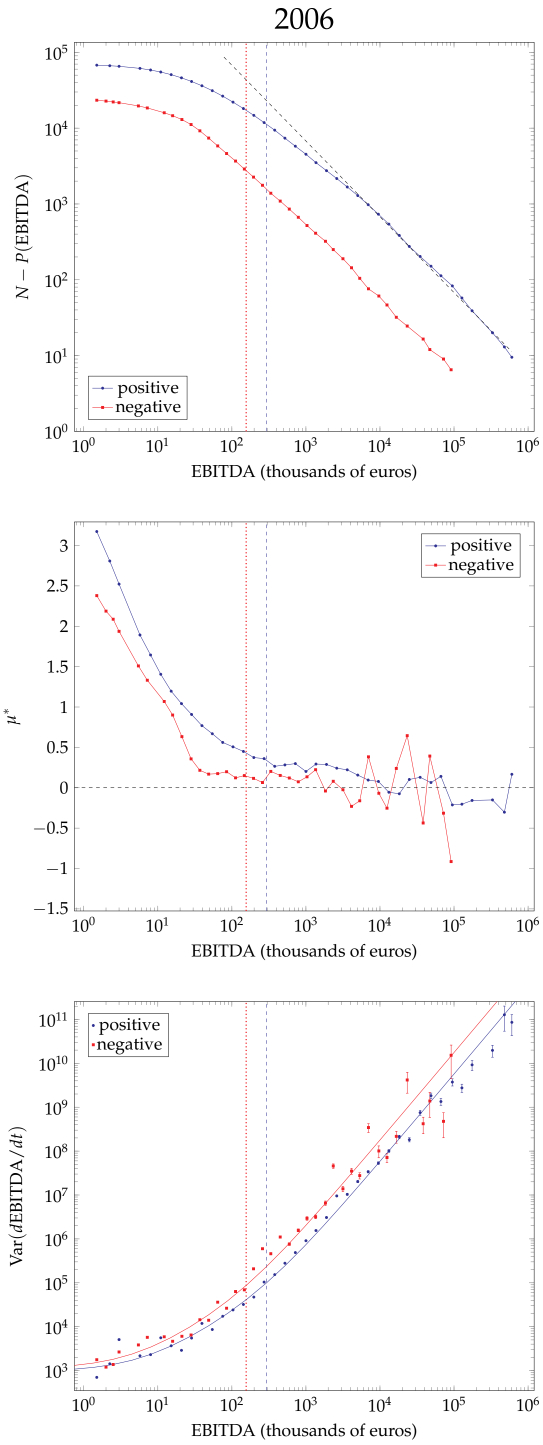

Figure 144: **Madrid 2006: Rank plot, chemical potential and variance.**
Positive EBITDA: 69605 firms.
$T_1 = 0.57 \pm 0.13$, $T_{1/2} = 168.40 \pm 26.73$, and $T_0 = 953.22 \pm 194.24$
Negative EBITDA: 25407 firms.
$T_1 = 1.76 \pm 0.41$, $T_{1/2} = 275.23 \pm 54.04$, and $T_0 = 1107.67 \pm 278.38$.
Total active firms 95012, total created firms 19998, and total destroyed firms 13132

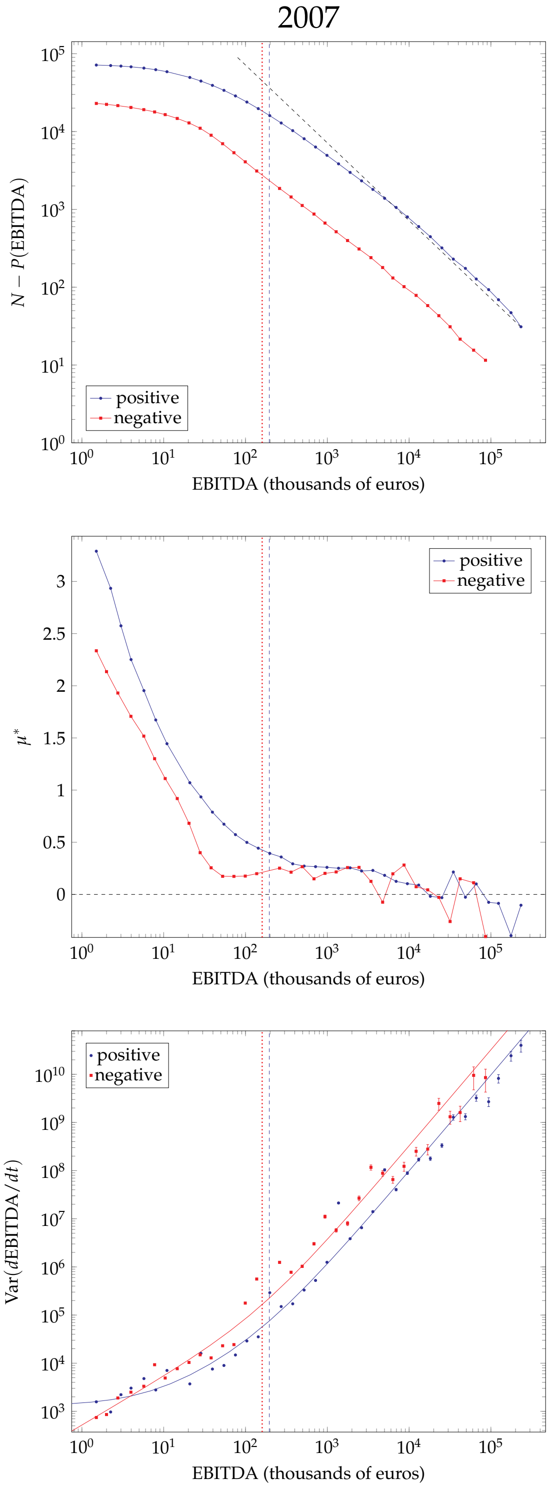

Figure 145: **Madrid 2007: Rank plot, chemical potential and variance.**
Positive EBITDA: 73353 firms.
T_1 = 0.97 ± 0.26, $T_{1/2}$= 189.47 ± 45.20, and T_0 =1305.34 ± 306.15
Negative EBITDA: 24975 firms.
T_1 = 3.23 ± 0.84, $T_{1/2}$= 516.15 ± 82.33, and T_0 =0.00 ± 216.98.
Total active firms 98328, total created firms 8309, and total destroyed firms 16769

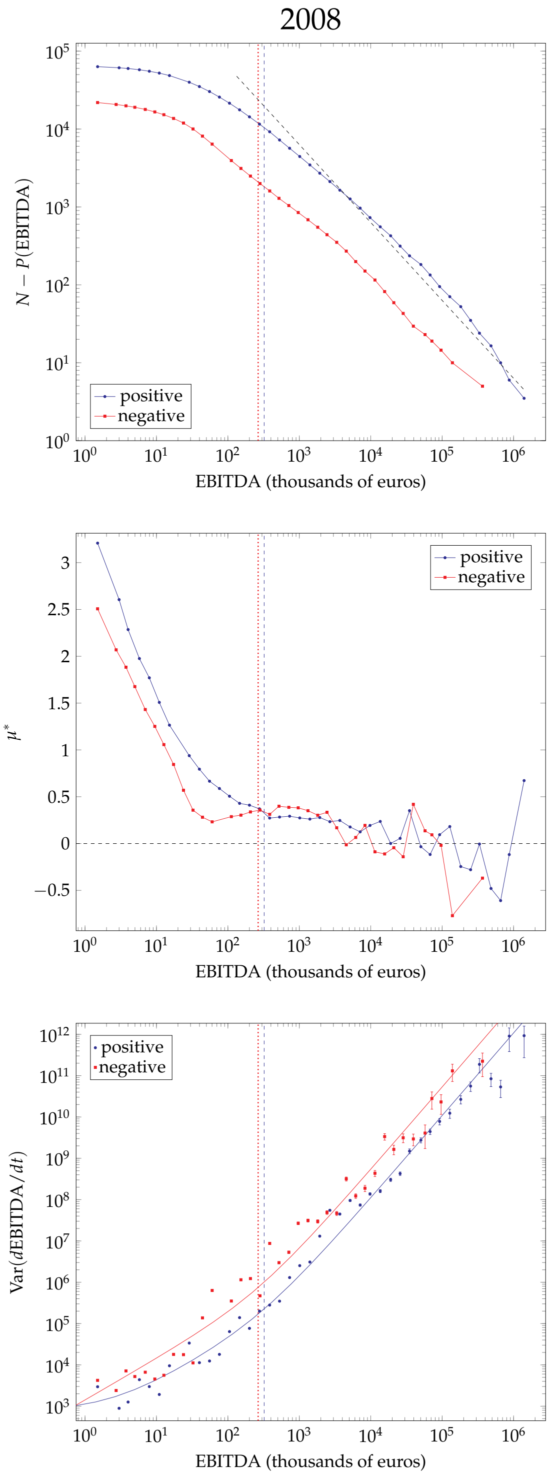

Figure 146: **Madrid 2008: Rank plot, chemical potential and variance**.
Positive EBITDA: 64799 firms.
T_1 = 1.08 ± 0.17, $T_{1/2}$= 348.30 ± 110.05, and T_0 =773.62 ± 758.01
Negative EBITDA: 23474 firms.
T_1 = 5.32 ± 1.18, $T_{1/2}$= 1409.10 ± 493.54, and T_0 =3.21 ± 2016.03.
Total active firms 88273, total created firms 32958, and total destroyed firms 18887

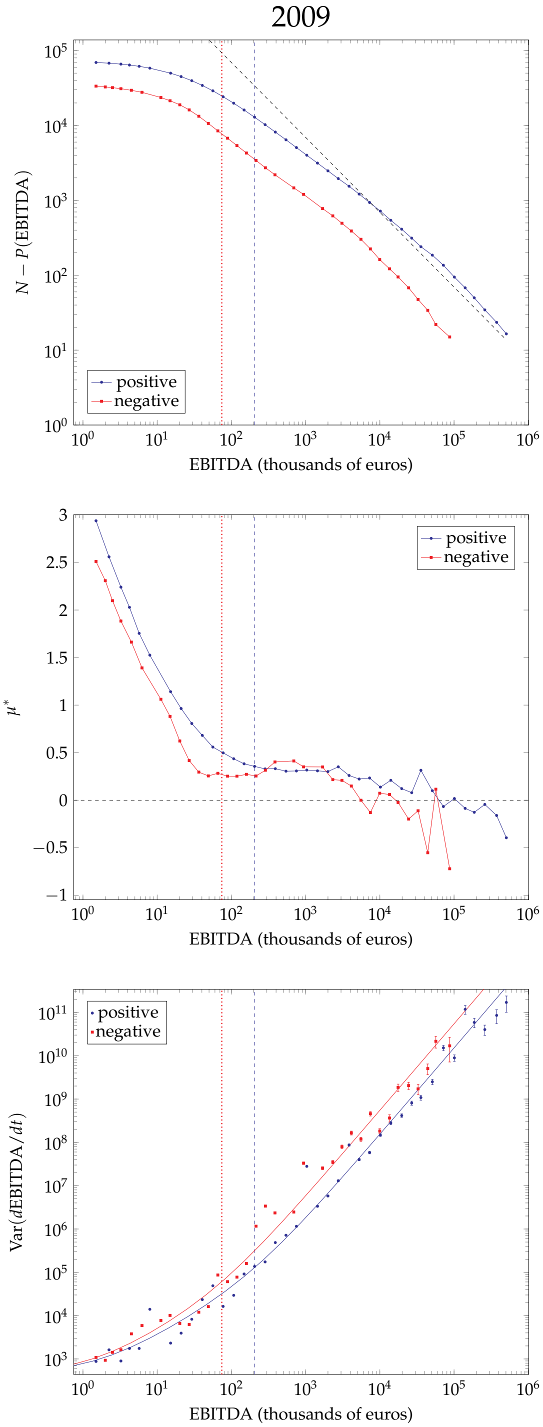

Figure 147: **Madrid 2009: Rank plot, chemical potential and variance**.
Positive EBITDA: 71911 firms.
$T_1 = 1.52 \pm 0.26$, $T_{1/2}$= 310.08 ± 105.75, and T_0 =467.32 ± 551.69
Negative EBITDA: 36135 firms.
$T_1 = 5.46 \pm 1.01$, $T_{1/2}$= 405.25 ± 169.98, and T_0 =450.60 ± 666.17.
Total active firms 108046, total created firms 17614, and total destroyed firms 12452

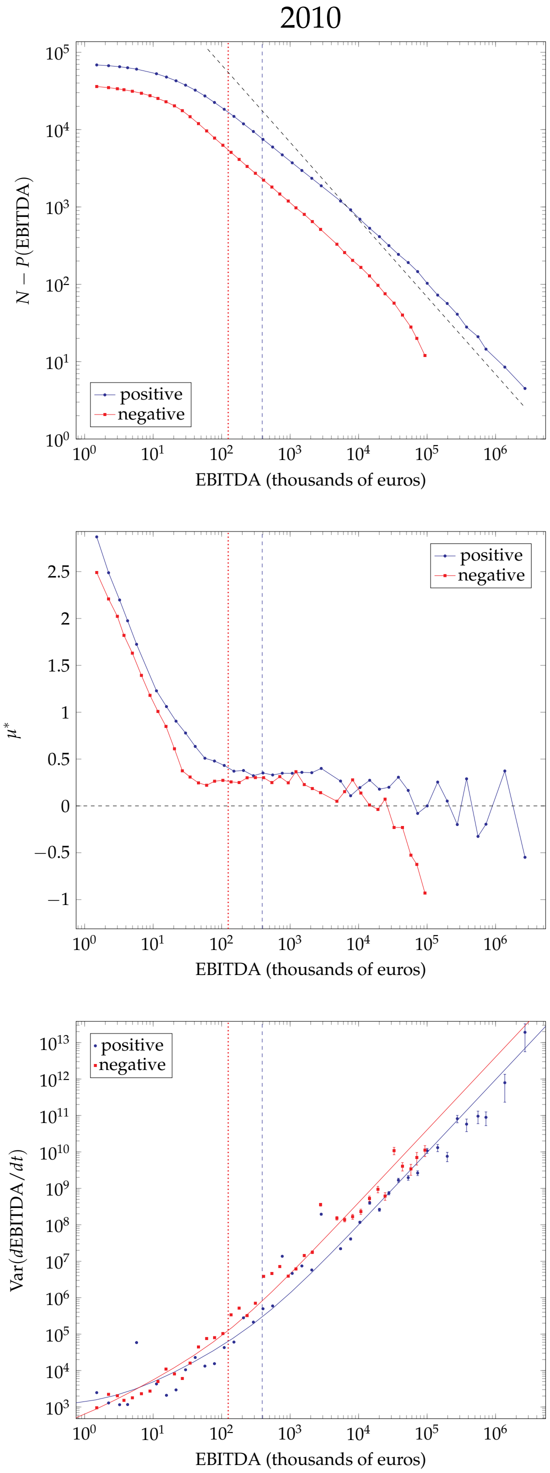

Figure 148: **Madrid 2010: Rank plot, chemical potential and variance**.
Positive EBITDA: 71282 firms.
T_1 = 0.96 ± 0.23, $T_{1/2}$= 376.93 ± 167.96, and T_0 =1019.90 ± 1181.77
Negative EBITDA: 38530 firms.
T_1 = 4.02 ± 0.75, $T_{1/2}$= 501.86 ± 168.38, and T_0 =129.98 ± 667.84.
Total active firms 109812, total created firms 13053, and total destroyed firms 15941

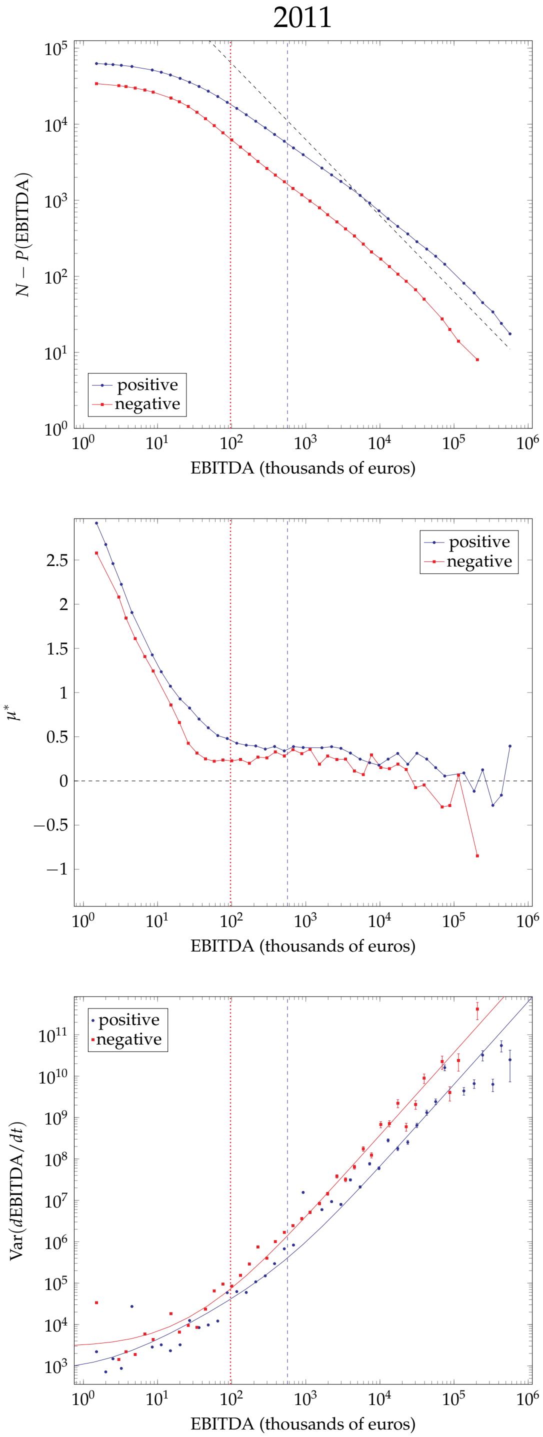

Figure 149: **Madrid 2011: Rank plot, chemical potential and variance**.
Positive EBITDA: 65043 firms.
$T_1 = 0.63 \pm 0.15$, $T_{1/2}$= 357.38 ± 127.46, and T_0 =742.67 ± 824.96
Negative EBITDA: 36191 firms.
$T_1 = 3.73 \pm 0.62$, $T_{1/2}$= 359.53 ± 173.80, and T_0 =2883.12 ± 1608.58.
Total active firms 101234, total created firms 4815, and total destroyed firms 21929

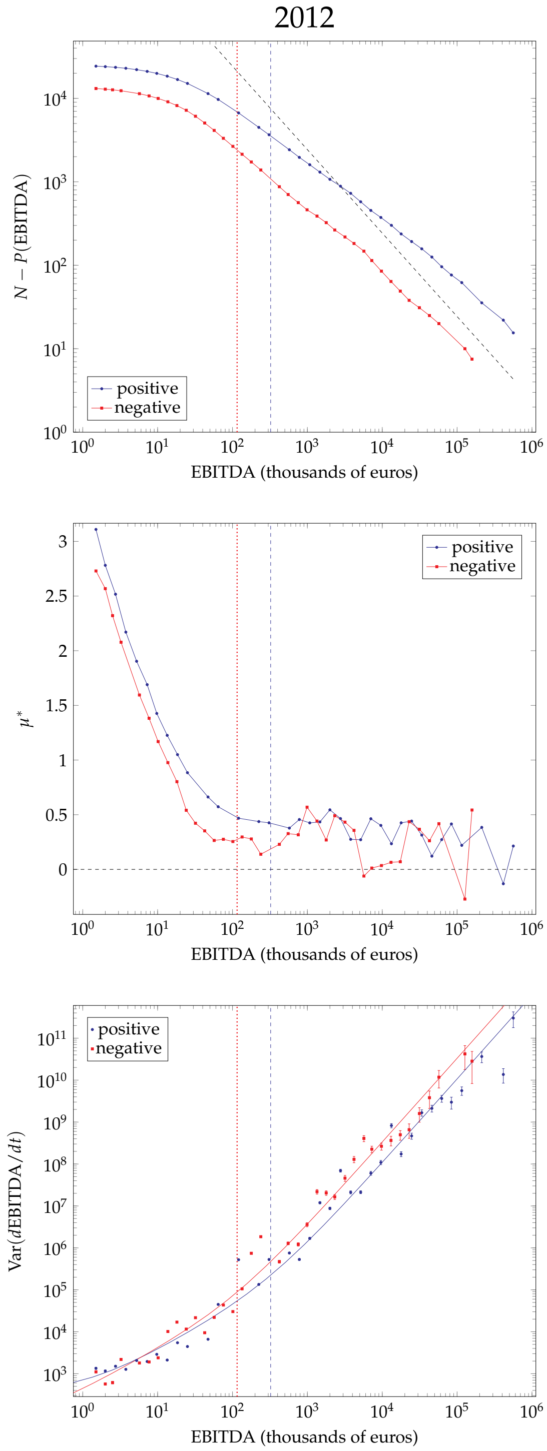

Figure 150: **Madrid 2012: Rank plot, chemical potential and variance.**
Positive EBITDA: 25097 firms.
T_1 = 1.06 ± 0.23, $T_{1/2}$= 343.96 ± 136.82, and T_0 =361.32 ± 630.33
Negative EBITDA: 13874 firms.
T_1 = 3.31 ± 0.56, $T_{1/2}$= 382.08 ± 120.13, and T_0 =60.62 ± 422.50.
Total active firms 38971, total created firms 709, and total destroyed firms 67996

Murcia

Tables of Temperatures

Positive EBITDA

Year	T_1	$T_{1/2}$	T_0	$T_{1/2}/T_1$	Num. Firms
2003	0.42 ± 0.13	27.57 ± 14.62	472.87 ± 140.53	65.7	11716
2004	0.47 ± 0.13	37.91 ± 10.61	265.82 ± 68.81	81.1	12667
2005	0.57 ± 0.11	37.46 ± 10.96	460.79 ± 99.99	66.2	13434
2006	0.64 ± 0.13	54.63 ± 12.68	244.85 ± 91.68	85.9	14319
2007	0.62 ± 0.13	54.51 ± 11.38	296.67 ± 63.18	87.4	15930
2008	0.73 ± 0.20	92.01 ± 25.82	540.82 ± 163.32	126.7	13813
2009	0.93 ± 0.28	209.52 ± 84.84	275.22 ± 426.30	224.4	12492
2010	0.70 ± 0.12	62.89 ± 26.22	662.57 ± 236.58	89.8	12949
2011	0.62 ± 0.21	183.94 ± 31.13	0.04 ± 82.59	297.3	11421
2012	0.38 ± 0.08	72.88 ± 20.80	243.73 ± 132.74	193.8	6442

Negative EBITDA

Year	T_1	$T_{1/2}$	T_0	$T_{1/2}/T_1$	Num. Firms
2003	3.01 ± 1.25	152.82 ± 519.84	945.99 ± 458.42	50.8	1854
2004	2.18 ± 0.66	280.45 ± 407.10	286.60 ± 253.41	128.8	2022
2005	5.21 ± 1.58	158.96 ± 718.96	2082.56 ± 717.44	30.5	2190
2006	1.82 ± 0.61	385.70 ± 422.48	331.45 ± 351.33	211.7	2403
2007	3.92 ± 1.55	270.93 ± 858.88	1103.41 ± 599.33	69.1	2852
2008	3.74 ± 1.19	360.02 ± 599.30	647.96 ± 390.27	96.3	2645
2009	4.01 ± 0.93	381.30 ± 1548.69	2885.29 ± 1489.78	95.2	3402
2010	2.32 ± 0.47	212.11 ± 558.66	1258.57 ± 627.17	91.6	4418
2011	4.11 ± 1.33	273.11 ± 542.26	655.26 ± 356.88	66.4	3982
2012	3.00 ± 0.46	92.75 ± 273.89	1548.89 ± 423.73	30.9	2503

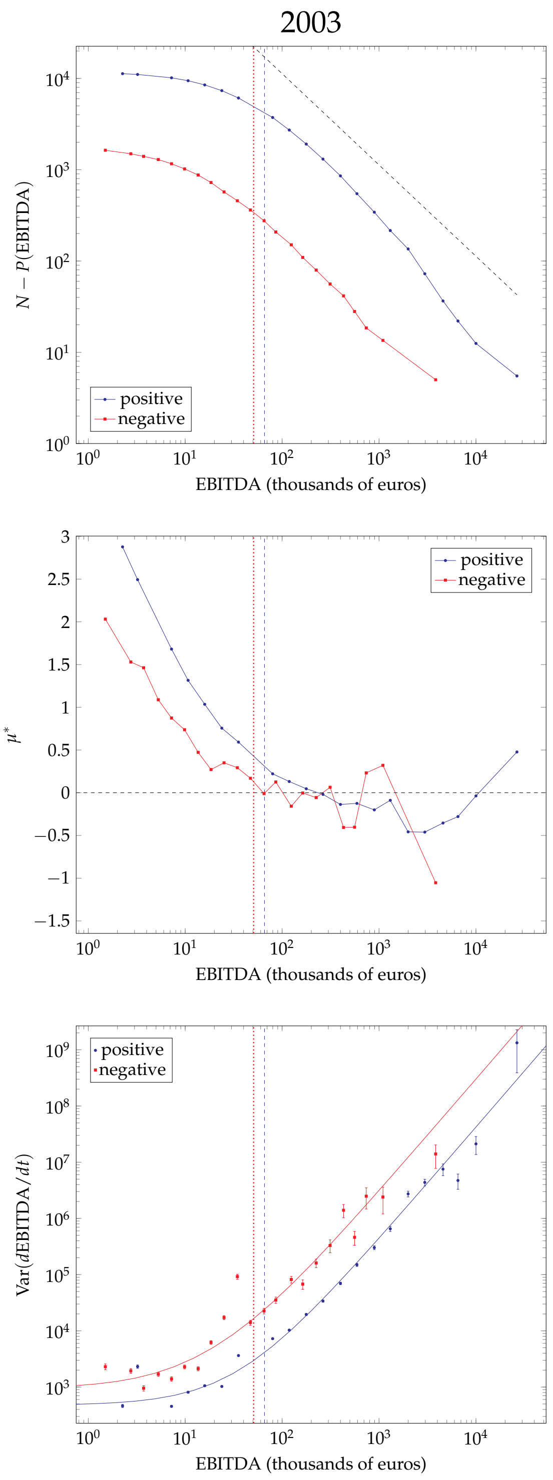

Figure 151: **Murcia 2003: Rank plot, chemical potential and variance.**
Positive EBITDA: 11716 firms.
$T_1 = 0.42 \pm 0.13$, $T_{1/2} = 27.57 \pm 14.62$, and $T_0 = 472.87 \pm 140.53$
Negative EBITDA: 1854 firms.
$T_1 = 3.01 \pm 1.25$, $T_{1/2} = 152.82 \pm 83.26$, and $T_0 = 945.99 \pm 458.42$.
Total active firms 13570, total created firms 3176, and total destroyed firms 789

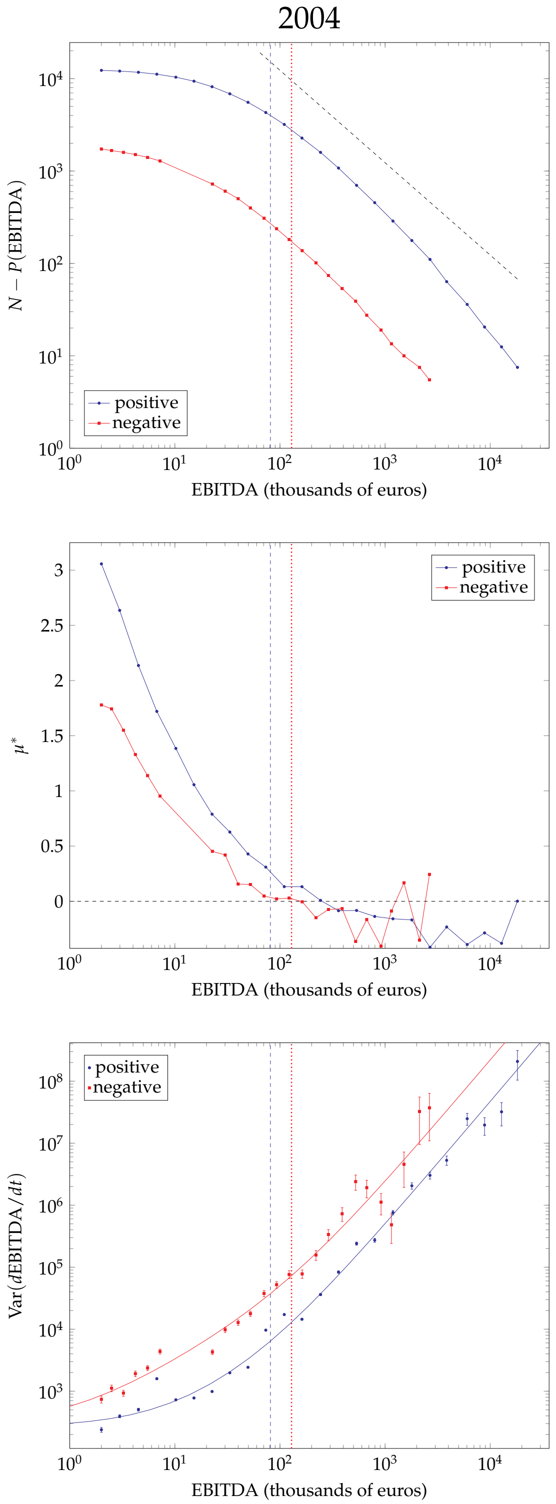

Figure 152: **Murcia 2004: Rank plot, chemical potential and variance.**
Positive EBITDA: 12667 firms.
$T_1 = 0.47 \pm 0.13$, $T_{1/2} = 37.91 \pm 10.61$, and $T_0 = 265.82 \pm 68.81$
Negative EBITDA: 2022 firms.
$T_1 = 2.18 \pm 0.66$, $T_{1/2} = 280.45 \pm 70.04$, and $T_0 = 286.60 \pm 253.41$.
Total active firms 14689, total created firms 3040, and total destroyed firms 2016

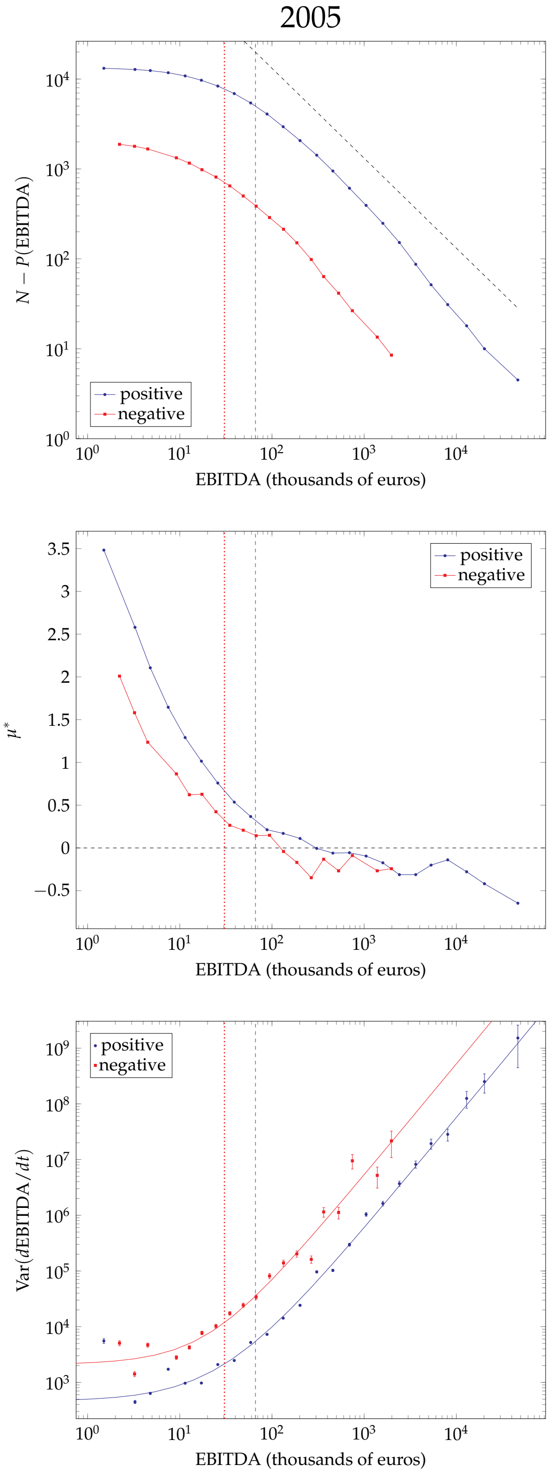

Figure 153: **Murcia 2005: Rank plot, chemical potential and variance**.
Positive EBITDA: 13434 firms.
T_1 = 0.57 ± 0.11, $T_{1/2}$= 37.46 ± 10.96, and T_0 =460.79 ± 99.99
Negative EBITDA: 2190 firms.
T_1 = 5.21 ± 1.58, $T_{1/2}$= 158.96 ± 104.71, and T_0 =2082.56 ± 717.44.
Total active firms 15624, total created firms 2843, and total destroyed firms 2126

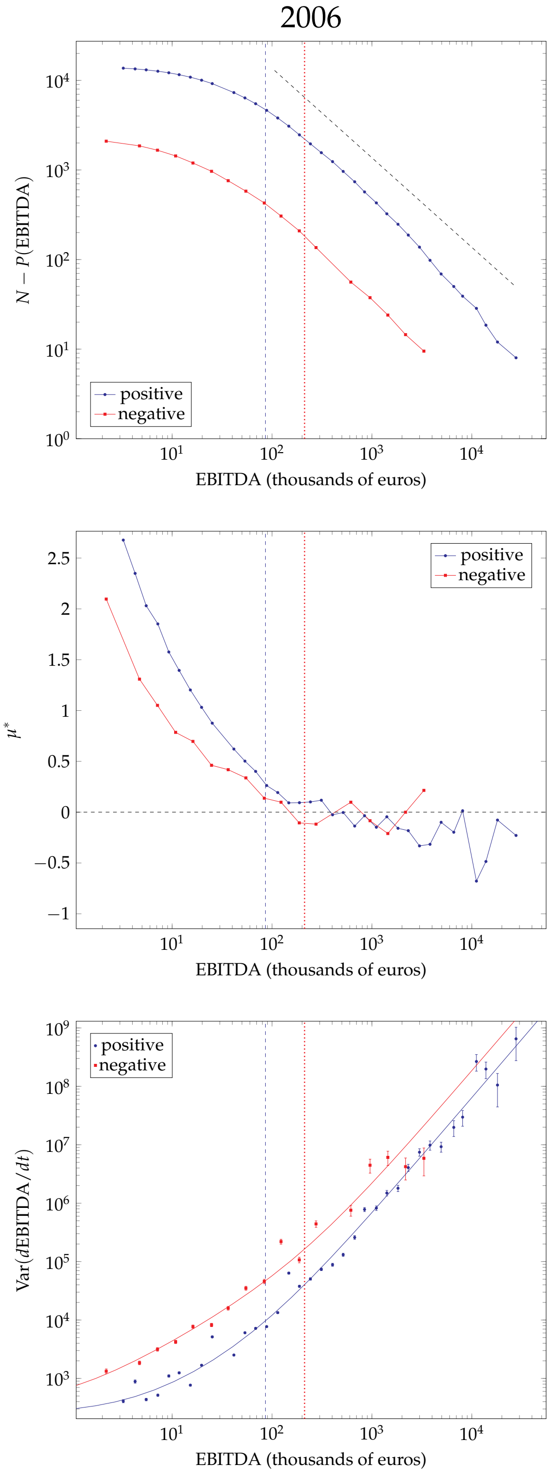

Figure 154: **Murcia 2006: Rank plot, chemical potential and variance.**
Positive EBITDA: 14319 firms.
T_1 = 0.64 ± 0.13, $T_{1/2}$= 54.63 ± 12.68, and T_0 =244.85 ± 91.68
Negative EBITDA: 2403 firms.
T_1 = 1.82 ± 0.61, $T_{1/2}$= 385.70 ± 71.90, and T_0 =331.45 ± 351.33.
Total active firms 16722, total created firms 4614, and total destroyed firms 1753

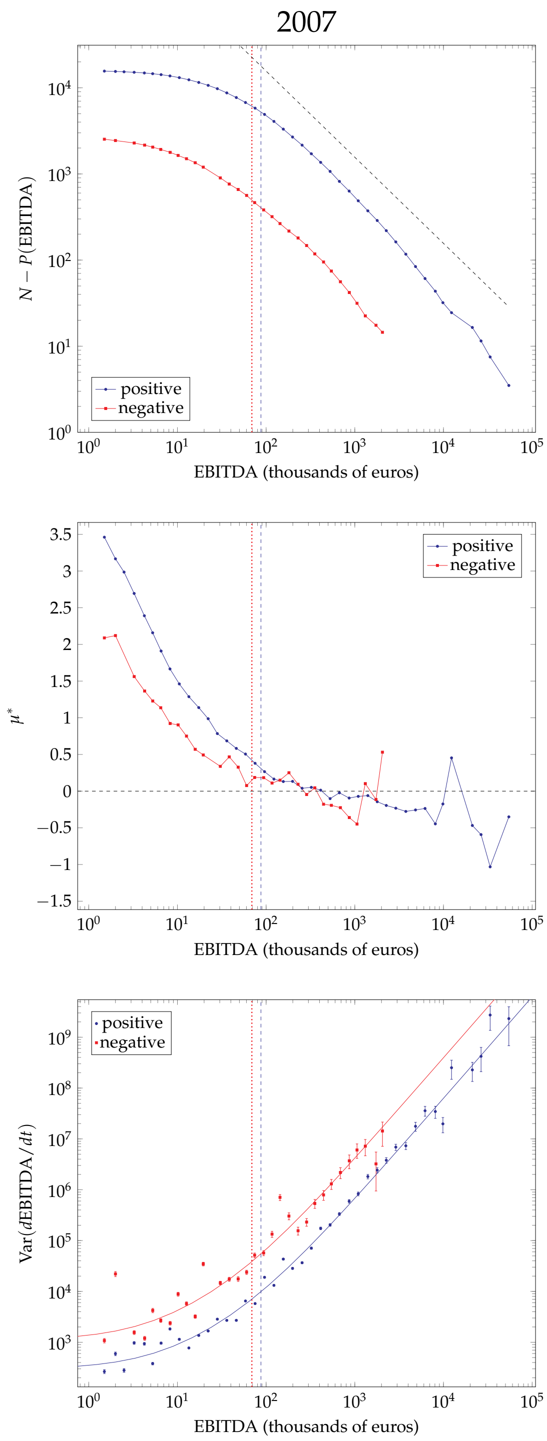

Figure 155: **Murcia 2007: Rank plot, chemical potential and variance.**
Positive EBITDA: 15930 firms.
T_1 = 0.62 ± 0.13, $T_{1/2}$= 54.51 ± 11.38, and T_0 =296.67 ± 63.18
Negative EBITDA: 2852 firms.
T_1 = 3.92 ± 1.55, $T_{1/2}$= 270.93 ± 118.74, and T_0 =1103.41 ± 599.33.
Total active firms 18782, total created firms 1595, and total destroyed firms 2470

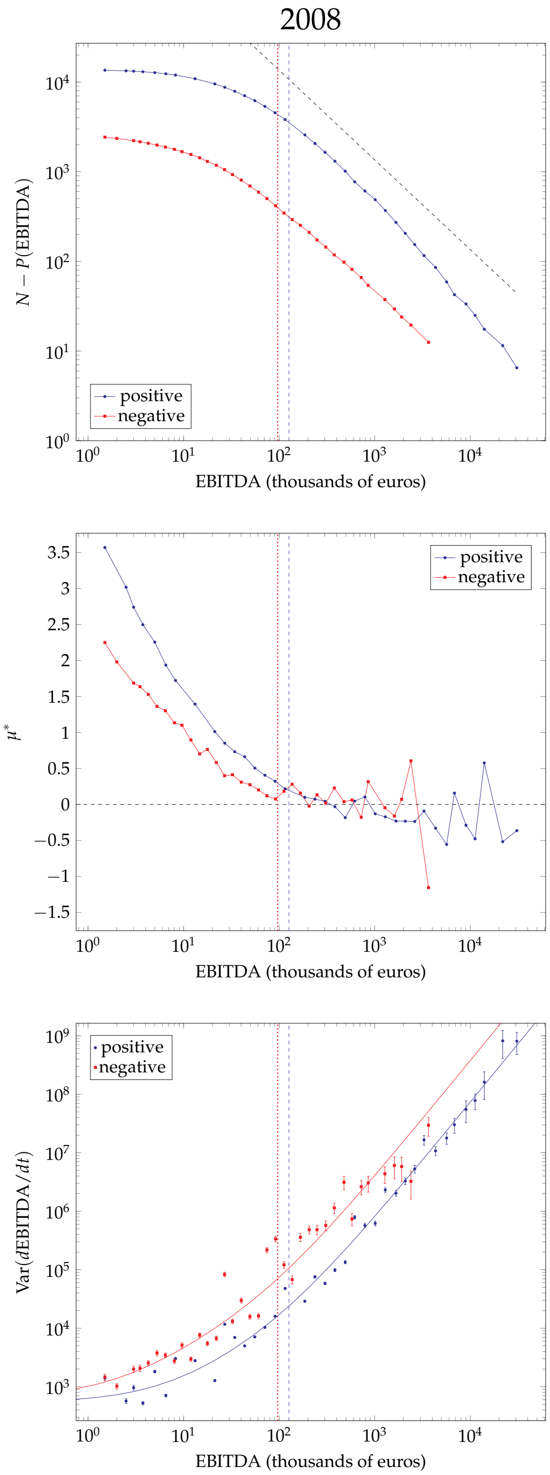

Figure 156: **Murcia 2008: Rank plot, chemical potential and variance.**
Positive EBITDA: 13813 firms.
T_1 = 0.73 ± 0.20, $T_{1/2}$= 92.01 ± 25.82, and T_0 =540.82 ± 163.32
Negative EBITDA: 2645 firms.
T_1 = 3.74 ± 1.19, $T_{1/2}$= 360.02 ± 92.06, and T_0 =647.96 ± 390.27.
Total active firms 16458, total created firms 3905, and total destroyed firms 4029

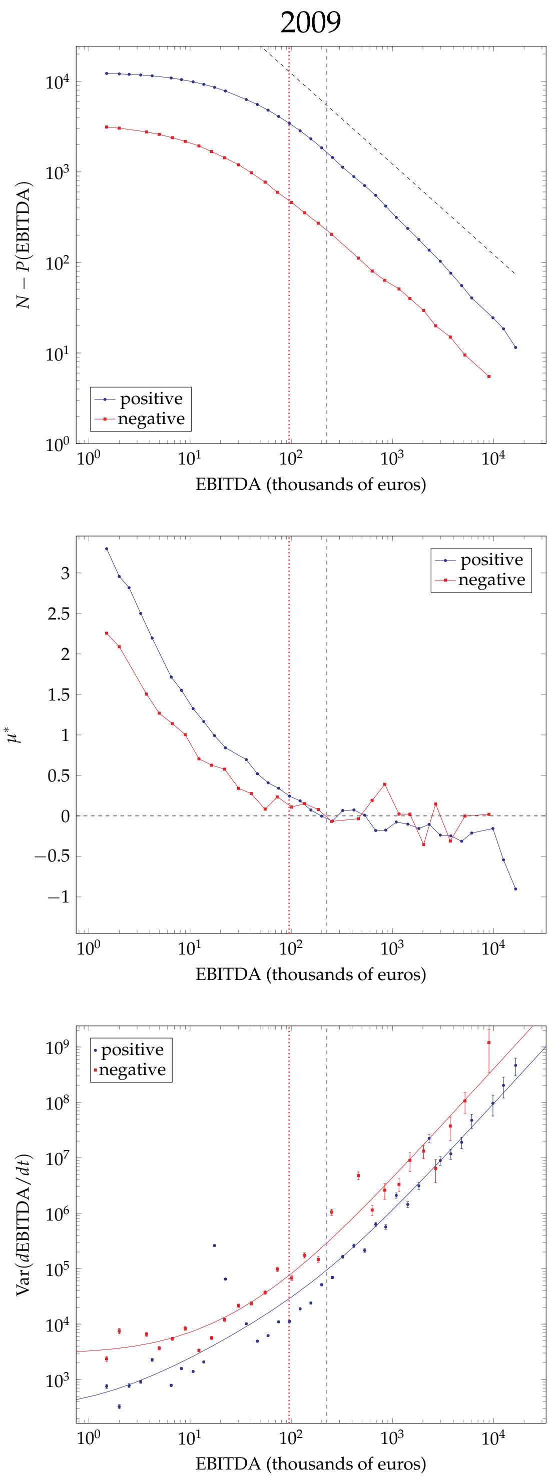

Figure 157: **Murcia 2009: Rank plot, chemical potential and variance.**
Positive EBITDA: 12492 firms.
T_1 = 0.93 ± 0.28, $T_{1/2}$= 209.52 ± 84.84, and T_0 =275.22 ± 426.30
Negative EBITDA: 3402 firms.
T_1 = 4.01 ± 0.93, $T_{1/2}$= 381.30 ± 180.15, and T_0 =2885.29 ± 1489.78.
Total active firms 15894, total created firms 2144, and total destroyed firms 2378

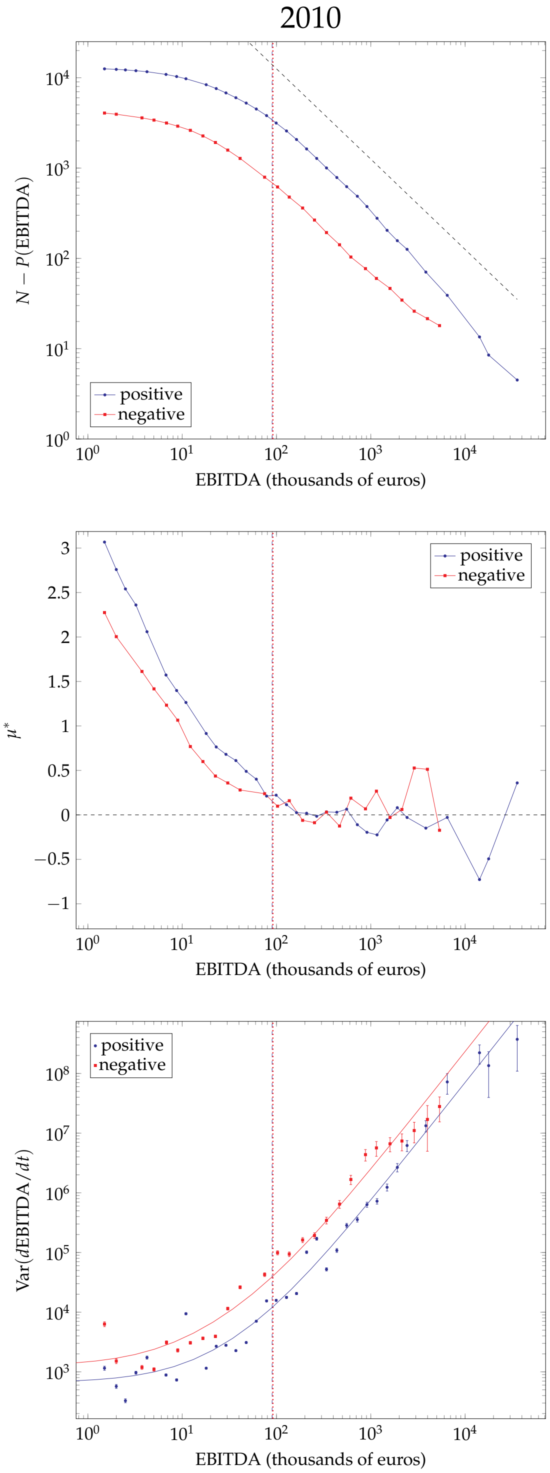

Figure 158: **Murcia 2010: Rank plot, chemical potential and variance.**
Positive EBITDA: 12949 firms.
$T_1 = 0.70 \pm 0.12$, $T_{1/2}$= 62.89 ± 26.22, and T_0 =662.57 ± 236.58
Negative EBITDA: 4418 firms.
$T_1 = 2.32 \pm 0.47$, $T_{1/2}$= 212.11 ± 87.60, and T_0 =1258.57 ± 627.17.
Total active firms 17367, total created firms 2094, and total destroyed firms 2684

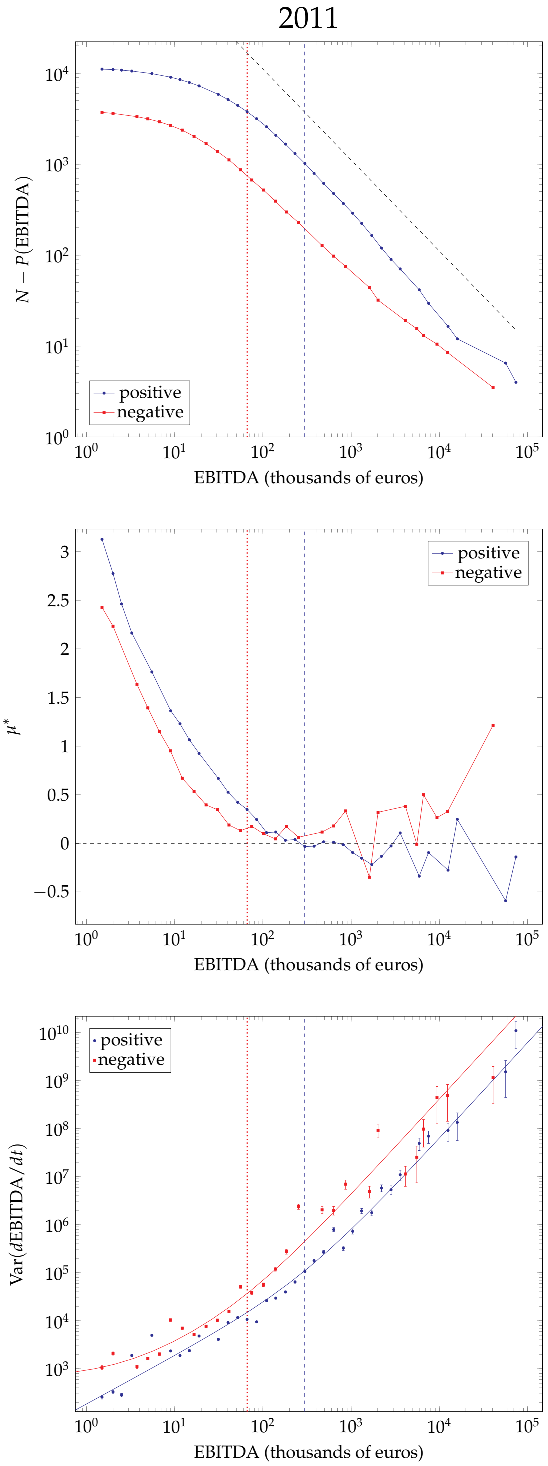

Figure 159: **Murcia 2011: Rank plot, chemical potential and variance**.
Positive EBITDA: 11421 firms.
$T_1 = 0.62 \pm 0.21$, $T_{1/2}$= 183.94 ± 31.13, and T_0 =0.04 ± 82.59
Negative EBITDA: 3982 firms.
$T_1 = 4.11 \pm 1.33$, $T_{1/2}$= 273.11 ± 85.78, and T_0 =655.26 ± 356.88.
Total active firms 15403, total created firms 988, and total destroyed firms 2371

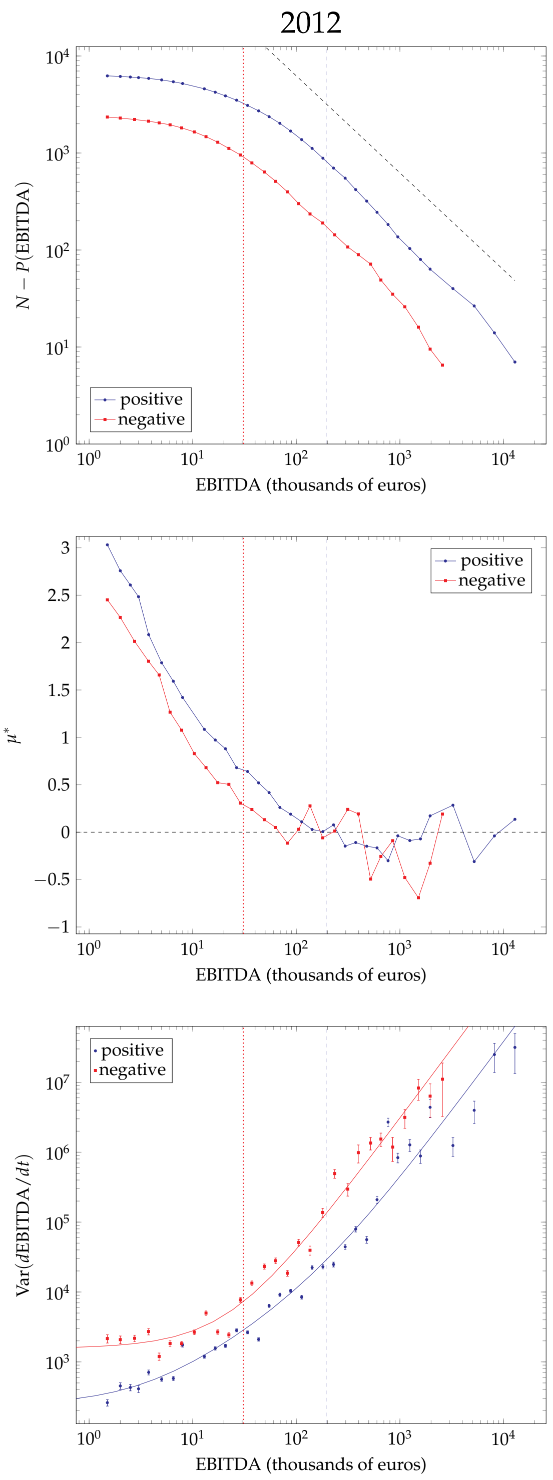

Figure 160: **Murcia 2012: Rank plot, chemical potential and variance.**
Positive EBITDA: 6442 firms.
T_1 = 0.38 ± 0.08, $T_{1/2}$= 72.88 ± 20.80, and T_0 =243.73 ± 132.74
Negative EBITDA: 2503 firms.
T_1 = 3.00 ± 0.46, $T_{1/2}$= 92.75 ± 52.92, and T_0 =1548.89 ± 423.73.
Total active firms 8945, total created firms 187, and total destroyed firms 9252

Navarre

Tables of Temperatures

Positive EBITDA

Year	T_1	$T_{1/2}$	T_0	$T_{1/2}/T_1$	Num. Firms
2003	0.36 ± 0.05	51.95 ± 16.10	303.20 ± 129.73	144.8	5857
2004	0.30 ± 0.08	146.59 ± 42.61	164.70 ± 242.94	484.4	6076
2005	0.41 ± 0.11	37.05 ± 12.26	406.83 ± 97.49	89.4	6436
2006	0.51 ± 0.14	31.02 ± 20.54	1011.53 ± 259.33	61.1	6821
2007	0.41 ± 0.10	54.69 ± 41.17	1629.73 ± 718.52	133.1	6987
2008	0.60 ± 0.17	183.39 ± 70.05	235.88 ± 351.01	307.1	6390
2009	0.74 ± 0.20	158.78 ± 78.73	397.73 ± 682.50	215.6	6594
2010	0.79 ± 0.17	28.08 ± 20.16	1107.93 ± 337.68	35.5	6690
2011	0.80 ± 0.22	94.25 ± 30.70	980.95 ± 474.29	118.2	6077
2012	0.32 ± 0.12	69.66 ± 20.38	549.43 ± 165.62	216.1	4120

Negative EBITDA

Year	T_1	$T_{1/2}$	T_0	$T_{1/2}/T_1$	Num. Firms
2003	1.80 ± 0.92	75.99 ± 381.28	311.50 ± 584.37	42.3	1020
2004	1.66 ± 0.81	317.84 ± 1021.63	0.02 ± 455.91	191.5	1039
2005	2.18 ± 1.55	280.96 ± 1319.38	748.07 ± 657.79	128.6	1137
2006	1.31 ± 0.72	149.81 ± 481.76	211.85 ± 650.33	114.7	1207
2007	3.12 ± 1.23	289.54 ± 1393.37	261.05 ± 765.88	92.8	1163
2008	4.92 ± 2.11	217.26 ± 1948.40	938.02 ± 1133.28	44.2	1137
2009	4.84 ± 1.16	233.34 ± 1147.99	1878.86 ± 993.21	48.2	1624
2010	5.44 ± 1.57	164.17 ± 1435.46	1785.77 ± 1282.76	30.2	2266
2011	2.75 ± 1.03	270.19 ± 603.85	835.64 ± 493.70	98.2	2018
2012	2.86 ± 1.71	59.55 ± 562.23	1026.19 ± 460.94	20.8	1710

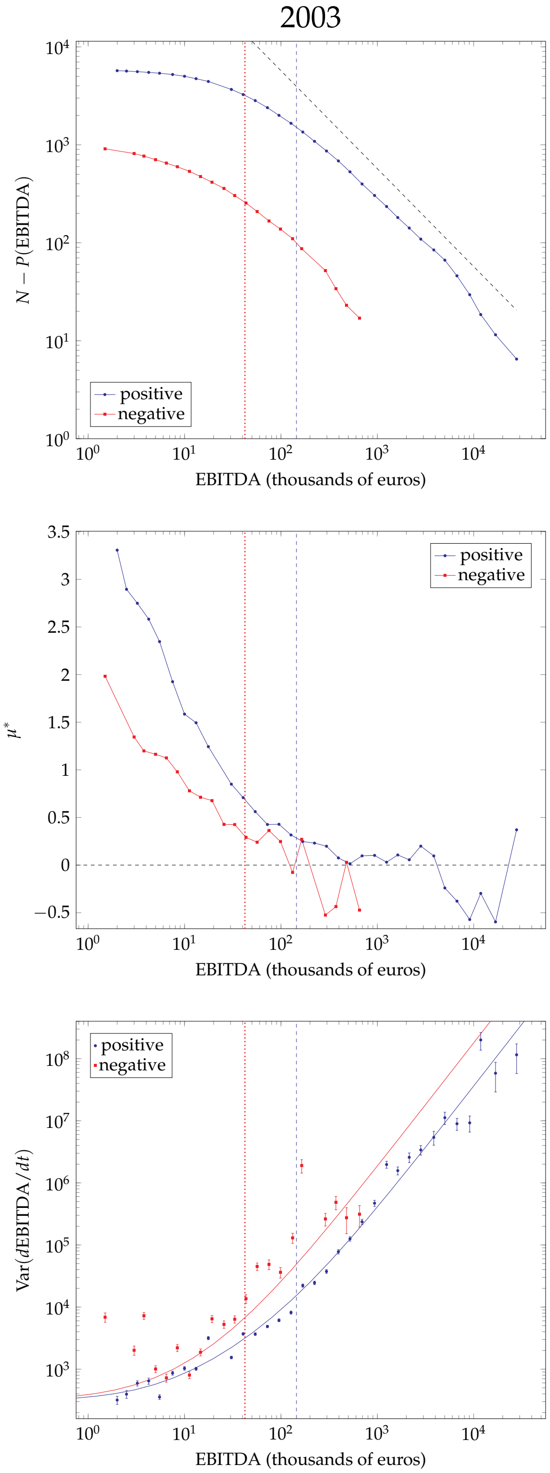

Figure 161: **Navarre 2003: Rank plot, chemical potential and variance.**
Positive EBITDA: 5857 firms.
T_1 = 0.36 ± 0.05, $T_{1/2}$= 51.95 ± 16.10, and T_0 =303.20 ± 129.73
Negative EBITDA: 1020 firms.
T_1 = 1.80 ± 0.92, $T_{1/2}$= 75.99 ± 66.87, and T_0 =311.50 ± 584.37.
Total active firms 6877, total created firms 1139, and total destroyed firms 364

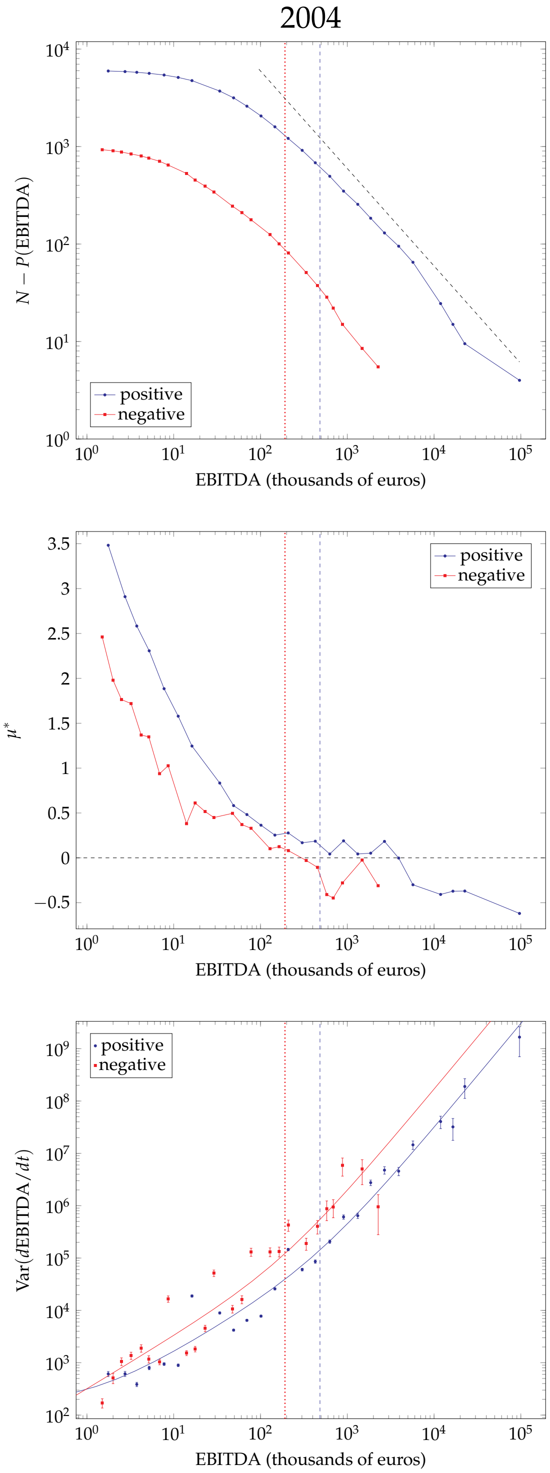

Figure 162: **Navarre 2004: Rank plot, chemical potential and variance.**
Positive EBITDA: 6076 firms.
T_1 = 0.30 ± 0.08, $T_{1/2}$= 146.59 ± 42.61, and T_0 =164.70 ± 242.94
Negative EBITDA: 1039 firms.
T_1 = 1.66 ± 0.81, $T_{1/2}$= 317.84 ± 134.24, and T_0 =0.02 ± 455.91.
Total active firms 7115, total created firms 1125, and total destroyed firms 900

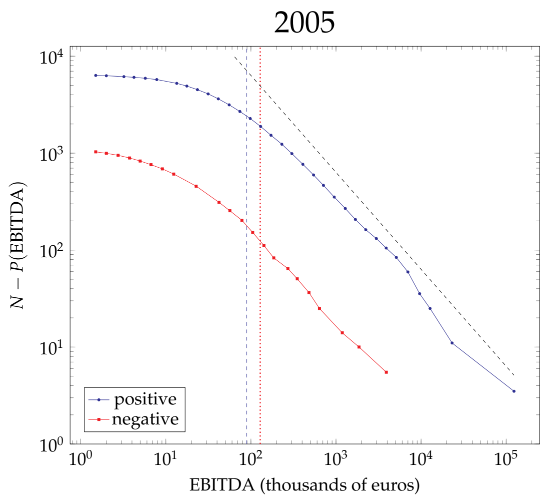

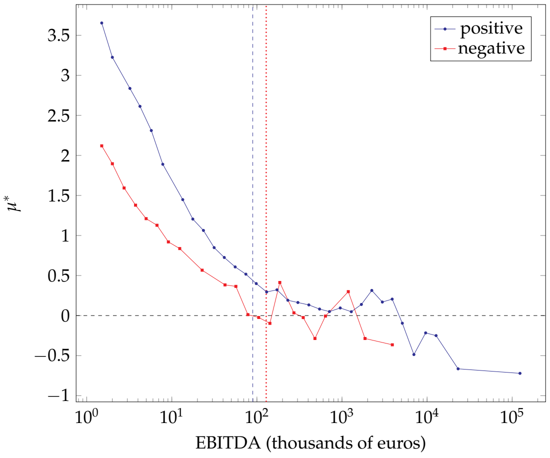

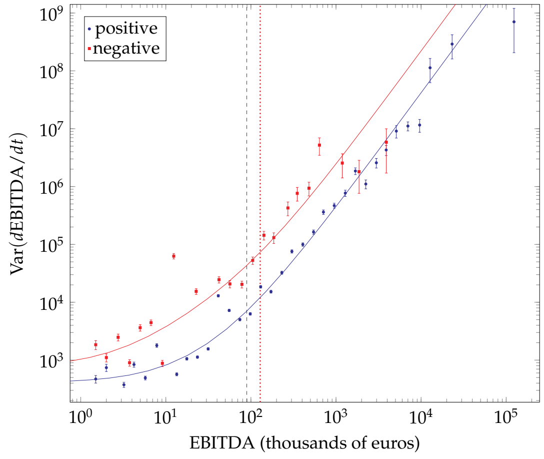

Figure 163: **Navarre 2005: Rank plot, chemical potential and variance.**
Positive EBITDA: 6436 firms.
T_1 = 0.41 ± 0.11, $T_{1/2}$= 37.05 ± 12.26, and T_0 =406.83 ± 97.49
Negative EBITDA: 1137 firms.
T_1 = 2.18 ± 1.55, $T_{1/2}$= 280.96 ± 160.86, and T_0 =748.07 ± 657.79.
Total active firms 7573, total created firms 1195, and total destroyed firms 677

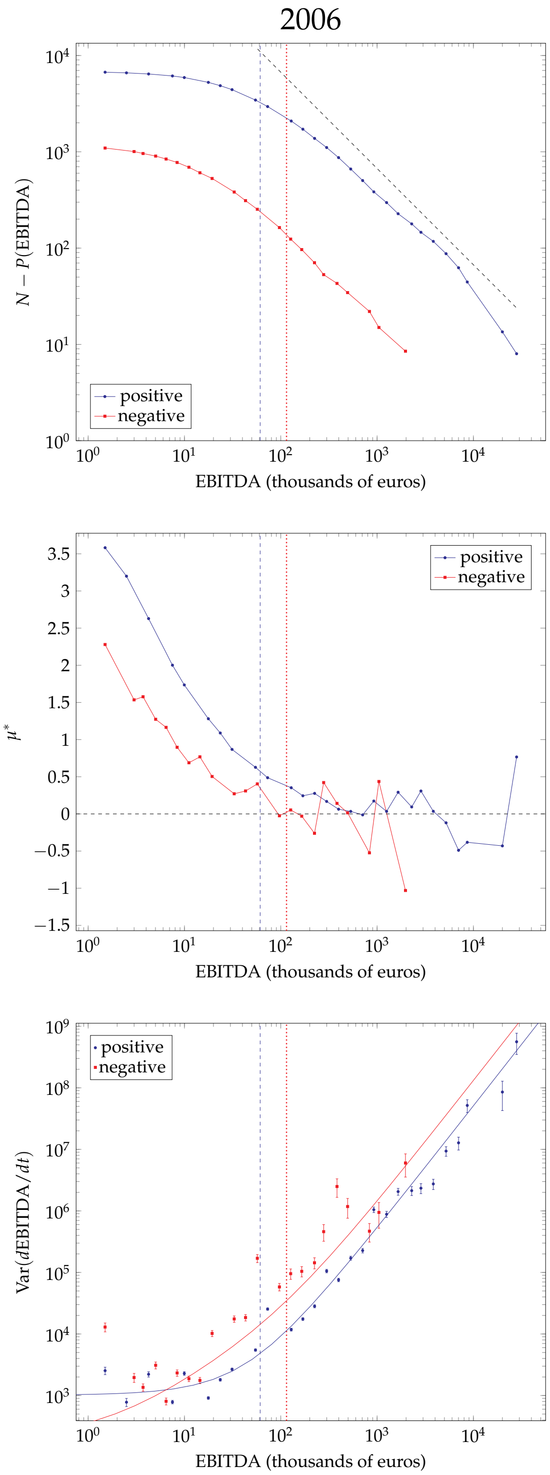

Figure 164: **Navarre 2006: Rank plot, chemical potential and variance**.
Positive EBITDA: 6821 firms.
T_1 = 0.51 ± 0.14, $T_{1/2}$= 31.02 ± 20.54, and T_0 =1011.53 ± 259.33
Negative EBITDA: 1207 firms.
T_1 = 1.31 ± 0.72, $T_{1/2}$= 149.81 ± 78.89, and T_0 =211.85 ± 650.33.
Total active firms 8028, total created firms 1312, and total destroyed firms 712

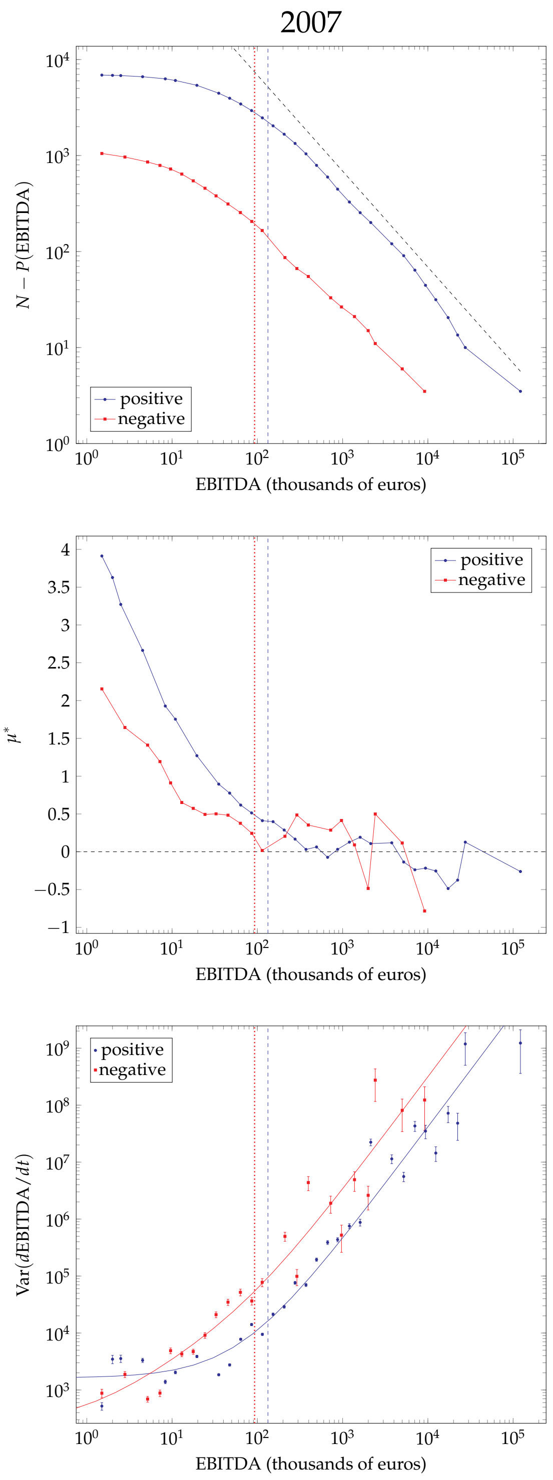

Figure 165: **Navarre 2007: Rank plot, chemical potential and variance**.
Positive EBITDA: 6987 firms.
T_1 = 0.41 ± 0.10, $T_{1/2}$= 54.69 ± 41.17, and T_0 =1629.73 ± 718.52
Negative EBITDA: 1163 firms.
T_1 = 3.12 ± 1.23, $T_{1/2}$= 289.54 ± 167.18, and T_0 =261.05 ± 765.88.
Total active firms 8150, total created firms 555, and total destroyed firms 1207

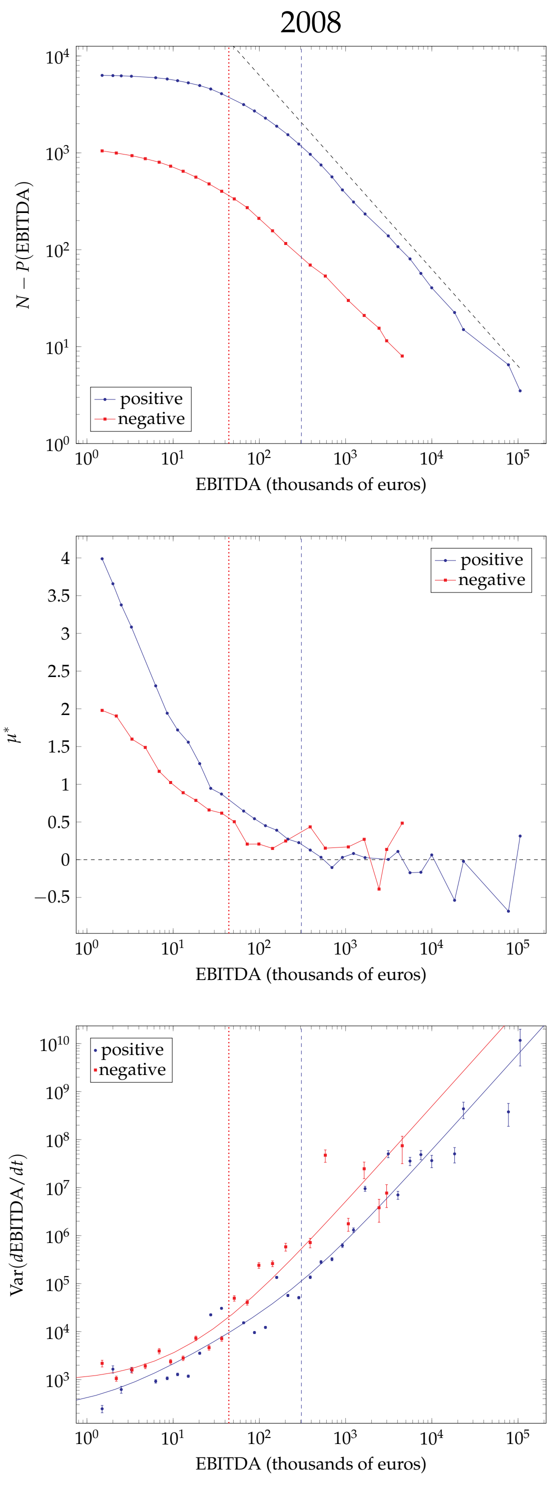

Figure 166: **Navarre 2008: Rank plot, chemical potential and variance.**
Positive EBITDA: 6390 firms.
T_1 = 0.60 ± 0.17, $T_{1/2}$= 183.39 ± 70.05, and T_0 =235.88 ± 351.01
Negative EBITDA: 1137 firms.
T_1 = 4.92 ± 2.11, $T_{1/2}$= 217.26 ± 211.91, and T_0 =938.02 ± 1133.28.
Total active firms 7527, total created firms 2207, and total destroyed firms 1207

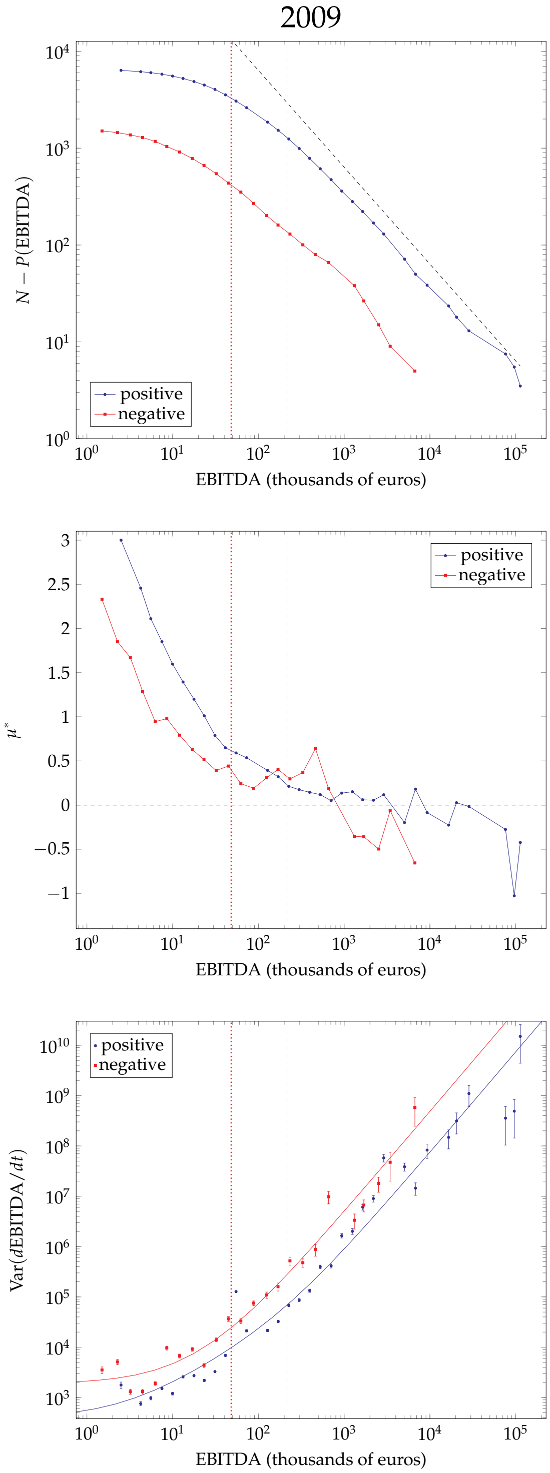

Figure 167: **Navarre 2009: Rank plot, chemical potential and variance.**
Positive EBITDA: 6594 firms.
$T_1 = 0.74 \pm 0.20$, $T_{1/2} = 158.78 \pm 78.73$, and $T_0 = 397.73 \pm 682.50$
Negative EBITDA: 1624 firms.
$T_1 = 4.84 \pm 1.16$, $T_{1/2} = 233.34 \pm 145.78$, and $T_0 = 1878.86 \pm 993.21$.
Total active firms 8218, total created firms 972, and total destroyed firms 752

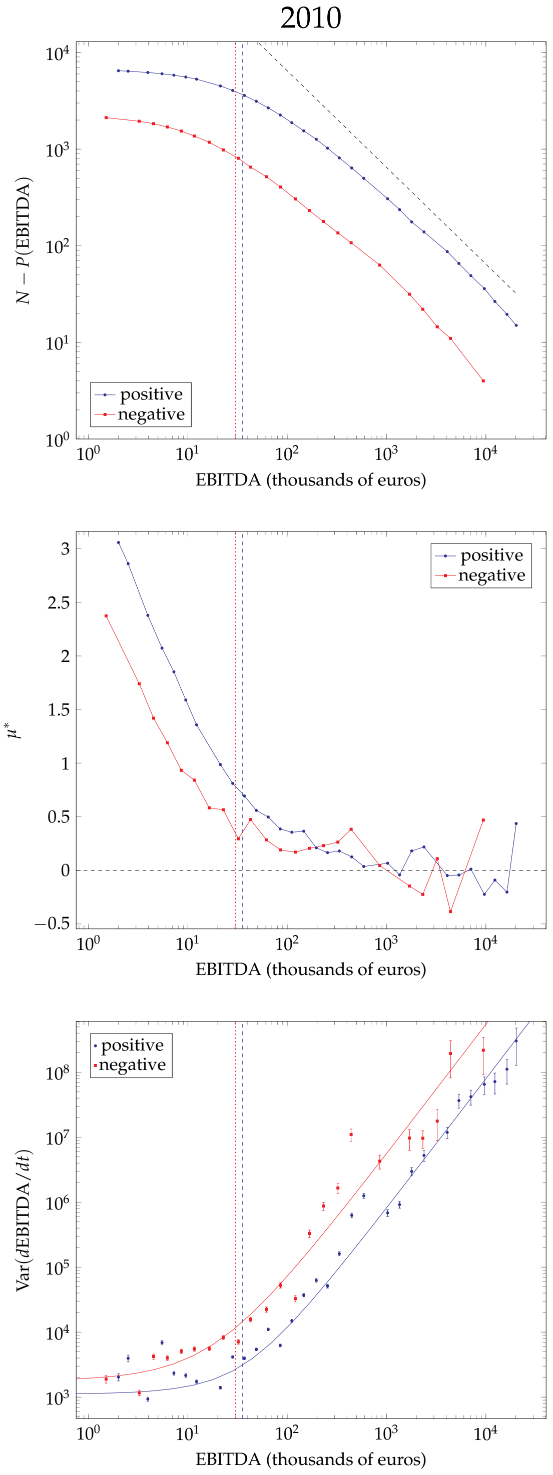

Figure 168: **Navarre 2010: Rank plot, chemical potential and variance.**
Positive EBITDA: 6690 firms.
$T_1 = 0.79 \pm 0.17$, $T_{1/2}= 28.08 \pm 20.16$, and $T_0 =1107.93 \pm 337.68$
Negative EBITDA: 2266 firms.
$T_1 = 5.44 \pm 1.57$, $T_{1/2}= 164.17 \pm 170.74$, and $T_0 =1785.77 \pm 1282.76$.
Total active firms 8956, total created firms 1075, and total destroyed firms 950

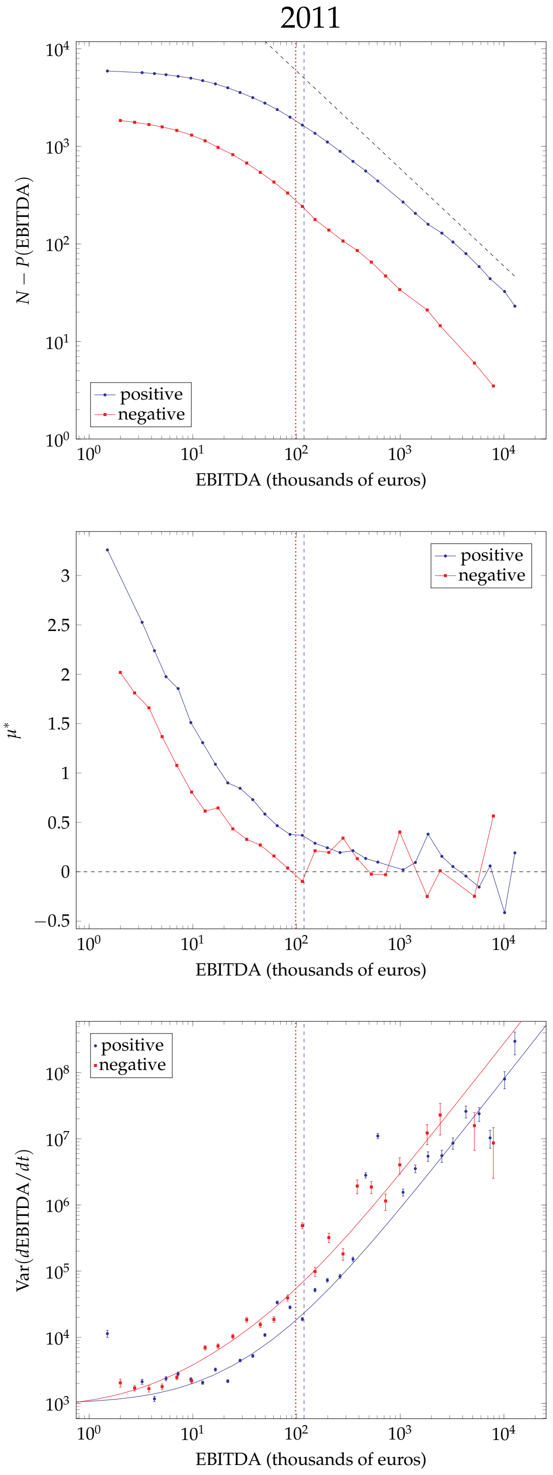

Figure 169: **Navarre 2011: Rank plot, chemical potential and variance.**
Positive EBITDA: 6077 firms.
$T_1 = 0.80 \pm 0.22$, $T_{1/2} = 94.25 \pm 30.70$, and $T_0 = 980.95 \pm 474.29$
Negative EBITDA: 2018 firms.
$T_1 = 2.75 \pm 1.03$, $T_{1/2} = 270.19 \pm 92.56$, and $T_0 = 835.64 \pm 493.70$.
Total active firms 8095, total created firms 562, and total destroyed firms 1056

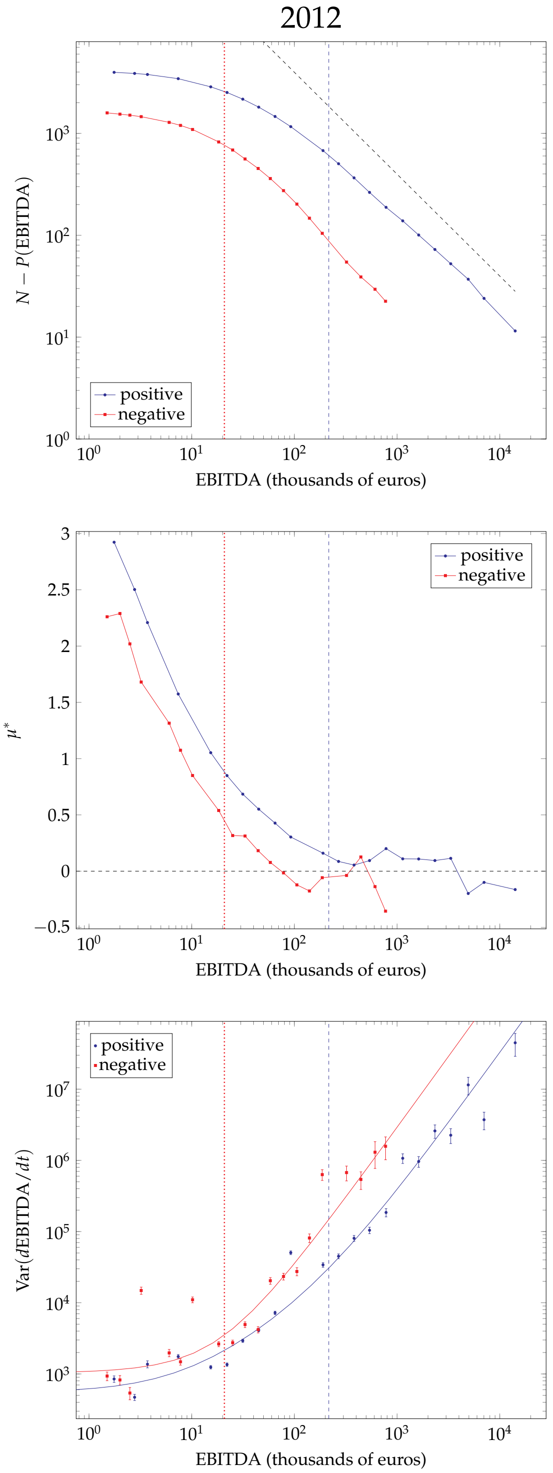

Figure 170: **Navarre 2012: Rank plot, chemical potential and variance.**
Positive EBITDA: 4120 firms.
$T_1 = 0.32 \pm 0.12$, $T_{1/2} = 69.66 \pm 20.38$, and $T_0 = 549.43 \pm 165.62$
Negative EBITDA: 1710 firms.
$T_1 = 2.86 \pm 1.71$, $T_{1/2} = 59.55 \pm 88.00$, and $T_0 = 1026.19 \pm 460.94$.
Total active firms 5830, total created firms 130, and total destroyed firms 3734

Valencian community

Tables of Temperatures

Positive EBITDA

Year	T_1	$T_{1/2}$	T_0	$T_{1/2}/T_1$	Num. Firms
2003	0.44 ± 0.06	52.19 ± 15.51	493.22 ± 147.12	117.9	50870
2004	0.35 ± 0.05	78.95 ± 19.53	218.26 ± 128.06	227.0	52966
2005	0.56 ± 0.10	48.75 ± 8.54	380.42 ± 53.59	86.8	54255
2006	0.70 ± 0.15	51.72 ± 13.57	501.53 ± 87.18	73.8	55714
2007	0.70 ± 0.12	45.13 ± 14.10	675.31 ± 163.73	64.4	57891
2008	1.04 ± 0.21	132.66 ± 24.40	662.64 ± 154.79	127.5	49916
2009	1.19 ± 0.27	75.60 ± 32.49	1473.82 ± 348.10	63.3	49888
2010	0.71 ± 0.08	99.76 ± 23.65	519.73 ± 171.10	139.5	51731
2011	0.75 ± 0.10	198.91 ± 44.53	981.86 ± 371.19	267.0	44563
2012	0.59 ± 0.12	73.49 ± 11.24	307.11 ± 55.85	125.2	28351

Negative EBITDA

Year	T_1	$T_{1/2}$	T_0	$T_{1/2}/T_1$	Num. Firms
2003	2.43 ± 0.55	171.08 ± 414.15	786.12 ± 461.69	70.4	10229
2004	2.70 ± 0.64	267.51 ± 556.85	373.89 ± 406.69	99.2	10911
2005	1.80 ± 0.37	261.02 ± 135.24	195.33 ± 102.34	144.7	11138
2006	2.52 ± 0.69	228.50 ± 266.49	501.65 ± 217.02	90.8	12030
2007	4.04 ± 0.57	224.82 ± 118.27	455.28 ± 168.31	55.7	11926
2008	5.54 ± 1.41	443.30 ± 962.79	1944.13 ± 628.22	80.0	12089
2009	5.01 ± 1.43	255.61 ± 654.80	1193.14 ± 576.26	51.0	17965
2010	3.41 ± 0.51	209.59 ± 421.58	447.97 ± 338.40	61.5	23094
2011	4.62 ± 0.59	342.36 ± 670.28	689.26 ± 472.68	74.2	20526
2012	2.69 ± 0.41	143.78 ± 96.29	478.50 ± 117.23	53.4	14009

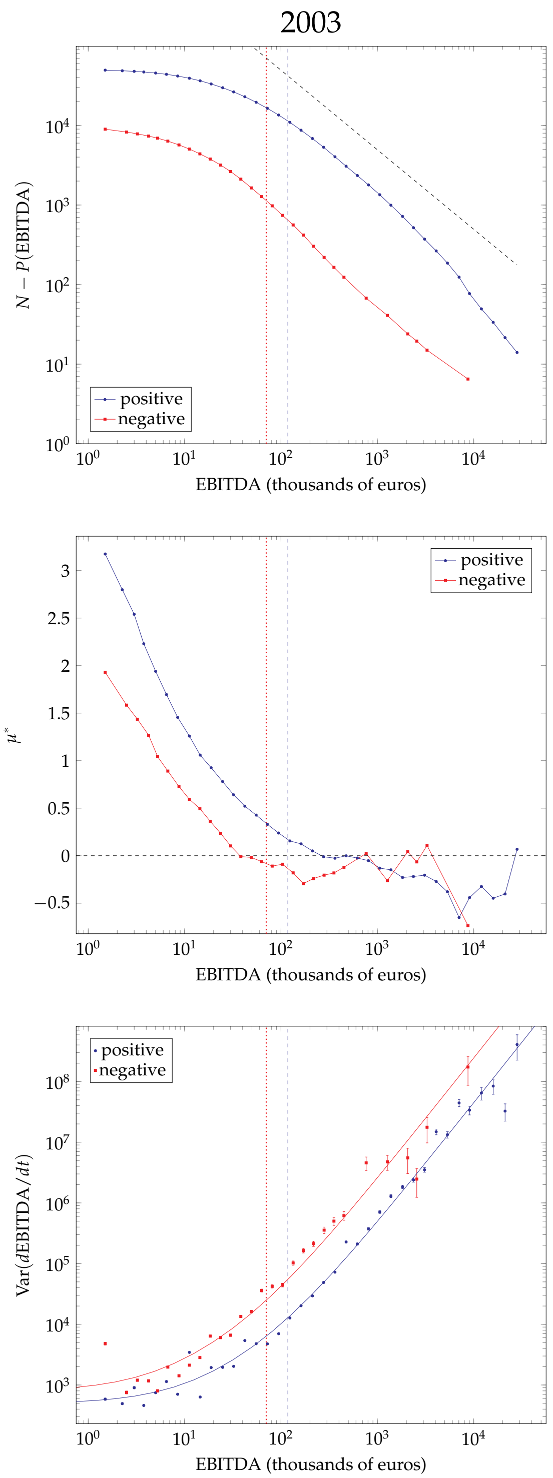

Figure 171: **Valencian community 2003: Rank plot, chemical potential and variance.**
Positive EBITDA: 50870 firms.
T_1 = 0.44 ± 0.06, $T_{1/2}$= 52.19 ± 15.51, and T_0 =493.22 ± 147.12
Negative EBITDA: 10229 firms.
T_1 = 2.43 ± 0.55, $T_{1/2}$= 171.08 ± 70.89, and T_0 =786.12 ± 461.69.
Total active firms 61099, total created firms 13754, and total destroyed firms 3284

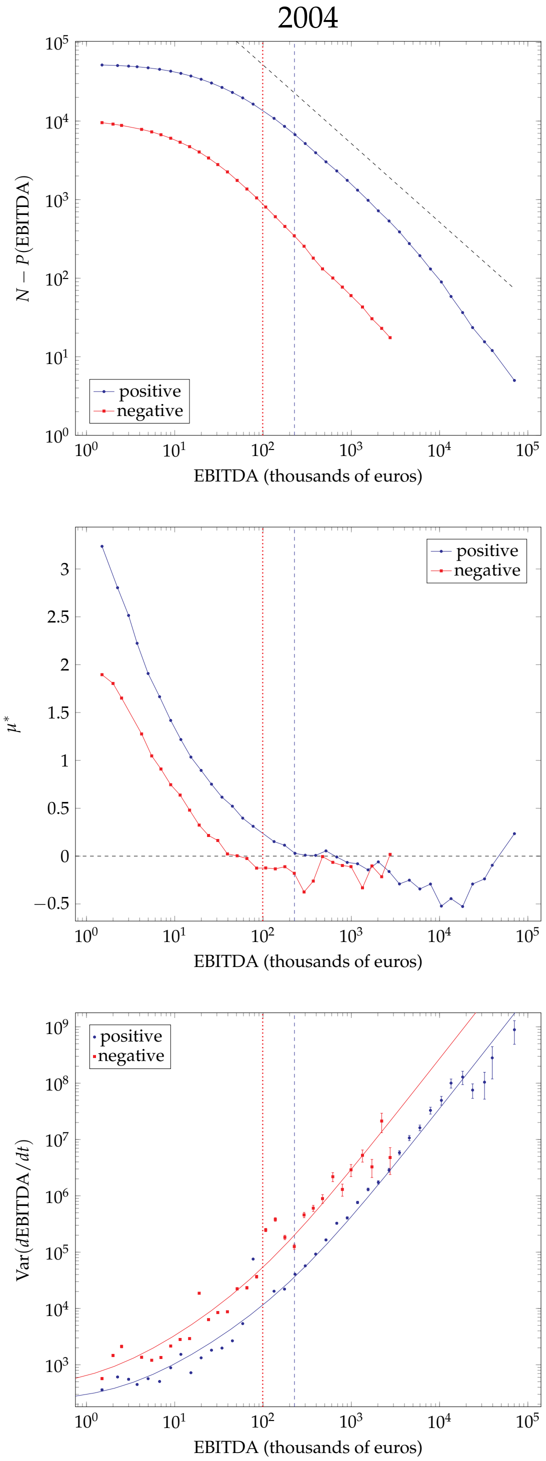

Figure 172: **Valencian community 2004: Rank plot, chemical potential and variance.**
Positive EBITDA: 52966 firms.
T_1 = 0.35 ± 0.05, $T_{1/2}$= 78.95 ± 19.53, and T_0 =218.26 ± 128.06
Negative EBITDA: 10911 firms.
T_1 = 2.70 ± 0.64, $T_{1/2}$= 267.51 ± 87.40, and T_0 =373.89 ± 406.69.
Total active firms 63877, total created firms 11299, and total destroyed firms 10778

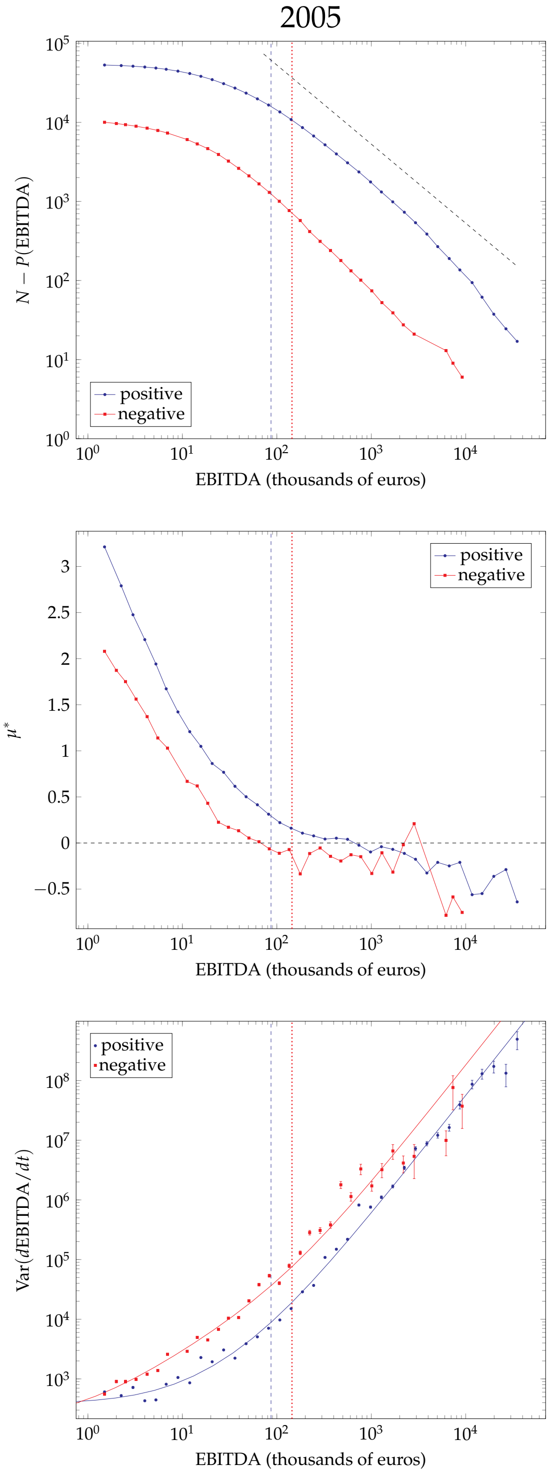

Figure 173: **Valencian community 2005: Rank plot, chemical potential and variance.**
Positive EBITDA: 54255 firms.
T_1 = 0.56 ± 0.10, $T_{1/2}$= 48.75 ± 8.54, and T_0 =380.42 ± 53.59
Negative EBITDA: 11138 firms.
T_1 = 1.80 ± 0.37, $T_{1/2}$= 261.02 ± 32.13, and T_0 =195.33 ± 102.34.
Total active firms 65393, total created firms 11430, and total destroyed firms 10084

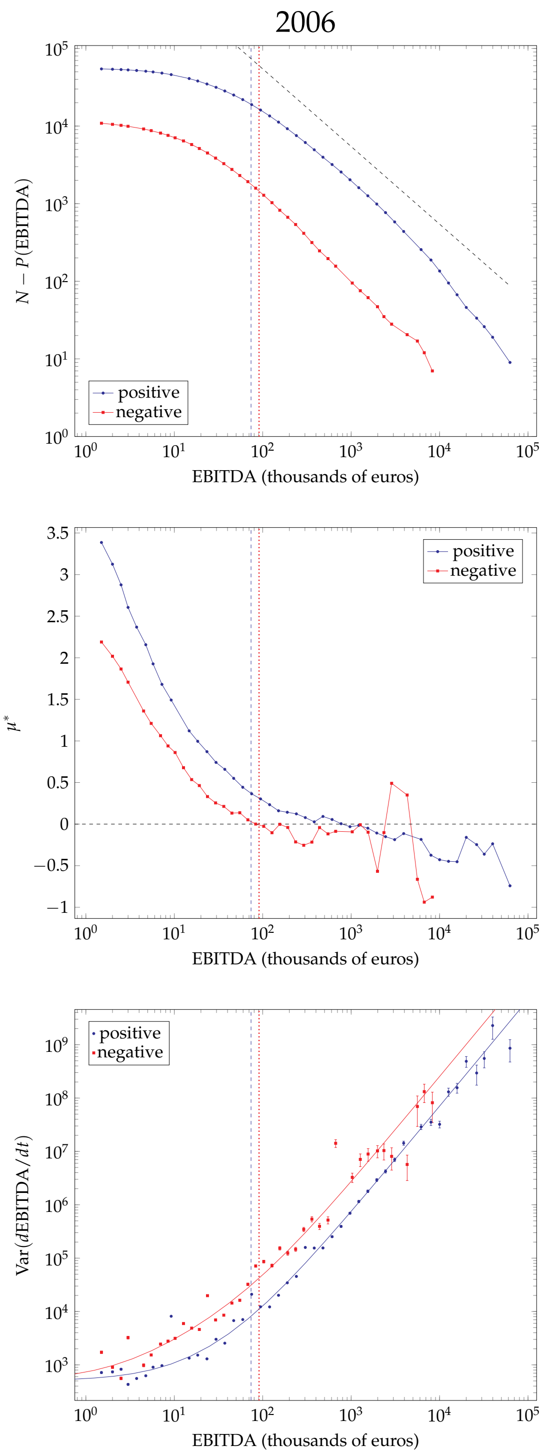

Figure 174: **Valencian community 2006: Rank plot, chemical potential and variance.**
Positive EBITDA: 55714 firms.
$T_1 = 0.70 \pm 0.15$, $T_{1/2} = 51.72 \pm 13.57$, and $T_0 = 501.53 \pm 87.18$
Negative EBITDA: 12030 firms.
$T_1 = 2.52 \pm 0.69$, $T_{1/2} = 228.50 \pm 51.91$, and $T_0 = 501.65 \pm 217.02$.
Total active firms 67744, total created firms 14367, and total destroyed firms 9054

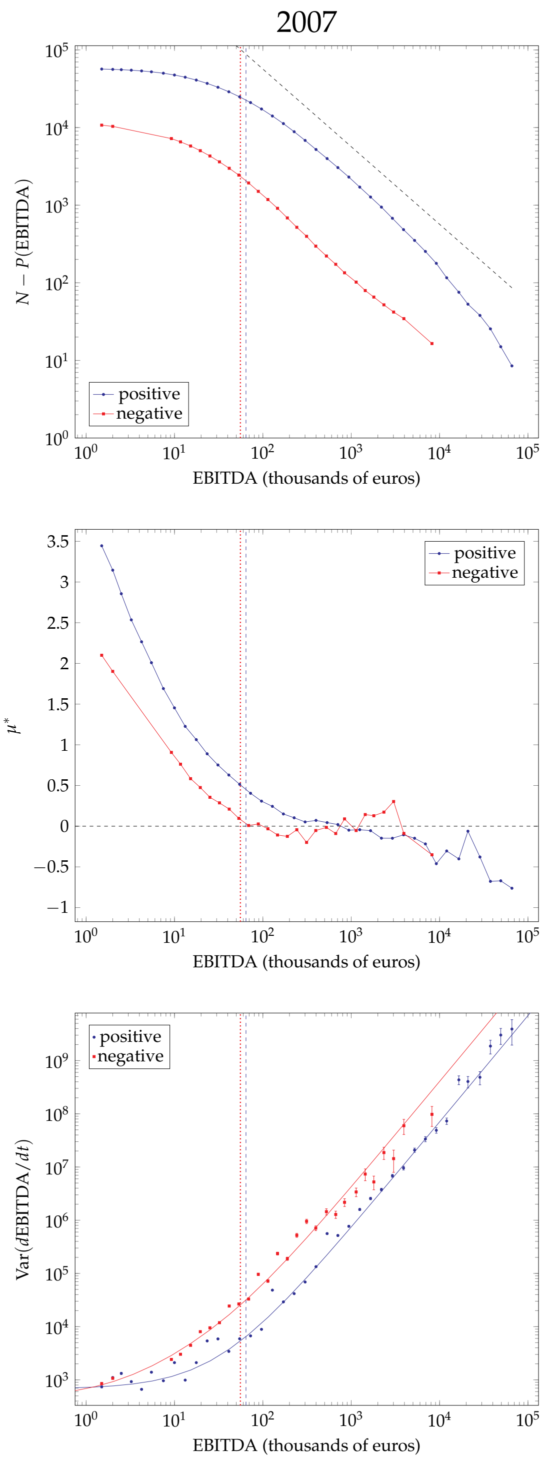

Figure 175: **Valencian community 2007: Rank plot, chemical potential and variance**.
Positive EBITDA: 57891 firms.
T_1 = 0.70 ± 0.12, $T_{1/2}$= 45.13 ± 14.10, and T_0 =675.31 ± 163.73
Negative EBITDA: 11926 firms.
T_1 = 4.04 ± 0.57, $T_{1/2}$= 224.82 ± 29.22, and T_0 =455.28 ± 168.31.
Total active firms 69817, total created firms 4306, and total destroyed firms 12201

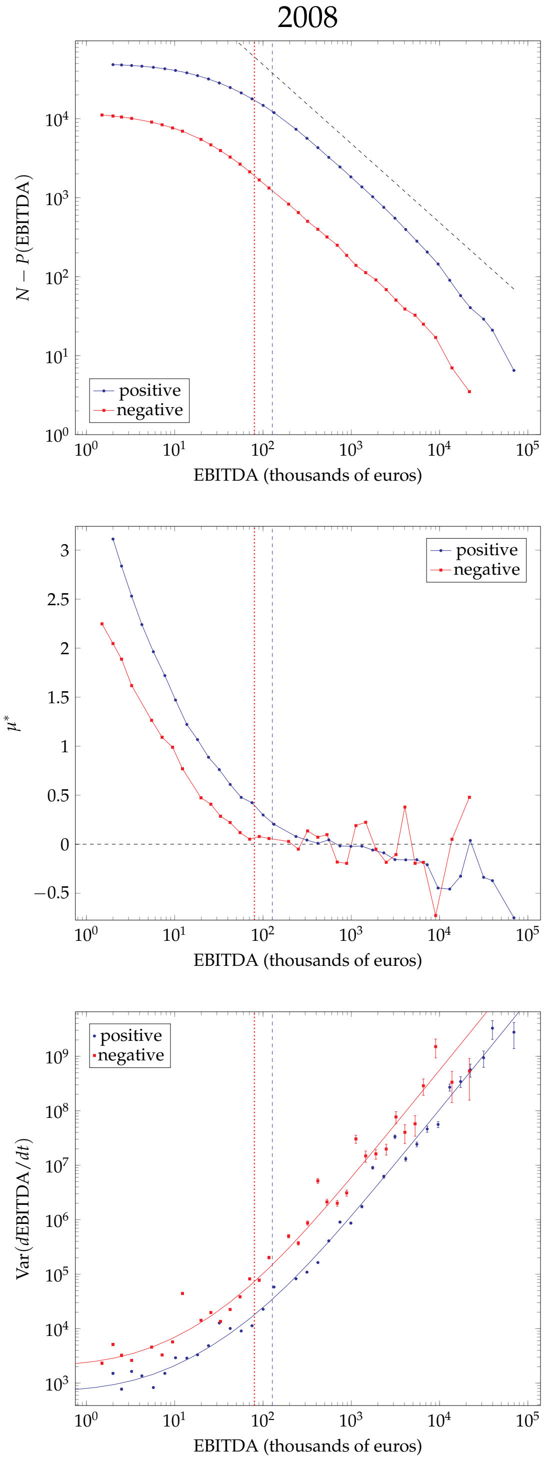

Figure 176: **Valencian community 2008: Rank plot, chemical potential and variance.**
Positive EBITDA: 49916 firms.
T_1 = 1.04 ± 0.21, $T_{1/2}$= 132.66 ± 24.40, and T_0 =662.64 ± 154.79
Negative EBITDA: 12089 firms.
T_1 = 5.54 ± 1.41, $T_{1/2}$= 443.30 ± 128.73, and T_0 =1944.13 ± 628.22.
Total active firms 62005, total created firms 22025, and total destroyed firms 12405

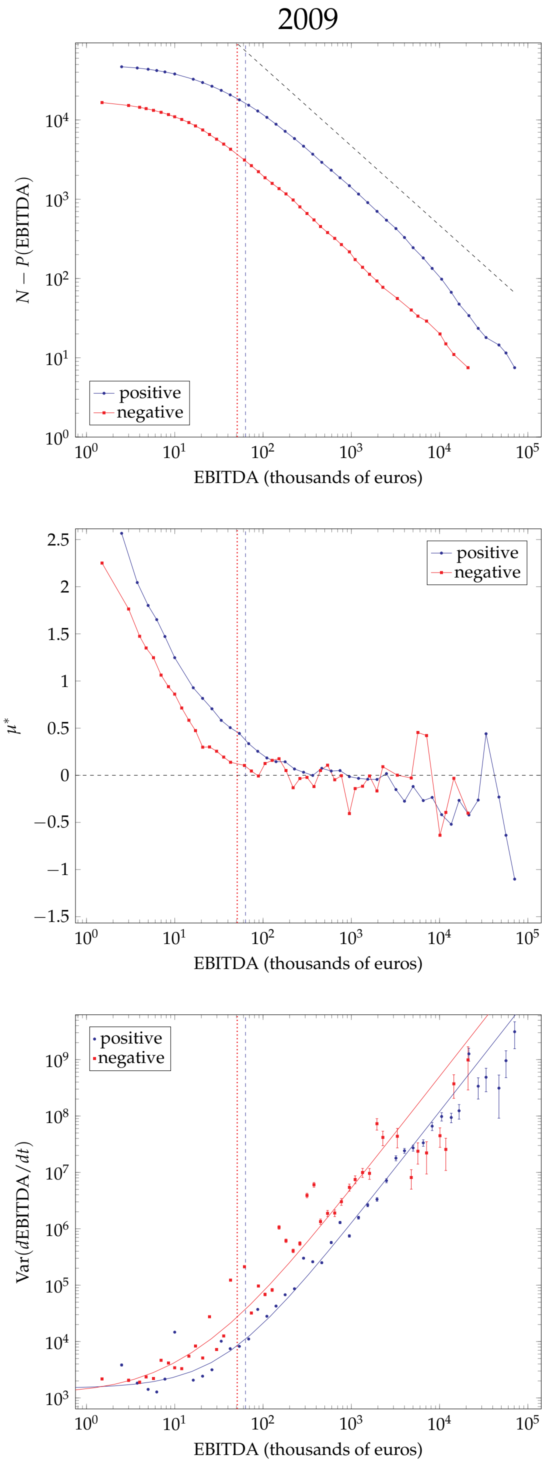

Figure 177: **Valencian community 2009: Rank plot, chemical potential and variance.**
Positive EBITDA: 49888 firms.
T_1 = 1.19 ± 0.27, $T_{1/2}$= 75.60 ± 32.49, and T_0 =1473.82 ± 348.10
Negative EBITDA: 17965 firms.
T_1 = 5.01 ± 1.43, $T_{1/2}$= 255.61 ± 98.01, and T_0 =1193.14 ± 576.26.
Total active firms 67853, total created firms 9015, and total destroyed firms 7367

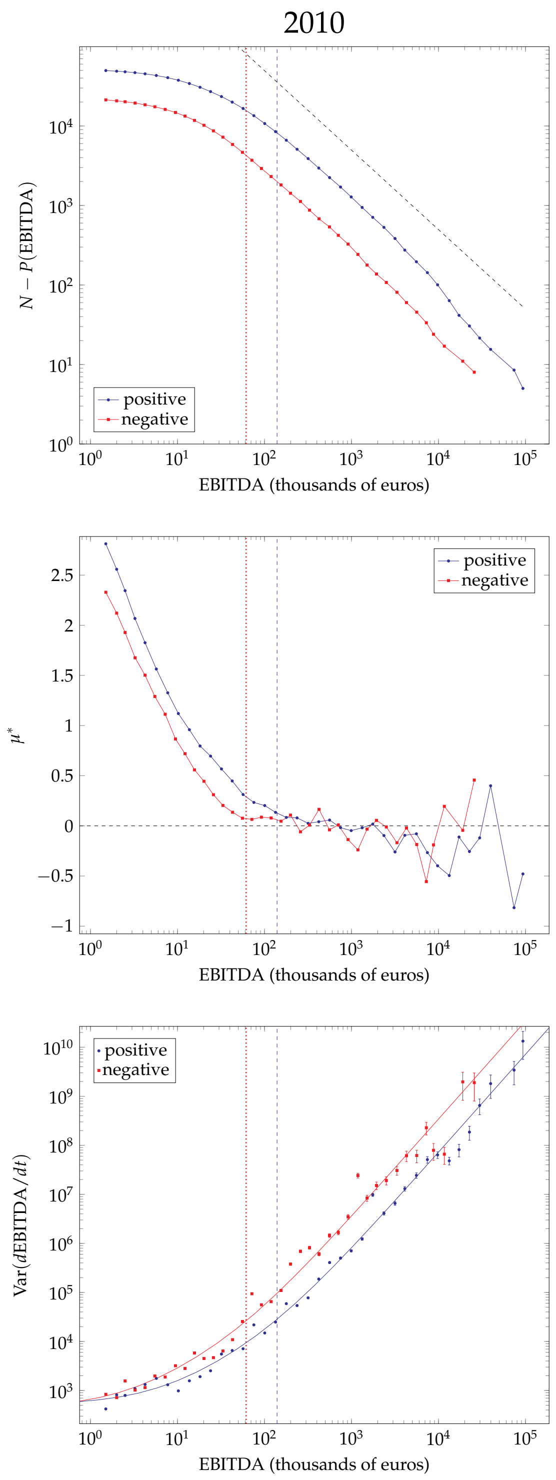

Figure 178: **Valencian community 2010: Rank plot, chemical potential and variance.**
Positive EBITDA: 51731 firms.
T_1 = 0.71 ± 0.08, $T_{1/2}$= 99.76 ± 23.65, and T_0 =519.73 ± 171.10
Negative EBITDA: 23094 firms.
T_1 = 3.41 ± 0.51, $T_{1/2}$= 209.59 ± 71.79, and T_0 =447.97 ± 338.40.
Total active firms 74825, total created firms 7388, and total destroyed firms 10373

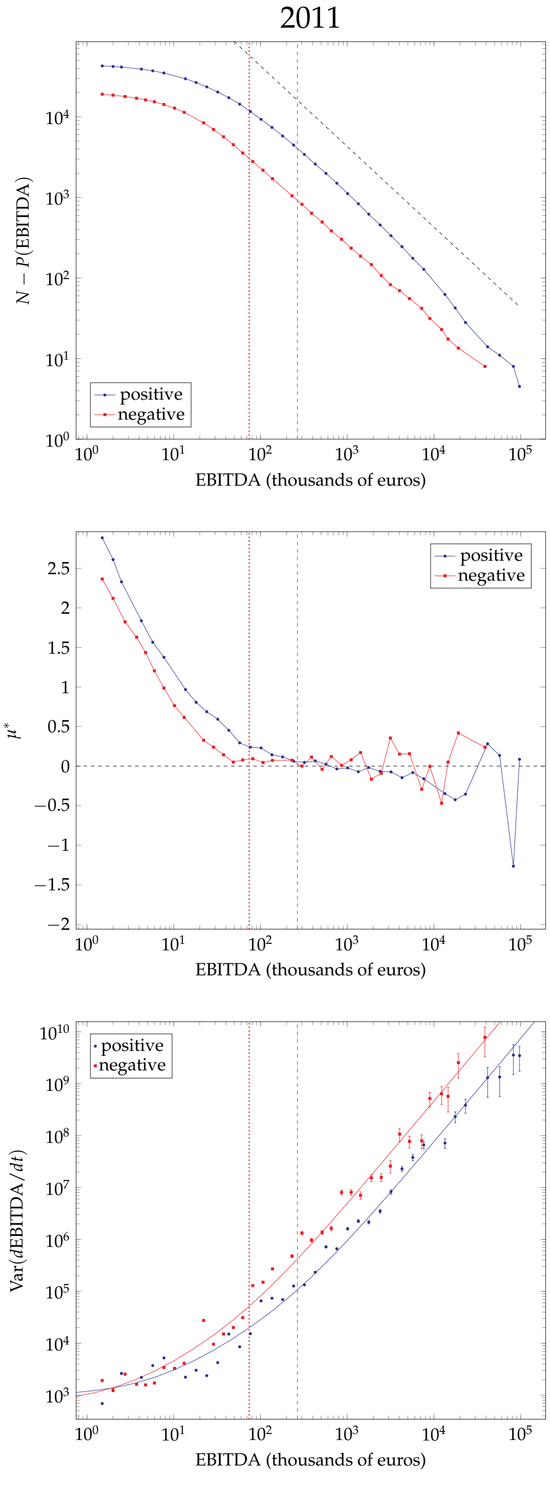

Figure 179: **Valencian community 2011: Rank plot, chemical potential and variance.**
Positive EBITDA: 44563 firms.
T_1 = 0.75 ± 0.10, $T_{1/2}$= 198.91 ± 44.53, and T_0 =981.86 ± 371.19
Negative EBITDA: 20526 firms.
T_1 = 4.62 ± 0.59, $T_{1/2}$= 342.36 ± 99.65, and T_0 =689.26 ± 472.68.
Total active firms 65089, total created firms 3485, and total destroyed firms 11249

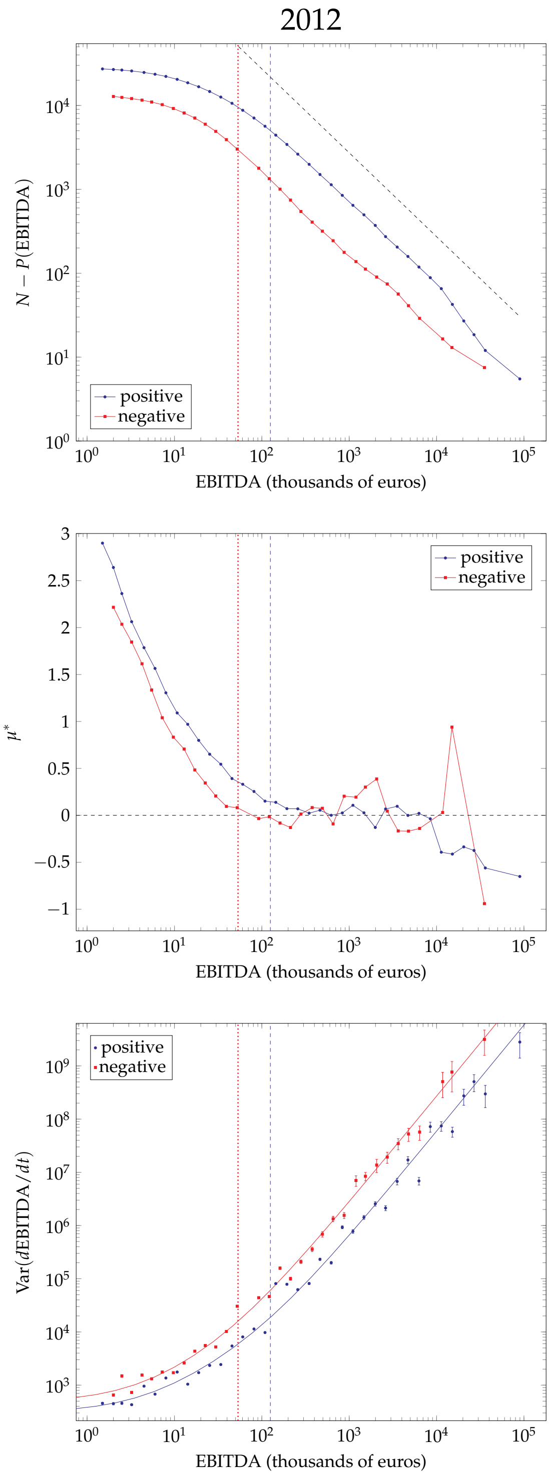

Figure 180: **Valencian community 2012: Rank plot, chemical potential and variance.**
Positive EBITDA: 28351 firms.
T_1 = 0.59 ± 0.12, $T_{1/2}$= 73.49 ± 11.24, and T_0 =307.11 ± 55.85
Negative EBITDA: 14009 firms.
T_1 = 2.69 ± 0.41, $T_{1/2}$= 143.78 ± 25.27, and T_0 =478.50 ± 117.23.
Total active firms 42360, total created firms 797, and total destroyed firms 32650
